# Identification and Expression Profile Analysis of Chemosensory Genes From the Antennal Transcriptome of Bamboo Locust (*Ceracris kiangsu*)

**DOI:** 10.3389/fphys.2020.00889

**Published:** 2020-09-09

**Authors:** Ran Li, Guo-Fang Jiang, Xiao-Han Shu, Yu-Qi Wang, Ming-Jie Li

**Affiliations:** ^1^College of Oceanology and Food Science, Quanzhou Normal University, Quanzhou, China; ^2^College of Plant Protection, Nanjing Agricultural University, Nanjing, China

**Keywords:** *Ceracris kiangsu*, antennal transcriptome, expression profile analysis, chemosensory genes, olfactory

## Abstract

Studies of chemosensory genes are key to a better understanding of intra- and interspecific communications between insects and their environment and provide opportunities for developing environmentally friendly pesticides to target pest species. The bamboo locust *Ceracris kiangsu* Tsai (Orthoptera: Acrididae) is one of the most important bamboo leaf-eating insects in southern China. However, the genes underlying olfactory sensation are lacking in the bamboo locust. In this study, the transcriptomes of male and female *C. kiangsu* antennae were sequenced and analyzed. A total of 125 chemosensory genes, including 91 odorant receptors (ORs), 13 ionotropic receptors (IRs), 13 odorant-binding proteins (OBPs), six chemosensory proteins (CSPs), and two sensory neuron membrane proteins, were identified based on sequence alignment and phylogenetic analyses. The expression patterns of all candidate genes on the antennae of males and females, maxillary palps, tarsi, wings, and thoraxes-abdomens were confirmed by real-time quantitative PCR. The analyses demonstrated that most genes are highly expressed in the antennae, and 35 ORs, 7 IRs, 10 OBPs, and 1 CSP exhibit significantly male-biased expression patterns, indicating their potential functions in mating behavior and the recognition of female sex pheromones. In addition to the antennal-predominant genes, some were abundant in the maxillary palps and some in the non-olfactory tissues, suggesting their different functions in the olfactory system of *C. kiangsu*. Our research offers an extensive resource for investigating the chemoreception mechanism of *C. kiangsu*. Further studies of olfactory function will provide comprehensive methods and original strategies for integrated pest management.

## Introduction

The bamboo locust, *Ceracris kiangsu* Tsai (Orthoptera: Acrididae), which is widely distributed throughout southern China, is one of the most important bamboo leaf-eating insects (Liang and Zheng, 1998). Because of their voracious appetite, wide distribution, destructive feeding habits, and the difficulty in controlling locust infestations, *C. kiangsu* has been called the second largest bamboo pest in China (Cheng et al., 2009). Until now, pesticide-based pest management is still the main strategy and method used for controlling this species, and this has led to damaging impacts on the environment and ecological systems (Chen et al., 1982; Lian et al., 2006, 2007). For this reason, significant efforts need to be made to find an alternative, ecofriendly strategy for controlling this pest.

As with most insects, olfaction plays a key role in many functional aspects of *C. kiangsu*, including mate recognition, oviposition site location, foraging, and avoidance of predators and other dangers (Schiestl, 2010; Leal, 2013; Wyatt, 2014). Previous studies of *C. kiangsu*, however, have focused mainly on behavior (Yu et al., 2010), ecology (Zhang and Zuo, 2005), and phylogeographics (Fan et al., 2014). Shen et al. (2009) first reported the mud-puddling behavior of *C. kiangsu*: their results showed that salt (NaCl) and nitrogen (NH_4_HCO_3_ and NH_4_Cl) in human urine can stimulate the directional movement of *C. kiangsu*. Electroantennogram (EAG) bioassays showed that the responses of *C. kiangsu* toward human urine volatiles were significantly influenced by the duration of human urine fermentation (Shu et al., 2014). Although these reports demonstrated that the chemosensory system could regulate many aspects of biological behavior, their underlying molecular mechanisms still remained unclear.

Insect olfactory proteins involved in the capture of volatiles from signal transduction and the environment include odorant receptors (ORs), ionotropic receptors (IRs), gustatory odorant receptors (GRs), odorant-binding proteins (OBPs), chemosensory proteins (CSPs), and sensory neuron membrane proteins (SNMPs) (Pelosi et al., 2006; Sato and Touhara, 2008). Odorant receptors, GRs, along with IRs are chemosensory membrane proteins located in the receptor neuron membrane, where the odorant signals are transformed into electrical signals (Leal, 2005). The insect chemoreceptor superfamily-ORs were first discovered in the genome of the fruit fly, *Drosophila melanogaster* (Suh et al., 2014). Odorant receptors generally exhibit a high degree of divergence, both within and across species, and are selectively expressed in olfactory neurons at low levels (Renou, 2014). Odorant receptors are expressed in olfactory receptor neurons (ORNs) and can receive a variety of volatile chemicals, including pheromones and general odorants (Leal, 2013).

In contrast, the olfactory receptor co-receptor (ORCO) gene is more conserved across insect orders and is expressed in almost all ORNs at various stages of development (Hallem et al., 2006; Sato and Touhara, 2008). GRs, which have the same membrane topology as ORs, generally detect sugars, salts, carbon dioxide, acidic pH conditions, and bitter compounds. In insects, GRs are also conserved in their sequence and structure and are highly expressed in the gustatory receptor neurons (GRNs) in taste organs (Engsontia et al., 2014; Agnihotri et al., 2016). ioNotropic receptors are ligand-gated ion channels that evolved from ionotropic glutamate receptors (iGluRs), but with three transmembrane domains (TMDs; Abuin et al., 2011). Insect IRs have been further classified into two subfamilies: conserved “antennal IRs” that play a role in olfaction function and species-specific “divergent IRs” that might be involved in taste (Rytz et al., 2013; Koh et al., 2014). Odorant-binding proteins and CSPs are regarded as the first step in the transportation of hydrophobic odorants in olfactory recognition (Fan et al., 2011). These two proteins are small soluble proteins, highly abundant in the sensillum lymph of the chemosensilla (Sánchez-Gracia et al., 2009). Odorant-binding proteins generally contain six highly conversed cysteine residues that are paired with three interlocking disulfide bridges to maintain a compact and conserved structure (Pelosi et al., 2006). When the odor molecules are detected, OBPs will specifically bind and transport them through the hydrophilic lymph in the sensillum to the membrane of olfactory sensory neuron (OSN) dendrites (Wang et al., 2016). CSPs contain only four conserved cysteine residues and are more conserved across insect species. Compared with OBPs, CSPs are present in more chemosensory organs and even non-chemosensory organs, which are involved in various physiological activities, acting as carriers (Sun et al., 2016). Sensory neuron membrane proteins, the members of the CD36 receptor family, are located in the dendritic membranes of pheromone-sensitive neurons (Forstner et al., 2008). These proteins are also essential for binding and transporting hydrophobic ligands.

To better understand the molecular mechanism of olfactory perception, the first step is to investigate the chemosensory receptor genes, which encode the proteins that function in odorant molecular detection. Only a few chemosensory genes (7 OBPs) of *C. kiangsu* were identified in our previous study (Li et al., 2018). This is much lower than for other grasshopper species, such as *Locusta migratoria* (Ban et al., 2003; Yu et al., 2009; Li et al., 2016) and *Oedaleus asiaticus* (Zhang et al., 2015), from which chemosensory genes have been obtained. In this study, we performed transcriptome sequencing on the antennae of *C. kiangsu*, with the aim of: (1) obtaining more chemosensory receptor genes, (2) revealing the homologous relationships of all chemosensory receptor genes of *C. kiangsu* with other insect gene sets utilizing phylogenetic analyses, and (3) examining the expression profiles of these receptors in various tissues of both sexes using real-time quantitative PCR (qRT-PCR).

## Materials and Methods

### Insect Culture and Tissue Collection

All nymph specimens of *C. kiangsu* were collected from Zijin Mountain in Nanjing, Jiangsu Province, China. Nymphs of different sexes were kept separately, and reared in the laboratory with moso bamboo at 26 ± 2°C under a photoperiod of 14 h light/10 h dark. The male or female individuals were collected 3–6 days after eclosion for subsequent analyses. Male antennae (MA), female antennae (FA), and the remaining body parts (maxillary palps, tarsi, wings, and thoraxes-abdomens) of locusts were rapidly dissected under a microscope. Approximately 50 antennae, 100 maxillary palps, and six bodies each of male and female insects were collected for RNA extraction, and three biological replicates were performed. All tissue samples were then immediately frozen in liquid nitrogen, and stored at −80°C for subsequent RNA extraction.

### RNA Isolation

Total RNA was extracted from all tissue samples using TRIzol reagent (Invitrogen, Carlsbad, CA, United States) according to the manufacturer’s protocol. Total RNA was dissolved in RNase-free water, and the RNA integrity was detected using an Agilent Bioanalyzer 2100 system (Agilent Technologies, CA, United States). RNA degradation and contamination were monitored by 1% agarose gel electrophoresis. The purity and concentration of isolated RNA samples were determined on a Nanodrop ND-2000 spectrophotometer (Thermo Fisher Scientific, Waltham, MA, United States).

### cDNA Library Construction and Transcriptome Sequencing

A total weight of 3 μg of RNA per sample from male and female antennae was used as input material and to construct two cDNA libraries separately. The libraries were constructed using a TruseqTM RNA sample prep Kit (Illumina, San Diego, CA, United States) and sequenced on the Illumina HiSeq 2500 platform (Illumina, San Diego, CA, United States) using the pair-ends strategy.

### *De novo* Transcriptome Analysis

Clean reads were obtained from the raw reads after filtering the low-quality reads, discarding unknown or low-quality bases, and removing adaptors and poly-A/T tails. A transcriptome assembly was conducted based on clean reads using the short reads assembly program, Trinity (v2.4.0) with a minimum k-mer coverage of 3 (Grabherr et al., 2011). The outputs were then clustered to eliminate redundancy and generate longer consensus transcript sequences by TGICL (v2.1) (Pertea et al., 2003). The consensus cluster sequences make up the final unigenes dataset, which consisted of the longest transcript of each gene. BLASTx and BLASTn alignment with an E-value threshold of 1 × 10^–5^ was then performed between unigenes and protein databases. In addition, the Blast2go pipeline was used to determine the gene ontology (GO) annotations of the unigenes (Conesa et al., 2005).

### Identification of Putative Chemosensory Genes

Candidate unigenes encoding putative chemosensory genes (ORs, IRs, OBPs, CSPs, and SNMPs, no found GRs) were identified according to the results of non-redundant protein (Nr) annotation from our antennal transcriptome dataset. We used “OR and odorant receptor,” “IR and ionotropic receptor,” “OBP and odorant-binding protein,” “CSP and chemosensory protein,” and “SNMP and sensory neuron membrane protein” as key words to screen the annotated sequences. All of the candidate chemosensory genes were manually checked using BLASTx and BLASTn searches (E-value < 10^–5^). Sequence alignments were performed using the ClustalX 2.1 program^1^ with default parameters (Larkin et al., 2007). The open reading frames (ORFs) of all putative chemosensory proteins were determined using the ExPASy (Expert Protein Analysis System) server^2^ (Gasteiger et al., 2003). The TMDs of putative olfactory genes (ORs and IRs) were predicted using the TMHMM server^3^ (Krogh et al., 2001). Putative N-terminal signal peptides of odorant transport proteins (OBPs and CSPs) were predicted using the SignalP 4.1 program^4^ using default parameters (Petersen et al., 2011).

### Phylogenetic Analysis

Phylogenetic analyses were performed based on amino acid sequences from candidate chemosensory genes from *C. kiangsu* and other insects. The sequences were aligned using MAFFT^5^, with the E-INS-I parameter set (Katoh and Standley, 2013), and are presented in Supplementary Table S1. Phylogenetic trees were constructed using the method of maximum likelihood with the Jones–Taylor–Thornton (JTT) model in the MEGA 7 software^6^ (Kumar et al., 2016). For an accurate tree, 1000 bootstrap replicates were created as the node support. Lastly, all phylogenetic trees were visualized using EvolView^7^ (He et al., 2016) and subsequently edited using the FigTree program^8^ (Rambaut, 2018).

### Tissue Expression Profile Analysis

Real-time quantitative PCR was performed in order to verify the expression patterns of candidate chemosensory genes using an ABI 7300 Fast Real-Time PCR System (Applied Biosystems, Foster City, CA, United States). Different tissues, including male antennae, female antennae, maxillary palps, tarsi, wings, and thoraxes-abdomens, were collected from the locusts. Total RNA was isolated using the methods described above and reverse transcribed into cDNA using the first-strand cDNA FastQuant RT Kit (with gDNase) (TIANGEN Biotech (Beijing) Co., Ltd., China).

Real-time quantitative PCR was conducted in a 20 μL reaction system, containing 10 μL Taq SYBR^®^Green qPCR Premix (2×), 0.2 μL ROX Reference DyeI (100×), 0.4 μL each of the forward and reverse primers (10 μM), 1.2 μL cDNA template, and 7.8 μL deionized water. The thermal cycling was set to be 1 cycle at 94°C for 3 min, followed by 40 cycles at 94°C for 15 s and 60°C for 1 min. To determine the reproducibility, each reaction for each tissue was performed in three biological replicates and three technical replicates. U6 was used as the reference gene for normalizing the expression of various samples (Li et al., 2018). Gene-specific primers employed in qRT-PCR were designed by Primer Premier 5 software^9^ (Lalitha, 2000). Only primers with a single PCR amplification product were used in the downstream analyses and are listed in Supplementary Table S2. The amplification efficiency of each primer was calculated from the slope of the standard curve (Kubista et al., 2006).

Relative quantification was calculated using the comparative 2^–ΔΔCT^ method (Livak and Schmittgen, 2001). The comparative analyses of target genes between different tissues were subjected to one-way analysis of variance (ANOVA) using SPSS 22.0 software (SPSS Inc., Chicago, IL, United States) (*P* < 0.05), followed by least-significant difference (LSD).

## Results

### Overview of the Transcriptome Sequencing

To identify the chemosensory receptor genes of *C. kiangsu*, the transcriptome sequencing of male and female antennae were completed separately. Approximately 134.3 million and 137.2 million raw reads and a total of 131.3 million and 134.4 million clean reads were generated in male and female antennae, respectively. In addition, the Q30 base percentage of all three biological replicates exceeded 93.10% and the Q20 base percentage exceeded 97.50% (Supplementary Table S3). After the reads from all samples were assembled into a single transcriptome, a total of 39,166 unigenes with a mean length of 1498 bp and an N50 of 2259 bp were screened from 63,631 transcripts. Length distribution analysis showed that the maximum length of the unigenes was 35,159 bp, and 28,517 (72.81% of all unigenes) were longer than 500 bp in size (Supplementary Table S4). The raw reads were deposited at the National Center for Biotechnology Information (NCBI) – Sequence Read Archive (SRA) database with the submission numbers SRR11364396 and SRR11364401.

**FIGURE 1:**
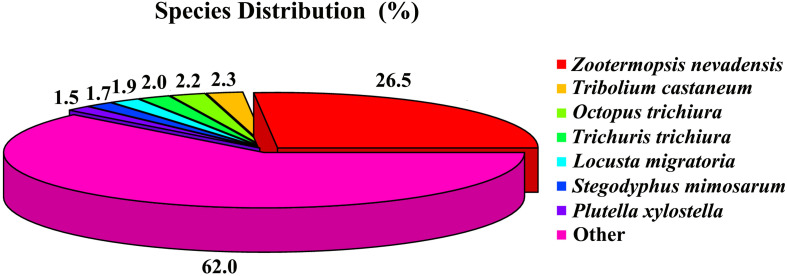
Insect species distribution of *C. kiangsu* unigenes’ best-hit annotation term in NR database.

For the function annotations of the database from the *C. kiangsu* transcriptome, a total of 23,241 unigenes (59.34%) were successfully annotated through diverse protein datasets, including NCBI Nr, NCBI nucleotide sequences (Nt), GO, clusters of orthologous groups (COG), Kyoto Encyclopedia of Genes and Genomes (KEGG), a manually annotated and reviewed protein sequence database (Swiss-Prot), and Protein family (Pfam) (Supplementary Table S5). Among the unigenes, 19,438 (49.63%) were matched to the database of Nr with the Blastx algorithm (cut-off E-value of 10^–5^). As shown in Figure 1, the best match species was *Zootermopsis nevadensis*, which covered 13,872 (26.47%) of the annotated unigenes.

### Identification and Analysis of Putative ORs

**FIGURE 2:**
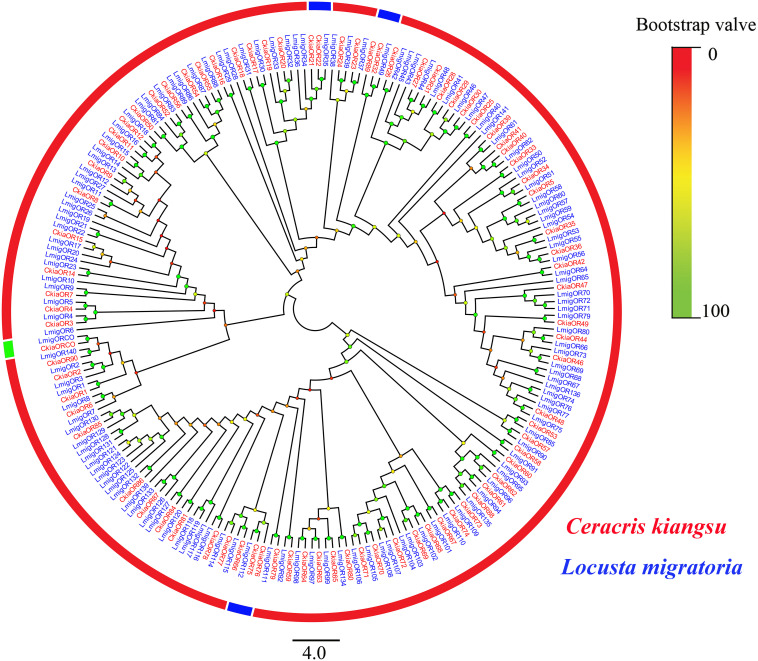
Phylogenetic tree of putative odorant receptors (ORs) from *C. kiangsu* and other insects. Branch support (circles at the branch nodes) was estimated using bootstrap values based on the scale indicated on the top right.

A total of 91 candidate ORs (*CkiaOBP1-90*, *CkiaORCO*) were identified in the antennae transcriptome of *C. kiangsu* by keyword search of the BLASTx annotation (Table 1). The putative chemosensory genes were submitted to GenBank (accession numbers: MT072553–MT072666). Sequence analysis revealed that 59 ORs were predicted to have full-length ORFs that encoded 300–486 amino acids, and four genes (*CkiaOR18*, *CkiaOR50*, *CkiaOR89*, and *CkiaORCO*) contained seven TMDs (Table 1). To identify the ORs in *C. kiangsu*, the putative proteins were phylogenetically analyzed with known ORs of *L.migratoria* (Figure 2). The *C. kiangsu* OR co-receptor, named *CkiaORCO*, shared the highest identity (95% with *LmigORCO*) with the conserved insect co-receptor in other orthopteran species. For the *CkiaOR*s, 85 genes showed an orthologous relationship with *LmigOR*, whereas the other six genes (*CkiaOR21*/*CkiaOR22*, *CkiaOR32*/*CkiaOR89*, and *CkiaOR75*/*CkiaOR76*) showed a 2:1 orthologous relationship with *LmigOR35*, *LmigOR49*, and *LmigOR112*, respectively.

**TABLE 1:** Summary of putative odorant receptors (ORs) identified in *C. kiangsu*.

Gene name	Length (nt)	ORF (aa)	ORF status	Tm domain	Best blastx match			
					Gene ID	Gene name	Species	Similarity (%)
*CkiaOR1*	1371	457	Complete	6	KP843273	Odorant receptor 1	*Locusta migratoria*	85
*CkiaOR2*	1338	445	Complete	5	KP843269	Odorant receptor 2	*Locusta migratoria*	89
*CkiaOR3*	1248	415	Complete	4	KP843253	Odorant receptor 4	*Locusta migratoria*	83
*CkiaOR4*	1278	426	Complete	3	KP843365	Odorant receptor 5	*Locusta migratoria*	87
*CkiaOR5*	1239	413	Complete	2	KF601292	Odorant receptor 6	*Locusta migratoria*	84
*CkiaOR6*	1308	436	Complete	5	KP843263	Odorant receptor 8	*Locusta migratoria*	85
*CkiaOR7*	909	303	5′	5	KP843310	Odorant receptor 9	*Locusta migratoria*	86
*CkiaOR8*	1149	382	Complete	5	KP843352	Odorant receptor 11	*Locusta migratoria*	84
*CkiaOR9*	1260	419	Complete	3	KP843348	Odorant receptor 13	*Locusta migratoria*	81
*CkiaOR10*	1341	446	Complete	4	KP843322	Odorant receptor 15	*Locusta migratoria*	88
*CkiaOR11*	1239	412	Complete	3	KP843234	Odorant receptor 16	*Locusta migratoria*	89
*CkiaOR12*	879	293	5′ lost	4	KP843295	Odorant receptor 18	*Locusta migratoria*	86
*CkiaOR13*	888	296	3′ lost	4	KP843329	Odorant receptor 21	*Locusta migratoria*	85
*CkiaOR14*	1215	404	Complete	2	KP843323	Odorant receptor 23	*Locusta migratoria*	85
*CkiaOR15*	1092	364	5′, 3′ lost	2	KP843345	Odorant receptor 24	*Locusta migratoria*	93
*CkiaOR16*	1311	436	Complete	6	KP843354	Odorant receptor 29	*Locusta migratoria*	78
*CkiaOR17*	1284	428	Complete	4	KP843304	Odorant receptor 30	*Locusta migratoria*	84
*CkiaOR18*	1293	431	Complete	7	KP843247	Odorant receptor 31	*Locusta migratoria*	84
*CkiaOR19*	1026	342	3′ lost	5	KP843286	Odorant receptor 32	*Locusta migratoria*	81
*CkiaOR20*	1209	403	Complete	6	KP843278	Odorant receptor 33	*Locusta migratoria*	81
*CkiaOR21*	1236	412	5′ lost	5	KP843363	Odorant receptor 34	*Locusta migratoria*	78
*CkiaOR22*	867	289	5′, 3′ lost	4	KP843355	Odorant receptor 35	*Locusta migratoria*	81
*CkiaOR23*	1275	424	Complete	6	KP843327	Odorant receptor 37	*Locusta migratoria*	83
*CkiaOR24*	1272	424	Complete	6	KP843237	Odorant receptor 39	*Locusta migratoria*	87
*CkiaOR25*	1227	408	Complete	5	KP843196	Odorant receptor 40	*Locusta migratoria*	88
*CkiaOR26*	1254	418	Complete	4	KP843326	Odorant receptor 42	*Locusta migratoria*	84
*CkiaOR27*	900	300	Complete	3	KP843275	Odorant receptor 44	*Locusta migratoria*	86
*CkiaOR28*	1194	398	3′ lost	5	KY964962	Odorant receptor 45	*Locusta migratoria*	85
*CkiaOR29*	1284	427	Complete	4	KP843249	Odorant receptor 46	*Locusta migratoria*	87
*CkiaOR30*	1347	448	Complete	6	KP843231	Odorant receptor 47	*Locusta migratoria*	91
*CkiaOR31*	1293	430	Complete	4	KP843245	Odorant receptor 48	*Locusta migratoria*	85
*CkiaOR32*	1290	430	Complete	5	KP843251	Odorant receptor 49	*Locusta migratoria*	85
*CkiaOR33*	1236	411	Complete	5	KP843367	Odorant receptor 50	*Locusta migratoria*	83
*CkiaOR34*	1230	410	Complete	6	KP843350	Odorant receptor 51	*Locusta migratoria*	86
*CkiaOR35*	1188	396	Complete	3	KP843316	Odorant receptor 53	*Locusta migratoria*	86
*CkiaOR36*	1368	456	Complete	5	KP843313	Odorant receptor 56	*Locusta migratoria*	81
*CkiaOR37*	567	189	3′ lost	3	KP843340	Odorant receptor 57	*Locusta migratoria*	87
*CkiaOR38*	813	271	3′ lost	4	KP843351	Odorant receptor 60	*Locusta migratoria*	86
*CkiaOR39*	1305	435	Complete	6	KP843248	Odorant receptor 61	*Locusta migratoria*	84
*CkiaOR40*	1266	421	Complete	5	KP843366	Odorant receptor 62	*Locusta migratoria*	88
*CkiaOR41*	1152	384	3′ lost	4	KP843243	Odorant receptor 63	*Locusta migratoria*	87
*CkiaOR42*	1233	411	Complete	2	KP843361	Odorant receptor 64	*Locusta migratoria*	88
*CkiaOR43*	543	181	3′ lost	3	KP843337	Odorant receptor 65	*Locusta migratoria*	86
*CkiaOR44*	1092	364	3′ lost	2	KP843265	Odorant receptor 66	*Locusta migratoria*	88
*CkiaOR45*	1113	371	3′ lost	6	KP843333	Odorant receptor 67	*Locusta migratoria*	82
*CkiaOR46*	1224	407	Complete	6	KP843299	Odorant receptor 69	*Locusta migratoria*	86
*CkiaOR47*	1254	417	Complete	4	KP843266	Odorant receptor 70	*Locusta migratoria*	82
*CkiaOR48*	522	174	3′ lost	0	KP843360	Odorant receptor 75	*Locusta migratoria*	86
*CkiaOR49*	1122	374	3′ lost	5	KP843282	Odorant receptor 79	*Locusta migratoria*	83
*CkiaOR50*	1305	434	Complete	7	KP843341	Odorant receptor 81	*Locusta migratoria*	88
*CkiaOR51*	690	230	3′ lost	0	KP843336	Odorant receptor 82	*Locusta migratoria*	92
*CkiaOR52*	1299	433	Complete	6	KP843257	Odorant receptor 83	*Locusta migratoria*	87
*CkiaOR53*	804	268	5′ lost	2	KP843252	Odorant receptor 85	*Locusta migratoria*	90
*CkiaOR54*	1269	423	Complete	6	KP843240	Odorant receptor 87	*Locusta migratoria*	86
*CkiaOR55*	765	255	3′ lost	0	KP843346	Odorant receptor 88	*Locusta migratoria*	84
*CkiaOR56*	1038	346	5′, 3′ lost	2	KP843305	Odorant receptor 89	*Locusta migratoria*	90
*CkiaOR57*	1197	399	3′ lost	5	KP843246	Odorant receptor 90	*Locusta migratoria*	89
*CkiaOR58*	1341	446	Complete	0	KP843314	Odorant receptor 91	*Locusta migratoria*	82
*CkiaOR59*	1260	419	Complete	6	KP843261	Odorant receptor 92	*Locusta migratoria*	88
*CkiaOR60*	1178	392	3′ lost	4	KP843319	Odorant receptor 93	*Locusta migratoria*	80
*CkiaOR61*	1362	453	Complete	3	KP843364	Odorant receptor 94	*Locusta migratoria*	81
*CkiaOR62*	927	308	5′ lost	3	KP843235	Odorant receptor 96	*Locusta migratoria*	83
*CkiaOR63*	1299	432	Complete	6	KP843256	Odorant receptor 97	*Locusta migratoria*	87
*CkiaOR64*	1272	424	3′ lost	6	KP843339	Odorant receptor 98	*Locusta migratoria*	87
*CkiaOR65*	711	236	5′ lost	2	KP843318	Odorant receptor 99	*Locusta migratoria*	84
*CkiaOR66*	783	261	5′ lost	2	KY965017	Odorant receptor 100	*Locusta migratoria*	83
*CkiaOR67*	1377	459	Complete	4	KP843309	Odorant receptor 101	*Locusta migratoria*	86
*CkiaOR68*	1338	445	Complete	6	KP843271	Odorant receptor 102	*Locusta migratoria*	83
*CkiaOR69*	1341	446	Complete	6	KP843239	Odorant receptor 103	*Locusta migratoria*	80
*CkiaOR70*	1368	455	Complete	5	KP843270	Odorant receptor 105	*Locusta migratoria*	82
*CkiaOR71*	1350	449	Complete	4	KP843274	Odorant receptor 106	*Locusta migratoria*	82
*CkiaOR72*	1347	449	Complete	0	KP843267	Odorant receptor 107	*Locusta migratoria*	84
*CkiaOR73*	1311	437	Complete	0	KP843338	Odorant receptor 109	*Locusta migratoria*	83
*CkiaOR74*	1362	453	Complete	3	KP843357	Odorant receptor 110	*Locusta migratoria*	82
*CkiaOR75*	1257	419	3′ lost	4	KP843264	Odorant receptor 112	*Locusta migratoria*	89
*CkiaOR76*	1305	434	Complete	1	KP843301	Odorant receptor 113	*Locusta migratoria*	82
*CkiaOR77*	1272	424	Complete	3	KP843317	Odorant receptor 114	*Locusta migratoria*	81
*CkiaOR78*	1047	349	5′, 3′ lost	4	KP843330	Odorant receptor 116	*Locusta migratoria*	88
*CkiaOR79*	528	175	3′ lost	2	KY965035	Odorant receptor 118	*Locusta migratoria*	89
*CkiaOR80*	1326	442	Complete	5	KY965036	Odorant receptor 119	*Locusta migratoria*	85
*CkiaOR81*	1260	419	Complete	6	KP843236	Odorant receptor 120	*Locusta migratoria*	87
*CkiaOR82*	954	318	3′ lost	5	KP843260	Odorant receptor 123	*Locusta migratoria*	85
*CkiaOR83*	900	300	3′ lost	4	KP843349	Odorant receptor 124	*Locusta migratoria*	83
*CkiaOR84*	1296	431	Complete	5	KP843285	Odorant receptor 127	*Locusta migratoria*	87
*CkiaOR85*	1320	439	Complete	5	KP843259	Odorant receptor 130	*Locusta migratoria*	85
*CkiaOR86*	1389	463	Complete	6	KP843298	Odorant receptor 132	*Locusta migratoria*	86
*CkiaOR87*	1314	437	Complete	2	KP843241	Odorant receptor 133	*Locusta migratoria*	80
*CkiaOR88*	921	307	5′ lost	2	KP843290	Odorant receptor 135	*Locusta migratoria*	88
*CkiaOR89*	1290	430	Complete	7	KP843232	Odorant receptor 138	*Locusta migratoria*	86
*CkiaOR90*	1221	406	Complete	4	KP843287	Odorant receptor 140	*Locusta migratoria*	89
*CkiaORCO*	1458	486	Complete	7	KP843368	Odorant receptor co	*Locusta migratoria*	95

### Identification and Analysis of Putative IRs

**FIGURE 3:**
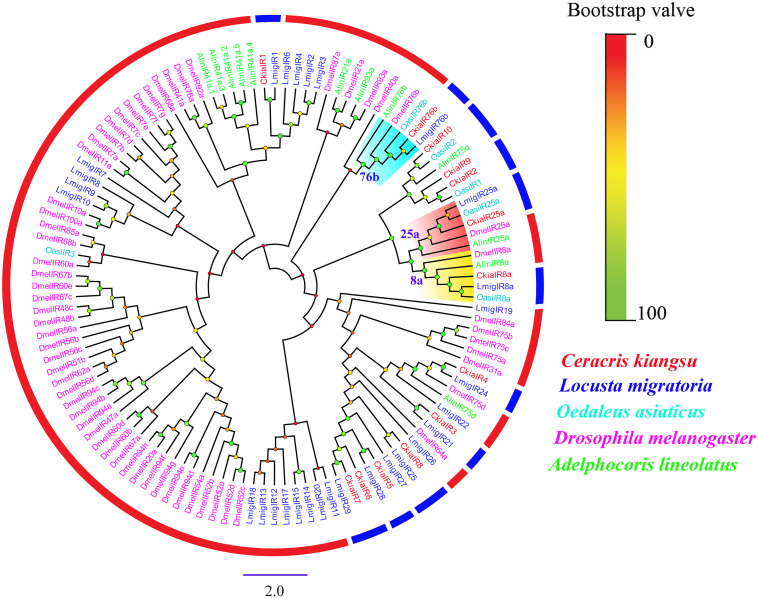
Phylogenetic tree of putative ionotropic receptors (IRs) from *C. kiangsu* and other insects. Branch support (circles at the branch nodes) was estimated using bootstrap values based on the scale indicated on the top right.

Thirteen putative IRs (*CkiaIR1-10*, *CkiaIR8a*, *CkiaIR25a*, and *CkiaIR76b*) were identified in the antennal transcriptome analysis of *C. kiangsu* according to their similarity analysis with known IRs. Among the IRs, eight IR sequences contained a complete ORF, and the remaining five sequences were incomplete due to a lack of a 5′ and/or 3′ terminus (Table 2). All of the IRs encoded longer ORFs (exceeding 2000 bp, except *CkiaIR7*) than ORs and had TMDs ranging from 1 to 4. An IR phylogenetic tree based on 120 protein sequences from five insects (*C. kiangsu*, *L. migratoria*, *O. asiaticus*, *D. melanogaster*, and *Adelphocoris lineolatus*) was then constructed, and the tree showed that all the *CkiaIRs* were clustered with other known orthopteran IRs into a separate clade (Figure 3). In the phylogenetic analysis, *CkiaIR8a*, *CkiaIR25a*, and *CkiaIR76b* were located in the clades of the IR8a group, IR25a group, and IR76b group, respectively, labeled with yellow, red, and cyan.

**TABLE 2:** Summary of putative ionotropic receptors (IRs) and sensory neuron membrane proteins (SNMPs) identified in *C. kiangsu*.

Gene name	Length (nt)	ORF (aa)	ORF status	Tm domain	Best blastx match			
					Gene ID	Gene name	Species	Similarity (%)
*CkiaIR1*	2010	669	Complete	1	KP843217	Ionotropic receptor 1	*Locusta migratoria*	91
*CkiaIR2*	2739	913	Complete	3	KP843203	Ionotropic glutamate receptor 12	*Locusta migratoria*	92
*CkiaIR3*	2316	771	Complete	3	KP843211	Ionotropic receptor 21	*Locusta migratoria*	92
*CkiaIR4*	2025	674	Complete	3	KP843214	Ionotropic receptor 24	*Locusta migratoria*	90
*CkiaIR5*	1935	644	Complete	3	KP843229	Ionotropic receptor 28	*Locusta migratoria*	91
*CkiaIR6*	1419	473	3′ lost	2	KP843229	Ionotropic receptor 28	*Locusta migratoria*	76
*CkiaIR7*	1971	657	Complete	1	KT279132	Ionotropic receptor 29	*Locusta migratoria*	92
*CkiaIR8*	902	300	3′ lost	2	KP843228	Ionotropic receptor 25	*Locusta migratoria*	92
*CkiaIR9*	867	288	5′ lost	2	KP843209	Ionotropic glutamate receptor 9	*Locusta migratoria*	92
*CkiaIR10*	639	213	5′, 3′ lost	1	KP843215	Ionotropic glutamate receptor 8	*Locusta migratoria*	95
*CkiaIR8a*	2691	896	Complete	3	KR349063	Ionotropic receptor 8a	*Locusta migratoria*	92
*CkiaIR25a*	2715	904	Complete	2	MH196264	Ionotropic receptor 25a	*Oedaleus asiaticus*	94
*CkiaIR76b*	1470	490	3′ lost	4	KP843210	Ionotropic receptor 76b	*Locusta migratoria*	92
*CkiaSNMP1*	1545	514	Complete	2	KU659599	Sensory neuron membrane protein 1	*Schistocerca gregaria*	89
*CkiaSNMP2*	869	289	3′ lost	1	MH196272	Sensory neuron membrane protein 2b	*Oedaleus asiaticus*	87

### Identification and Analysis of Putative OBPs

**FIGURE 4:**
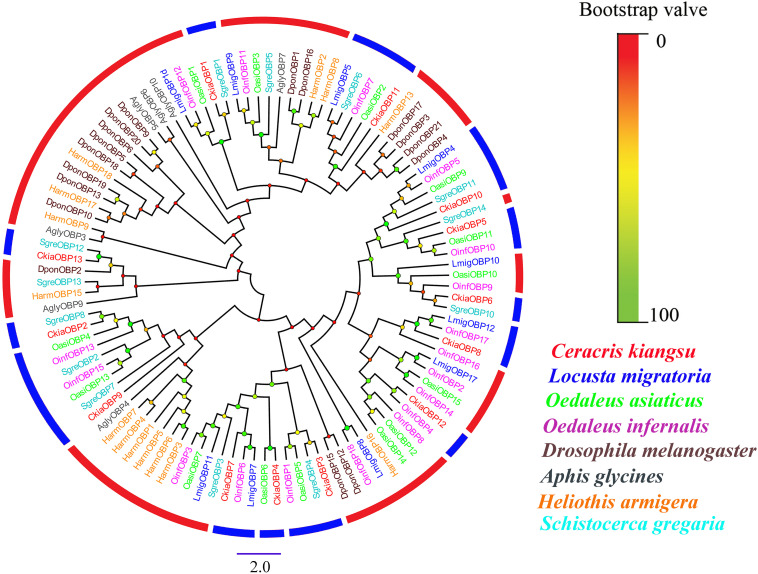
Phylogenetic tree of putative odorant-binding proteins (OBPs) from *C. kiangsu* and other insects. Branch support (circles at the branch nodes) was estimated using bootstrap values based on the scale indicated on the top right.

Antennal transcriptome analyses of *C. kiangsu* identified 13 putative OBPs (*CkiaOBP1-13*), of which five were newly identified. Bioinformatic analyses revealed that all identified *CkiaOBPs* except *OBP5* had a complete ORF, with lengths ranging from 136 to 272 amino acids (Table 3). All predicted proteins with complete ORFs had six highly conserved cysteine residues and a predicted signal peptide at the N-terminal region (Supplementary Figure S1 and Table S6). The conserved domain prediction of *CkiaOBPs* showed 11 had an insect pheromone/odorant-binding protein domain, and the other two (*CkiaOBP5* and *CkiaOBP7*) had a PBP/GOBP family domain, all of which belonged to the InterPro family (InterPro: IPR006170). To reveal the homologous relationships of all putative OBPs of *C. kiangsu* with other insect gene sets, a phylogenetic tree was constructed using the protein sequences of 106 OBPs from nine species (*C. kiangsu*, *L. migratoria*, *O. asiaticus*, *Oedaleus infernalis*, *Schistocerca gregaria*, *D. melanogaster*, *Aphis glycines*, and *Heliothis armigera*) (Figure 4). The phylogenetic analysis demonstrated that all 13 *CkiaOBPs* were distributed along various branches, and each was clustered with at least one other locust ortholog.

**TABLE 3:** Summary of putative odorant binding proteins (OBPs) identified in *C. kiangsu*.

Gene name	Length (nt)	ORF (aa)	ORF status	Signal peptide	Best blastx match			
					Gene ID	Gene name	Species	Similarity (%)
*CkiaOBP1*	459	153	Complete	1–21	KP255951	Odorant-binding protein 1	*Ceracris kiangsu*	100
*CkiaOBP2*	441	147	Complete	1–21	KP255952	Odorant-binding protein 2	*Ceracris kiangsu*	100
*CkiaOBP3*	465	155	Complete	1–20	KP255953	Odorant-binding protein 3	*Ceracris kiangsu*	100
*CkiaOBP4*	465	155	Complete	1–18	KP255954	Odorant-binding protein 4	*Ceracris kiangsu*	100
*CkiaOBP5*	307	103	5′, 3′ lost	No	KP255955	Odorant-binding protein 5	*Ceracris kiangsu*	100
*CkiaOBP6*	465	155	Complete	1–20	KP255956	Odorant-binding protein 6	*Ceracris kiangsu*	100
*CkiaOBP7*	491	164	Complete	1–19	KP255957	Odorant-binding protein 7	*Ceracris kiangsu*	100
*CkiaOBP8*	468	155	Complete	1–18	KP255958	Odorant-binding protein 8	*Ceracris kiangsu*	100
*CkiaOBP9*	513	171	Complete	1–19	KP293574	Odorant-binding protein 8	*Oedaleus decorus asiaticus*	89
*CkiaOBP10*	450	150	Complete	1–25	MF716568	Odorant-binding protein 11	*Schistocerca gregaria*	90
*CkiaOBP11*	435	145	Complete	1–29	MG507284	Odorant-binding protein 7	*Oedaleus infernalis*	95
*CkiaOBP12*	408	136	Complete	1–23	MG507281	Odorant-binding protein 4	*Oedaleus infernalis*	90
*CkiaOBP13*	816	272	Complete	1–22	MF716569	Odorant-binding protein 12	*Schistocerca gregaria*	92

### Identification and Analysis of Putative CSPs

**FIGURE 5:**
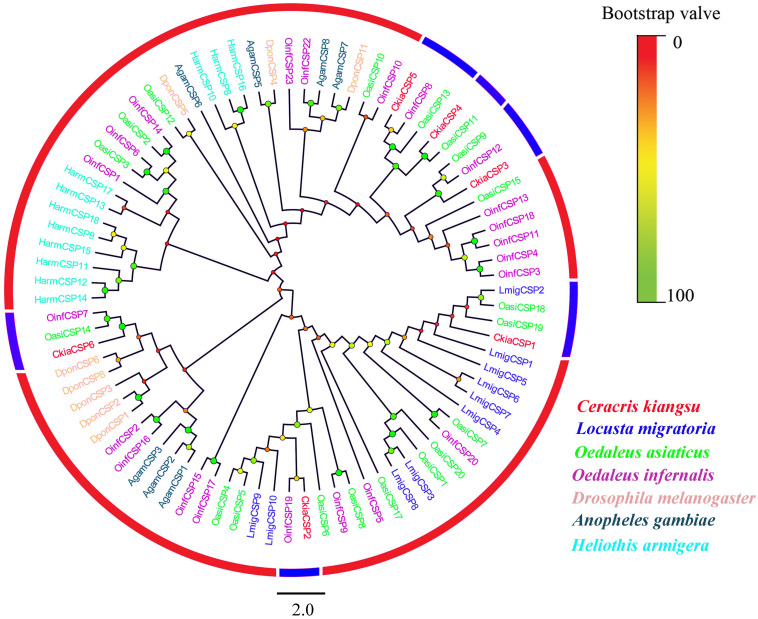
Phylogenetic tree of putative chemosensory-binding proteins (CSPs) from *C. kiangsu* and other insects. Branch support (circles at the branch nodes) was estimated using bootstrap values based on the scale indicated on the top right.

Six different unigenes encoding putative CSPs (*CkiaCSP1-6*) were identified by analyzing the transcriptome data of *C. kiangsu*. Sequence analysis revealed that all putative *CkiaCSPs* had full-length ORFs, with sizes ranging from 118 to 146 amino acids (Table 4). All candidate CSP genes had four conserved cysteines in the corresponding position and a conserved OS-D domain (InterPro: IPR005055) (Supplementary Figure S2 and Table S7). The signal peptide prediction of the SignalP test showed that all six *CkiaCSPs* had a predicted signal peptide at the N-terminal region. The constructed insect CSP tree using amino acid sequences of the 83 CSPs from seven species (*C. kiangsu*, *L. migratoria*, *O. asiaticus*, *O. infernalis*, *D. melanogaster*, *H. armigera*, and *Anopheles gambiae*) indicated that all six *CkiaCSPs* were clustered with at least one orthopteran ortholog, which accorded with the results of the sequence similarity analyses (Figure 5).

**TABLE 4:** Summary of putative chemosensory proteins (CSPs) identified in *C. kiangsu*.

Gene name	Length (nt)	ORF (aa)	ORF status	Signal peptide	Best blastx match			
					Gene ID	Gene name	Species	Similarity (%)
*CkiaCSP1*	426	141	Complete	1–35	KX905075	Chemosensory protein 19	*Oedaleus asiaticus*	89
*CkiaCSP2*	387	128	Complete	1–19	KX905060	Chemosensory protein 4	*Oedaleus asiaticus*	90
*CkiaCSP3*	396	128	Complete	1–20	KX905065	Chemosensory protein 9	*Oedaleus asiaticus*	82
*CkiaCSP4*	354	118	Complete	No	KX905067	Chemosensory protein 11	*Oedaleus asiaticus*	91
*CkiaCSP5*	396	132	Complete	1–27	KX905069	Chemosensory protein 13	*Oedaleus asiaticus*	89
*CkiaCSP6*	441	146	Complete	1–26	KX905070	Chemosensory protein 14	*Oedaleus asiaticus*	86

### Identification and Analysis of Putative SNMPs

**FIGURE 6:**
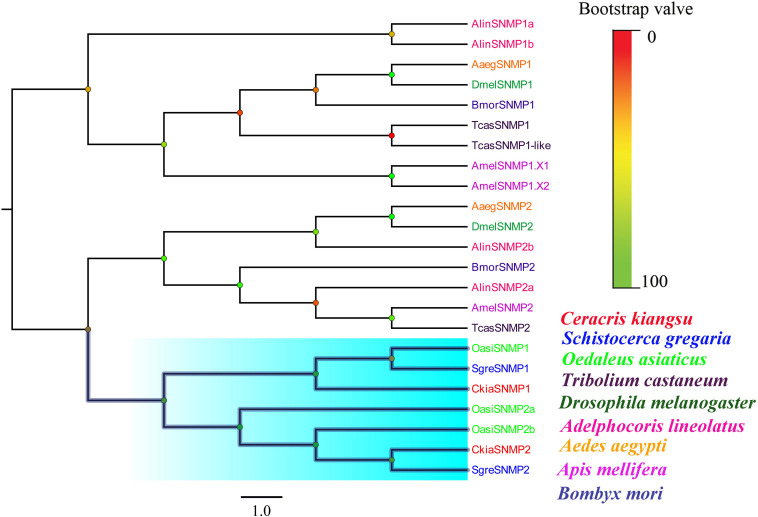
Phylogenetic tree of putative sensory neuron membrane proteins (SNMPs) from *C. kiangsu* and other insects. Branch support was estimated using bootstrap values based on the scale indicated on the top right.

Two putative SNMPs, termed *CkiaSNMP1* and *CkiaSNMP2*, were identified in our transcripts based on the BLASTx and cluster analysis results. *CkiaSNMP1* has full-length ORFs encoding 514 amino acids, and *CkiaSNMP2* is incomplete because it lacks a 3′ terminus (Table 2). In addition, the candidate SNMP1 contains two TMDs, while SNMP2 contains only one Tm domain. Phylogenetic analysis was performed based on amino acid sequences of 23 SNMPs from nine species (*C. kiangsu*, *O. asiaticus*, *S. gregaria, D. melanogaster*, *A. lineolatus*, *Tribolium castaneum*, *Aedes aegypti, Apis mellifera*, and *Bombyx mori*). In the phylogenetic tree, the SNMPs of orthopteran species were clustered together and were classified into two distinct subgroups, i.e., SNMP1 and SNMP2 (Figure 6). As expected, the two putative SNMPs of *C. kiangsu* were grouped into two subclades.

### Expression Analyses of Putative Chemosensory Genes by qRT-PCR

**FIGURE 7:**
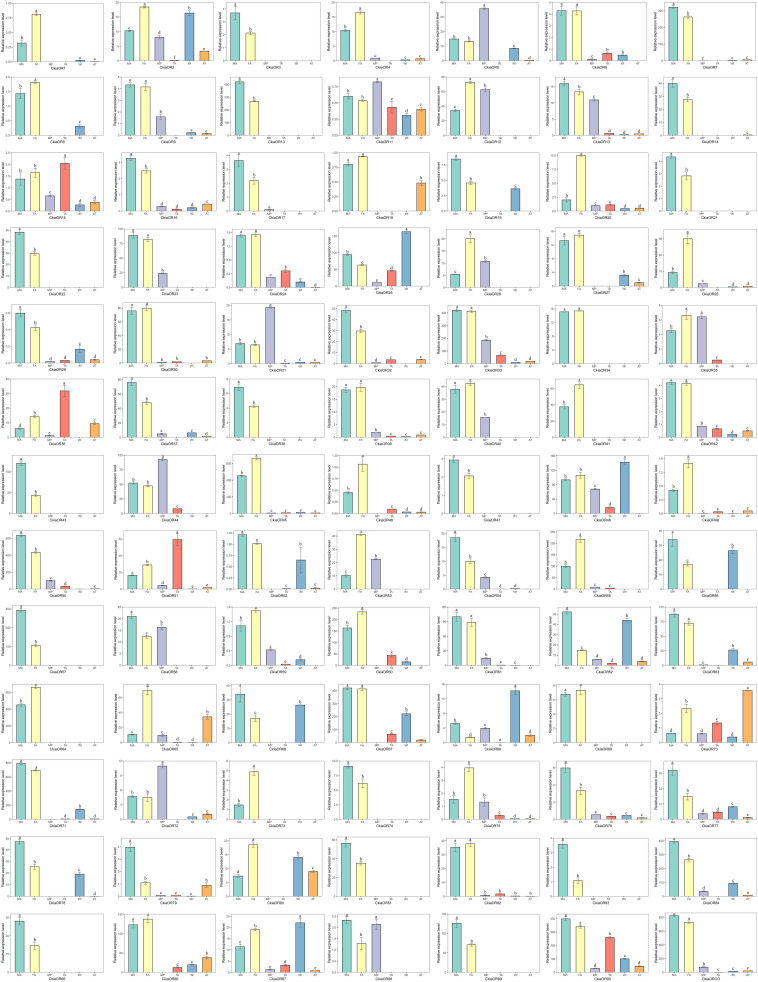
Relative expression levels of ORs in different tissues of *C. kiangsu* as measured by RT-qPCR. FA, female antennae; MA, male antennae; MP, maxillary palps; TA, tarsi; WI, wings; AT, thoraxes-abdomens. The bar represents standard error and the different small letters above each bar indicate significant differences (*P* < 0.05).

The expression profiles of all candidate *CkiaORs* were analyzed, and these results indicated that 78 out of 91 genes were expressed at the highest levels in the antennae rather than in other tissues, and 18 genes showed an antenna-specific expression pattern (Figure 7). Of these, 35 *CkiaORs* exhibited significantly male-biased expression patterns, 22 exhibited strongly female-biased, while the remaining 21 *CkiaORs* showed similar expression levels in the antennae of both sexes. Five candidate ORs (*CkiaOR5*/*11/31/44/72*) displayed the highest expression abundance in the maxillary palps. Besides the two olfactory tissues, relatively high expressions of *CkiaORs* were also detected in other non-olfactory tissues. For example, four genes (*CkiaOR25/48/68/87*) were expressed significantly more in wings, *CkiaOR15*, *CkiaOR36*, and *CkiaOR51* exhibited the highest expression levels in the tarsi, and *CkiaOR70* was more highly expressed in thoraxes-abdomens than in other tissues. In addition, 21 *CkiaORs* were expressed at different levels in all olfactory and non-olfactory tissues.

**FIGURE 8:**
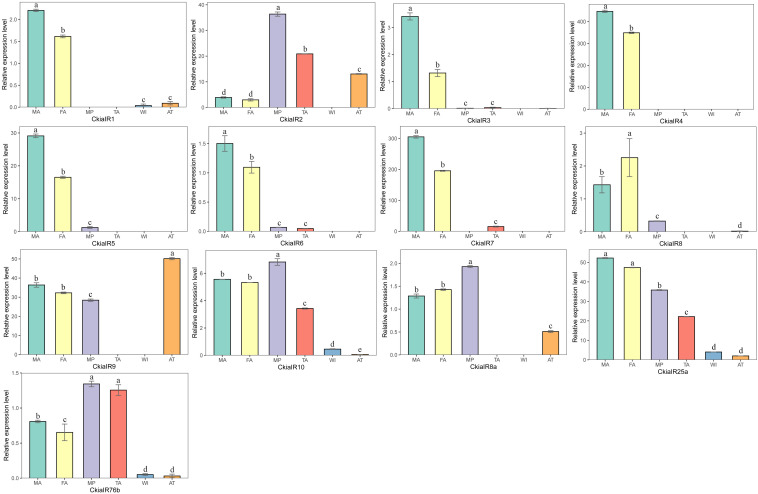
Relative expression levels of IRs in different tissues of *C. kiangsu* as measured by RT-qPCR. FA, female antennae; MA, male antennae; MP, maxillary palps; TA, tarsi; WI, wings; AT, thoraxes-abdomens. The bar represents standard error and the different small letters above each bar indicate significant differences (*P* < 0.05).

The expression levels of 13 *CkiaIRs* in different tissues were detected using qRT-PCR (Figure 8). The analyses revealed that eight genes (*CkiaIR1/3/4/5/6/7/8*) were antennae-enriched. Of these, *CkiaIR4* was expressed specifically in the antennae, and seven IRs (except *CkiaIR8*) displayed the same expression profiles that had higher expression in male antennae than in female antennae. Four genes (*CkiaIR2*, *CkiaIR10*, *CkiaIR8a*, and *CkiaIR76b*) were most highly expressed in the other olfactory organ (the maxillary palps), and *CkiaIR9* was most highly expressed in a non-olfactory organ (the thoraxes-abdomens). Furthermore, *CkiaIR10*, *CkiaIR25a*, and *CkiaIR76b* had different expression levels in all six tissues.

**FIGURE 9:**
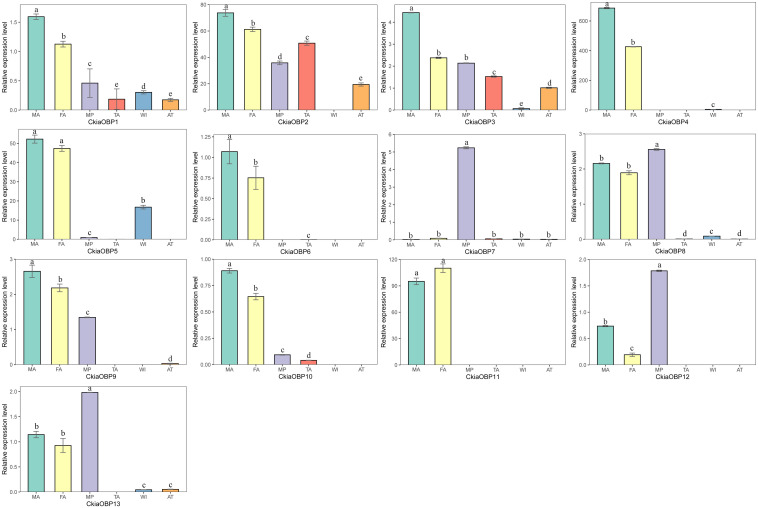
Relative expression levels of OBPs in different tissues of *C. kiangsu* as measured by RT-qPCR. FA, female antennae; MA, male antennae; MP, maxillary palps; TA, tarsi; WI, wings; AT, thoraxes-abdomens. The bar represents standard error and the different small letters above each bar indicate significant differences (*P* < 0.05).

The results of the expression profile analyses of the OBPs showed that all of the candidate genes were expressed in the antennae of *C. kiangsu*, except for *CkiaOBP7*, which was almost exclusively expressed in the maxillary palps (Figure 9). Of the OBPs expressed in the antennae, *CkiaOBP6* and *CkiaOBP11* were specifically expressed in the antennae, and ten genes were more highly expressed in male insects than in female insects. Three putative OBPs (*CkiaOBP8*, *CkiaOBP12*, and *CkiaOBP13*) had higher expression levels in the maxillary palps than in the antennae. In addition, several OBPs were expressed not only in the olfactory tissues but also in the non-olfactory tissues; for example, *CkiaOBP1* and *CkiaOBP3* were widely expressed in all tissues tested.

**FIGURE 10:**
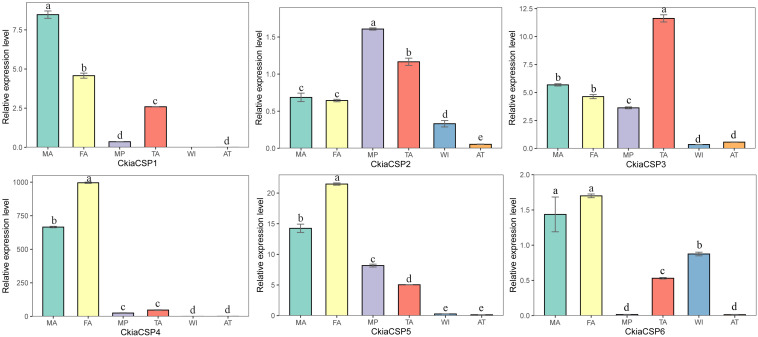
Relative expression levels of CSPs in different tissues of *C. kiangsu* as measured by RT-qPCR. FA, female antennae; MA, male antennae; MP, maxillary palps; TA, tarsi; WI, wings; AT, thoraxes-abdomens. The bar represents standard error and the different small letters above each bar indicate significant differences (*P* < 0.05).

The quantitative expression levels of CSPs showed that all six genes had a ubiquitous expression in the antennae, of which *CkiaCSP3* had similar expression patterns in females and males, and three genes (*CkiaCSP4*, *CkiaCSP5*, and *CkiaCSP6*) exhibited higher expression levels in females (Figure 10). Moreover, *CkiaCSP2* and *CkiaCSP3* were most abundantly expressed in the maxillary palps and tarsi, respectively.

**FIGURE 11:**
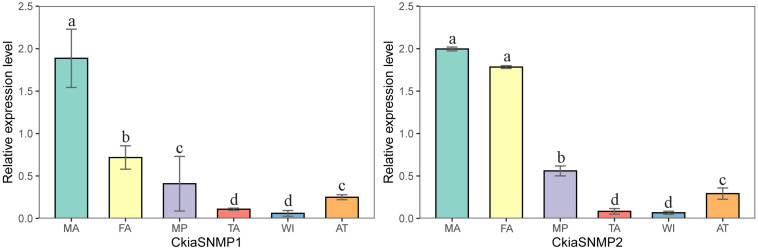
Relative expression levels of SNMPs in different tissues of *C. kiangsu* as measured by RT-qPCR. FA, female antennae; MA, male antennae; MP, maxillary palps; TA, tarsi; WI, wings; AT, thoraxes-abdomens. The bar represents standard error and the different small letters above each bar indicate significant differences (*P* < 0.05).

Of the candidate SNMPs, *CkiaSNMP1* and *CkiaSNMP2* were widely expressed in all of the olfactory and non-olfactory organs tested (Figure 11). Two genes displayed the highest expression levels in the antennae, and, in relative terms, SNMP1 was expressed significantly more in males than in females, while SNMP2 was similarly expressed in both sexes.

## Discussion

The bamboo locust, *C*. *kiangsu*, is one of the most invasive and destructive pests in southern China, resulting in large economic losses of gramineous plants. In order to better understand the molecular mechanism of olfactory perception, the first step is to investigate chemosensory genes, which encode the proteins that function in odorant molecular detection. In the present study, we sequenced and analyzed the antennal transcriptomes of female and male *C. kiangsu* for the first time. We also identified 125 candidate chemosensory genes from the transcriptomes, including five multigene families of ORs (91), IRs (13), OBPs (13), CSPs (6), and SNMPs (2). Of the candidate chemosensory genes, eight OBPs (*CkiaOBP1–CkiaOBP8*) were also characterized based on the head transcriptome of *C. kiangsu* in our previous study (Li et al., 2018). The total number of chemosensory genes identified in *C. kiangsu* was less than that identified in *L.migratoria* (195) (Wang et al., 2014, 2015). One reason for this difference is that our dataset was derived from transcriptomes, whereas that of *L.migratoria* was derived from the genome, which contains much more information than a transcriptome. Another reason may be that the genes were identified from only one tissue (antennae), and genes that were specifically expressed in other important chemosensory organs (e.g., maxillary palps) were not found in our analyses. In addition, GRs, the other important multigene family of chemosensory genes, were not found in our analyses. One possible reason for this is that GRs have no expression, or weak expression in the antennae of *C. kiangsu* but may be expressed at high levels in other tissues, such as the mouthparts, wings, genitalia, and the tarsal segments of legs.

ORs, which are expressed in ORNs, play an important role in insect olfaction (Zhang and Löfstedt, 2015). In this work, a total of 91 *CkiaORs* were identified, including one *CkiaORCO*, which is more conserved across insect orders (Hallem et al., 2006; Sato and Touhara, 2008). The number of identified *CkiaORs* was less than that in *L.migratoria* (Wang et al., 2015) and *S.gregaria* (Pregitzer et al., 2017), and more than that in *O.asiaticus* (60) (Zhou et al., 2019). The significant differences in gene numbers might be due to a range of factors, including sample preparation, sequencing methods, and sequencing depth. Despite this, there is an expanded OR family in orthopteran species compared with lepidopteran and dipteran species (Vieira and Rozas, 2011). The expression profile analyses of *CkiaORs* showed that most (78 of 91) genes were expressed at the highest levels in the antennae, of which 18 genes (*CkiaOR 3/10/21/22/34/38/41/43/47/57/64/69/73/74/81/83/85/89*) showed antennal-specific expression (Figure 7). Our results were consistent with those of previous studies, which have revealed that most OR expressions in insects are localized in the antennae (Vosshall et al., 1999; Wang et al., 2015; Zhou et al., 2019). For the antenna-predominant genes, 35 *CkiaORs* were male-biased expressed, indicating their potential function in mating behavior and female sex pheromone recognition; 22 were significantly female-biased, suggesting that they may be involved in the detection of oviposition sites; and the remaining 21 showed no significant differences between the two sexes. The five ORs (*CkiaOR5/11/31/44/72*), which were most highly expressed in the maxillary palps, might play an important role in oviposition site selection (for females), mating selection (for males), and host selection (for both sexes). In addition, there were 21 *CkiaORs* expressed at different levels in all of the tested tissues, and four genes (*CkiaOR5/48/68/87*), three genes (*CkiaOR15*, *CkiaOR36*, and *CkiaOR51*), and one gene (*CkiaOR70*) exhibited higher expression levels in wings, tarsi, and thoraxes-abdomens, respectively (Figure 7). Our results revealed that the genes could also play other general roles in the non-olfactory organs of *C. kiangsu*, which supports the conclusion of a previous study on *L.migratoria* that 11 *LmigORs* are highly expressed in the non-olfactory tissues (wings and legs) (Wang et al., 2015).

IRs, the other multigene family of chemosensory genes, are also ligand-gated ion channels and are assigned a tentative role in both olfaction and gustation. A total of 13 candidate *CkiaIRs* were identified, including three co-receptor genes (*CkiaIR8a*, *CkiaIR25a*, and *CkiaIR76b*) (Benton et al., 2009; Chen et al., 2015). Compared to *CkiaORs*, the *CkiaIRs* are more conserved among different species, and the similarities with the reference sequences of *L*. *migratoria* are between 90 and 95% (except *CkiaIR6*). Similar with *CkiaORCO*, the IR phylogenetic tree showed that three co-receptor genes (*IR8a*, *IR25a*, and *IR76b*) were clustered with other orthologs (Figure 8). In the expression levels of 13 candidate *CkiaIRs*, seven genes (*CkiaIR1/3/4/5/6/7/8*) exhibited significantly high expression in the antennae, which is similar to the expression pattern of *CkiaORs*. The antennae-enriched IRs of *D.melanogaster* displayed high expression in OSNs associated with the detection of sex pheromones, odors, and amines (Hussain et al., 2016; Ni et al., 2016; Tauber et al., 2017). Therefore, most of the identified *CkiaIRs* had potential functions in odorant reception. In addition, three genes (*CkiaIR10*, *CkiaIR25a*, and *CkiaIR76b*) had different expression levels in all test tissues, which was consistent with the result of previous studies that IRs were expressed not only in olfactory organs but in many non-olfactory organs and were involved in multiple functions (Koh et al., 2014; Ni et al., 2016; Ganguly et al., 2017).

Numerous previous studies have suggested that the OBPs and CSPs of insects have important functions in mating, oviposition, and host location selection (Zhang et al., 2013; Paula et al., 2016). Two soluble proteins are the first step of odor perception, which transfer odorant molecules to chemoreceptors. In this study, 13 putative OBPs and six putative CSPs were identified by analyzing the transcriptome data of *C. kiangsu*. The number of *CkiaOBPs* was similar to the number in *S.gregaria* (14 *SgreOBPs*) (Jiang et al., 2017), *O.asiaticus* (15 *OasiOBPs*) (Zhang et al., 2015), and less than the number in *O.infernalis* (18 *OinfOBPs*) (Zhang et al., 2018) and *L.migratoria* (22 *LmigOBPs*) (Wang et al., 2015). Eight of our 13 *CkiaOBPs* had 100% identity with the genes identified in the head transcriptome of *C. kiangsu*, which are reported in our present study. The same genes identified in different tissues indicated that these OBPs were expressed abundantly in the antennae (Figure 9). Our analyses using qRT-PCR showed that all of the *CkiaOBPs* (except for *CkiaOBP7*) were expressed in the antennae of *C. kiangsu*. Similar to other insects, *CkiaOBPs* are highly expressed in the antennae, which play roles in the recognition of host volatile compounds and sex pheromones (Gong et al., 2014; Brito et al., 2016; Guo et al., 2018). Among the 12 antennae-enriched *CkiaOBPs*, ten genes were expressed at higher levels in males. Hence, our results revealed that these genes might be playing a role in sex pheromone detection in *C. kiangsu*. Other than that, several *CkiaOBPs* displayed higher expression levels in other tissues. For example, *CkiaOBP1* and *CkiaOBP3* were widely expressed in all tested tissues, including non-olfactory tissues. *CkiaOBP7* was almost exclusively expressed in the maxillary palps. These *CkiaOBPs* were expressed in non-antennae tissues, implying that the functions of these genes may be involved in binding host plant volatile compounds, taste functions, pheromone release, and detection of egg-laying substrates (Sparks et al., 2014; Sun et al., 2017). All of the identified candidate CSP transcripts had a ubiquitous expression in the antennae of *C. kiangsu*, which demonstrated that CSPs might work on the chemosensory process (Figure 10). Moreover, we also found that half of the *CkiaCSPs* were most abundantly expressed in the maxillary palps and the tarsi (non-olfactory tissue), suggesting that the possible functions may differ from that of the olfactory process. As found in many previous studies, some insect CSPs were expressed in numerous non-olfactory organs with multiple other physiological functions (Yasukawa et al., 2010; Sparks et al., 2014; Sun et al., 2017).

Two candidate SNMP genes were identified based on their similarities with other IRs in orthopterans. The phylogenetic tree showed that the SNMPs of orthopteran species were clustered together, suggesting that the genes within the Orthoptera are more conservative. *CkiaSNMP1* and *CkiaSNMP2* exhibited the highest expression levels in the antennae of *C. kiangsu*, as found in previous studies of several insects (Figure 11; Vogt et al., 2009; Gomez-Diaz et al., 2016; Jiang et al., 2016). Notably, *CkiaSNMPs* were expressed significantly more in males than in females, and we speculate that these genes might have other functions (sex pheromone detection) and require further functional verification.

## Conclusion

In this study, a total of 125 chemosensory genes belonging to five multigene families were identified from the antennal transcriptomes of *C. kiangsu*, including 91 ORs, 13 IRs, 13 OBPs, 6 CSPs, and 2 SNMPs. These genes were classified based on sequence conservation, transmembrane domain prediction, and phylogenetic analyses. Expression patterns were validated using qRT-PCR and showed that most candidate chemosensory genes were highly expressed in the antennae, some were abundant in the maxillary palps, and some were expressed in the non-olfactory tissues. In addition, among the antennal-predominant genes, different expression levels were displayed. Our data provide valuable molecular information for future investigations of the chemoreception mechanisms of *C. kiangsu*. More importantly, our studies provide an important genetic framework for the development of environmentally friendly pesticides against this bamboo pest in the future.

## Data Availability Statement

The datasets presented in this study can be found in online repositories. The names of the repository/repositories and accession number(s) can be found in the article/Supplementary Material.

## Author Contributions

RL and G-FJ conceived and designed the experiments and drafted and revised the manuscript. RL and Y-QW carried out the experiments. RL and X-HS analyzed the data. G-FJ, RL, and M-JL contributed reagents, materials, and analysis tools. All authors approved the final version of the manuscript.

## Conflict of Interest

The authors declare that the research was conducted in the absence of any commercial or financial relationships that could be construed as a potential conflict of interest.

## Footnotes

1 http://www.clustal.org/clustal2

2 https://www.ncbi.nlm.nih.gov/orffinder

3 http://www.cbs.dtu.dk/services/TMHMM

4 http://www.cbs.dtu.dk/services/SignalP

5 https://www.ebi.ac.uk/Tools/msa/mafft

6 https://www.megasoftware.net

7 https://www.evolgenius.info/evolview

8 http://tree.bio.ed.ac.uk/software/figtree

9 http://www.premierbiosoft.com

## Supplementary Material

FIGURE S1 Alignments of the *C. kiangsu* odorant-binding proteins (OBPs). Six conserved cysteines are highlighted in red.

FIGURE S2 Alignments of the *C. kiangsu* chemosensory-binding proteins (CSPs). Four conserved cysteine are highlighted in red.

TABLE S1 Amino acid sequences of chemosensory genes of *Ceracris kiangsu* and other insects used in phylogenetic analysis.

TABLE S2 Primers used for qRT-PCR.

TABLE S3 The summary of the Illumina sequencing data.

TABLE S4 Length distribution and quality metrics of the *C. kiangsu* transcripts and unigenes.

TABLE S5 The summary of functional annotation of *C. kiangsu* transcriptomes.

TABLE S6 Conserved domains of odorant binding proteins in *C. kiangsu*.

TABLE S7 Conserved domains of chemosensory proteins in *C. kiangsu*.
