# Supplementary figures and images for: Identification and Expression Profile Analysis of Chemosensory Genes From the Antennal Transcriptome of Bamboo Locust (Ceracris kiangsu)
Source: Front Physiol. 2020 Sep 9;11:889. doi: 10.3389/fphys.2020.00889 (PMC7509195; doi:10.3389/fphys.2020.00889)

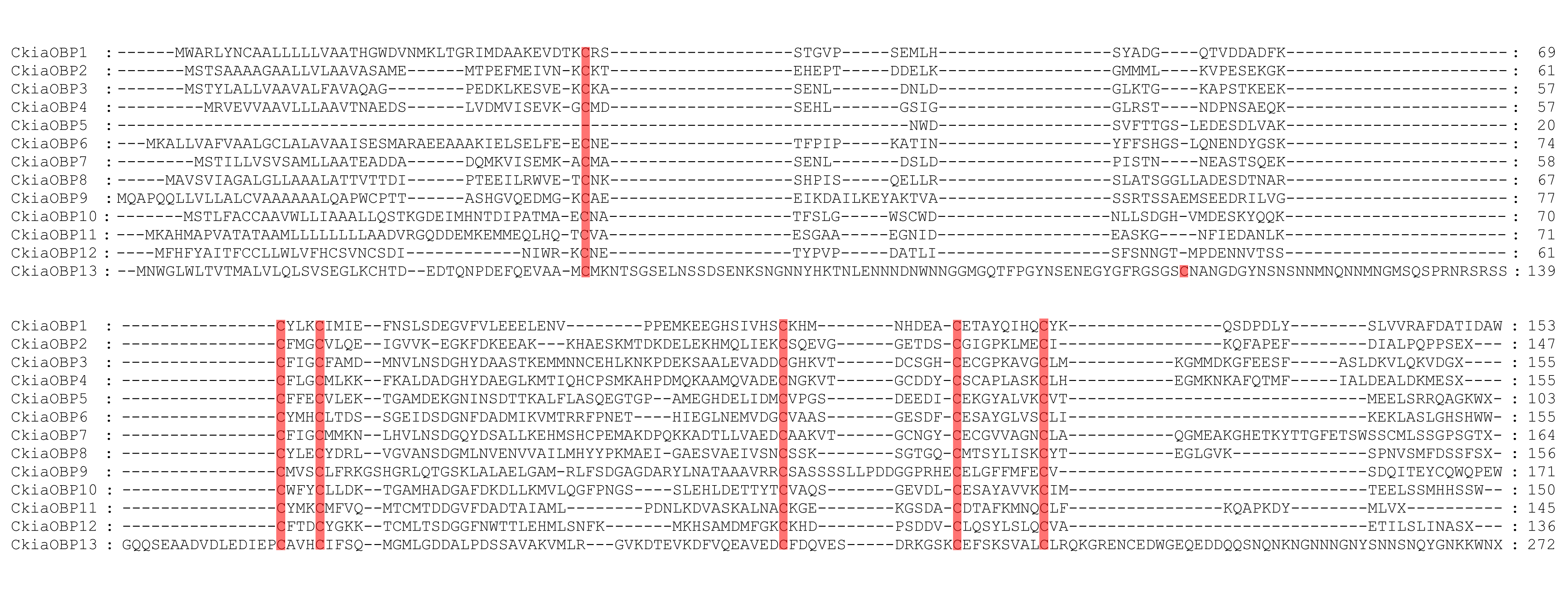

Supplement: FIGURE S1 — Alignments of the C. kiangsu odorant-binding proteins (OBPs). Six conserved cysteines are highlighted in red. [file Image_1.JPEG]

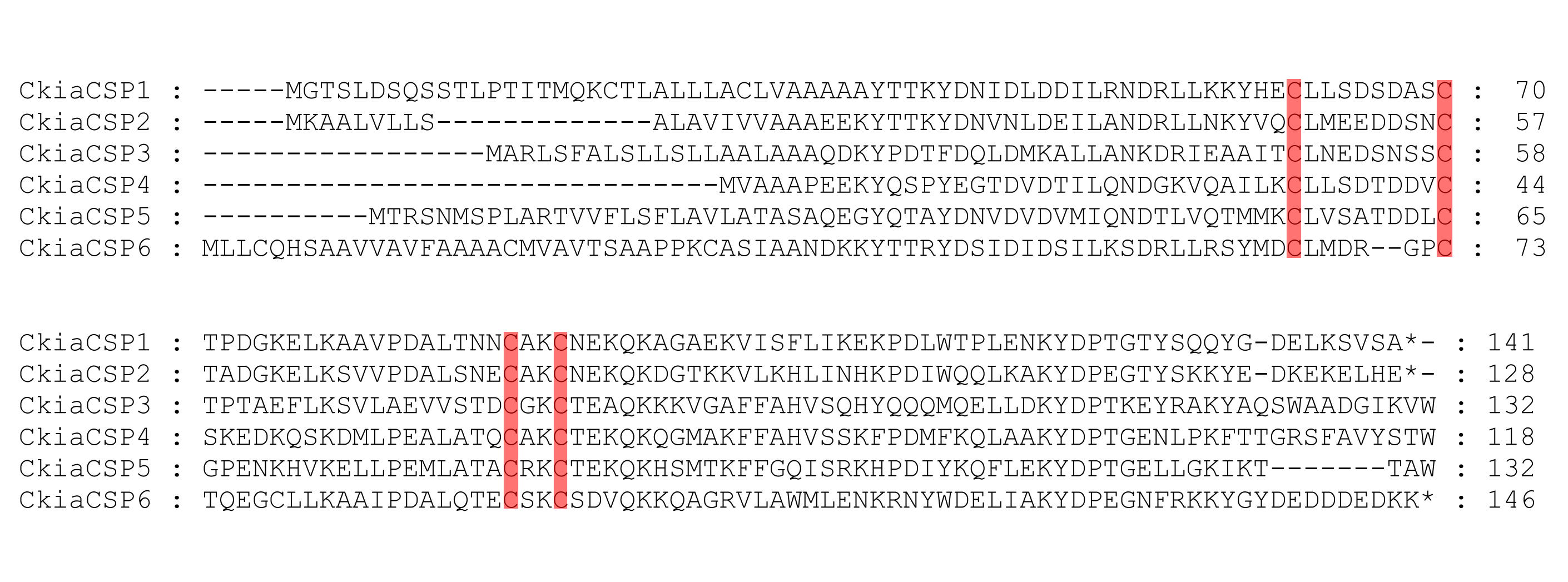

Supplement: FIGURE S2 — Alignments of the C. kiangsu chemosensory-binding proteins (CSPs). Four conserved cysteine are highlighted in red. [file Image_2.JPEG]
