# Supplementary material for: Identification and Expression Profile Analysis of Chemosensory Genes From the Antennal Transcriptome of Bamboo Locust (Ceracris kiangsu)
Source: Front Physiol. 2020 Sep 9;11:889. doi: 10.3389/fphys.2020.00889 (PMC7509195; doi:10.3389/fphys.2020.00889)
Supplement: TABLE S1 — Amino acid sequences of chemosensory genes of Ceracris kiangsu and other insects used in phylogenetic analysis. [file Table_1.docx]

**Table S1** Amino acid sequences of chemosensory genes of *Ceracris kiangsu* and other insects used in phylogenetic analysis.

**File S1.** Amino acid sequences of 218 ORs of *Ceracris kiangsu* and other insects used in phylogenetic analysis.

>CkiaOR1

MSNSNESMYFGTIINLMHFFKMWSPDDSKSNMSFSVYILIPAYLFFFALSCIEIYHNWGDLLSTTDAVNTFVIFFVTGHKCFSFIYHEKDFKKLIKIIDNNFSVPTWQNDESHNSMIKNYVQQVKKLNMIWSITAFTKHSGFMILPLADGLFHYHTANKTAEVEWKIPFSTWTPFNDYGTIVTLPLYMYHMFMGFVLVYFMPAFDTLFCTLMTHACAQLKILQNSLVNMVAISTQNVHSDENNSFTFDTMLNEHCTFEENKDRSMPQAPKMNSALNYYTPNGNYDSTLNPMHDSELDNKMRKNVGQLVNHHESMLEFIDGVEAIVNAVFLTHILCSVTAFSATGFQLTVVLEEQQLVSYLNMMGLLGGAMFEMGMFCYYSNRVMDEFINVGKAAYDSQWYYVSKDYGISVSMIMARCTRPPSMTFGKFAELTMEKFGSILHVSYSYFTLLTGLNEW

>CkiaOR2

MLRQVYDALRNANEPSRYLQFNVLMVRFMGVLVGRGVAGALYTWSLLALLLTQGLAGVLDLVDSSDIADITANLPVTTIIFSSAYRLFFFTLHRDRYQAIVDTVGERFVASSTSVDMAAWLRRSSMISMVYFTYGSFVAGTWQLHPLISAQLTSAAMAKESNGTFEGLPSELWEFPFSAEYPFDASQPYVYTVIFILQGATTFVAGCMILVLDMMFITLTSLMSGQLEMVKDKLRNMSKMATGESKNEEIGLVGKALQKQALERRSSILNSNIDATKDENFNEDIVTSKMNLLLGECVEHHNMLLSLISEIEFMHWSAFMVNFCVLLIMFSFAAFEVTSGTPTSPAKVVNLAEYLLISMLQMFLLCDCGDKLVDQELSVSQAAYESQWYHCSESVKCTLQIIVLRASQPEQITVGKMAGLNLDTFSDMLSRSFSYFTVLSQMRDG

>CkiaOR3

MLANGMSGLDLTDYLQPILSPLAATGMWSSSIYSNNVHKTVASTIGLSLLGLFLVELSAEVVGTPLALASNGDVDRLIHDLSVLGIHLDSFWKWLFMLTQRRRLTQLLQLLQRCFCLGVLASPPDRLALQNGYIRQQQSLAVPALLREELGSTRLRCLLVTVLWTGSCVLGACHWYFVPRLKGDSSLRFDAWYPFHSQQPPIKQLVYWLQYSCSISSLLMLCFFDCLLVWLQQLLCVQLRSLASNLSGLSRNDASLNCDQRSILAVCVSHHEHILSAMRELNAFVAPLLFLQCFKNMMVLCVVAFLASVAGVNDLLELSSLLLYFMAACQQLFFYCWCCEELTHLGLELCDAAYDSGWENWDVSSQKSIVIMMWRAQKPFFFSGGWFYTLNVATFVDLISLSFSYYTVLRNMSEG

>CkiaOR4

MLAQGQACSGTGAGLPGTRVSSVDLRAHLSPILKCLAVFGMWPTSAYTRVSAKAAALTTGLVTLSLFLMQLMAEVMALAASPVNGPAELYRFIYNFSVVDLHLQGLGKWAMMVVRRRRYTGLVDRLQHCIHLSGLADADCSASLLPTSQKSQKAAVTSVHQQLGDCRRWGVRANAFWLSACVYGVTHWCLVPLLIGDNSLPFDALYLFDTDQPPLRQVAHCIQYVAGLQNVLLSVFFDLFVFWLHLLLCAQLRYVAGNLRRLRRLTDTGEYKSLLVACVAHHSHLLSTMQELNSCAGPSFFLQCFENTISMCMIAFMATTTVADQMQVWSSAQFFLAAVGQLFLYCWCGQQLSHLAEGMSEAVYDSGWEEHEVSTQKNVAFIMWRAQKTLIFKGGWFYTLTTETFVELIRLSFSYYTVLRNINDSW

>CkiaOR5

MDWNPREEAPLTWRQTASSVISFDVRILYVMGVWAMPVTKLYRTYTGVILVLAVAYSVEAVMHIWSVRKNMEEVTLALSTYAVIVTSCFKLLSFLQHEPGYWRLVRRLDALVADQKRFCEERPPLQTVFDEAKKRATSYPNALRIYNTSLILAWVFSPLLAPHGQSPLPFHQLPLSETDDFPLYLASYLMQSLCMFSLCLVSGCLDSFFTATMIHTAAQFKILSLRIASLRQDNGESKQDTSSDVHEKVRKDAATEHVYEELRLCIRTHQEITSFVAHLESVMNPIAALQLITGVTNGCLMMFPTAASSESGALLKCIGCMPTICSQVLIYCLGAHAVMEQSEAVSEAAYGSAWPDTSPRCRRALLVVMARAMKPLTLTAGGIYTMESATFLSLLNAGYSYYALLKNFNSREW

>CkiaOR6

MLNAPDTMALQEKRSLVEFGWNRRLLRCVGLWPEERGSLWRPSSSPNALLQQGLLLAMVSCELAALRRFWGGELSHTTVNACIVLLVAVAFCKAASLLSLRRPIMQLMHTLSDTSLPQNESEARIYSGAASHARLLTLVLCVDYAIVAVIWDLLPLVNFLSQSHQGRYNNSDAIFSQYPIIALHPWAVQSGPAYAFTFALQVVGGGIFTMTHLACDTFLLSLVIYICSQMDVLCASLQQLGRRADGAGAAGSGGGQRKAPSDQCSEPELYRELVACMKHHQNIMAYVGVLQQVLSPVALAQFMCSMLIICLSGFGMAISNDFGTLFRYSVYFTAAAIQLLLFCWYGEVLITKSERVSEAAMGCGWTEARGRRFTSSALLIMTRAQRPLALTGGKFYVVSLKTFVQLLNASYSFFAVLRQLNESGHREDDGVLDSLW

>CkiaOR7

HVIHQAAMAAGRRRLRGDSRAAYTFTMGFVVCGHVTVASWSLLPLLLKPVDKLSLPLVAWTPFDSSRGIGFLVTYVYQFTCTLFMAWTSGATDLICVNVVMQLCSHLGILCSHLESVGAQCCGDQTQFCNHGYLGKSGESPAPLKDDLRDQLSRCIRYHQEMISVAREMDNLMWTIVLSQCLCGMAVLCLLLFQMALYTLTIETIAKYMSYMASMLLQIFCYCWFGDNLSSKSTAVARTAYTCDWTSGSSGFGRSLSILMARAQRPLVVSGGSFYVLSSEAFISMLNASYSYFAILYSMSDEW

>CkiaOR8

MRHPRAGRWASRAFRLLNAAAVATVVVFNCSLALGIWRGAANNLDRFTHFSSHCNTITMWILRLGIIAAHERSFHHLAGQVERDFVEFMAAEDIPLLRDRGLRLRLVVRAYLWWGFAGCMWWLLYPVLCFGLTVEGMPYMMAVPYDVSQPLVFIANLLFCTVATLHVAVMTMVSDSYSVSLMVQLRLQLEILSKNLVAMARGDEETGQEKCIGKGPPTGTSQLQDSMRQNIRHHQAIISNTELLEKCMGTTLLAQCLSIGASVCIQLYQIAMHAQRLADAGKFGCYLFIMLAQLFVYCWFGDDFMTESLKVSTAAYDSVTSLEGCSSSSKSSLLLVMQRAQSPLRITAAGLFPLSSEAFVAVVNMSYSFFAILRNFKDESVI

>CkiaOR9

MVSKMERAASPLPATRPPPPLEYLLRLLHWTGVMRHPRADSWSGRAFYIRKVVVSVAISAFLISQALALWTGGTADIDRFTLLLCTFNTVLAWLFRLGHIALHERELHYLTAQVDRDFGDFLRPRDVHLVQTHDRHLRRIVLAYIWFSIAGCLWWIMFPLLRFGFCAEGLPFIMELPYEVTSPAAFIPTWFFCSILTLHPTPMTMAVDSFNVSLVAQLRVQLTLLSSNIIELANEDKDRSPVLVKHISSGNDLSSESVNGIHIARRLRKMILHHQAIISNTELLEKCLGVMLLGQSLSIGTSVCFQLFQVALSAGSVAEAGKFGCYLSVMLAQLFVYCWFGDDLITESEKVSLAAYSAVTSLHGIPIPQKRSLLLLMVRAQSPLRMTAGGFFPLSSESFVSVVNMSYSFFAILRNFKGE

>CkiaOR10

MDPAMSTASDAEEVTSLDYLLWPLHWTGAMCHPKAGPWACRVSYLLSFLAVVATSYADGCDVVAVCQEGISDFDRFTMTLSVFETGNTWVYRRCHLTWNERKFQKFAQQVRDDFGEFMTPDDIPVLRILADPLRRFVTAYLMMGVFSVVVWLSCPTSGEGLPLLIFLPFQTDHSFGWFTGWLICAYITADAVMVNFMVDCLNVCLMEQLRTQQIVLSKHIAELGNEFGSTEYSLLEKDSGLEFGNNFAKYRDSCKPQGADYQKLHTKASLEKEGAEGSQQPTEAANSSDIHSRLRGIILHHQAILSNGESLQKCLGNMLLVQSLSLGSVLCIILVQMSLSAQGARETTKICGYLFMMLGELLLYCWFGDKLTSESDNLTLAMYDAVTSLDESPTSIKRSLLLLLLSSQSPLCITAAGFFPLSKESFVSILNISYSCFTVLRNFKEE

>CkiaOR11

MPPAVPLEQSPGEAAQVACDLGYLLRFLHWTGTMRDPRAGPLASRAYYAANAAVTLAFVYFVCSQAVVLFRAGTADLDNFTLTLSLIDTQGMWLLRISHIAALEPHFHSLAYQVGRDFGQFASAEDVPLLRAGSRRMRKVMLLYLAFGLAECCVWLTAPASETGLPFVLALPYDVTRPVAYVATAVYCCFITLHTIMANFAADAFNASLIVQLRMQLALLNSNIVNINKVAEEPSSSAYKSADALQPYQSFSNSDVYYRLRKNMLHHQAIISNVELLQSCLGSILLGQSLSIGISVCFQLYQVAKSADSLQDAGKYSSYLFTMFAELFVYCWFADDLMSESENVAQAAYDAVPSLLEYPTSVKRSLLILLHRAQRPLSITAAGLFPLSSESFVSIVNVSYSFFAILRNFKED

>CkiaOR12

FASAEDVPLLRAGSRRMRKVMLLYLAFGLAECCVWLTAPASETGLPFVLALPYDASRPLAFAATWLFSVYIVFCVHMGTMAADSFNVTVILQLHNQLDLLGSNLRSLNNSISDMKYFPIHTLPVKYKQHSDDSRDIHYRLRKSVLHHQAIISNVQLLERCLGGMLLGQSLSIGTSFCLQLFQAATSAKRVQELGKTCSYLVTAFSMLFMYCWFGDDLISESEKLAFSAYDAVTSLQTCPTSIKRSLLLLMMSAQSPLRLTAGGFFSLSSESFVAVLNASYSFFAILRNFKEDW

>CkiaOR13

MTAAMSSAPQPPSSLAAAASAASDMRHLLRPLHWTGVLRHPRFAGSSPLLFRLYTAALASFALVFICSETAALIHEGTADMDVVMLLLSTINTASIWIFRMVHIAVFERDFHKLALQVGHDFAEFLTWDDLPVLRSQSRLVRRFALTYIWFGVGACAYYLVSPVSAEGLPFILALPFDASQPLGFAVTWVFCSVVTLHAVVMTMVLDSFNASVIAQLRLQLKLLSGKIVSLAMEMSEGDVDSSETTAYRELHYRLEKCIRHHEAIIKNADLLESSLGTMLLAQCISIGASTCFQMF

>CkiaOR14

MASSAAPRSVTSAAEGASDLNHLLRPLHWTATLRHPRSAATSPLIFRLCKIVMTCIIFSFFCSEATVLYRVGTGDLDIFTMTIGVADTNFIWCCRMLHISVCERAFHKLSLQVGQDFAEFLTCEDIPVLRAQSLVVRRFTQMYVWWGVGCVVYYLFSPASDAGLPVILALPYDMNQPLTYAVTWLYVAMATFHVQVMTMVFDSFNVSIMAQLRTQLTLLSNKVVVLAKEMSEKPVHSAETTAYRELHCRLEKCVRHHQAIIKNSDLLEKTMGPMLLAQCLAIGTCACFQMFQVATNTNGLQETGKYGAHLVVMLAELFEYCWFGEGLITESENLALAAYDAVTSLEDCPISIKRSLLLMLQRAQRPLCITAGGFFPLTSESFVSVVNVSYSFFAILRNFKNEEE

>CkiaOR15

AAAGAASDLSHLLRPLHWAAVLRHPRSSPLCFRLCTIAMASFAFTSTCSEVTVLFRDGTADLDAFTLTLSVVDTNTIWLFRMAHTVACERAYHKLAHQVGHDFGEFMTWDDMPVLRAQCRVVRRFTLAYIWFGVGACAYYLVSPVSAEGLPFILALPFDATQPLNFAGAWLFCTMTCMHVVVMTMVLDSLNVSLIAQLRMQLALLSKNIVSLAKEMSDSPMHSLGTSSYQDLNHRLKKCVLHHQVIISNADLLESRLGAMLLAQSMSIGSVACFQMFQIVTSGSGGQVGKFGCYLSTTLTELFVYCWFGDDLITESENLALAAYDALTLLQGCPVSIKRSLLLLMQRAQRPLCITAGGFFPLSS

>CkiaOR16

MALHSSLSLCVRLLRLAGLAPPDSCRSWRSPAGLLYRTYTTIAVGLVGHVLMPQAAGLVHYWGDMKVATENLCLLFGYATACYKLLAILLRRPRILSFVSEVDARVTAMASASAEARAVMSRSDRWSRSLAVFMVVQSTSTAFIWSMNSLRMSLLMGSSQRTLPMISWYPYDMTVWYNYTVTFVIQFLSLITVAFCNSTMDILIITLMCQMSSLLEMLNLKFRDISEDNWLQADKSSTEANPNYIWQSAASVFSTTTGPNAVLEYVRTSISENVSQSEMYTKLTSCMEVHQEIMSCAKELEMLMNDMFLIDFVCCMMVVCSTLYVSTSESEKLGDLMSHFGYLVAMTYPVLLYCLSAHDIMDQSERVSLSAYCSPWPQAAASYRRALCIVTCRGQRPLTLTAGKFYVISRATFLTLMNASYSYYQMLREINVKRSD

>CkiaOR17

MTVRVPWTGSLFSVNARILAVAGICAPPGSRLYLVYTGWMLFTQLSFFAAQVQGLWYFWGDVDKMTLDACLMVAEVLGIIKIATFLRSQSDFYSIVNKLDDVVTRQRHSGDAEVRSILEASSKTASALTVCVTLLGCPSPAVWTASPFLMALVGGGGGAAHSSLPAAARYTAFDTQSPWFEALTALQFFSMQYSFFTTVGVDMLVVSIMIHASAQLEVLNVVFCSLGKSLGSSVENSDPGASSSYSANIYSCAGSYGMSRQANSAETTTKKKFYGELSDCIRHHQDVMQLVADVERLLTSMILTQVLGATLIICVTLFQFATNIDNIGTVLSVSVYMSFMMYEVFMYCWYAHNIIDQSCRIAESAYCCGWPGAPASLQRAVMVVICRAQRPLALTAGKFYLVSRATFMQLINASYTYYALLSQMSDHW

>CkiaOR18

MSARVPWRDSVLWANARLLCVAGVWPPPGHGGSYLLYTCWLFASQCFMIAGQLAGLWYFRADMDKVTLDVCLTVTVVMGVIKGGVIVAHKWRFFSIVNRLDDATAAQLEEGDPEVAAVINSTVRLSRTITVMVPVMGSLSPVMWGLTPLLLRLLGGSQKPELPVVCWYGSWDTVSPYYEVLYVVQFVTMQGGYLVVMGSDLFFMTLMMHAAAQLRMLNMKLVKILNNEAEVKGKHFEGIKCVPGYHNKDLKSIETSDKGWNSPAKHQHNLSDETSSYDELRLWVEQHKDVIKLVQHLEQLLNMIILFQFLGGTMIICVTLYQSSAKTGEVTTLFKLQLYLGTMLSEMFMYCWYADGMVQQSARLATSAYSCGWPDAPQPFRRSVLIIMRRTQRPLFLTAGKFYTISSATFVSLINASYSYYALLRQMNDHW

>CkiaOR19

MKEEETPWSDTCLWLIARVLALGGSWRPPRARGFALYRAWVLFTQFSFLIGQLQGLYYFWGDTNSIIQDVCLLVTTILGLFKFFVFVVKQEDVFHIVRAIDDRRRDQSKLGNPSVASILEESYRSASTITVYMAGLGGTAPAVWAVMPLVMSRLGVGPPDRELPAMAWYTGRDTATPVYQLLYVLQYFSMQYSYFAAMCLDLFFACLIIHVAAQLEVLNVRLSQIREDYYRNEQTREDSKVDHAEEDSAWKELSECVEHHKDALKLVDDLESLVNPIILSQFMGATIIICVTLFLITTNKQHVVALVSLQAYLAVVVYEMFMYCWFGDDVMYQNSRLVSSVY

>CkiaOR20

MKEEETPWSDTCLWLIARVLALGGSWRPPRARGFALYRAWVLFTQFSFLYGQMRGVFSFWGDATRVIQDVCLLVTTILGIFKFLVFVARQEDVFRIVNTIDARRREQSALNNPRIDAILYASYSSARRITVHMALVGGWLPGVFAVTPFVLRGLGVGPAERELPATARYTDRDTATPVYELLSLLQYFSMQYSHFAAMCIDLFFACLIIHVAAQLEVLNVSISQIREDYYRNGDAGAGASDEEAAEEAAWNELCECVDHHKAAIQLVGDLESLANIVILTQFMGATMMMCVTLFLITTSKQHFAALVKLQGYLVVVVYEMFMYCWFGDDVMYQNSRLATSVYTCGWPGAPQKLQKALIIILMRAQSPLGVTAGKFYYVSRGAFVSLMKASYSYYALLNQMNKW

>CkiaOR21

MPETMPWSHTALSMNVRLLAIGGMWSPPWCQPKWYLLYSTWVFFCLFSFFVGQVQGFWYFWGNMDKITHDVCLNIGMILSFTKYFIFVLSQQDYHRLVQKTDGISAEQTNTGDSEMTSILDASYSSASTMTLYMTCLGGSSPGIWAVIPTIMRKLGMFPPESELPATAYYFSSDTETPYYQMLCVLQFFSMQYGFFAEVGPDLFFASIMIHTAGHLEILNASLSRIGQSTGNIGANSDKQDNAQEKYFDEIPCEELVWSDLCTFIRHHQEVMELIKEIESLMSKIVLVHFTGATIVICVTLYQSSQNTENIAALLMLQVYLGVVIYEVFMYCWYAGDMLYQNSQLATSAYSCGWPGASPKLQRALAFIICSTQSPVGLTAGKFYYVSREIFVRLMSASYSYYALLNQVNNKW

>CkiaOR22

DKITHDVCLMISMILSITKFFMFNLSGQELLRLVRKMDNTSAEQSNSGDSETVSILDASYSSASAVTLSMTCFGPVPAIWAIIPIIMRKVGMFPPERELPGTSWYTGSDSESPIYETLYVLQYFSMQNSFFTAVGPDLLFVSMIVHAAGQLEVLNASLSRVGEMTNPHKQLKPQEELPHEVCCEEMAWTDLCSCISHHQAIMKLINEIESMVSKMVLLQFLGATVMICVTLYQSSKHTENMAALLMLQGYLGLIMYEVFMYCWHAEDMLYQLMSASYSYYALLRQVNDK

>CkiaOR23

MQFRLSSDSVMRWNVIHLQLAGLWPVENSRLFSLYTAWLFFTQCHIVVLESIDLCTYTGDFSSAVLNICAMATFFANVCKMFHFQFRKGDYFRLVTELDAFVSEQRTKYSHNETVKELLDTSTKRTSSITKAVMVYLMVWTFMVVPALFLMEAPYGVFPLMAWYPFTADMWPRYEIMMTLHFLTIGYCFFTSWGMDLFFGCLMYHLSLQLRLLNYHLANIRCSSKADRILEKSLPEIEALSTSHVERETTKTAQQKQFEGEVYESSAEDVMYDDLLQCIKHHQSMIRYAESVENVANPVMLSQFVVSVLVLCVVLFQTSSELETLTAFVSFLVYLLELLLQIFIYCWVAHQIFEESEHVGEAAYNSDWPDGSASYKAALRLVMRRSQRPITISAGTIYAIDSTTFVSIVNASYSYYAVLRQINN

>CkiaOR24

MYEVPDKGLTSLPGPNSVINVNIGVLKMAGLWPTRPYGLFAAYTVWMYLTQWAVFALDFMSLFYYWGNLNMMTAVFCNLTSMTAGIIKMTHFFVYKPKYYMLVNKLDAMVASQQQITDTNVNSKSILVETSKLNKYSTYIIVTYGNLVGVPWIILPFVMDIGDTESTLPVVEWYGITQDKSPVFEIGYVLQCLTMMYWFFASWGLDLFFGSLMIHLAGQFKILNKRIASVGRKVDSALETSSSGETERKMINSGEVSKSTNVQQQETALYSDLHTCIKDHQEMMSFMTDLEETVNFVVLVQFMAGTLVICVNLFQAALNVQDFSSVLKVCMYMFELMLQLFMYCWCAHDVMVESERLSTSAYFSEWTGAPRRFTTALHILMARAQKPLTISAGRMYTINSSTFVSLINASYSYYAMLRQMSDRW

>CkiaOR25

MEDSLSARHRESVLKWNVWVLSTGGLWPAGPPRLFAVFTSFVFIVKWTHVLMAVRTLYLSWGDLNEITLTLLSMITMLGGSVKMTIFLKNKTVYYQLVQSLDDVVRYQQQYYLGNETMVTTFQKASKKALRLTFITLGYLNVLGPLWFVMPLLENSSEKHLPFIPMHGLNFTSVPLYELSYATQCTATFFWHLVSVGLDMFYASVMMHVTAQLTILNLRFKNLRLESEDFSGRSNLSSFTASLDNSELAHAHIKMYKELCDLVSSHQKIIEFTNYLENVMNSTVLVQFLSSVLVACVTLFQATINSEGNTVVKCWLYLPMPAFQIFVYCWCAHDLMDQGLEVCTSAFLSAWVEGTRGLRRGLLVVMVRASSPLELTAGRLHPISRATFVSLINASYTYYAVLRRVNDR

>CkiaOR26

MLSEAHGVLQNNVRVLQLSGLWPPTPRRMRGWALVFPLYTAALYACFLAVLAVVLQLAYLSQRDINDLTYALIVVMSHVGVLFKMTHFLYNSAAYLQLVHRLNGLVRQSVTDVQSDPQGVLSACHMKAMRLTFYTFAYLALTGLTWYLVPIVDAIRSGSEGSRLPVVNPQWMDNTDTALYAVGYVVQFPSIFYFVALSVGLDGFFATTMIHVAAQLQLLSLRLSRLNEGGSPLSALTTSSDEHKRHVSSTAVLGMQGIDGSEGMYQQLVQEMKRHQEIVSFVKSLEAVMSPVAFVQFLFSVGSICVTLFQSTFNPRPDVVLKCAMYLPTPAFQMYMYCWCGHDMMEEGASVSLAAYSCAWTGASKRSKDALRMLACSTQRPLLLSAGKIYPVSKATFLSMMNASYSLFAVLQQMSARW

>CkiaOR27

AVFTSSHRKALWLIWAPMVYLNILGPVWFMMPVVAWASGAPGRQFPFANVRGVVKTNFPLYITVYFMQCHSVFYWNFLSFGLDMFFVTCMIYMAAQLHMMGKRLSNVGSGAKVDGNSGVDNEKSSLQKLEDKSYKPLEADGSSEESLAELVDCIKAHHHILSFLLVLQDVMSPVAMAQFVCSATAACITLFQATFNPEGNSIFKCLMYLPMPAFQMYMYCWGGHEMIDEGSSLSVSAYSCAWIGATRRFTTALHILMCSAQKPLILTAGKLYPVNRDTFVSLMNGSYSFYALLRQMSGHW

>CkiaOR28

MVSLVQKASQMPPADVLSTNVRILRWSGLWPPEARGCWARLFAAYSAHAFLSQVVATAMTLHLIYHSWGDIYEITITMMVTMTLVGGVLKMAHFFGNADTYYSLVRDLRELIDLQSGLCERDETVAGIFCSYHRQAIRFTCGALGYLNILAPTWFLMPVISGAANDPTDSKLPFSQLKGLRSDDLLGYSMAYFVQCHAIFYWNFISVGLDVFFATAMLHAAGQLKMLSHRLSSLGKGPNGHDEQTTHWNDLRSDTTQVSQMVPYQETKDLYSELRSCIKDHQEILRLVLFLETVMGPVAFIQFLCSVVAACVALFQATFNAEGNGVLKCTMYLPTPAFQMFIYCWCGHEMMEEGLAVSMAAYSSGWVGAGHRVTKALRVVMCRAQRPLLLTAGKLYPV

>CkiaOR29

MVVLEASASQTLGESGEPWDVLRRNVKVLRFGGVWRPASRSGWRPCLFPLYFASVCGSLLNIMTLDMVSSWLLWGDMTAVTFALVSAMTNLNGVVKMVHCFSHHDTYSRLVSDLNGLVALQRRYCESNGALKAAFQSACRRAARLTVGCLAYMNVLGQMWCVVPLLSAEPPDARESALPLVSLPGLRSDNRAWYSFAYLVESHAVFYWNFASLGMDMFFASAMIHVTGQLNMLNIRLAQLSREGSTEDQLQPSSSTLNGNNRPQERLAGDSATMYGELCECVKHHQAMLSYLEFLESVMSPVALTQFLCSVVAVCVTLYQMTFNPEGSGVIKCAMFLPIPALQMFVYCWCGHDMMEAGLSVSLAAYSCAWVGVGQRVTSALRVVMCSAQRPLQLTAGKVYPVNSDTFLSLMNASYTFYTLLRQMRNR

>CkiaOR30

MVAIGSRAKPMLVQQSKSTSKGKDAGDEYGSSPSENVVRRNIRILRMAAVWRPTGRWRSRVYPLYFGSVCASMLHIGALAILRSATIWGNMTEVTFALVSGLTCFNGAVKMIHHYTHSEAYYRLVDELNILMDRQRPHCEGDAELDEALQNAHRKAKRLTWGVLLYMFVLGQMWCIVPLFMQFPPDDPSSPLPLVTITRVHKVHNHTLYSLAYLSECHTVLYWNWSSLGMDVFFGSIMIHVTGQLNMLNMRLSRLRGGGPGEDLSQYSSFGKGGEQLKGDIRDSASMYAELCECVKDHQEMLSYLDFLENLMNPVPLAQFLLCVGGMCLTLYQMTFNPDDGGVIECILFLPIPALQMFIYCWAGHGMMEESEYVSFAAYSCRWGGSDRKVTDVLRIVMSSAHSATMLTAGKVHPINSDTFLSLLNASYSFYTLLRQMKNLEEENEASS

>CkiaOR31

MAGRKSGRSLCADDVLRHNVKQLRLGGAWPPDSRRGLRRLFPLYTATIYFCQSATIAMAVLLTYDLWGDVDAIMLTYVNTFTLIGGFIKLIYFSSDVRGYRELVAVLRDVTSQQWPHCEAHAELMAMFTAGYRKASWLTFGPLVYLNMLGPTWFFMPLIVRATTGSKERLLPFVNMRDSVTEIFPLYVAIYVVQVYCMFFWNMISVGLDMFFVACMIHVATQLSILNARLSSLGEDSSDEACSAGLNRNIIREFGSSQKRPFSRDGRTSDMYEELRNCIKTHQHIRSSLKTLQRMMSPVAMTQFLCSASGVCITLFQATFNPEGNSTLKCLMFLPMPAFQMFIYCWAGHEMVYQEELLSLSGYRSVWVGTGSRISALLHILMCNAQKPLQLTAGKFYLINRDTFVTLMNASYTFYTLMRQTSDEGSMAQT

>CkiaOR32

MESSTKYDEGVSGERGGGRWTLWKDPETASSVLSLNVRCLLLGGLWSKSKGLLYLAYSAFIQLCSVVYIVMCMLSMFSPEGDINDVTLTLMHTFEIVCGILKAVIFFLKRDEYYQIVHNLDRLVSSQRQYLTASKDEHLLMLLDAAHKKTNFLTLVLTGYIYGLVFVWLPFPLMLMPSESLMPFVQMPGNYFKEHFSVYMIAYAIQSFVPLALIMVVDGLDCFFVSSVVHAEALLKVLSERIASLGHPHYTDFSLQRFSDMERESRKSNVENNADMTAELRSCIMHHQEIMEFLQSLEKAMNIMVLMQLSFAMFNLCMALYQQTKMPNFTSALKYVIYVPFPTMKMFFYCWAAHNVKEQGEEVSWAAYNCAWPDTNQEFRKSLAITMCSAQSPLLLTAGRMYPINKDAFVSLLKGSYSYYTLLRQFESKW

>CkiaOR33

MERSDDEATEGSVLTWRETADSVLKYNIRQLCVFGVWPLPGSALFHAYTVFVCAIGLAGLAQDLAGVYACWDDLQEVTMALIHILSVSSGFVKLAYFVCRRPVLNALVRRTGQLVSAQSHFCDSDERLRATFSASHSKAVYITLLAYGYLSVQGCMWFPLPMIAYPGERKLPFMQMPGATTGSIYVYVLLYSLQCLSSMFVTFIGVTVDCFFAVVMIHTAVQFSILNARIEALRPDVAGSASTSITVNDQSKYHDNMYNQLCQCIQTHQRLLSFVSYLDSVMNPVAMTQFTFGVMVVGMTLFQASDSPSSSTIFKCATWLPMPGTQIFMYCWGAHDVMDQGEAVGGALYRCGWVDAGGSLKRAMLIIMSCARKPVSLTAGRVYAINSATFISLMNAAYSYYTLLRQVNGRE

>CkiaOR34

MEKQKESSFQVQDLGSGDGEDLSLDEVECSVLSQNIRLLYYMGLWPMESSWAYHCYTVFNLTSSAAIMLMNVIGVCYSLSDIDQVTGALSTILPMSGGLINGLFLLKQSPTICSMVRTIDRLVASQRQYFQRDAQLDAIVARARSQTLLVTVGVSGYLFAIASYWIVIAFTLPPSLSVLPFVQLPWMPSSGLGLFWFTFAAQLYTAPFCSYTTLFVQFFFLAVMLHLSAQFKVLGSRFASLGRSLPSKAAVDSDVVYEELRLCVKTHQELLSFVSFLDDVMSPFAMMQFVAGTLAVCVVLFQAANNQDLNTNLKCAGWLPGPSLELFIYCGGAHEVVHGGEALVEAAYDCLWYNVAPRVGRTVRLVITRAQVPPVLTAGHLYPITSPTFVSLVNAAYSYYALLSQMQNKW

>CkiaOR35

MIAEVGQTWKSTGGSVLKYNVRLLFLCGLWPLRRGRLFAAFTAAVLVAAALHAAGAVVGLCSEPGGLQEVTLALANLFVVSSAIVKSCFFLADRTSFCALVSTLDRLVLENGERVVVDALLSRRLTGSRRSAVRLTLSFHVYVLSALVGWSLMPALKHQRSLPFQRLRWLDTSSAAVYGASYALQCTATCFCSFINTHLDVFFMAVMIHVADQFSILAARFADLRLDADADSEGQSVSLGEELYRELSRCIQSHQDLVSLVQLLDDVMSPMATTQFVVGAVNACMVLFPATYSTDVGAVLKCWAALPMVGIQIFLYCSGAHDIMEQAETVSTAAYGCSWLGAGQRRRRALLLVMSRAQRPLQLTAGKLYPINSPTFLSLLQVTYSYYTLLQNLNSR

>CkiaOR36

MAEWEAEKSPGQSVVGTNTRLLCTLGLWQPVESALFHAYTAVVLAMGVGHLALASVGLWLRPGGLAEVTLGLANLFVIFTALSKSVLLLSRRPLFYELVRRVDETAADQREFCGGDALLQLVQSSQARVSRLSLFLHWYVMFAVVSWALIPLLAPSGVRVWPFQQLPEEPWASSPLYELSYALQCAGTIYFSLINMNTDSFFMAVMTHMSLQFRILASRFAKLNSDELALAETKPSTVRVLSPKSPMSAHITDQELRACMQTHQKLLSLVSFLNVVMSPMAMMQMAVGVINSCMVLFPATYSEDDAAVMKCWGALPLLATQVFLYCSGAQNLMDQAEAVSTAAYSCGWVDCSWQLRRSLLLVICRAQRPLGLTAGSMFIINNATFLSAAELPQRGHEPRGHGAARRRRRQLLHGALPSHMQHGQRGRHEVLGGPASAGHSAVPLLLGGAAPHGPGW

>CkiaOR37

MGWYSSEDQPLTWQYTGDSALKYDVRILHMIGLWPLRASKLYRCLVTAIITLCFGNFVEAVISLYTLHGDLEDFTLSLSNLAVVIVGILKVSFFLRHERDYCRLVRWLDALVVSQSVYTRGRPQLEHAFTGDHSLATRITSWFCVYNASVVLTWVLAPLAAPPEAKSLPFQQLPFAEGSPFSLYALSYA

>CkiaOR38

MDWDPKETKPLTWQYTARSVLKYDLRILHLLCLWPLPGSLFFRTLTAFFVALCLGHFAEGVVNLCTLSGDMEDYTLALSNFSVVTIGVVKMTFFLRNERSYCHLVRWLDALVAAESESAGGRQLMEAILPAAQKRSVRVAAGLLLYNCFLLFIWLTAPLAAPSEASMLPLQQLPLTDANAYPLYELSYALQALSTVFVGLINVHLDCFFMVAMIQTAALLKSLSSRLADLQVRNTLSSPKVNEQGKNLMTTDDMYTELCLCISTHQEITSF

>CkiaOR39

MQAKDSVSKSGAVSASFRPADGGSEDKDGDSAQPTESQESILTWKGTSQSVLKHNVRYLCVLGAWPLTRSRLYHLLSGTAFLLGVMHIGVAVFGAYLYSDNIEEMTLMLANLFLVCSGITKLVLFLVYRNKYRQLVLVTDEVTTSQRSYCEGDAALKSILLDSESLTARLTLFVPAYMATLSIVWVPMPLMAYDERSLPFVQLPMVNQVSSLMYALLYVMQSVPSLLFFNIGFGVDAFFASVTINAATQLRLLFLRIKDLRIGGSDAVKLSIGDDISHESMFGELCTCIQMHQQLVSYLTFVGKVMDPIAMTQVVFSVLIACVTLFQATYSADSNAAFSCVGFLPTPGAQVFLYCWGAHNLMEQSAAVSEAAYSCCWVEASGRFKRALCLLMCRAQSPLVLTAGGLLRINRPTFISLLKASYSYYTLLGRINNR

>CkiaOR40

MKDEEPSASVEDGSPLTWEYTRRSLLQLNVRLLWALGVWPLPASWRFSLLKAWLALLAVGNAIENVLGVWKNWGDLTEVTYSLLNAFTIGAGVAKTCHLAFYQERYCVLVRRVDLLARSQRGYCDADPAMRGVTLSCRRTARRVTVAAFTYLTVLCLIWMFMPLVAHPGERQLPFNHMPWEPQSFPLFYEISYAVQSASSVMYVFISFALDCFFAVVMIFVTEQLMVLNLRIKQLYARPAGDGSPLVSKQFLDKMSMNSDEEMYKELCLCIDTHKDIMSLISFLDTVMNPIVLIQFMLSVMAACVTLFLESYSADGSSVLNSVSYLPTPGIQVYLYCWSAHNVLEEGAAVSEAAYGCAWYEGSGRFKRALRTVMCRAHKPLLVTAGSLYPISSATFVSLVNASYTYYALLSRVHNRDAING

>CkiaOR41

MKDDEGRASFSDSSAMTWDYTKKSLLWLNVRLLWAFGVWPLSSSWFRYVTKCMLFMLAVGNALENTLGVWANLGDMEEVTYSLMNAFTMAAGIAKTFHFVLFQGSYCLLVRRVDSLVLSQREYIEGDPEMLAVTRRCRKMALRMTVAAFAYLTALCLIWALFPVVVHPGERWLPFNHFPLEPAPLPLYYELSYAIQSASSLLYIQVSFGVDFFFTVVMILITEQLMILNARLAQLHLYAGGGKSTATSVSSMAASEDRDEMYEELCLCIDTHKEMMSLISFLDSVMNPIVLTQFTLSVMAACLTLYQQTYSPDGNSVMKSASYLPTPGIQVFVYCWGAHSILEQAEAVSEAAYSCAWFDGSPRFKRALRMLMCCAQKPLVVTAG

>CkiaOR42

MPTSTTTKPMTWSESGNSILKVNISELCLSGVWPLTDWKLFRAYSVVIWILGLENMVEAMVGIYLSNGDLEQMTLVLPNTFTTAGGVFKMAFFLSDPHSYNVLVRLMDELLSDSIQYSTGDHQMTAIASEARSSAQRLSVFIYAFVSTQIIIWFPMPLIAYAGEGKLPFMQHPWINSTTFPAYDTMYALQCLSSGFHIFISLGMDCFFAVVMIHSTACLKMLYLRITALRSGNARSSEGPATGMRSEDRESLAHDEMYKNLRACMCSHQKIIGFISYLETVMNPMAMTQFAFSVLVACVALYQATYSQDVSAACRCAAFIPTPGAQVFLYCWAAHNVMEQGLAVSAAAYDSSWVSGDGRFKRALRILMCRAEKPLVLTAGHLYPINSPAFLSLVNASYSYYALLGRLQCRW

>CkiaOR43

MKEKGGRQWEDSAEADDTATLTWRETASSVLKVNVRGLALFGSWPLPESRLYHAFFAVVFASNLGNIAEAAVGLYMGHGGLGEITLVLPNTLTTAAGVFKMVFLYRDRGRYYGLVSRTDLLTTSQLGVPGHDAAAVVREASRHSLKLTYSVFAFVSLQMVVWFPMPLYAYAGQRKLPFVQL

>CkiaOR44

MSSALGNDMGKPLSWAESSGSVLKLNIRHLWLCGVWPLPGSWKFYAYAASVLGLGVWNAVEGPLAVYFCWGNLEQTTMVLMNTFTNGSGLVKMAFFLRNTRQFNSVVRHVDALTSEQAEACSADAPLGRIFQASRKRAFGLSLGMVLFMASQGLVWFPMPLVAHPGERLLPFVQHFWDNNSNYYELSYAAQCVAGLWMAEISFGMDCLFASTMMLAAAQLEILSGRVLKLGQRSLVGNKSNPAAEEMYSELCRCVESHQKILSFVSRLQETMSPIAMTQFVCSALVLCVTLFQATYNKDIITSLSSMTFLSNPCGQVYLYCWAAHNVADKANAVSTAAYSCSWVEGSARFKRAVSILISRAQKP

>CkiaOR45

MKDSEFSTMDDKLSWTVSSKSVLKLNIRHLWLLGIWELDQSSVFKLQSTIAFGLSIWSTVECIMAVYFIWGDLEQTTLVLLITFTCASGVVKTFIFVYDSRQYDSLVRQLDTGALMKDSEFSTMDDKLSWTVSSKSVLKLNIRHLWLLGIWELDQSSVFKLQSTIAFGLSIWSTVECIMAVYFIWGDLEQTTLVLLITFTCASGVVKTFIFVYDSRQYDSLVRQLDKLMALQRGCCSEDPNLAAILDWSRRKATRLTLGLLVFMLSQSLVWYFVPLIAHPEERRLPFVQHQWDNNSVYELSYSVQCLSAVWVSQISFGVDCIFVSAMVLVAAQLEVLAMRIEGIAAGASSSYSAKAKYCEKELSSSGTDEM

>CkiaOR46

MGHGQEEDLEKTLSWSESNQSVLKLNIRHLWMFGLWPLFESCLFHLYTFYGYMLGLWNFMECLVAIYFAWGDMDEVTLLFMTIASNLNGLIKMTFFLYDRRRYNSLARRVGALVSVQSDVCGSEPALAAILTSCQSRAFRLTLALLLFMFSQCFVWFPMPLMAHPELSRLPFAQHAWDDNANLYGLSYFAQCAAGLWMTQMSFGMDCLFASVMILLAAQLDILAQRIVALSKDDYKEKGGYPEKKPAPSFGDEMYDDLCLCIQSHQKMLSFVTHLQNTMSPIAMTQFAFSVLCICLGLFQATFSEDFSAVFKCASFLPIPCAHVFLYCWAANNVTVQAEAVSAAAYGSSWVEASERFKHAISILVSRAQKPLVLTAGHLYPMDSEAFLSLVNASYSYYALLSQMNNR

>CkiaOR47

MESYQRKEMMKTLSWSESGKSVLHLNIRHLWFFGVWTLGQCPGFKVYTGFAIAMGVWSVVECALAVYFTWGQLGETTLVLMFTSTCGCGIVKTLFFVRDEQSYSLMVQQVANLLALQNEAICKDPALAAMLQDSRSRVFRLTLGMLLFMFSQCFIWFPIPLVVYAGERSLPFPQHAWDNNSNYYALSYTQQCVSALYMAQISFGLDCLFASIMILVAAQLKILSGRILKLKKEVVPAEESESALQKHQMAVDKDHSKIYESLCFCINSHQKILSFVADLQDTMSPMAMTQFATSVVILCMALFQATYGEDMSASVKCASYLPIPGGQVYLYCWAADSLSENGESVSTAAYNCPWVDSNASFKHTMSTLMVRAQKPLVLTAGGLYPINSEAFLTMKIGMATLHAIGELYSQPSADITA

>CkiaOR48

MILAAAQLRILTLRIAGLKTENCGVKPEDSASCVHGVQANTRDKMYENLCLCIESHQKILSFLKHLESTMSSVVMTQFCCSVLVACVALFQATYSTDFTAVLKCASFLPVPGGQVFLYCWAAHNVTEQAEAVTMAAYSCSWVEASGRFKRALSILISRAQKPLVLTTGHLYPIN

>CkiaOR49

MRGEDSGDVEGPLSWSDSGRSVIKLNIRHLWLFGAWPLGANRLYDLYTAFAFALGVWNTVEGVLAAYYIWGDLEETTLVFADTFTIASGLVKLAFYTRDRSLYSALARRLDDLLSVQKEVFSEDPELASIFEGSRKQAVRLTVGMLLMMLSQGFVWFPIPAIAHPGEQRLPFAQHGWDNNTNYYGLSYAVQCVAGMYLSQISFSMDCLFAAIMMLMAAQLKIVSCRVRKVKADDITAQGEENVGETALSALQKPYTNLSRCIETHQNILSFIKRLQDTMSPMAMTQFFFSVIVACMALFQATYSKDFTAVFSCVAFMPMPCGQVLLYCLAAHNVTEQAEAVSYAAYSCCWVEAGPSLKHALSLVMCRAQKPLVL

>CkiaOR50

MAKHKDLPDPVVDLSWMSRALAPVGMWGPSGGSRLYDAYSIWTIVQLVLTAAGQMAGLQGHWDDLTTVFTSLCFDLTVTCTIIKGTIFVLQRESLDALSRHIERNAVEFCSHLPEERRALLVRARNLSRLIVCSFQSVGGVTLVSFITGPIVQNGRDRELLASGLSNVTVDRQLGHNYPMLMWWFGGLPVSSPGYEAAYLLMCYWLVLMYICTNVPDAYYVGLLNYISAQLRLLHIALRSIAHPEPGDLLTEKLSHTYGLTLKGDVPAKGGAQESSRDGVFASLLECIRFHQEIMKCVDEMESLLSLTVLIQFFTSTLVICLTAITVINTEAAYLPTYAAYLATMFYQLFIYCWYGGEVYLESESLQFSAYSCNWPDTDARFSKTLSICMARMQSPLNLTAYKFYKLSRETFLLLLNGSYSYFTLLLQMNQKND

>CkiaOR51

MSKYSVMIVELAWSSLGSDLRRRLVHAVEEGEVRVGTVDQQQEGLAGEPMELAGGELDGALQAAHHDAQRPLEAGVRVRPRAAVRRQLQLLALQEQLPAMPAVQEQLVEHGRQVCDVRVQVRSLRRNNSECCETDYERTGHELHEYSSAQNSFHLSCSLYNLLVEAYAFDEPVAGSALGPHVGTPARGLELPPPGSLAAASALSVRRRQRAGQRGVEQGELRRQVDDERD

>CkiaOR52

MEDGQLPQPVVDLSRPCLFLRAIGLWAPVRLRGLYSLYTVWSLLQLVVSVAGQLAGLQGHWDHLPTVAISVCLIVTSICTLFKASSFALRRGRVDALVSLVGSNLSTFCAHRPRASAAVVRAASDRASRMFDTFLGMGGVALVFFYLGPIMQNVKDNSILAKAAANETVDPLLGRNLAVLLWWPSGQPVDTPAYELTYVGVCYWLMLLYLSTSTVDAFYVTLIIYLSSQLKVLNVDFLSITEDAQDDSDSPAERTSKPEVEHKLTVKLPDGDHKTTTQSTQQSLLECIIFHQEVMKSVEEMESILSASILVQFLASSLVICFTAFVITTAENKQDLPTYITYLATMFYELFLYCWYGNELLSESERLQTSVYACGWVDEGPVLQRTLRVVMVRLQSPVCLTAGKFYKICRETFLLLLNGSYSYFALLHQMNDH

>CkiaOR53

LAWYASPLIANAALAQDPATNATLPSQLLFDAWFPFDPVPSPNYELALVYQSVTLYIAFITTAVIDVFYVSVMVYLGVELEILNEAVASSCRPPQEKEKDKDKEEGEGELGDDCSLLAACVRHHQHLNSCVQTLQEVMGISIFVQFVFNMLLICVYAFVLTTTKSDLGTLVKFAMTLESYLFENLLYCWFGNNLMEQSERLPFSAYSSAWPDGGRKFQQSLRLLALSAGRPLQVTVGSLYTLSSHTFLHLLNGSYSLFAVLHHLNSKW

>CkiaOR54

MVKAHKAGDGGKRLRTDLRLQQWLLRFVGAWAPRRGPSTSVWWRLYGAYSACIVAILMLFVASLLFAMVHYWGQMLGVTMNACLMFTYVMNIIKIVAFLSMSPAIDEFIDELDSCLQEFGQELRSQREVVFSWIALKSRIVSVARLSVTAMGCVYWSVMPAVRASACGDTVQCSASVGLPAHVWYPFSYTQSPVYEVIYAEVAAGLMYGALLSSIMDGFLVSLFIYMAGHLQLLNLMLRNMCGGQAEDEDHHGKSPHHLAAVHQEQLMSWRLAQCVTYHCHIDSSVQRLSTLFGPILLGQFLMDIMAIAATAFVAIAKNADSTWLVKYTSYLSAVIQQLLFYCWFGTDVLSESERLQTSAYSSDWVDASPRFRMELRIFLCRTHRPMRLTASKFYTISSETFLMLMNASLSYFAVLSEINAKW

>CkiaOR55

MLVAMSGCLYWTLAPGARAHACGGSVHCRGRVGLPAHVWYPFHFTHTPVYEVVYTLVASGLLSGALISCIVDAFFVSLIIYQAAHLQLLNLMLASVGNQQRSGPAASSDRKRSEAAGPEDLEDDMSRRLAECVCYHCDIDSCVQHLSSLVGPILLGQFLGDMVTISATAFVAMANNADSSWLLKYMSYLSSVIQQLLLYCWFGTDMMTQSERLQMSAYSSNWVSASPRFGSELLVFLCRAHRPLRLTASKFYTIS

>CkiaOR56

LHFWGDILSATNNACVTSSYSMAIFKFFAFRLMRRSVEHLVRDLDSCLQVYGNEYAAEKEAIFGACAQKARLVSRLQVSLGVSVYLSWVVLPAIRAQACHSTDCRVHTGFPALVWYSFSFTEPPVYQMMYAVICMGLFYGCVIFTSQDGFFWSLIIYVGAHLRFLNFMVTNMSSAEGDAKTPTGSPEMEENMSRRLSECVCYHNDMDSCVQRLSSLLGPVMLGQFLTDIVTISASAFVATMLKADSGWLLKYGSYLSGTIEHMFLFCWFGDDILTESERLPLSAYSSDWTDASPRFRKELRMFLCRSQRPLMLTASKFCAISRQTFLSLMNASYSYFALLNQLSSE

>CkiaOR57

MQKGSAQYSQASLEVRRGLDLSTCEHILHWFSCWRDGSVWYIAYVVAARVSVGAVVASQLGLLPHVWGDLYSTSLCVYLTLAVLSAWFKMVQFDLASPKVDALLQELSGELLLHADDPSEGTEDVFRRFQKRSGVFRRVFLVLGHLTILSWAVKPFLDRLLGSDGALSLPQDAWYPFDTAHSPYYEIAYLHQVVSLYVAATCVVTVDVFFTTLFMYITAQLHVLNLHLQAMDCRPQLTEDADKVASWEKRVTAQKSAGADGLPSADPPDAYGQLLRSVQHHQVIMRSVSIMEDAMGPCVFVQFLFNVMVICIYAFVMAGVHNMNMGSLIMCLTNLISTCVENFGYCWFGNEMISQSEQLAFSAYSSGWVGGGSRFQSSLMLLTMRAQRPLCLTVGSLYT

>CkiaOR58

MSVDEEQPGDELLRLVVSALRWGGLWRPPGSGLWYRLYQTAVLSVGPLLVAGVLLSAAHVARQAEVGGSALAVHVYYCTVLLSNTAKTAVFAACRNSLSHALAMLAHCPSSERSKEMGVISRRKASLMFALPQVMVVLAVATHSVVPLLGTEDNGPSICSSLQAEDGLSTAGGCFSGRFPLELWYPTAAHSAPLYHMVYVLQLLAIYYTCHTAINVDLFFFAVTNHASSQLEELNNALCSMGSRRPAHHIPMNRRRGSAESDHSLNGSRGDGPSWDTSEKNLVQVEHQKTMYQGLIRLMSLHQMITSAVTELEPVISYALFGPMLTNILNMCLHMLVLTTEHDNMGTNSKAFVGMVFNLIQNGLYCSFGETLTHQSDRLFISMYSSGWEDGYPKFEQAVAILLFQTSKPLQMKVAKLYTLSSSTFLQLLNNSYGLFNLLYQVKNPE

>CkiaOR59

MRVTTVHKETAVASEAGRDLMGPGEAVLRLLKVWPPPGPEARGRLRGLPFPLLALQAGMLAVLASTAAYMHQGGATEGDAQEHHVTVSIFVAGTIIGMMVKIWSFMGQRGHMQLLLSLLLEMRRRYQRDDTGARPRAQAQGVTLFYILQVNAVAAAVAWSAQPLVSGGGRRLPLPAWLPFDATASPYYEAVYAGQSLSLLLVPQISLCLNICYFALMLHLAAELAVLCDNVAAVGWRRPSKQPHHQPQDREFAAQSENRLLEDNVRHHQLIIRAVSELQQMTSTSVYIHLFVNMINVCSHIFVISVVLLETGDMAVVVSMASSLAVFLSGIALYCIIGHTIIDQSERLPLAVYSSGWTGADMSFRSTVSILMVRAAQPLSITVGKMRVLSKPTFVQVLNGSYTLFNFLYRTQSDKERKG

>CkiaOR60

MRGHKSAGQEHAEMQMALEAGGQLCPCFAGLHSLGLWRPQTAGGGGAARWLRATLLAAAHLVLPVTLAINFLSCRFDSLDLIATNIFLFFGMGGVSTKALLFVLDSNRFDHLLVRLQQSRMHFADNSGASERCRKFATWLYHTQHVVVQLVVVLWVAQPTATALLNGSAPADGELPMPLCVPAHHVHLSPYFELFYAMQALCLFVAVEGMVNLDASYLTLMLNIAAELEVLNNNLVSINYTKLLKNEKCSHSSDCYPLSPSNPAVEEDMYMQLVENMKHHQHIMCRASELESVMSGPTFVHLFYSLVCISLSIVSVTVLLQTDGNMQKILKTIFVIAVFVSQLGFFCMLGNNIIEQSEQLMMSAYSSRWPDADPRFKKTLLVFVLRALHPLH?

>CkiaOR61

MEQLVWPVSPVSSGLRLLGVWVAPPGRRRRHHAVLCWVLVSHAFLLAVGAASLVMDTPEDLPQLSFTAYTTLTTFGLVAKLLSFSLDGARLTRLLQLLLECRGRFPDIGGRRGQHHLMAVSLHRFLQVSYRVNSVMWMFAPIVSAVIAARSNGDAPVKSTYVLPLWLPFDTQSSPTYEAVYVVQLATGWTLSETTVLLDGALLALMLHAAGELCVLNDSLCSNTMNHDPEVPALAADPGMPLGYPKDDTATSSDLKQDHMFRQLVNNVQHHQLIITYTDLLQKVVSRAMSVLLACNTVSICFHIIATVALLQEDIELVGMTKMVVGSTLYAYQTAILCLLGQSITTQVSQPYTKHGVRVRLPPLPPMQCVNQGISICVTPQSERAAVGVRLQQRVVGGRQPLPEAAAGVLRALLQGAVHQGVRPLLALQGDTTAGAESCLFSVQFHVPGSRDE

>CkiaOR62

VTYSINTSYWLLAPIVRNMIAVMSKKTYNSDIPIPMWLPFDIHWSPAFEILYTMELAFGWAISETTVLVDGSLMAMMLQVAAELAVLNDHLADGTDPTLKEALNEIIATVGTVKDLDVQMPPKSSLVVGVSSDFQHSSVRSDGEMYQQLVGHIQHHQTIMSCVSLLQKILSRATSVLLFCNTISICFQVIATAVLLQEDGEIVETLKMLMGSTLYAYQVALFCLLGQSIINQSERLMRSAFSGDWPDGDVRCWRLVNMLCMNTKTALSLKICGIYTLSSMTLLQMLNVSYSLLNFMYQTKTEQNNAQE

>CkiaOR63

MEEASDLVGATSVALQLMGLWGWGAGGVAAHVAVAAPTLFVMGSAAVLSGAKLCADPPAVYEELIAVIFILVASVSWTFKASAFVGQRERLQALAAHLVGAGRNYGGVGGGTRAQYRALARRVFIYTQAITAVPIAMWALEPLLSGGQNTPLPAWLPLDLHETPAYELLCTFQAVAVTLSVEASVCLDMFFIVLMIAVAAELHMLNDNLETMRLHPVDSTPLKPADDSAARLLSNASSLTEKELPSMQQFEFCRDNHKSTNAFHGTADARELMYYSLVKNMQHHQLILKCVKELETAMTYSIFVLLFLNMVTICTLIISTTVLLQSDSDPTSLYKMVSSLPIVMFQTGLFCIFGQMIIDQSERLPAAAFSSGWLEGDVRLRSALLLLMRRAASPLCIIVGSMYPLSSHTYLQLLNGSYTIFNMMYQVRGSSD

>CkiaOR64

MSISLFVFRPTLEAVVCGMETADQLLGPSASALRLLGVWPGAASTSAAVGTCWVCGCYLALVAFAASQLLAEPWRPLEELAIAALVFVTSASFTFKGAMFLKYRPRLLKLATLLCGGGLRSAVGAETRRRYSERGRKVFIYLQAILSVPTLLWALQPLLVPPGGSAAIVWNATAAVRMRSTPLPMWLPPVLQRSPTYELVYGLQVASMLVVLQTSVFTGIFFLVLMLSIAAELHVLNDNIASNSTLLSNSSSQQLSSFTMEMHHASEGGPTNDSAVANFDSKLFLQQMYSDLVKNIRHHQLIMKCVKELEAMMNLPIFVLLFLHMVNICAQIFVTSLLLQKDNDSTTMFKVLFTLPMYLYETGLYCVFGQMIIDQSERLSNSAYSGAWLQGDVTFRSAMLLLVSRAARPLTLTVGKTYTLSSHT

>CkiaOR65

LSIAAELRVLNNNMMAMGKCSHCEAGARYETDEVVSSDRVASSLLIDHRKPRVPLAAANKSSPGPVAVHVHKVGYQYMYHQLLKNIRHHQVILVSVEELQKAMTHSIFVLLFLNMLNICVVIFAGTTLLQKQADQVAMYKMLFSVPIYMYETGFFCVVGQTIIDQGERLSMSAFASAWLDSPSKLHRLLLVFMLRCTSPPTITVGKTYTLSKRTFLRMLNGSYTMFNMLYQFQSKS

>CkiaOR66

PARSPGYEMVYAVQALCGSVAVLVSILLDTSFYKLILMVTAELQVLNDNMALVGSAVVSANPNGSSAAGAAGQKGEDVAVPPIKPSAAPYTEDDDSARKLLYLQLVENVRHHQAIMKCFQLLESALNYSISILLLTNMLTVCFSIFFASVMLQSDGGLSSAMKMTSSIPNLLIETGMFCMFGQMVVDQSESLPRSAYSCSWVDADARLKRALLMFLMSTSQPLEFTVGKLIKLSKETFLKILNSSYTLISLLYQFQESNDW

>CkiaOR67

MQVSGPRHLSMEQHSRDGSSVGQSEDVGRLLGLGAWGLRALGIWAGPEPATTRWGRAGPTLLLTALFAASATAQLGRERLDDLEHLGEQIFVSTTLTVTSMSIMYFMAYRLRMQRLANLLLEARRRFPEQGAAIRKSFQSRATSMCIGFQMTATVPLAMWVLNPVLAAAMTSPPPRGLNASASASAAAAVRQTPLSLWLPVDDQRSPGYELVFALEGFLIVFTGMASLFLDMFFIVHIIHITAELNVLNDNLAAIRVGAVSGGSSSISNTGSMRRSFNSPDKFAKLNTSTSVISRIDSDEYSHLVEAIRHHQTVMSYVQELENAMSQPLYMLLSTNMVNMCLHMFTFMTLLQSNIEPASMMKFMFTFPAYLYQTGTYCIFGQAMIDQSERLVSSAGSCGWPECAPDFRRMLLVLMEQASRPLCISVGKLVTLSSNTFLQLLKDTYTMFNMLFQLQGDKW

>CkiaOR68

MSVTAAQSAALLSPCSVALSWLGIWSPPGGRRWRWGLGVLGAVLIAGLDITISSLALLQMLIASPEDPAEFREVFFICSCGLSWSVKVVAFLLQWERLQSMVLCLLDAMTRFPDHGAGVRESYTKMAYTVWRLWQAFPAVTVLLWMSDPLLQTLMAPPAENATSPLIFWLPVEVQGSPAYEITYALEAFFMGTVSETSILMDMFLIILLVYAAGEMAVLNENVAGMGHAKQRDPAKAQPVESSGEKLSTYKASYAPSRSSPAPDVTGGSKAELLAMDSDDARSELYSALASNIKHHQVIMTYINDLEVVLSTSIYVLLLTNALNVCLHSFGLVALFQEGATSSTVVKEIISFVSFLAQTGLFCFFGQLMIDQADQLQFSAFSCDWPNADEAFRRSLRIFMVSATCPLKVTVGKLVELSSNTFLQAVNASYTMFNMLFNLQTSDEN

>CkiaOR69

MGTAAQSDALLGPSSVALFWLGLWGPPGGRSPGLRLLGAAFITAANVVIFVCSGVQMLVDTPADPALFREAFFLCTCSCSWIFKVVSFMLHRKRLQRMVLSLLDASTRFPDSRGGVRESYSSTAYTVWKLWMGVTVTVVLWMGDPLIQTLTSPPAENSTWPLIFWVPMDAQHSPAYEMTYALQALCMGAVGTTSILMTMFFITLILHAASEMTVLNENIARMSLANSRDREKEPEGDTSVQRVDSTTWEVSSLQATYVLSEAMPAPQVSSEYHGSLLVLDTDGKCCDLYSALVKNMRHHQHIMTYMKDLQVVASTSLYLMLLANALNVCLHSFGFVALFQEGATRSTVMKEVLSFPSFLGQTALYCFFGQVVIDQAERLQFSAFSCDWPHADEEFRRTLRIFMLQTARPLNVKVGKLVTLSSNTFLQALNASYTMFNMLFNVEKSN

>CkiaOR70

MPAARAMKNQVDNDEKMWDTTKEVEAMLGPSAVLMRAIGLWEPPGGEPAGAKVLITAFVLLMMGVVFVGGVVEIGLDPPPLDDILEATFTCACSLTWGISMAMILLRQSRVQRLVVDVLSMSSRFTENAAVLRKKYHSRGLIVCVAWVVFPLLAVPMWFVEPALTKKVVTTSENTTMVVRKTPFIMWMPMDTQGHPNYEMTYAMHLFLIVTIVSANVVVDLFFACLMINMTADIAMLNNNVANMRLHKEDGFTKNSGKGVKMSGTLDVTYNGKQSLTEKSEPHDDNTQVNASYTTNTSAQLYSTLVNNIQHHQLMMSIISDLESIMSESSVIMLAVNSVNICLQALGFVDAFRPGAKSSSVLKKVLTFPAYINQTAHFCWYGQEIIDQSERLLESAFNCGWSNADKRFCSSLRIFMLQTSRPLKLQIGKMFTLSSNLFLQILNTSYTIFNMFINF

>CkiaOR71

MSDQMHSGGEQVWDTTKEVEAMLGPSAGLMRWMGLWQPPGGEPAGAKVLVAVAMDVMFFMIFAGGLVQVLLDPPPMDSMLEVSLTLACSVTWAVSNMTILVRQNRLQQLVLDLLNMSSRFTENGEFFRKKYYSRSLIGSTLLLGIPMFAMPMWLVEPALTKTLVTTSENATMVVRSTPLVMWMPMDTQTHPSYEMAYTFQLLLISIVVNANIVIDIFISCLMITVTADMAVLNHNIANMRMCKEGFTENSEERLKLSSAWEITCKSKKPQMEEGEQYDDSKAVHASYTTDSSAQLYSTLVKNIQHHQFLMSIVNDLESLMSESSVLMLGLNSMNICSQGIGFVDGFRPGANKTTVLKSFLTFPAYVNQTAHFCWYGQDIIDQSERLLESAFSCGWPDADQRFCSTLRIFMLQATRPLKLQIGKMFTLSSNLFLQILNTSYTIFNMFINF

>CkiaOR72

MEAEMKSLVGPSGWALQQVGVWRPVDSGPAGLRLLAAVFVVATDALVSTSSLVQLVVDTPTDPETLRDVFFQSTCSGAWAISAVMFMQKSRRLQRLLVTLLDTSKSYAEEVPGISKSYDRSAAILFFAWQMLPLTAMSLWALEPATAPAETVVVGNATVVLRREPLVLWLPIDTQQSPAYEGIFALQVVGIATVSEVSVLLDIFFVTLMIHVTAEIAVLNANVTSMRLSSLSGKARQPYGSADATAVGQQSYKGGGQLADGDGPTGWSAAGTGHLSDKTLFYSQDMEDTQSRLYASLKTNIQHHQAIMLCVNELEAGMSDSTYVTLAVNALTICLHAFGFVELFQGGGKGPAVVKSLLACPIYMGQTALFCLYGQSLIDHSERLLDSAFSCGWPAGDSRFCSALIIFMQQASQPLKISVGKMVTLSSNSFLQIMNVSYTMFNMLLNTQW

>CkiaOR73

MEGPGEEERRVLGPVGGLVGLASGQRRSLSARLAECGRVAAAALNILANASLVTTTAVQLCADTPKETESASVTAFIFSVSTINFVKAVSLLRHRWRLRQLAGRLVAISGAFADHAGTRRSYARKAGRLAITWVVTAEMNVAFWCLDPLISELLGGAVQERQLPLPLWLPFNQSQPSNYALLFTLESTMLMSGVQIAIIVDALFVTLIMNVTAEMHVLNSNIRSMSKAATYDGGLLSMAPASASVGTTENSSKISSNSSSMSEVTAEPLSNESSADDEMYGLLVKNIQHHQLIIMCVSELEKAVSMGTFALLSMNILNLCSHIFSLVVMLEGENSASAMTKMFVAVPVFMLQNGLYCLTGQAIIDESDRLINSAFGCGWPDADERFKSSLRVLMARASQPLRISVGKLISLSSATFQELLKGSYQLFNVVYQVHTN

>CkiaOR74

MLPSADDEKLLLGYTSFLLGKSVNWRNSSFSTLLIDYTTTAALLSTAAANLLIMVCCALKLYLDPPEETEKASQVALLMTVSMANVMKGISMVQQRVQLQQLVAWLLVMSSAICDGTGVRNVYTRSATVLSNVWMVMSVILGMVWAVDPLLNQPAQLNGTSPDPALPLPMWLPFDVSAPVTYGVMFPLEAAVCGWTMFFVMSVDMLYVTFILNFAAELHVLNHNVQMTGNSDDFTAHPNSKMGVSNRVVTSRYEGNGDDSTVNQNFSSAQFITDNPLMPAYTQQHFKVEMSEGNDTYRSLVKNIQHHQLMISCVNEFEKATGLPVLLVVSINVLNMCSNIISLAVLVEEDPNVSAVGKSLLFTISFASQTALYCLPGQMILDQSDRLAHSAFSCHWPDTDGRFKSSLLIFMVCAGSPLRLSVGKLVTISSETFLELLKLSYQLFNLVYQLQSS

>CkiaOR75

MEESGDVIGPSMCVLRLMGLWVPASASGGVCSGAAQYLPGALSCAAIGLLSLSCASKLFLDTPKELTELTVCAYLFVIVTANFVKAFCLLLQQDTLHELVTLLAEAKKKNVIDVQHNEEIRSQYGRTSVSLYRYLQVMIVVSSIAWLFVTVLIRVISAGSTNIEWPTPIPIWLPLDMQLSPTYELIYLAQVSCAVMTAGTMLGVDTLFFHLTLMIVAELQVLNGNVSVVGSTTSRNKEEVVFGISSDCVTSSLNDGGVSLANSREMVTEEENYLDLIEIMQHHQIIIKMVSLLQTIMDYSVSVLLLTNVLDMCFLMFTMSELLHHEKGLHAVLQTMLSLPCLLCESGAFCMFGQMMIDQSENLVQAAFSCEWLDAGSRFSEALYTLMLMATHPLQIKLGGATKLSSSTFLQALHGSYSL

>CkiaOR76

MEATDEEVYSVVPCAARIGLLGYWRRGGAAEGLGRYLRGWVSCSLITFITLSAAEKLLIDPPSDLAELTMTAFELTVPLTVVSKGVCFMLQRKTIHRLIDLLVEMSRQYAESDNGPGSRSACYLYVLAVQRALLVMGLMVIGGWVAAPVLPHVLSLSSQNLSSAPWQTPLPLWLPVDTQRSPLYECLYLLQTCCVLASLLSAAALDAFNCNMTLVIAAELQVLNDNIASPSGKEPVVDKGELKSLAVGDHTRYNGGATADAHPSNTEMSSSRKQTTHSLVQNIRHHQQILRCISLLETSMKYSIPFQLLNSVTSMCFVLFVCSASLQSGTGLNSALKTFLCMPYLIFETGIYCMYAQEILDQSEDLLQSAVTYEYIEAGPNCCSTLLMFMLMVSSPREVKVSKIVPLSKPTFLQILNGSYTLINLLYHFSVPDE

>CkiaOR77

MEGSKQSVAALEDAEDLVCPNTALLRRLGMWNGLDAAGGRIKSWVVWAMAAFMSGSGAWKLCFDTPPELADVADYGYFVCHLSAVAMKVAFFILQSGTIQDLVKQLAYTSKTYGSTEANHRVRDVYRRRATKVYIVLQVLVVAMVSLWVSAPLFQDISGPHPIWHPEHSPAFEMVFVMQLVFGTTATELAMLLDTSYYKLMLMVTAELQILNDNMAVLGSADESADLKGSSATDVPGKESDVAIPPTKQTPVPDTEDNDLTSRLLYFQLVENLRHHQAIMKCFNLLQSMLMYSVTIILASNVFTVCFSIFIASVLFQSDGGMKSIKTLSGIPSVLGETGMLCMFGQMIVNQSESLRVSAFSCGWPDADARFKRTLLMFTMSTSQPLQITVGKLISLTSPTFLQILNSSYTLINLLYQFQGPKNW

>CkiaOR78

LKLSIEPAHDLLDLTWTVFLVFAGALVFVKVMVTTAWMIVVFLPLLNHQVPATEQELTAKEHAPLPIWLPFEVHSSPFYEFTYAAQMLWLAFVAETSICVDCIFVNLMLMITAELHILNSTLSTLQEDSIVNKVPDVATNKTTEDLLSSSHSSSIQYSTQLYEKLISIEGTDNNVSDMSDTFYANSQKKMKQEMYQQLLKNVQHHQTVMKCTASAQKAMNFSVFVLLSTNILEMCSSMFGTAELLKNDMPSAAMKTLSMIPLILSQSGMYCFFGQMISDESEKLVQSAYNCDWYEGDIHFSHMLLFLMLSATGPLKITVGKTMSLSSQTFLQVLNGAYALLNMAYHVNK

>CkiaOR79

ITAFNVSVDRSTQSILEDMYDQLVGNMRHHQMIIECVEVLQTAMNYSIFALLFFNMTSMCLNIFVTAILLQSDADLVTAMKAVFTTPVFLCESAMYCIFGQMIMDQSEQLPLSAFSCGWTAADTSLQKALLVFIMSSSQPLSIKVGKTYQLSKDTFVLNGSYALFNMLYTFHGKK

>CkiaOR80

MIDEVSELLGPVASALRLLGLQAKPSASSTSWSLTWKAALLLAVNVSVPAMAASKLWVAPAAALEELAVEVFVCVTGVAMTVKVAAFLWQQERTQRLAQLLAGCRRNFKCGRGGSGDTRGRYGRHIRRIVVLMQVMVVVPTSLWLLDPLVGGGSWARRLPMPMWVPPSLRCSPGYQILYAFQALAMITVVEASLYLDVCFVLLMLSVSAELHVLNDAVASIKAPGALRTAPYSAWNSETNLTNEGYLHSQTGAVACNSHQSTGERSETTTIMTDDANGTVTDMYRALAKSMKHHQAMIRCVEELESAMSQSMFVLLFLNMMNICVHIFVTSVLLQKEVERTTMSKMLCTLPIYMYETGLYCVFGQTIIDQSEQLTASAFSGDWPEGDARMRKALLVLMLRSSSPLKLTVGKLYVLSSHTFLQMLNGSYTLFNMLYQVQKNKW

>CkiaOR81

MATTGATAVALLGRNATMLRLLGLYQQKGGGSKVAPALLSAFSLVFLLYHPVFAAMKLYMEPPEDLVEFALCSFSFMISDGVFMKTAIFIADSGMLHQMLQVLSDSSSLYGGEETSKKIRNRYEDLAERVLLYMQVSTMLASVGWLAAPLVFRALLLASGESEVPRKLPLPVWLPVDVQASPTYEILYVMEAYCVTLTGLVTLCMDVLFMRLMLMVTAELQVLNNNVATMAKSREKISNELHPQGTETQDFYSGDKALQMANTSYEEGLDDQLYQQLVTNVRHHLIILSTVDLLEAAMSKSIIILLFINMGALCSNLLVVGVLLQAGEGVTSPLTLTAMIPFLLYQTGMFCVFGQMVTDQSEMLMTSAFSCGWNESDSRFRRSLLIFMAMVNRPLEITVGKTCKLSSEMFLQVCKEEYF

>CkiaOR82

MGHPSADSDSLEGPGVILRRVMGLWRPRGRVARIFNVLLAGITLVSMAFLVVCVALKLYADPPEELEQIALCGLVASLCVGFFFKASLFMALGGTLRQTVRLLEDTSVEFFSGDNNKLTRRRYQKLSSNIYYYGQMVAVPAAIAWVTCPLLSRILAKTDQDHHEVQRQFPVPVWFPLDVYASPMFEYMYVVQSFCVLVVAECCISTDIFFVHTMLMVAAELEVLNSNLSSLGHTNLQTKKVKGEESIFSYKTYDSRLTLLNGVQPLGEHASTEDTVHEWLHEQLVKSVRHHQAMLSVVSLLQSAMDVSIFILLFVNMA

>CkiaOR83

MADSGSLAGPGGALRRLMGLWQPEGRVGRLLNGVLAAMTLGCVSFMALCVTLKLYGDTPEEVEQITLCALVASICVGFLCKVTLFVTQGGTLRQAVRLLEETSARFCNGDHNKLTRRRYLDHSNNVYYYCQMVAVPAAIGWVVCPLLSRVLTKTDEGQPQPQWQLPLPTWFPVDMYASPTYETLYVVHSFCVLVAVQSCLSMDIFFVHMMLMVAAELEVLNCNLSAMQHINFQTTSTEEEGFISSYKSNGSRLALLSSGQSLADQTLTEGASQKELNQQLLKNVLHHQAMLSSVSLLQSA

>CkiaOR84

MGQPANEPVSVLGPEASVLRLLGLWRPQEGAGQLAPLLLAAVSLASVAFMPVGVVLSLCGDFPQQTEETAHCSYMCIVCFGCIVKAVLFIVEGGTVRELVHLLHAMSSEYGTDEGSERIRSSYQSTVDRMYRYFQVMALVPTLFWICSPLVFAALANTPQDPQTNHHQLPLPFWLPGGVSSTPTYQLLYAMQALSLTVTVVSAVCLDVFFIRLMMMVAAELQVLNEKISAIESFQASGSMYGREAQEYALVTSGDRARDSTKKSAENFSDDEMFSQLLNNILHHQVMLSCIWLLQTAMNVSIFILLFVNMANLCFNIFVTAGLLQDGSNVTKAVKAFSTVPGLILQTAMYCLFGQITTDQSEKLLYSAFSCGWDDCNTRFKRNLLIFMLMVGSPVEITVGKTYKLSKEMLLQVLNGTYVLLNMLFHVHSDD

>CkiaOR85

MNQAETEYPSLLGPGVTIRQVMGLWSPQGRRGHFQSAACATVTLVSLAFLPTFCALKLIMDTPPELEETTLCCFVIFICSGFLIKAALFIYHGDILKKLLQILSDNSSIYSNDENSEDIRYTYLKLSRRVYLYMQVAVLPGVAAWVLAPMLARVFLSSEDDATEASQQFPVPLWFPGNMYLTPTYEMLYGAQSFCLLVAAQSTVVMDVFFIHMMLMVAAELQVLNENFSLMQKLNVKAQEYEEEEWSLSIKSNNDELTFSSNYHPTSATHSWSEENSDEKMCLQLVKNIQHHQHILSSVFLLQSVMNVSIFILLFVNMADLCSCMFVTAVLLQSDGNVTKAVKPLLVIPPLLYETGMYCIFGQILTDQSEKLMSSVISSGWIDCDARFKRDLLIFLMAASSPLEITVGKMSKLSKQMLVQVLNGSYGLLNLLYHFHSTQ

>CkiaOR86

MPPSTTCCCTLPVLTQFEVSDENRRQSDGFPLLGPGATLSRVMGLWRPEGGGGGGWRGGGASAAVTLAAIAFPVACSVLKLCADPPSPLEEVTLCGFVACMDFGFLIKAALFIKQRGTMRQLVRLLWETSEKYTNGQNNESARARYQKLVRRMYVYMQAVVVPALACWVWSPLLSRAVLTSRQNATDASSQLPVHVWLPADVNRSPTYEALFAAQSFSLMVLSQATVCMDVFFVHLMLMVAAELEVLNNNVSTMQRGNVQRGSSTYVETTVIQDENNGGPTFTSSDHCLGVAVTSSQRDQNEHMYVELVKNVRHHQAVLSSVNLLQKAMNPSIFILLFINMANLCGTVFVAAVLLQSDGNMTKALKELMLMPCVLYETGMYCMCGHMMINQSEKLVMSAFSCGWTDCDRRFKRSLLIFMMAAMRPLEITVGKMCKLSKQMLLQVLNGSYALLNMLYHFHRTLW

>CkiaOR87

MDGDADVDKVVGPVLVLMRLLGLWPQAGRDRTGCLTGAVRRTLAFVPMALMVVGSALKLCLDTPEQFEDITLCAFMTNVVAAMLLKAVMLVVQGGRLRQLSRLLAEAWAEFPEAAPGGSTGTRGRYEALADRMSLCFQVGGLAPLLCWLSAPLVPRLTAAPGQSRRRQRQLPMPAWMPTDVYASPAYELVYALQALGCAGACAATICADSFFVRLLLLIAAELKVLNENISSMRSTDPVRGDSGYTCSCRETAVILPSACEDCRNTSIPPSEKTADELHRQLVNIIRHHQMIISMVSLLQEAMDVSMFVLLFANMANLCSSLFTAAMLLQEGGSMSKILKGLSPLPVVLYQTSLFCIFGHIVTDQSEELVNAAAGCQWAGCDVRFKRSLLIFMTAALSPLDITVGKVCPLSSEMLLQVLNGTYALMNMFYYYHHKTK

>CkiaOR88

LQLLAEARQRFPDAEGRRASHHRIALKLHRFLQVSYSLNSAMWTVAPIVAGAMASPPAQRGFMMPLWLPLDTQASPAYELLLGVQVPCCWLCSETSVLLDCAMLALMLQAAAELAVLNDSLCGVGANSGDTAGGKGALQPQADVLHRLVENMNHHQIIITYMHLLEMMLSRGMSVLLICNTISMCFHIVATVALLQEDIEPVGMTKMVLGSTLYAYQTAILCLLGQSITTQSERLPVSAFSCDWPSADNRFRRLLMVFCLRSSQALVIRVCGLYSLSSETLLQVLKAAYTLFNFVYQTVGEEEPLNW

>CkiaOR89

MERMQSAVEGRDGGGQPRALCGHVVYLAVAGLWPRARGALYVWHAALVQACAACYVALGLASVHSARGDVDAISHTLMHTLEVVSGMVKAGLFFGKRRAFYRLVHDLHALVSADWERPELARARRRARRMTVSLSVYIYALILLWLPAPLLVAGERSLLPVVQIHGVDWSRRPGAYAALYTLQCVVLLTQVPVVIGLDCFFVAAMLHAGALLQLLGRRVSGLWLSGDAEAPVDGGYDTQQALYADLCGCIRTHQKITKFLRDLESAMSAMVLVQFSSNMVGLCMTLYQQTQIKTCLEMKSASLQIKDAYFQMKDACLQMKGMCSQIKDALTALQYALILPFYTGQLYFYCWTADVITEQGEALSSAAYSSGWVGSSAGFQSALSVVMARAHRPLVMTAGRLYPITTATFVALMKASYSYYTLLSQLDADW

>CkiaOR90

MFESEVDVSAKEEHVAGEEDPDKELELRVQQEFGMYCRLMSLGGVWPTGGAEPAWSKAVRWLLLANSLLTTAALASEAALAAARTSGLTDFACRCAFVVALGSNNVIMLLMARHHGRLRRLMAWAVTIRPRASCRRLQRNASAGATAMAAQVLLWLAGCFCMGPHALLLDISYPVDGLVAGRASQVLLLLDQAAVAFNHLCSVASFSTMFAHFVYMACQHLQRSMDDLTADNCDIAAVVRHHQQTLSYIKGMEDVYCIIMLWVFLPMMAVMCLIMFVVLKMTSVDIEFLEMLAFFLIYFITNGVISMCGSMLTSKAERVVLAAYSSAWPERGRGFSGAVRVVMVRFLQPAQLTVAKFVPLSINTFSKLVQESFSYLMVMLSLVNEKDSEPQPAVVLEAASNHSAHY

>CkiaORCO

MQKPHGLVADLWPLIRMVQYSGHWMLEYSGGKALRAIYSSVVSVLVVTQFALMAVNLIQRSGDVNELAANTITVLFFLHPVTKFGYFAVRSKAFYRTLATWNQSNSHPLFAESQARFHQLSVVRMRRLVMYVVAVTALSVVSWTSITFMGDSTREVTDPDNANETIIEEVPSLMISTWYPFDASSGMGYMLGFVYQLYWLTATLMHSNLMDVMFCCWLIYACEQLVHLKEIMKPLMELSATLDTVVPHTSELFRAASTLPTSEPLYGMGPDMSNGVTDGMTIRGIYSSQRDFSGFNRRSAALSTVREADAGGAVTSAGGIGPNGLSKRQEMLVRSAIKYWVERHKHVVSFVGNIGDAYGAALLLHMLTTTVTLTLLAYQATKIDSVDVYAASVLGYLFYTLGQVFLFCVFGNSLIEESSSVMEAAYSCHWYDGSEEAKTFVQIVCQQCQKSLMISGAKFFTVSLDLFASVLGAVVTYFMVLVQLK

>LmigOR1

MSNGNEGIYFGTIINLMHCFKIWSPDDSKRTISFSVYILVPAYVFFFALSCIEIYHNWGDMLSTTDAVNTFVIYLATSHKYFRLIYHEKDLKKLMKMVENNFSVPVWQNDALRNSIVKSYVQEVKKLTILWTTLCFTTLCGFMILPLVDGLFHYYTTNVTTEIEWKLPYRTWTPFNDYGAIVTVPLYVYHMFMGFVLIAEIPAFDTIYFSLINHSCAQLKIIQNSLINIVSISAQNVLSNEKNGVTFDTMLDEHYTAEENKDQSSLENKMNNGLNSYTPSGSYDSMLNLLHDSELDKKIRKNVGELVNHHEKILEFIDGVEAIVNAVFLTQFLCSATLFCLTGFQLTVILKEQQLARFLNMMELLGAAIFEMGMFCYYANRVMDEGINVGKAAYDSQWYYASKDYGNSVSIIMARCTRPPKITFGKFADLTMENFASVLQISYSYFTLLTRINE

>LmigOR2

MLRQVYEALRDADEPSRYLEFNVLMVRFMGVLVSRSLTGALLTWSLFVLLATHCLAGVFDLVNNSGDIADITANLPVTTIIFSSTYRLFFFTLHRDRYQAIVDTVGARFVASSDSIDMAPWLRRSRIISILYFTYGFFVASTWQLHPLISAQLTAAQMAKESNGTFEGLPRELWEFPTRAEYPFDARQPYVYTVVFILQGAAIFVSGCMILVLDMMFITLTSLICGQFEILKDKLRNMRKIATNQRQNEDIGLVGKALQKQALERRKRILDSNIEITKQDENFNEDLITRKINLLLGECVEHHNMLLSLISEIEFMHWSAYLVNFCVLLIIFSFSAFEVTSGTPTSPAKVVNLAEYLLVSVLQMFLLCDCGDKLVDQELSVSQAAYESEWYHCSESVKRTLQIIVLRSRQPEQITVGKIAGLNLDTFSDMLSRSFSYFTVLRQIRDDS

>LmigOR3

MEEAVEAARGRAPRLLQTLRGDVAAPAHYLAFNVWLCRLVGLLPRPGQPLWATVQPLVWSCLLLHLMCELVDIALNIADVQQLGKNLPISSLVGGSWYRLSYFTMRRDAYWRLVSKVGESFHRGAPGRMRRWLRRSRGFTLAYFVYGTIVCLFWLGHPLLLQQTTHTMFTSSNSTNRSRMSETAEFPSGAWYPFDVRERRVYGAVYGFQCLALYFAAMLIMVTDIMFITLMLLACGQFEELGDKLRHCWEIATTRALSRAGTTPERELQKVLAHCVRYHDMLLGIVGDIEDLHWTSMLVNFVLQLIILSFLAFEATASADLTNPLKGTNLLMYLVMAIFQLFLLCSCGDRLMEAEEMVARAAYESQWFDAPQGAKRSLSIIVMRARLPQRVTVGKVVGLNLVTFSETLSRAFSYFTVLRQIRTSN

>LmigOR4

CEMAVLGADWETRMLTHGAPCLDLTVYLQPILKPLAATGMWSSSIYSNSVHKRAARAAGLSLLALFLAELCAELVGTPLVVARGGGVDRLIHHLSVLGIHLDSFWKWLFMLTQRRRLTCLLRLLQRCFQLGVLASPTHHLPLRNTYIQKPQQSLDPQPQAMPAQLSEVLGKTRMRSWLVTVVWTVSCVLGASHWFFVPLLKGDTTLRFDALYPFDSQQPTTKQTVYWLQYVCSIYSLLLLCFFDCLLVWLQQLLCVQLRCLATNLRELSPNDEHRRKLAICVSHHEHILRAMRELNAFVAPLLFLQCFKNMIVLCVVAFLASVAGVNDLLELSSLVLYFMAACQQLFFYCWCCEELTHLGMEVCDAAYDSGWENWDVSSQKSIIIIMWRAQKPFFFRGGWFYTLNVATFVDLIRLSFSYYTVLRSMREG

>LmigOR5

MLGQGQAGPGGRGQHLPRVDLLAHLSPILRCLAVFGMWPPSVYTSVSQKVAALTTGAATLSLFLMQLVAEVMALAASPVSGAAELYRFIYNFSVVDLHLQGMGKWAVMVARRRRYTALVHRLQHCVRLSGLADADYKNQQAAVSRLRQQLDDCRRWGVRANAVWLSVCVYGVTHWCLVPLLIGDNSLPFDALYLFSTDQPPLRQVAHCIQYVAGLQNVLLSVFFDLFVFWLHLLLCAQLRYLAGNLRRLRHLTDAAQYRRMLAACVAHHSHLLSTMQELNSCAGPSFFLQCFENTIRMCMIAFMATTTVADQMQVWSSAQFFLAAVAQLFLYCWCGQQLSHLAESISEAVYDSGWEDHDVSTQKTVAFIMWRAQKILVFKGGWFYTLTTETFVELIRLSFSYYTVLRNINDT

>LmigOR6

MDVSFAAVSVMRAGLPAEESGGNRRARRVTQWSLALWLGTTCSWMLAALLRLDLPFFAWFPFDTTHHYAEAFIYQLVTANLVVVIISGLDCFCLELMMHLSERLQTLNKLFRSFATDNARQQPQLRMQPSTPASHRGKSPYGIITKKSFMKWVVAPLDVHRKPAKAINSLYSRHDSPNVGNANGSFKHCIQYHWELIELKKETEKFCGVVLFFQILASMFIICFVTFQATVNTMDAGSLTKCVMYLSVALLQLGLFCNEGTNIVTQSEELMLAVYSSEWPDCDAALKQSVIVTMMRLQYPLQIRAASYCTLSFETFSKILHTSYTFFTLLRQVSETQ

>LmigOR7

MRELEEEWERQRLRGLRLVVRALQLMGAWPPGADAGAGGGGGGGGVRRRLYRAYVAAATALMASYPACALCVLAGTRADIQVTAVLVGVTSSYVGAIFKMFTLCYKRSQVSGLVRAVQREFPRSPLLAGRAQRAVAASAGQHGARLTALFVSCFSSVFNWSLGPIALHLLGGGGGALALCLPWFPDHQPAGAAFRLVYGYQVATLVLVALWVGALDAFLLVLLVYAAGQLRVLNCTLLQMGARSDDGTEGQREKFASNASAMLRECAKHHLEVCRFVQDVEQVAAPALTLQLLVSTFLLCMSAFTATQIPVGSPLLARVVVYMLTGASELLIFCKYCDDVISESERVQQALYGSGWSAQGGAFSRGVLIMLARAQRPLCLRAAHVHPVSLQTFTKVLNASYTVFTLMRQIKD

>LmigOR8

MDYQLGDHGCGYPLRCITDRSACDGVRSDEPGCTNGKTSFFSDESRFCLRHHDGGIRVWRHSGKRTLQACIRHRHTCVSPGVMVWVAIGYTSRSPLIRIDGTKRQEELCLATDSSYIVDNGIETLAVLWGFEKFRYFLYGCKTREYTAHSLDLSPTENVLSMVVEQFARHNPPVTTLDELWYRVEAAWASVPVHAIKVLFDSMPRRIKAVITARGAVLTRLCPVVPQYPIIALYPWPVQSGPAYALTFSLQVLCGGLFTMTHLACDTFLLSLLIYICSQIDVLCASLRQLGRRLLRIVSLVSYRYVGVLQQVLSPVALAQFMCSMVIICLSGFGIAISNDFGSLCRYSVYFTGAAIQLLLFCWYGEVLITKSEHVSEAAMACGWPAVRRGRFQSSALLLMVRAQRPLALTGSKFYVVSLKTFVQVTNAIITYFKIAPTLGK

>LmigOR9

KLSLSTLRPRRYRRLMDHVTRRLAAHLRHDRAGRLRLRRDSRAAHAFTMGFVVCGHVTVASWSLLPLLLKPADKLRLPLVAWTPFDSSHGTGFLVTYIYQFTCTLFMAWTSGATDLICVNVVMQLCSHLDILCSHLERVGRRCCREGDGGRCAHGHLDDGDQRPAPGRDDLCGQLRGCIRYHQDIIRCRVAREMDSMLWTIVLSQCLCGMGVLCLLLFQTAMYTLTIETIAKYLSYMASILLQIFCYCWFGDNLSSKSTAVARTAYNCDWTRGSSAFGRSLCILMARAHRPLTVTGGSFYVLSREAFIRILNASYSYFAVLYTMSDE

>LmigOR10

MELAHLLLRLLHWSGALRHPSKPCGCYLLYSAAVVTIIGLFVVTQVAAVVDRQAGSDLDQATLALCVASTMCVGMCKILNILRSEHLFLKLADEVSRQPDGLSAWEAGAWLWSRGRVRRLSAVYLSLAASMAVTWPAAPLAVGGGALPFVARFPFDVAAPAGYAAAFAFQVLVVAVVGVVVPCTDLFTVSCVEHLNSLLHILVHRVERLNALETGARPQHATADDKAAAVADPLHRDLADCVALHQRIISEAECLNQAIGGVLLLQVVASSAGICFLLFQVAKKTTFHLVETGKLLGYLTFMLSQLLFYCWFGDDMLSKSESVSLASYRCRWPDAPTRFQRSLLMVSMRAHRPLTLRAGKFFVFSRQAFVQVMNVSYSYFTVLRSLSEA

>LmigOR11

MGNKVRVYLNEGNLPREHGSSLGEGGRGLKLKGGMGGEGDLLEDMGTLEGIDVLENSGHKADYMLDGGRGSEGVAGVQRRVAEMDGDGRSSGSVHLRWCCRQVERDFAEFLSPEDVPLLRASGRRLRRVVRAYLWFGAAGCMWWLLYPVACFGLTVQGVPYQMLLPYDVARPAVFAANWLFCTLPTLHVAVMTMASDSYSVSLMVQLRLQLQVLGKNLVALARGAEANHQKKCVKHGPSAEGLVRRSQLEDAIRQNIRHHQTIIRNTELLEKSMGAILLAQCLSIGATVCIQLYQIAVHAQGLVDAGKFGCYLFIMLAQLFVYCWFGDDFITESLKVSTAAYDAVTSLEGSSCSTKRSLVLMMLRAQRPLRITAAGFFPLSRESFVAVVNMSYSFFAILRNFKDEMNS

>LmigOR12

SSGLDLLQRLLHWSGTLRHPRAGRWSSLAFYPLNAAASAAIALFLCSQGAAIWREGARDLDRLTLVLSTFNTIATWLFRLGHIAVHEHQFHYLSFQMERDFKDFLNPRDVPLLQASNQAHRRFVLSYLWFGVVLCMVWTLFPVATFGAGPDGLPFIMALPYDVSPLHAFIPTWIFGAFITVHVTMMTIISDTFNVSLMAQLRFQLVVLNEKIINLTTDIETPKSPLTKEEYITTKSAYESVKHTNIHHRLRQNLLHHQVIIRNTEMLENCIGGILLAQCLSIGAAVSFQLFQVAVSTQSLVQAGKFSCYLTVVLVELFMYCWFGDDLITESENVALAAYTAVTSLQGFPAADRRSLLIAMTRAHRPLRITAGGLFPFCRESFVSIVNMSYSYFAILRNFKDD

>LmigOR13

MWAPAAGLDYLLVSLHWAGVMRHPLAQPGSWSGRSFQLRKAAVAAALRGLPXXXXXXXXXXXXXXXXXXXXXXXXVSACLFRLGHISVHEHQLHYLAAQVERDFGAFLRPRDALLVQKRDRQLRRTLLGYIWFGVAGCAWWIAFPLLRFGFCAAGLPFILELPYEVTSAAAFVPTWLFCCLVTLHTAVLTIVVDSFNVSLMAQLHLQLRLLSRNLLALSDGDQDHDSATFKRVASGNEVSCGYANDIHIAHQLRKIILHHQAIIRNTELLEKCLGAMLLAQSLSIAAAMCFQLFQIALSAGSLAEAGKFGCYLSVMLAQLFVYCWFGDDLITESEKVSLAAYSAVTSLQGLPAPQKRSLLLMMVRGQRPLHITAGGFFPLCRESFVSIVNMSYSFFAILRNFKDE

>LmigOR14

EGLPLQLVLPFDTSRPAGWAAGWLFCAFLTVHCVVMNMMADAFNVSLMAQLRTQLILLTRHLTDLAGDVQSEGPSLPQTPRKVVEQPVKFYTPVYRHYSLEGTSSHYQKTNPSVVDAEVQRSQHSYEPSNSVDYRLQKIILHHQTIIRNVDRLQQCLGGVLLAQSLSLGSAICMLLFQVALSAQGAQETGKICGYLCAMFTELSVYCWFGDQLMSESEKVAFAAYDAVTSLQECPISMKRSLLLMMHRAQRPLCITAAGFYPLSREAFVSILNVSYSFFAILRNFKEE

>LmigOR15

RLLAVAAVSYANGCDVVAVCREGITDFDRFTITLSVFDTGNTWVYRRCHVAWHERDFQKLAQQVRDDFGEFMAPSDVPVLRGLAASLRRFVTAYVLMGAFDTVVWLTHPTRGEGLPLLVLLPFQTQRGLGWLSGWLFCAYITADCLTVNFMVDSLNVCLMEQLRMQLLILRKHIAELGNRSESTKYSLSEKESESAKEVGKPQQGKLAFVNETHKGSQVSSEAANSSDIHSRLRGIILHHQAILRNAEALQKCLGNMLLVQSLSLGSVICILLVQIALSAQGARETGKICGYLFAIFGELWLYCWFGDKLTSEGENLTLAVYDAVTSLQESPTSIKRSLLLLMLRSQKPLCITAAGFFPLSRESFVSILNISYSCFTVLRNFKEE

>LmigOR16

MAASAAAAAAPEESVGSAAQSACDLGYLLTFLHWTGTMRHPRAGRRASRAYAAANAAVTLAFVYFVCSQVVVLFRAGTADLDNFTLTLSLIDTQGTWLLRIRHIAAMEPHFHRLAYQVGRDFGQFASAEDVRVLREGSRRMRTVMLLYLAFGLAECCVWLTAPASETGLPFVLALPYDVTRPAAYVATAVYCCFITLHTIMANFAADAFNASLIVQLRMQLALLNRNIVNVNRIVEQERSSPYKSESADALRPYKSYSTSDVNERLRKNILHHQAIIRNVQLLQSCLGSVLLGQSLSIGISVCFQLYQVAKSAESLQDAGKYSSYLFTMFAELFVYCWFADDLISESENVAQAAYEAVPSLLECPTPVKRSLLILMQRAQRPLTITAAGLFPLSRESFVSIVNVSYSFFAILRNFKED

>LmigOR17

MGHLLTPLHWTGVLRHPRYSHQSPLLFRLYTAAVSSFALCFICSEAAALVHDDTGDMDVIILLISTINTASIWIIRMVHIAVFERDFHKLAVQVGHDFAEFLTWEDIPVMRAKSRRVRRFTLGVVWFGVSACSYFLVSPVSPEGLPFILALPFDATTPLGFAVSWLFCTITCMHAVVMTMALDSFNVSLIAQLRIQLTLLSNKIVSLAREMSQRPIYSPETSSYHELHSRLEKCVRHHQTIIRNVDLLERRLGSILLAQSISIGAVACFQMFQIATSANGVQQVGKFGCYLTTMLTELFVYCWFGDDLITESEKLALAAYEALASLQGCPMPITRSLLLLMQRAQRPLCITAGGFFPLSRESYVAVLNVSYSFFAILRNFKEEEQQPD

>LmigOR18

CSATSVSSCRRATCRVVNADGLPFVLALPYDATRPLAFAATWLFSVYIVFCVHIGTMAADSFNVTVILQLHNQLDLLGRNLRSLKDSVSHVKSASTETLPKRMRHSDNSRDIHSRLRKSVLHHQAIIRNVQLLEECLGGMLLGQSLSIGTSFCLQLFQAATRAKRVQELGKTCSYLVTAFSMLFIYCWFGDDLISESEKLAFSAYDAVTSLQSSPASIKRSLLLLMVRAQRPLQLTAGGFFSLSRESFVAVLNASYSFFAILRNFKEEYD

>LmigOR19

QAYIWFGVGACTFFLFSPASAEGLPYILALPFDASQLVGFAVAWLFCMVVTFHVVVMTMVLDSFNVSIIAQLRMQLALLNRKIVSLAKGVNEKLQQCSDTSEYSDLHSRLEKCVLHHQAIIKNADLLEKCLGTMLLGQSLSIGAAACFQMFQIATSANGLQQTGKFCCYLFAMLAELYVNCWFGDDLITESENLALAAYDAVTSLHGCPISIKRSLLLLMQRAQRPLCITAGGFFPLSRESFVAVLNVSYSFFAILRNFKNEDQ

>LmigOR20

MPPSLPPEAAAASSDLGYLLTLLHWTAVLRHPRFIGTSPFWFRVYTLTLLTIDASFVFSVFIVLFREGTEDLDVFTLTLSVADTNGTWLFRLAHTVACEAAFHKLSQQVGHDFAEFLTWEDIPVMRAKSRRVRRFTLGVVWFGVSACSYFLVSPVSPEGLPFILALPFDATTPLGFAVSWLFCTITCMHAVVMTMALDSFNVSLISQLRVQLMLLNSKLVTLAKEESENSKLSSKTTDYRELHYRLVECIRHHQAIIKNADLLESSLGAMLLGQSISIGASACFQMFQCVTSGNGLQQTGKYGCYLALMLAELFVYCYFGDDLITESENLALAAYDAATRLQGCPLSIQRLLLLLMQRAQRPLRITAGGFFSLSRESFVSVVNVSYSFSAILRNFKE

>LmigOR21

AAAAEASDMGHLLTPLHWTGVLRHPRYSHQSPLLFRLYTAAVSSFALCFICSEAAALVHDDTGDMDVIILLISTINTASIWIIRMVHIAVFERDFHKLAVQVGHDFAEFLTWDDIPLLTSQSRVVRRFSKLYMWGGVGACAYFLVSPVCPEGLPYILALPFDAMQPLGFAVTWVFCSVVTLHAVVMTMVLDSFNVSVIAQLRIQLKLLSTKIVNLSKEILNTDVDSSEANVYQELYYRLEKCIRHHEAIIKNADLLERSLGTMLLAQSISIGASTCFQMFQLATRANGLQQAGKFGCYLFAMLAELFVYCWFGDDLITESENVALAAYEAVTSLQGCPLSMKKSLLLVMHRAQRPLRVTAGGFFPLSRESFVSVVNVSYSFFAILRNFKMEEQ

>LmigOR22

MPPSLPPEAAAASSDLGYLLTLLHWTAVLRHPRFIGTSPFWFRVYTLTLLTIDASFVFSVFIVLFREGTEDLDVFTLTLSVADTNGTWLFRLAHTVACEAAFHKLSQQVGHDFAEFLTWDDIPLLTSQSRVVRRFSKLYMWGGVGACAYFLVSPVCPEGLPYILALPFDAMQPLGFAVTWVFCSVVTLHAVVMTMVLDSFNVSVIAQLRIQLKLLSTKIVNLSKEILNTDMESSEANVYQELYYRLEKCIRHHEAIIKNADLLERSLGTMLLAQSISIGASTCFQMFQLATRANGLQQAGKFGCYLFAMLAELFVYCWFGDDLITESENVALAAYEAVTSLQGCPLSIKRSLLLLMHRAQRPLRITARGFFPLCRESFVSVVNVSYSFFAILRNFKEEEQQPD

>LmigOR23

DLDHLLRPLHWTATLRHPQSAHSSPLLFRLCKLVIITIIFSFFCSEATVLFRVGAGDLDVFTITIGVADTNIIWFCRMVHISVCERAFHKLALQVGQDFAEFLSWEDLPLLRAQSGAVRRFTRLYVWFGVGCVVYYLFSPASDAGLPVILALPYDMHRPLAYALTWLYVSVTTFHVVVMTMVFDSFNVSLMAQLRTQLSLLSRKVVSLAKEMSEKPVHSPETSAYRELHSRLEKCVRHHQAIINNSDLLERSIGPMLLAQCLAIGACACFQMFQVATNTNGLQETGKYGAHLVVMLAELFEYCWFGEGLITESENMALAAYDAVTSLQDCPISIKRSLLLMLQRAQRPLRITAGGFFPLSRESFVSVVNVSYSFFAILRNFKNEEE

>LmigOR24

MAPAAASSSAAAAAAASASDLGHLLRPLHWAAVLRHPHSAAASPLFFRLCTVAMASFAFTSTCSEVTVLFRDGTADLDAFTLTLSVVDTNTIWLFRMAHTVACERAYHKLAHQVRNDFGEFLTLEDLPLLRGQSRVVRRFALAYIWFGVGACAYYLVSPVSAEGLPFILALPFDATRPLSFAATWLFCTVTCLHVVVMTMVLDSLNVSLIAQLRIQLTLLSGKIVGIAKEMSEKPVRSSETSLYSELHYRLEKCIRHHQTIIKNADLLERSLGAMLLAQSVAIGAAVCFQMFQIATSANGLQQTGKFCCYLFAMLAELYVYCWFGDDLITESENVAQAAYDAVTSLQECPVSIKRSLLLLMHRAQRPLRITAGGFFPLSRESFVSVVNVSYSFFAILRNFKDEEE

>LmigOR25

GPALHPGAALRLVHAFGLRRHVALLHGGHRARRGDDHGVGRLQRQPHRSVAHAAHVAQHRKLVTIANDSSMRPLDSLRTTDYRELHCKLRKCVLHHQTIIRNADLLERCLSGILLGQSISIGSVACFQMFQVALILLIYNWNHRTSLKKLSFVIGKFGFYLVAMLAELFIYCWFGDDLITESENLALAAYDAVTSLQGCPMSMKKSLLLVMHRAQRPLRITAGGFFPLSRESFVAVVNMSYSFFAILRNFKDQKV

>LmigOR26

EGLPLQLVLPFDTSRPAGWAAGWLFCAFLTVHCVVMNMMADAFNVSLIAQLRMQLMLLRRKIVRIAEEGSSRSLDSLHTTDYRDLHYRLHKCVLHHQAIIRNADLLESCLGAMLLGQSISIGTVACFQMFQVAMSANGLQQVGKFGCYLFAMLAELFIYCWFGDDLITESENVALAAYEAVTSLQGCPLSMKKSLLLVMHRAQRPLRVTAGGFFPLSRESFVSVVNVSYSFFAILRNFKMEDQ

>LmigOR27

MAAAAPESSGGDVEYLLRLLHWSGTLRHPRAGRGASLAFYARNAAVATAVLLFVLSQAAVILGEGPADLDRFTRALCFFNTSLTWLLRLAHVALREKQFHAIALQVGSDFGEFLTPRDAETLGRRGRALRRFVLAYLCFGVAAGAGWDVFPVVRHGVCGDGLPFHMALPYRVDRPLPFAATWFYCFCMTMHVAVVTMVFDSFNVSLMAQLRQQLSVLSGNIRSLADEQRRTASSGVDSPEHTERVRYRIRTIVRHHQAIIRNVESLEKCLGDMLLGQSLSIGASICFQLFQSAESLAEAGKFGCYLSVMLAQLFVYCWFGDDLITESEKVSAAVYSVVPSLQGCPTSVKRSLLLVMRRAQRPLRLTAGGFFDLSRESFVQVLNVSYSFFAILRNFKEE

>LmigOR28

AARGVTSYYVGVGGAAVVVWNLMPLLLEAGRSLPTIAWYPYDETKTPFFEVTYLLQGISTFYCCITNVGLNVFLVSLMIYISDELKNLNDSISSINYRSIYNCCCNGDKQIGLLNWSSKIVTDDCDINVDTHIAHSKHIRQRAEFCCVVKAQDYLRMCLQYHQELIRTVKKLETTITAIVFIEFVAGIIVTCLTLFQAAVNAGNMALFVKFIMYLLYMTVGMFIYCWYGQDLMQKSEDIKWAAYSCNWQGAPKSFTDLLKIVILWAQQPLTLSAGKYYKISLKTFVTLLNASYSYYAVLRQMSDTQK

>LmigOR29

PPAAGLLYRAYTAAVVGLLCHITLPEAAGLVHFRGELRTATEVACLLFAFATTCYKLLAVLLRRRRILRFVHDLDARVAAMAEESAEARAAVSRRDRWTRRLAVLMVLQSTSTAFTWSMNSLRLSLSKGCSLRFLPIISWYPYDMTVWSNYAITYIIQFFTLIASAFSNRTCDILIITLMCQVGSLLEMLNLRFLEISRDSQSKADKRRTEWNQKHIMKRATSVLCKTIGANGLLDHVPTTSEEVPQDEMYAKLTRCIKAHQEIIRYAKELESLVNDIFLVDFLCCMIVICSTLYISTSASSNFGDLMAHFGYLVAMTYPLLFYCLFAHDIMEQSGRVAVSAYCLPWFLGNTKYRRAVCVALCRSQRPLTLTAGKFSVVSRATFLAIMNASYSYYQILREINEVKRSE

>LmigOR30

RRLTLAVTLLGCPSPGVWTAVPLLAPLVGDGAAPRNRSLPAAARYTARDTESPRFEALTALQFFSMQFSYFTTVGVDMLVVSIMIHASAQLELLNLSFGRLGQAAGSLPGNRERRRAEVCVREAADGPGRSGISRRADAEETPPREKFCQELRDCIRHHQDVIQLVADVERLLTSMILTQVLGATLIICVALFQFATNIENIGTILRVSVYMSFMVNEVFMYCWFAHNIIDQSSRIAESAYSCAWPGVPPSLQRSLLVVICRAQRPLALTAGKFYQVSRETFIQLINASYTYYALLRQMND

>LmigOR31

MCVRVPWRDSALWANARLLSVAGVWPPPSSGGWYLLYTCWLFGSQLFMVAGQLAGLWRFRGDLDKLTLDVCLTVTVVMGVIKAGAIVARRRRFFGIVRRLDAATAAQLLAGDPEEAAVVASAASLARTITVWAPVLGSLSPVVWGLAPLLLRLLGNAPRPRELPVVCWYGGWDAASPYYELLYLVQFVTIQGGYLVVMGSDLFFVSLMIHAAAQLRILNMRLVKIARNEVENDKTFGGMKCVPGYRNSNLKIIDKEWTSSATKTQDLTDETSSYDELRSWVEQHKDVIRLVQQLEQLLNVIILFQFLGGTIIICVTLYQSSAKTGEVTTLFKLQLYLGTMLSEIFMYCWYADGIVQQSARLATSAYSCGWPDAPQPFRRSLLIIMRRCQRPLSLTAGKFYTISRATFVRLVNASYSYYALLRQMNDH

>LmigOR32

ATARYTDRDTATPVYELLCVLQYFSMQYSHFAAMCIDLFFACLIIHVAAQLAVLNVRIGQIREEYYRGGAEEAVPPEERRAREDEAWQQLRECVEHHKLAIKLVGDLEDLANVIILSQFMGATIIICVTLFLITTSEQHFAALVKLQGYLIVVVYEIFMYCWFGDDVMYQNSRLATSVYTCGWPGAPQKLQRALLIILMRSQRPLGVTAGKFYRVTRETFVSLMKASYSYYALLNQMNK

>LmigOR33

MQVTRHERRFCPQPGTRTVSFLIRYRESIRTIGAIFQKHPFPELFNVVMQVTSHERRFCPQPETASMREEDIPWSDTVLWLNARVLALGGSWRPPGARGFALYRLWVLFTQFSFLIGQLQGLYYFWGDTNRILQDVCLLITTILGLFKFFTFVARQEQVFRIVRSVDDRRREQSKLGDARVTSVLEASRRAARTITVWMAGVGGLAPAVWASMPLVMRGLGLAPPERELPARARYTDRDTATPVYELLYLLQFFSMQYSYFAAMCIDLFFACLILHVAAQLEVLNVRFGQIREDYYRNGQTREARDDAKVEDVEEDAAWKELCECVEHHKGAIKLVDDLETLVNPIILSQFMGSTIIICVTLFLITTNKQHFAALVRLKAYLAVVVYEIFMYCWFGDDVMYQNSRLVESVYACGWPGAPPRLQKALVVVLQRAHRPLGVTAGKFYRVSRETFVSLMKASYSFYALLNQMND

>LmigOR34

MAGNIPWTDTVLWMNARVLALGGMWRPPWFQPKWYLLYRAWVLFTQFSFLFAQVQALWFFWGNIDKITHDTCLLITTILGLVKFFTFVLRQEDFFRMVQKIDDSRAEQSKSGDSEIVSILDASYRSARTITLYMTFLGGSSPGVWAIIPTILRRLGVFPPERELPATAWYSRRDTETPYYQMLCTLQYFSMQYSFFMAMCLDLFFVCIIIHAAGQLEVLNARFRRVGQIAGNHSADSHKQQKALEEFSGDVFSPEEIWEDLCDCIKQHQDIIELIKEIERLLSKIVLLQFLGATVIICVTLFQSSKNTDNIAALLLLQAYLGVVIYEIFMYCWYADDILYQSSRLAMSAYACNWPGAPPQLQRALVFIIRRTQRPLGLTAGKFYYVSRETFVRLMSASYSYYALLNQVNDK

>LmigOR35

MAVDAPWSDTALWLNARLLALGGMWRPPWCPARCYLLYRAWVFFTLFSFFVAQIQALWHFWGDMDKITHDVCLMISIILSITKFFIFNFKEREVFRLVRRIDDTRAEQIETGDSEITSILDASYRSARGVALMMTCLGGSIPGVWAVIPILMRRLGIFPPERELPGTSWYTGRDGETPIYETLYVLQYFSMQNSFFTAVGPDLLFVAFIIHAAGQLEVLNARLRRVGGASDARKLQKAREEEEESGEAGCGELAWRELCGCIRHHQHVIGLIKEIERMVSKIVLLQFLGATVIICVTLYQSSKHTENMAALLMLQGYLGLIMYEVFMYCWYAEDILYQNSRLAVSAYSSGWVGAVPQLQRALVFVICRTQRPLGLTAGKFYYVSRESFVSLMSASYSYYALLRQVNDK

>LmigOR36

MPAAAPWSESALWLNARVLALGAMWRPPGCRGPALSACWALYTGWMLFTQLSFLVAQARALWHFWGDVGKVTHDVCLMVTVVLGLIKFGVFSLRKDDFFRIVRQIDSARSEQSRSDDAEIASILRASYRSARNVTLYMTLLGGSSPAVWAVTPALMRKLRVGPPERELPATAWYSGRDTDSPRYELLCVLQLFSMQYSFFAAIGLDLFFVSIIIHIAAQLQVLGVRLRGIGKLGHKQSSSLSNSPILLRDVEDGFYNEKSLWMEFCSCIQQHHSIIELVKEVESLLNIIILLQFLGATVVICVTLFQSSTNTSNFMTLLKLQAYLMVIIYEIFIYCWYADDILYQVNSFTSGIYFTVRLQDLDASQVLQGIREWDAVVEDKRGGGEPDTSSRLSAAAAGKFYYISRATFVRLISASYSYYALLNQMNDK

>LmigOR37

MDAREGKISSVQSQCKPGGIIPVVELVIPDAMKRTGCGLLQADALCDYFRLVTELDAFVSEQRSKYYNNEKVIEMLDASSKRTASITKAVMAYLICWTFIVVPALFLIESPYSVLPLMAWYPFTANIWPRYEIIIMLHFLTIGYCFFTSWGMDLFFGCLMYHLSLQLRLLNYHLANIRYRCKSECVLQEGFEGKTDFSTPRVELEVIRNGREKQQVAEYTRSAEDAIYVDLLQCIKHHQRIIRYADNVENVANPVILSQFVLSVLVLCVVLFQTSSELGTLTALVRFLVYLLELLLQIFIYCWVAHQIFEECDSLLQLSFVVKTLDGRRAIRTRSVKCLLQESFWSVMIMPRPVTFSAGKIYAIDRTTFVSIVNASYSYYAVLRQINN

>LmigOR38

MKEIRNLTISNANNSMKVRHAYRLGINSEGDRSDDGLGRDRNQSFDNRAFIISVQPSESSVGAFLRKRFIVKYLPKVLSTVHRLARSITLAMLVFGAASSIGWILNSLLLGGNSMLPMVAWYGIDQASSPTYEMLYVSQSLVIFYCFLTSWGLDLLLASVMIHIAALLKTVCIHFSLVTAVSSNRTPGMQMLQRNKTLPTAGNNLETVVLEMTSTDYKYLQCVKCIQDHQRVIAMVKDLEDLANPVILTQFVAGIVVICVNLYHTTTDTHGFLWASKFASYLLMLVFQIFIYCWCAHNIMEQNLLLAEWTWENGWRLWRAAVDLSPGAGGGGRVNGYACTLLFVSVAGIGLFSSSLSSFRKLINTSYSFYAVLRQLNSR

>LmigOR39

MSKELFSLPGPNSVINVNIGVLKVAGLWPTRPYGLFTIYTVWIYLTQWAVFALDFMSLFYYWGNLNMITAVFCNLTSITAGIIKMTHFFVYKPKYYMLVNKLDALVDSQQKITHPNVDSKSILLQTSKLNKYSTYIIVTYGNLVGVPWIVLPFVVDSGDTERTLPVVEWYGITQDKSPVFQIGYVLQCLTIMYWFFASWGLDLFFGALMIHLAGQFRILNNRIANVGREVDPRLDVSSTLKIEMKEINIDAATRTTLVQRKDAELYSELRKCIMDHQEMISFMNDLEETVNFVVLVQFMAGTLVICVNLFQAALNVQDFSSVLKVCMYMFELILQLFIYCWCAHDVMVESERLSTSAYFSEWAGAPRRFTTALHILMARAQKPLTVSAGRIYTINRSTFVSLINASYSYYAILRQMSDR

>LmigOR40

MEGRLSARHRESVLKWNVWVLSSGGLWPAGPPRLFAAFTSFVFIVKWTHVLMAVRTLYLSWGDLNEITLTLLSMITMLGGSVKMTLFLKNKSAYYQLVQRLDEVVRYQEQYYLGNETMVSTFQKARKKALRLTFITLGYLNVLGPLWFVMPLLENSSEKHLPFIPMHGLNVTSLPLYELAYVTQCTATFFWHLVSVGLDMFYASVMIYVTAQLTILNLRFMNLGLETKDFIGRPSLGSITASFDNRMFADAHDKMYKELCDLVRSHQKIIEFTNYLEQVMNSTVLVQFLSSVLVACVTLFQATINSQGNTVVKCWLYLPMPAFQIFVYCWCAHDLMDQGLEVSTSAFLSAWVEGSRGLRRGLLLVMVRSGRPLEH

>LmigOR41

MAHAQRPRGLPPANVLSANIAILRWSGLWPPERRGGWARLFAAYSAVAFLSQAVAAGMTLHLIYHSWGDIYEITLTMMVTMTLVGGVLKMLHFFRHAGAYHLLVRELPAVAAGMTLHLIYHSWGDIYEITLTMMVTMTLVGGVLKMLHFFRHAGAYHLLVRELRRAALFTRGALGYLNILAPTWFLMPVISGAADDPAGRKLPFTQLKGLRADDLVGYSVAYFVQCHAIFYWNFISVGLDVFFATAMLHAAGQLKILSHRLSRLGKGPAARQHWNPQDDTTQIRQIMPQEGTNDLYTELRSCIKNHQEILRLVLLLESVMGPVAFIQFLCSVVAACVALFQATFNAEGNGVLKCTMYLPTPAFQIFIYCWCGHEIMEEFSRYSLATRRACXYSSGWVGAGRRVSRGLRVLMCRAQRPLLLTAGKLYPVNRLTFVSLINASYTFYALLRQMRDR

>LmigOR42

MRGRARLFALYTAALYACFAAVLLMALQLAYLSQRDINELTYALIVVMSHVGVLFKMTHFLTSRGAYLQLVERLNRLVGQSLADDSSGPVLAACHRKAMRLTFCSFAYLALTGAIWYLVPIVDAIRSGSEGRRLPVANPHWIDTSKTALYAFLYVVQFPSIFYFVGISVGLDGFFATTMIHVATQLRLLSLRLSGLNHGGSRISSVTKFSEDNKSDLPSPLRVREIERLDESEGMYQQLVQEIKRHQEIVSFVKFLEAVMSPVAFVQFLFSVGSICVTLFQSTFNPRPDVVLKCAMYLPTPAFQIYIYCWCGHDIMEEGARVSLAAYSCAWTGASKRSKDALRMLACSTQRPLLLSAGKIYPVSKATFLSMINASYSLFAVLQQMRSR

>LmigOR43

ERRPRLYALYTASVLASQAACIAMGLRHALDSWPDTDAVMLTFVNTATLLGGVAKLAHLCAHVRDYRRLVAALRGLVAAQWPACRRDPRLLAAFRRSYRRALRLTFGMIAYLHFIGPIWYAMPLVARATGGEQRQLPFVDLRGAVKEDLSLYVSVYLLQCHAIFFWCFVSPSLDMFFVTCMLHVAAQLGILNARLSELGGVRVPDGGEMVALPAGRRKSRNLEQHSDDSDISEELRDCVKIHQDILSFLQDMQRVMSKVAMAQFVCSSVSICITLFQATCNPEGNSNLKCFMYLPMPAFQIFIYCYGGHELIDQGLAVSLAAYSCAWVGATRRVTSSLHIMMCRAQKPLTLTAGKLYPINRITFVSLLNASYSFYALLRQTRDR

>LmigOR44

MSRAYIAHVGNSLTESFSPKLLRKRTVLFFLSLRSRGRALEMPEQKVQLQRGARICDVLRHNVLLLVATGAWPPTTRRWWRPLYPLYTASIYFSMLATIAMGFQFAYQSWGDWDSIMLTFVNTFTLIGGAVKLAHFSSHVDAYRRLVTALRDVIGTQWAHCERDAALMAAFAGSHRKALWLTWAPVVYLNILGPVWFMMPVVAWASGAPGRQFPFANVRGVLKTNFPLYVAVYFVQCHSVFYWNFLSFGLDIFFVTCMIYVSAQLHILGKRLSNVGRGPNVDQNGIVDEKQTKLQQFGQKPYKSLEVDRESNEMYAELVDCVKAHQHILSFVAVLQGVMSPVAMAQFVCSATAACITPFQATFNPEGNSIFKCLMYLPMPAFQIYIYCWGGHEIIDEGAALSASAYSCAWMGAPRRVTSAMHVLMCRAQKPLTLTAGKLYPVNRDTFVSLINGSYSFYALLRQMRGH

>LmigOR45

MAATERRQGEVGEDEVLRSNVRLLQLAGAWPPTAPRGLGRLFPLHLASVYLSQLANIAMALRLMQTARGDMHEITQALMNAMTLVGGILKLLHLSTHVPAYRRLVLALRDVIRIQRHQCERDPHVASLLARAHRKALRLTFWPIAYLNMLALGWYSIPVVFWALGWEKRLLPFFTLHGVDSYDFVLYAAIYFVQCHAIFYWCFISIGQDMFFVTCMVHVAAQLQILNARLSNLGGGQGASNELGRLYSCRAEEDGPCERKPISDTMYTELRNCIKTHQEILKFVQLLQQVMSPVAMAQFLCSVGAACVTLYQATFNPEGNSSLKCLMYLPIPAFQIFVYCWGGHELMENEENIGSVRACRSSASQWLFAGRRVTSSLRTLMCRAQRPLLLHAGKLYPVSRDTFLSLINASFSFYTVLRHMNNR

>LmigOR46

MAVLEASGRQTLGEATELWGDVLRRNVKVLRFGGAWRPAALVGWRPRLFPLYFGSVCGSLLNIITLDMVRSWLLWGDMTAVTFALVSAMTNLNGVVKMVHCFRHHGTYGRLVSELNGLVALQRPYCEADGALLAAFRRASRRAARLTVGCLAYMNVLGQMWCVVPLLTPEAPDSRESALPLVSLPGLRSRNRLWYSFAYLVECHAVFYWNFASLGMDMFFASAMIHVTGQLDILNIRLAQLRREGSTEDQFRSFASDAGRSDRQRERGDGDSSKMYSELCECVKHHQAILKYLEFLESVMSPVALTQFLCSVVAVCVTLYQITFNPEGSGVIKCAMFLPIPALQMFVYCWCGHDIMEAGLSVSLAAYSGAWVGVGRRVTGALRVLMCRAQRPLQLTAGKVYPVNRDTFLSLINASYTFYTLLRQMRNR

>LmigOR47

MVAIGSRAKPILVQQSKTSSKGQDGGEESVVSPSENVLRRNIRILRLAAVWRPPGRWRQRLYPLYFGTVCTSMLHIGALAILRSYTIWGNMTEVTFALVSGLTCFNGAVKMIHHYTHSETYYRLVDELNLLIDRQRPYCEGDAELTEALQTAYKKAKRLTWGVLLYMFVLGQMWCIVPLFMKFPPDDPSSPLPLVTITRVHKVHNHTLYSMAYLSECHTVLYWNWSSLGMDVFFGSIMIHVTGQLNILNIRLSRLSHEGVGDGLAQYSSFVKGSELHKGGIHDSASMYDELCKCVKDHQEILRYLDFLESLMNPVPLAQFLLCVGGICLTLYQITFNPDDGGVIECILFLPIPALQIFIYCWAGHGIMEESEYVSFAAYSCRWGGAERKVTNVLRIIMSRAQRASLLTAGKVHPINRDTFLSLLNASYSFYTLLRQMKNLEEENEASS

>LmigOR48

MASPDALLRHNVRLLRLGGAWPPEHGRGLRRLFPLYTASVYFCQSATIAMGALLTYELWGDVDAIMLTYVNTFTLLGGFVKLVCFSGDVRGYRALVAQLRAVARHQWPHCQADAHLMAIFGAAQRAALWLTFGPLAYLNVLGPTWFFMPLIVRATTGSHQRLLPFVNMRDSVTEIFPLYVAIYVVQVYCMFYWNIISVGLDMFFVSCMIHVAAQLRILNERLSNLGRARADDDDDSCRAEANHKNIPGFGSSQKRSFSREGRVGNMYEELRNCIKTHQHILSLLKTLQRMMSPVAMTQFMCSASGACITLFQATFNPEGNSTLKCLMFLPMPAFQIFIYCWAGHEIVYQEELLSLSGYRSGWVGCGRRASALLHILMCNAQKPLQLTAGKFYPVNRDTFVTLINASYTFYTLMRQTRDQGSIVQT

>LmigOR49

MESSARHDDSVGAKRGAVRLTLWKGPEQTTSILRLNVRCLLLGGIWPKSRGALYLAYSAFIQLCSISYIVMCMLSIFSPDGDMNDITLTLLHTFEVVCGVVKAAIFYLKRHQYYQIVRDLDQLVSSQRQYLTASKDDHLLAMLDAAHKKANFLTLVLTGYIYGLVFVWLPFPLILSPSDRLLPFVPLPGRYYKDSLLRYITVYAIQSFVPLALIMVVDGLDCLFVASVVHAEALLKVLSERLASLGHSHYTDFRLQRSSGREQEPRKGNVGDNSNITAQLRSCIIHHQQIIEFLQNLEKAMNIMVLIQLSFAMFNLCMALYQQTKIPDFTSALKYVMYLPFPTMKIFFYCWAAHNVKEQGEEVSWAAYSCAWPDADQEFQKSLAIIMCRAQRPLLMTAGRIYPINKDAFVSLLKGSYSYYTLLRQFESK

>LmigOR50

MKRGDTAVEEDEKDGEVLTWQETGGSVLKYNIRQLHVFGVWPLPGSAPFHAYTVLVSAIGLAGLAQDLAGVCACWGDLQEVTMALIHILSVSSGFVKLAFFVRRRRHFNALVRRTDRLVAAQGHFCDADATLRATFRASHRKAVYVTLLAYGYLSVQGVIWLPLPLIAHPGERRLPFMQMPAAATASVYVYALLYSLQCLSSMFVTFVGVTVDCFFAVVMIHTAVQFRILNTRISALRADAAVPVDTGTAVGGQTSEHDSLYKQLCQCIQTHQRLLRFVTYLDSVMNPIAMTQFTFGVIVVGITLFQASYSPSSSTMFKCVTWLPMPSTQIFLYCWGAHDIMDEGQSVSRALYSCGWVDAPPGFKRALRLVMSCAQRPISLTAGRVYAINRATFISLMNAAYSYYTLLRQFNSR

>LmigOR51

MKKEKERRVQLEDLGSGEDLSRAEVESSVLRQNILLLHYMGLWPMGGSRAYRCFTAFNLSSSATIIVMNVVGVCFSLSDIDQVTGALSTILPMSGGLLNGLFLLHQRPTLCRMVRTVDRLVTSQAQFVERDARLVAIVGGARRRTLVVTLGVSGYLFAIASYWVVIAFTLPPSLRVLPFVQLPWMPSSDPGLFWSTFGAQLYTAPFCSYTTLFVEFFFLAVMLHLSAQFRVLGSRFASLGRSSASKVAADSGAVYEELRLCVETHQELLRFVRFVDNVMSPFAMLQFVAGTLAVCVVLFQAANNQDLNTNLKCAGWLPGPSLELYIYCGGAHEVVHAGEALVQAAYDCLWYNVAPRIGRAIRLVITRAQVPPVLTAGHLYPITRPTFVSLVNAAYSYYALLSQMQNK

>LmigOR52

MAKQKEKSSRLQDLDSGDGEDLSWDEVSHSVLKNSVRVLYCMGLWPLRSSRAYHCFTAFSLASSAVVIAMDIVAACYSLGDIDQMTGALSTILPMSGGLANGLLMILHRPDLCRVVRAVDRLVVHQQRYLRQDSHLAAVVARVRRQTLLVTIGVSCYLITIASYWIVIAFGKPTGLRVLPFVQLPWVQSSGLAHYWSTFAVQFYTAPFCSYATLSVEFFFLAVMLQLSAQFEILGSRFASLGRNPPPKAVVTTKSDMGTADSDAVYEELCLCVKTHQELLRFVRFLDDVMSPFAMLQFVAGTLAVCVVLFQAANNQDLNTNLKAAGWLPAPSLELYIYCGGAHEVVYEAEALVQAAYDCLWYNTAPRVSRAIRLVITRAQVPPVLTAGHLYPITRPTFVSLVNAAYSYYALLCQMQNK

>LmigOR53

MSAEPAQTWRSSASSVVSYNVRLLFLCGLWPLRRGRAFSAFTAAVLVAAALHAAGAFVGLCTEPGGLQEVTLALANLFVVCSAIVKSCFFLADRTRFCTLVSTLDRLVQVSGQQSAVGAGLRSRLSASARRAVRLTLAFHLYVLSALVGWCLMPALKRQRRLPFQQLRWLDTSSAAVYGASYALQCFATFFCSFINTHLDVFFMAVMIHVADQFAILAARFADLRLDADADPEGRLPQGERAVLAEDAYQQLRLCVRSHQELVRLVQLLDDVMSPIAMTQFVVGAINACMVLFPATYSTDVGAVLKCWAALPMVGIQIYLYCSGAHDIMEEAGAVSGAAYSCSWLGADRRRRRALLLVTCRAQRPLQLTA

>LmigOR54

PRLAAAFRESCRWSAKLTFLFFSWVLLALVAWSLMPLTLYPRVRLFPFQQLPWPVLTQSPTYWFLYLHQILATFFFCSIDMNTDCFFATVMTHMSTQFKILASRIADLRLRENTQKSKLCAEVDTSTPHDEMYKELCLCIETHKELIRLVGLLESLMNPVAMLQFLVGAVSSCVVLFSATYSPDSSSAMKCWGSLPLLLTQLFLYCSGAQHILDESE

>LmigOR55

MVDRSVETPLTWRESAESVLRPNIRLLCSLGLWQPVDSMLFHAFTAAVLAVGVAHLAVAALGIWQRPADLAEVAIGLSNAFVIFTALSKAVLFLTRRPLFYSLARLVDQMTAEQKAFRAGDPSLQEVFSAARRSAGRLSVFFHWYVLVADVLWSLIPLVQASREKRWPFQQLPLDGWATSPAYQLSYGLQCASTLFFSLISVDVDCFFVAVMTHITAQLKILTFRFAAIGNRMYISDTSLNNQTASTKDASHEKLRGCVQTHQNILRLVSFLNVVMSPVAMMQLAVGVVSSCMVLFPAANSTDSAVVMKCWAALPVLGVQLFLYCSGAQRLIDQAEAVSGAVYSCAWPEAGGRVQRSLLVVVSRAQRPPELTAGRMFPINRPTFLSLVNATYSYYTVLKQVNSH

>LmigOR56

MDQRATSQSEEEEWAGQSVVRTNTHLLRLLGLWRPARSRLYDAYTAAVLAVGVADLALASAGLWLRPGGLAEVTLGLANLFVILTALSKSVLLLGRRPLFYELVRRVDGATAAQRPFCGEDPLLARLSADARARADRLSRAMHWYVVFAALSWSAVPLLAPPGDRVWPFQQLPPRPWARSPLYEASYALQVAGTTYFALINMDSDCFFMAVMTHVSLQFRILASRFAKLNSTEESLADKKASDVRVTSSESTLPVDDTDRELRACIQTHQKLLRLVNFLNDVMSPMAMMQLALGVINSCMVLFPATYSEDSSDVMKCWGALPLLAIQVFLYCSGAQRLADQLVQATYSYYTLLQHFNSH

>LmigOR57

MGWDSKEDQPLTWQYTADSVLKYDVRILHMIGVWPLSGSQLYRCVVTVIIALCLGHFVEAVINLYTLHGDLEDFTLALSNVSVVIVGILKVTFFLRHERGYCHLVRWLDTIVASQREYTRGRPHLEEAFAGAQTLAVRITRGFCMYNATVVLAWVLAPLAAPPEAKRLPFQQLPFGEGSPFSLYALSYAMQGVSMLLIALISVQMDCFFTAAMIHAASQLRILNSRLSDLQLGKAGLQLQGGTTLDSMYDELRLCIHTHQEITRFVEHLENVMNPIAMMQLAVGVFNGCMLIFPATYSAENDALVKCLAAAPTISAQLLLYCLGAHSVREQGEAVPLSAYSCGWADASAAFRRSLLVVMARAQKPLALTAGRIYPIQRATFLSLLNAGYSYYAVLRNFNSR

>LmigOR58

MGWNPREEKPLTWKETANSILRFDVRILYVVGVWAMPVTKLFRAYTAFTLVLAVGYSVEAVIHIWLVRNSMEEVTLAVSTYAVVVTSACKLVSFLQHEPGYWRLVRWMDAVVADQRHFCEERPELRAIFDEARKRAKRYPNALRVYNTSLIISWVFIPLLAPPGLRPLPFQQIPLSETEDFPLFLFSYLLQTFGMLFMCLVSGCLDSFFTAVMIYTAAQFRILGLRIAALRQDNDEIKRQARSDVYEKPRKGAATDHVYEELRLCIRTHQEITSFVTHLESVMNPIAALQLITGVINGCLMIFPTAASSESGALLKCIACVPTISAQVLIYCLGAHAVMEQSEAVSAAAYGCAWPDTSPRCRRSLLVLMTRAMKPLTLTAGGIYTIERSTFLSLLNAGYSYYALLKNFNSR

>LmigOR59

MSWGSEEVEQLTWKYSAKSVLKWNIRILNLVGLWPLTESLLFRSSTAVIVMLCVAHIAEAAVNLGTLRGGLQDFTLALSNVSVVCVGVLKLTFFLRHERSYCRLVRSLDVLVDSQREFVQGQPPLAALFEATQKRTVRVTVGFLVYAITQLVAWSFAPMIAAPGTWRLPFQQLPLTDETAFLIYELSYAMQVVSIIFIALINCQMDCFFMATMLHTAAQLRILSARIMCLKLQNEDIPAVFRSNEGEEVQASATHDSTYRNLCLCIKTHQELVRFVRHLDQVMSPIAMMQLGLGVFNGCMLIFPAAYSAESDALVKCLAAVPTISTQLLLY

>LmigOR60

MDWDPKETEPLTWQYTTHSVLKYDLRILHLLCLWPLPGSLFFRLLTAFLIALCLGHFVEGLVNLCTLSGDMEDYTLALSNISVVTIGTVKTAFFLRNERKYFRLVRWLDALVAAEKKSVSSRPLSEAIFPAAQKRSARVAAFLLLYNCFLLFIWLTAPLAARPEARILPLQQLPLTDSNAYPLYELSYAMQALSIFFIGLINVHLDCFFTVAMIQTAALLKSLASRLADLQVRNAPSRRNVDEGRKNIVTADDMYRELCLCIRTHQEITRFVQHLENVMNPIAMMQLALGVFDGCMLIFPAAYSSETSALVKCFGAAPTVCMQLLLYCLGAHSVREQGESVSVAAYSSGWADASARFRRAVQVVITRAQKPLVLTAGGIYPIQRATFLSLLNAGYSYYALLQNFNGR

>LmigOR61

MQSKDPVSKSVSFRPPDGGSQNKDGDSAQPTESQDSFLTWKGSSESVLKYNVRCLCILGVWSLTRSRLYYLLSGTAFLLGVMHIVVAIFGSYLYRDNMEEMTLIVANMFVVCAGVTKLVIFVVYRNNYRQLVTVTDGLTDRQRSYCQGDPALKSILEDSERLAVRLTLFVPAYIATLSVVWVPMPLIAYNERRLPFVQLPFVNEVSASTYALLYVMQTVPSLLFFNVGFAVDAFFASVMIHAATQLRILFHRIKDLRLDGRGTLKLLSGDRHDIMYGELCTCIQLHQQLVRYLSFIGKVMDPIAMTQVVFSVLIACTTLFQANYSADTNTAFRCLAFLPTPGTQVFLYCWGAHNLMEQSAAVSEAAYSCSWVEASRRFKRALCLLMCRAQRPLVLTAGGLLQINRPTFISLLKASYSYYTLLGRVNNR

>LmigOR62

MKQSASLEDCSPLTWEYTQQSLLKLNIRLLWALGVWPLPGSWVFSLLKVWLAALAVGNAVENVLGVWKNWGDLTEVTYSLLNAFTIGAGVAKTWHLSRYQPRYCLLVRRVDRLTRSQRRYCDGDAAMRAVTLGCRRTARRVTLSAFAYLTALCLIWMFMPLVAHPGERLLPFNHIPWEPRRFPLFYELSYAVQSASSVVYVFISFALDCFFAVVMIFLTEQLMVLNLRIRQLYARRDGDGSVLVAKQHSDKTVLDKHEEMYKELCLCIDTHQDIIRLLSFLDAVMNPIVLTQFMLSVMAACVTLFLESYSPDSSSVLNSISYLPTPGIQVYLYCWSAHNVLEEGFAVSEAAYGCAWYEGGGRFKRALRIVMCRAQKPLVVTAGRLYPVSRATFVSLVNASYTYYALLSRVHNRG

>LmigOR63

LTALCLIWALSPVVVHPGQRWLPFNHFPLEPAPLPLYYELSYAVQSASSLLYIQVSFGVDFFFTVVMILITEQLMILNARLAQLHLYAGGGKSRAAATRVMTTAATEDRDEMYEELCHCIDTHKDIMRLISFLDSVMNPIVLTQFTLSVMAACLTLYQQTYSPDGNSVMKSASYLPTPGIQVFVYCWGAHSIMEQGEAVSAAA

>LmigOR64

MPTPTATETMTWSDSGNSILKVNIRELCLSGVWPLTGRKLFRVYSVIIWILGLENIVEAMVGIYLSNGDLEQITLVLPNTFTTAGGVFKMAFFLRDPYSYNALVRLMDELISDSSRYSTGNQQMLSIVRESRRSARRLSVFIYAFISTQIVIWFPMPLIAYAGEGKLPFIQHPWINSTTFPAYDTMYALQCLSSGFHIFISLGMDCFFAVVMIHTAACLRILSLRISALRSGDAGSSEVPVTSACSWGRESVAHDEMYKNLRACICSHQKIIGFISYLETVMNPIAMTQFAFSVLVACVALYQATYSEDMSAAYRCASFLPTPGAQVFLYCWAAHNIMEQGLAVSAAAYDCSWVSGDARFKRALRILMCRAERPLVLTAGHLYPVNRPAFLSLVNASYSYYALLGRVQSR

>LmigOR65

MEHPYLAAVTPCRVMKESGGGQRAGADDTASLTWRQTAGSVLKVNVRGLALFGSWPLPESWLYHAFFAVVFASNLGNMAEAAVGLWLGRGGLEEITLVLPNTLTTAAGVCKMVFFYRDRGRYYRLVRRTDLLAGSQLAASGRHGADAVRQADRQSLQLTYTVFAFISLQIIVWFPMPLYAYRDQRKLPFVQLPWNEDKDIPVYELSYALQCFSSFTIIFITLGMDCLFAVIMIHVAAQFEILIVRIRNLRLDLQTTGVTQSKASLGQLSRNDVSSINVQTDYKEVSHHQQQINEAHDKLYSELCHCVESHQEIIRFVRHLETMMSPIAMTQFVFSVLVACVALYQATYSDDLSAVFRCAGFLPVPGAQVYLYCWAAHHVMEQSEAVSAAAYACPWIEA

>LmigOR66

SYAVQCVAGLWMAEISFGMDCLFASVMILAAAQLEILSGRILKLGQSPYVAKKGSPTPDEIYKELCRCVETHQKILRFVSRLQETMSPIAMTQFVCSVLVLCVTLFNATYNKDIITSLSSMTFLSNPCGQVYLYCWAAHNVAEKANAVSTAAYSCSWVEGSERFKRAVRILMSRAQKPLVLTAGSLYPIDRAAFLSLVNTSYSYYALLGQINNR

>LmigOR67

MEDKLSWTFSGNSALKLNIRHLWLLGTWKLGESRLFKMQSTVAFGLSIWSTVECILAVYFIWGDLEQTTLVLLITCTCSSGVVKMFIFVYDRRRYDSLTLRLDALLSLQTGPCSEDPALAAISDWSRKKASRLTMGLLLFMLSQSMVWYFVPLIAHPEERSLPFVQHQWDNNSLYELSYGVQCLSAVWISQISFSVDCLFASVMILVAAQLEILGQRLINLKNGRDSAEKEEKKQLDSKTGESMYDDLCLCIETHQEILRFVTQLQDTMSPIAMTQFALSVVIACMALFQATFSEDFSAVLKCASFLPIPGGQVYLYCWAATNVTEQAEAVSAAAYSCSWVDASERFKRSLRIIISRSQKPLVLTAGHLYPINREAFLTLVNASYSYYALLSQMNNR

>LmigOR68

MEDKLSWTFSGNSALKLNIRHLWLLGTWKLGESRLFKMQSTVAFGLSIWSTVECVLAVYFIWGDLEQTTLVLLITFTCGAGVVKMFIFVTTGRRYDSLTLRLDALLSLQTGPCSEDPALPAIADWSRRKASRLTMGLLLFMLSQSMVWYFVPLIAHPEERSLPFVQHPWDDGGLFGLAYGVQCLSAAYVSQISFGVDCLFAAVMILVATQLEILGQRLVNLRNGRRVAGRREKEQLARKTGESMYDDLRLCVETHQEILRFMTQLQDTMSPMAMTQFAVSVVIVCMALFQATFSEDISAVLKCVLFLPIPTGQVYLYCWAANNVTEQAEAVSAAAYSCNWVDASERFKQSMRIILSRSQKPLVLRAGRLYPINREAFLSLVNASYSYYTLLSQMNDR

>LmigOR69

VVQSARRRAYRITLGMLLLMFSQYFVWYPMPFYVDPGARRLPFAQHAWDNNTHLYGLSYFAQCAAGLWMTQMSFGLDCLFASVMVLLAAQLDILARRILALGSGAHDEKAEYPEKKPAPRFGDQMYDDLCLCVQSHQKILSFVIHLQNTMSPVAMTQFAFSVLVICLGLFQATFSEDFSAVFKCASFLPIPCAHLFLYCWAANNVTVQAEAVSAAAYGCSWVGASERFKRALRIIVSRAQKPLVLTAGHLYAIDREAFLTLVNASYSYYALLSQMNNR

>LmigOR70

MEMYQGKGMLKTLSWTESGRSVLKLNIRHLWLLGVWPLGHSQVFKVYTSFTFAMGVWSVVECLLAVYFTWGHLEETTLVLIFTSTCSCAIIKLVFFLRDERSYSLMVREVASVMAAQSEACRDPALAAILRDSRSRTFRLSLGMLLFMFSQCFIWFPIPIVANAGERRLPFSQHGWDNNCHFYELSYTLQCLSGLYMSQISFGLDCLFASIMILVAAQLKILSGRVLKLNQEVIPPERNDSVLLRNQLAGDKYCDKFYEGLCFCIDSHQRILSGGVQTEECLRVAILDVWRVALFCWNWFVTLLQDTMSPVAMAQFASSVVIACMALFQATYTLHSALYHIHEQLLSVDSKNVLSAIFLWSPSPWHSPPDVQLGTIPRSFANGEGESVSVAAYSCSWVEGSAAGRLYPINRAAFLSLLNASYSYYALLGQMNKRSMKDLASHN

>LmigOR71

MEKYRGKGTVESLSWAESGRSVLKLNIRHLWLMGVWPLGHSPVFKVYTGLTFLMGIWSVVECLLAVYYTWGQLEETTMVLIFTSTCSCGIVKLFFFVRNESSYSLMVREVASVMAAQSEACRDPALAAILRDSRSRAFRLSLGMLLFMFAQNFIWFPIPVVAHAGERRLPFSQHGWDNNSHFYGLSYTLQCLSGLYMSQISFGLDCLFASVMILVAAQLEILSGRILKLNQEVILEQRNESVLWKNKMTMDENRDTFYETLCFCIDSHQKILRFVTLLQDTMSPVAMTQFANSVVIACLALFQATYGEDMSAAFKCACYLPIPGGQLYLYCWAAHSVTENGESVSVAAYSCRWVEGSARARHALRTLMARAQRPLALTAGRLYPINRAAFLSMVNASYSYYALLGQMNNR

>LmigOR72

MEKYRGKGMVESLSWAESGRSVLKLNIRHLWLMGVWPLGRSPVFKVYTGFTFAIGIWSVVECLLAVYYIWGQLAETTVVLMFTFTCSGCIIKMFFFVCNERSYSLMVREVASVMAAQSEACRDPALAAILRDSRSRAFRLSLGMLLFMFAQNFIWFPIPVVAHAGERRLPFSQHGWDNNSHFYGLSYTLQCLSGLYMSQISFGLDCLFASVMILVAAQLEILSGRILKLNQEVILEQRNESVLWKNKMTMDENRDTFYETLCFCIDSHQKILRFVTLLQDTMSPVAMTQFANSVVIACLALFQATYGEDMSAAFKCACYLPIPGGQLYLYCWAAHSVTENGESVSVAAYSCRWVEGSARARHALRTLMARAQRPLALTAGRLYPINRAAFLSMVNASYSYYALLGQMNNR

>LmigOR73

MKKSGMEDLGRTVSWSESGRSVLRLNIRHLWLFGVWPLWDSPLFILYTGYGLSLGVWNVVEGALAASCTWGDMEQTTLALMATSTNCNGLVKMAFFLRDRRRYQALVRRVAALVALQGESCAADPLLGRVQRGSRRRAFRLTAAMLLFMFSQCFVWFPMPAVAHADEGPLPFAPDNTTAYGLSYLAQCAAGLWMTQVSFGMDCLFASVMVLLAAQLQIVAGRIARLGEQNYDFAFSFVGHLQDTMSPIAMTQFAFSVLVVCFGLFQSTYAEAVTMAAYSCSWVEASGRFKRALRILISRAQKPLILSAGHLYPIDREAFLSVRS

>LmigOR74

MGDAEQPLSWSETEHSALRLNLRHLCLFGAWPLSRCRAYHAYGAFNLALGGWYVGEATASLCFLWGDMEEATLVLISAFNIASIVLKLALFAARRPRYAXXXXXXXXXXXXXXXXXXXXXXXXXXXXXAARLTAAMLLLMVSQYATWFPMPLLASAGARRLPLAQHPWDNNSHLYALSYAVQCAAGAWMTQISFGIDCLFVSVMILVAAQMKVLASRVASLRTADVGFGRKQHPLSSPGEQVYRELCVCVETHQQLLGFVKELENAMSPIVMTQFACSVLVTCVTLFPATYSTDFSAVIRCAGFLPVPLGQLYLYCWAAHTVSEQAEAVSAAAYTCTWVEASERFKRALRIIVSRAQKPLVLTAGRIYPINRAAFVSLVNASYTYYALLCQINKRSTQRVP

>LmigOR75

MESGAAEDVEGPMGWSESGGSVLRLNVRHLWACGVWPLPGGWLFEAYAALGLALGAWNAAESLLALSFCWGDMEETTLLLTSTFTIGCGSAKMALLLARRGRYRALARRVQALASLQTGHCLADPALDDIRRGSQRRAFRLTLSMLLFMFSQCFVWFPMPVLAHWEERRLPFAQHAWDNNTRLYALSYAVQCVIGAWTSQLSFGVDLLFVAVMILAAAQLRILTIRIASLKTESWKVKPEGGARCEDVRPENGRDVMYENLCLCIDSHQKILRFLKHLENTMSSVVMTQFCFSVLVACVALFQATYSTDFTAVLKCASFLPVPGGQVFLYCWAAHNVTEQAEAVTMAAYSCSWVEASVRFKRALRILISRAQKPLVLTAGHLYPINREAFVSLVNASYSYYALLGQMNRR

>LmigOR76

MNTIDEAVRANRLRWWGHVTRMGEAMLSKRLMSSAAEGPWLETSCQHWCRSRLFGAYTLFAFSLGLWQTAESLLSCYFSRGRMEQATMVLMTTFNIGGSTVKMALLALQRRRYFSLVRRTDLLVTEGAPPADCSKERPRSLPGPVAAAQXAAHGRHAAADRGAHPWDNNRRFYALSYALQCMGAAWTTQASFGVDCLFVTVLMLVVAQLEALASRVRAIRVEPEVGGGAADKAADQMYAELCACVDLHRKILSFVKQLESTMSPIVITQFAISVLVACVSLFPATYSTNFTDVLLCAGFLPVPLGELYLYCWAAHNLTQQAEAVSAAAYSCSWVEASERFKRALRIIISRAQKPLVLTAGRLYPINRAAFVSLVNASYSYYALLGQINSRSTETTH

>LmigOR77

MKRSGMEDLGRTVSWSESGRSVLRLNIRHLWLCGAWPLPGSWLYRWYAILGLVVGAVNAVESAVSLYFYWGDMEETTLLLTSSVSNGCGTVKLAVVMRNQRQYHALARRVQALMALQGDLVAADPALSAVVEAARRRAYRITLGMLLLMFSQYFVWYPMPFYVDPGARRLPFAQHAWDNNTRLYALSYFLQCAASARGTQLSWSLDLLFMSVMLLAAAQLQVLTRRITALQEEKREAKLGNAANSEKAALADGGGNMYDNLCLCIETHQKILSFIKYLDDTMSSVAMIQFCVSVLLVCVALFQATYSTDSTAVLRCALYLPMPGTQVFLYCWAAHNVTEQAEAVSLSAYSSSWVGTGRRIKQALRIIISRAQKPLVLTAGRIYPIDREAFVSLVNASYSYYALLASMSKRE

>LmigOR78

HDWSGDGGSFYWPSYAQQCCSAFWLGKISVVLDCQFAAIMVLVTAQLEILSARLANLRPDGRALRESPNCKVKLSYVDHDSEMYDELRRAIQSHQEILSFVSCLQQVMSPLAMTQFVCSVIVICVVLFQATYSQDFATVLKCVAFLPVPCGQVFIYCWAADNMTEQAKEVSSAAYRCCWVDAGPRFKRCLLLVIRRAQRRLVLNAGHLNIDRAAFLSLVNASYSFYTLLAQMNRS

>LmigOR79

ELSYAVQCVAGLYLSQISFSMDCLFAAVMILVAAQLKIVSCRILKLKAEESGEQGGGSGDVGGGVALENEQKPYENLCQCIESHQQILRFIIRLQNAMSPIAMTQFFFSVLVACMALFQATYSKDFTAVFRCVAFMPIPCGQVLLYCLAAHDVTEQAEAVSQAAYSCSWVEADRRFKRALRLVMCRAQRPLVLTAGRLYPIDRGAFLTLVNASYSYYALLGHINRRSMQDQL

>LmigOR80

MQTGEVEDADGHLSWEETARSVLRLNIRHLHLFGLWPLRASRLFPLYTAYAAALGVWNTAEGFLAVYFSWGDLEQTTLVLMNTFTNASGLAKICFFARDRRRYASLARGADRLARLQAQACARDRALAQVLRRARRAARRLTAAMLLFMFSQCFVWFPMPAVAHADERRLPFAQHPWDNNTAHYELAYAVQCVAGLWLSQISYGVDCLFASVMLLAAAQLEILAGRVAALGERRNGRGGEPLPADAPDSMYADLCACVEAHQKILRFVSDLENTMSPIAMTQFVCSVLVACSSLFQATYSKDLSAAFSSMSFLPIPGGQVYLYCWAAHEVTEKAQMVSAAAYGCSWVGASERFKRALRIIVSRAQKPLVLTAGHLYPIDREAFLSLVNASYSYYALLGQMNKR

>LmigOR81

SRLYDAYSVWTILQLLMTGVGQMAGLQGHWDDLQTVFTSLCFDLTVTCTIIKGSIFVAQRGSLDALSRHLEANARQFCSHLPAERRALLERARNLSRVIVCSFQSVGGVTLVSFITGPLVQNGRDRELIASAPGNATLDRHLGHNYPMLMWWFGGLPVSSPGYEAAYLIMCYWLVLMYICTNVPDAYYVGLINYISAQLRLLHIALRQIAHPGADDALAEKLGHIYGLDVKGGSPHKDGAQDASRDSVYDRLVECIRFHQEIIKCVDEMESLLSLTVLIQFFTSTLVICLTAITVINTEAAYLPTYAAYLATMFYQLFIYCWYGGEVYLESESLQFSAYSCNWPYTDARFRKTLKICLARMQRPISLTACKFYKLSRETFLLLLNGSYSYFTLLLQMNQKDD

>LmigOR83

MDDGALPQPVVDLWRPCLFLRAIGLWAPARLRWLYSAYTVWSLLQLVVSVAGQLAGLQGHWDHLPTVATSVCLIVTSICTLFKASSFALRRGRVDSLVSRISHNLSTFCAHRPRTRAAVVWAARRRATRMFDTFLGIGGVALVFFYLGPIIQNAKDARVAASAAGNETLPPLLGRNLAMLLWWPSGQPVETPAYQLTYVGVCYWLMLLYLSTSTLDAFYVTVIIYLSSQLKVLNVDFLSITEGDDDSPAEETDPLSKRGGKHELEAKLPKGYEEATDQRTQERLLECIIFHQEIIKTVDEMESILSASILVQFLASTLVICFTAFVITTAENKQDLPTYITYLATMFYELFLYCWYGNELLAESERLQTSAYSCGWVGRSAGLQRSLRVVMVRLQRPVCLTAGKFYQISRETFLLLLNGSYSYFALLHQMNDH

>LmigOR84

MAAGELPEPLVDLRLPCGVLRAVGLWRTGEGGLFYSAYTVWCLLMLVCSVLGQLAGLQGHWANLPTVSTSVCLTLTTSCTIFKALVFLRQRRRVDALVSRVERSVSAFWPLPPARRAAMLLEARRSALLMFSIFFGIGAVALSLFYAGPALQNIKDRELIDSAPDNATLDRHLGRNLPMLIWWYGGQPVEAPYYQLAYALICYWFMLIYLSTSTLDAFYVTFIIFLSSQLKMLNAALADAVQSAGLSDELGNRSGGQPTGAAAGRRADYRRLVHCILFHQQIIQSVHEMESLLSPSVLTQFATSTLVICFTAFAVTTSTKKQEMPAYATYLATMFYELFMYCWYGNELLEQSDRLRLSAYSSAWPDAGGHFQHSLCIVLSRAQRPVCLTAAKLYKLSRETFLVLLKGSYTYFALLHQMNDRQYNA

>LmigOR85

VCCRQLSLHASRPRAGAGMEAHLSPAVRALGAVCLWKPSRAGCWYHAATAAGSLAALSLLASVAAGLPAQWARGDITSFSMNAYVCFAIFAAQIKVSTFGYWGGPQRLVAQLGAERRAAGAAEAALPPGRAERLLGESGTLLRRSAAAFYACGHLMMVAWYASPLIANARLAPDPDTNATLPRHLLFDAWFPFDPVPSPNYEAALLYQSVTLYIAFITTAVIDVFYVSVMVYLGVELEILNEAVARSCRPFEEQENKDKDKEGEKKEEGRGQGDDCSLLVACVRHHQHLNRCVGTLQEVMGISIFVQFVFNMLLICVYAFVITTTKSDFGTLAKFAMTLESYLFENLLYCWFGNNLIEQSERLPFSAYSSAWPDAGRRFQRSLRILALRASRPLQVTVGSLYTLSRQTFLHLLNGSYSLFAVLHHLNSK

>LmigOR86

MAKYSVRGGGGGAPALDLRLQLRLLRLAGAWGPSPSASSTSLPYAGYSAAVVLLLLAFVGSQVSAMLHFWGDILSVTTNACVTFTYTMAMFKLLVVLIMRPSAEYLIQELNRCMQEYGRDLSSEKAAVLARCGRLSRHVAAAHVAIGAVAYVCGVALPAARGRLCPSAACRAADGFPVLVWYPFPFTVSPAYELVFAAVSLDFFYGYILSTTLDGFFVTLIIYVSGQLRLLNLMAQNVCAGAGGGGSEASEQRVRDRLAQCVRYHTDVDRCVQRLSALLGPILLGQVLADVVTISATAFVTTTGKTDSGWVFKYGSYLAAIAEQLLLYCWFGNDVLTESERLQLSAYSSQWVSAPARFRKGLLVFLCRAHRPLRLTASKFYTISRETFLLLMNASFSYYAVLRQLNSD

>LmigOR87

RRESLRSHVTATCLMNCGGGRRLRTDLRLQQWLLFFVGAWAPHRGAPAVCSLLYGVYSACVVLVLLLFVASLLFAMVHYWGHMLGVTMNACLMFTYVMNSIKIVAFLKMRPAIDQFIDELDNCMQEYGGEQQSERAALFGWTALKSRIVSVARLSVTAMGCVYWSVMPAVRARACGDTVRCRARVGLPAHVWYPFSYTQSPVYEVIYAGVAAGLMYGALLSSIMDGFLVSLFIYMAAHLQMLNLMLQNLCVDQPQDGSKGLPPGHHQHLCRWRLAQCVNYHCRIDRSVQRLSMLFGPILLGQFMMDIIAISATAFVAIAKNADSTWLVKYTSYLSAVIQQLLFYCWFGTDVLTESERLQTSAYSSQWVDASPLFRLELRVFLCLAHRPMRLTASKFYTISRETFLMLMNASLSYFAVLREINAK

>LmigOR88

MVNVQKAGGGGRRLCTDLRLQQWLLFLVGAWAPHRGAPAICSLLYGVYSACVVLVLLLFVASLLFAMVHYWGHMLGVTMNACLMFVYLMNIVKIVSLLKMQPSAEEFIRELDRCMQEYGQSLEMEKAAVFQWTALKSRIVSVARMLVALSGCLYWAVVPAARAHACGGTVQCRDQVGLPAHVWYPFHFTHTPVYEVVYTVVAGGLLSGALISCIVDAFFVSLIIYQAAHLQLLNLMLAAVGTEKCYQPGPPTSPGSKRRLAEESAGAEQRMHRQLAECVSYHCHIDCCVQHLSSLVGPILLGQFLMDMVTISATAFVAIANNADSAWLLKYTSYLSSVVQQLLLYCWFGTDIITQSERLQLSAYSSQWVSASPRFGRELLVFLCRAHRPLRLTASKFYTISRETFLLLMNASVSYFAVLREISSK

>LmigOR89

AYAGCVVSALLFLVASQLSAMLHFWGDLLSATNNACVTSSYSMAVFKFFAFRAMRPSVEHLVRDLDRCLQVYGREYAAEKEAGFGACERQARLVSRLQVSMGVSVYLSWVVLPAIRAQACRSTDCRVHAGFPALVWYPFAFTEPPVYPVIYAVLSIGLFYGCIIFTSQDGFFWSLIIYVGAHLRFLNFMVANMSSSEADAKIPRGSPEMEENMRSRLKECVRYHNDIDRCVQRLSSLLGPVMLGQFLTDIVTISASAFVATMLKADSGWLLKYGSYLSGTIEHMFLFCWFGNDILTESARLQLSAY

>LmigOR90

MKKASAQYSQESLSARRGLDVSTCERILHWFSCWRGGSAWYVVYVVVARASVAGVVVSQLGLIPHVWGDLYSTSLCVYLTLAVLSAWFKMVQFDLARPKVDALLQELRGELLLHAEDLSEGTEDVFRLFQKRSGAFRRVFLVLDHLTILSWAAKPFLDRLLGSDGPLSLPQDAWYPFDTTRSPYYEIAYLHQVVSLYVAATCVVTVDVFFTTLFMYVTAQLRVLNLHLRAMDCRPQLTQSEKITRCDKGVVAEKSGDANRLPSADPPDAYAQLVHNVQHHQVIIRTVKILEDAMGPCVFVQFLFNVIVICIYAFVLAGVHSMNIGSLILCLTNLISTCVENFGYCWFGNEIMSQSEQLAFSAYSSGWVGGGGRFQKALVVLTMRAQRPLCLTVGSLYTLSRETFLALLNGSYSFLAVLLQMNNSN

>LmigOR91

GGSALAVHAYYCTVLLANTAKTLVFAACRHSLGRAIHVLSRCPRAERSEKSGASARLTFALPQVMVLLAVATHSLVPLLGAGDGACADPEATGQGGGGCFSGRFPLELWYPAAALATPLYQVVYALQLVAIYCTCHTAINVDLFFFAVTNHASSHLQELNDALCRMGVRHTPVNRRRGSDESDHSADGKHGNGPLRHTSEESLVRAEHQRARYQELVRLIRVHQTITRAIKELEPVISYALFGPILTNVLNICLHMLVLTTERDNMGTNSKAFVGILFNLIQNGLYCSFGETLTHQSDRLFISIYSSGWENGCRRFKKAAAILMFQTRKPVQIKVAKLYTLSRRTFLQLLNNSYGLFNLLYQVKNPE

>LmigOR92

VQRPAAGMRVTAVHKQTALASEAGRDLMGPGEAVLRLLRVWPPPGPEARGLLHRLPYPLLALQAGMAGVLASTAAHLHQGGAGGDEESEAQQTTVALFIVGTIIGMLVKIWSFMGQRGRMQVLLSLLLEMRRRYLRDHTGARPRAQDHGVTLFYILQGNAVLAALAWSVQPLLSGSARRLPLPAWLPFDATVSPYYEAVYAGQALSLLLVPQISLCINICYFALMLHLAAELAVLCDNVAAVGWRRTSKEPAPQSQQQVLDREFAASSENRLLEDNVRHHQLIIRAVSELQQIMSTSVYIHLFVNMINVCSHIFVISVVLLETGEMAVVVSQACSLAVFLSGIALYCVIGHTIIDQSERLPEAVYSSGWTGADLSFRRSVSILLVRASHPLSITVGKMRVLSKPTFVQVLNGSYTLFNFLYRTQSDKEQRERLGLQDS

>LmigOR93

MRLRAALLAAAHLVLPVTLSVNFLSCRFDSLDLLATNIFLLSGMAGVSSKALLFVLDRERFELLLLRLQRTRMRFPDNSGARERRRRMATRVYYAQHGSAQLVVLLWVSVPGVTALVSGEGRELPMPLCPPATAADVHLSPCFELIYALQAACLFVAVEGMISLDSSYLTLMLNIATELEVLNDNLVSIRSGRLPEDKKCSQLSDQSPSNPAHEDDMYFQLVENIKHHQHIISRAQELESVMSGPTFVHLFYSLVCISLSIVSVTVLLQTEGYTPKILKITFVIVVFVSQLGFFCILGNNVIEQSERLLVSAYSSYWPGAQPRFQRALLVLMLRARHPLHISVAKLYPLSKETYLQILNASYTLFNLVFQTNGRN

>LmigOR94

MEALWWPVSPVRRGLRLLGLWVAPPGRRALHRLALSWVLASHAFLLLVGAASLVMDTPEDLPQLSFTAYTTLTCFGLIAKLVSFSLDGARLTRLLQLLAECRARFPDPGGRRGQHHLMAVRLHRFLQVSYRVNSVVWMFAPVVSAALAARSGGEEPVKRMYVLPLWLPVDTQASPAYEAVYVAQLATGWMLSETTVLLDVALLALMLHAAAELAVLNDRLRSQPAAAAAGPALASPVAYPKDGDTDPKHDHMYRHMVENIQHHQIIIVYTGLLQSVVRRAISVLLACNTVSICFHIIATVALLQKDIELVGMTKMVVGSTLYAYQTAILCLLGQRITTQSERLSASAYSSAWWEGDGRYQKLVVVFCERASGALSIRVCGLYSLSKETLLQVLKAAYSLFNFMYQAMETSNH

>LmigOR95

MQQADPLWPLCSVVGELGLLGLWLPPPGRRLRHRLVLAFVVASHLGCFFGALASITMDTPSDLPQLSFIAYNCLTDAGLTCKMLSFSLDGCRLTELLRLLSESRRRFPDRAGHRASQHATAVRIHRFLQMMYRINTAYWLLGPVVRNIVAAVSRQPSISVRDIPVPLWLPFDARRSPVYEALYGLVLAFGWAISETSVLVDSSLIALLLQVVAELAVLNDNLASGTPPAGKLTTRVTADAAGSRVQIPLTTAPSVLEGAVCDLVHRGEVYQHILDNIQHHQTIISCVRLLQKVLSRATCVLLFCNTISICFQVIATAVLLQEDGETMQTLKILMGSTLYGYQVALFCLLGQRVINQSERLIRSAFSGDWPEGDVRSWRLVHMLCMSTRQSLSLRICGIYTLSRMTLLQILNVSYSLLNFIYQTKTEQSSGKQEL

>LmigOR96

PGRRLRHRLVLAFVVASHLGCFFGALASITMDTPSDLPQLSFIAYNCLTDAGLTCKMLSFSLDGRRLTELLRLLSESRRRFPDRAGHRARQHATAVRMHRFLQVTYRINTAYWLLAPVVRNIVAVISNKPYSRDIPIPIWLPFDIRSSPAFEILYSLELAFGWAISETTVLVDGSLIAMILQLAAELAVLNDRLAAGTTPAVKQTPSDAATTEEGTLKDSRVRMPTKSSSAFVETTCTDFLQSRSSIGSEDEMYQQMVGYIQHHQTIISCVRLLQKILSRATSVLLFCNTISICFQVIATAVLLQEDGEIVQTLKMLMGSTLYAYQVALFCLLGQRIINQSERLIRSAFCGDWPDGDVRSWRLVYMLCMSTKKSLSLRICGIYTLSRMTLLQILNVSYSLLNFIYQTKTEQSSGKQEL

>LmigOR97

MEEEEASELLGATAVALQLMGLWRGGGGGVAAQAAVAAPTLLVMGSAVLLSGAKLCAEPPAVYEELIAVIFILVASVSWTFKATAFVGQRRRLQALAALLVAGSQNYGDGSGTRAHYRALARRVFIYTQAITAVPIAMWALEPLLSGGQNTPLPAWLPLDLHATPAYELLCTFQAVAVTLSVEASVCLDMFFIVLMIAVAAELHILNDNLESIRLQPVHSLPLKPVDDSAMQSYRNASSLTEKDIPQQYEFYRDSHKSMNAIHGTADAHEVMYCLLVKNIQHHQLILKCIKELETAMTYSIFVLLFLNMVTICTLIISTTVLLQSDSDPTSLYKMVSSLPIVMFQTGLFCIFGQMIIDQSERLPAAAFSSGWLDGDIRLRRALLLLMRRAASPLCIIVGRMYPLSRHTYLQLLNGSYTIFNMMYQVRGRSD

>LmigOR98

ATSRTCLVAGCYLALVAFAGSQLLAEPWRPLEELAIAALIFVTSASFTFKFAMFLRYRPRLLKLATLLCGGGLRPAEGEETRRRYRERGRQVFLYLQAILSVPTLLWAMQPLLVPPGGSAANLRNATAPVRMRSTPLPMWLPPALQQSPAYELVYALQVVSMLIVLQTSVFTGVFFLVLMLSIAAELHVLNDSISGNRSKPSSSSGRRCSSLSMEIHCPSNGRLVNDSSVINFEGRLFRQQMYCDLVKNIRHHQLIIMCVKELEAIMNLPIFVLLFLHMVNICAQIFVTSLLLQKDNDSTTMFKVLFTLPIYLYETGLYCVFGQIIIDQSDRLPTSAYSGAWLQGDARFRRALLLLTSRASRPLTLTVGKTYTLSRHTFLQILNGSYSLFNMLYQVQGNK

>LmigOR99

MNAYVCAMGALLSVKVAAFLWHRERLWQLACQLVSCWRQFEDADGGVRDMYRSQAARVVRYMQVMAAIPAMMWILEPLFSGGDEQSQGRSLPLPTWLPLTLQQSPTYEILYVLQVLIIIIAVAVSVYVNIFFAVLMLSIAAELHVLNNNMMAMGQCRDCEVPVRYEREEGRSRDQVTSSFLRTHRRQRVPKAAANKSLPSPVVVHMHKGSYDRMYHQLVKNIRHHQVILRSVEELQKAMTHSIFVLLFLNIFNICVVIFAGTTLLQKQADQVAMYKMLCSIPIYMYETGFFCVVGQTIIDQGERLSMSAFASTWLDGPRRLHRLLLVFMLRCARPPTITVGKTYTLSKRTFVRILNGSYTMFNMLYQFQRNK

>LmigOR101

PGSTSAAGLCRERVDDLEEMSEGIFICTTVIVSTARMLYFLAYRMRLQRLASLLLEARRCFPVQGAALRSRYQRHAANVCIGFQATAVLPVSLWVLDPLLTAAVTSQNTNNASADAAAEASRLPLSLWLPVDGRQSPSYEAVYAFEGFLVVFTAQVLLFLDMLFIVLIIHITAELNVLNDSVAAIREAVAGGGETGNGTDASGKLDNTYGSVSRSDSDMYGQLVEAIRHHQTVMRYVQELEDFMSQPLYILLFTNMMNMCLHMFTFVVLLQKDIERSSMVKMMLTFPAYLYQTGTYCIFGQTIIDQLLKDTYTMFNMLYTLQGNK

>LmigOR102

MEDAAGQSATLLSPCSVALAWLGIWRPPGGRRSGLGLPGAVFIAALDITISSLALVQMLIDRPEDPADFREVFFICSCGVSWSVKVVAFLLQGDRLERMVLSLLDAKTRFPDNGSRVREKYTAMAYTVWRMWQAMPAVTVLLWMADPLLQTIISPPADNATRPLIFWLPVQVHDSPAYEVTYAVEAFFIGTVSETSILMDIFLIILLVYAAGEIAVLNENVARMGLTMQRETAKAQPVESSSEKASMSKTRYVPSGDSPAADVGAGGKGQLPLLDGDDALWDMYSALVTNIRHHQAIIAYINDLEVVLSTSIYILLLTNALNVCLHSFGLVAFVIIVFLQLLAEGATSSTVFKEVISFASFLAQTALFCFFGQLIIDQADRLQFSAFCCDWPDADESFRRSLRIFMARATCPIKVTVGKLVELSRNTFLQALNASYTIFNMLFNLQTSDE

>LmigOR103

MEALEKGEPTNGELHLINGTFTGPQMAGRSCSSPASYSKYFHDAPVSGHLGFVKTLDWKGFYRSVNHYEASAANGAESAGRQEAVPRQRSRHPGKIRQDCLHRVETVDGESPRTGVTVTVVLWMGDPLLQMLTSPAGNSSRPLIFWVPLEVRHSPAYEITYAVQALGIAAIGQTSILMDIFFVVLLLQAASEIAVLNENIAGMGFKKLRDRENESEEHTSIQRVESSTYVVRTSYVLSECTPHPEISSDNNGNPFAFDRGHKYCKMYSTLVRNIRHHQHIIAYVKDLEVVMSTSLYLLLLANALNVCLHSFGFVALFQEGATRSTVIKEVLSFPSFLGQTALYCFFGQVVIDQADRLHYSAFSCDWPHADEPFRRSLRIFMMQAARPLNVKVGKLVTLSRKTFLQALNTSYTIFNMLFNVERRS

>LmigOR104

MLHVYLGTCFTASPLRQWVSAPACRQVATAAQEVRRLVGPAAPALEWLGLWHPPGSPPTGFKALRGATVLLIDLLLFVFCSLLLLLDPPADAEGQRETFFYIMASFTWTVRGVFFMLERTRHEKLVSILLSLRQRFPDDGCDIRGTHLKNAALLSLAWQVAPVLVLPAWIIEPMLETHYVTYGNITEVYRRTMLYMWTPGDMQQSPNYEISYVSQVVVSLIAVEASVLQDIFFVNIMVQVTSELDVLNANISSMRLPAAAKSQSVANGPEYSDTKLEQYEATYFSHKSKTSAEDELQRSVTIYKNTNDENEELYKKLVKNVRHHQMIMVCIDELEAAMSKSIAVVLVISTLNICVHAFGFVGMFQEDAPRVTVFKRTVAFVIYMTHNALFCFLGQSITDQSERLLHSTFSCGWADADRPFKRSLMIVMRQTSRPLVINVGKFFTLSRNTYMQIVNTSYTIFNMLLSVQ

>LmigOR105

MKNQMHSDEKVWDTTKDVEAMLGPSAVVMSAIGLWQPPGGRPAGAKVLVTSAVLLMMLTIFLGGLVQLVLEPLPLEEILDAVFTCACSFTWGIRVMVIRLRQRRVQQLVVDVLNMRKRFTENAAALRKKYHRRGLMVCLAWVVFPALAVPMWFVEPAMTKKLVTTSENTTMVIRKTPFIMWMPMETQTHPNYEITYVAHLSLLVCSVSPTLIIDLFFACLMTAITADIDILNNNIANMRLYKEDGFTENSEKGVEVTATWETTYQSKKSLAEKSERVDDNTQVLATYSTDPYEQLHRTLAKNIQHHQTLMSIVGDLESIMSESSVLMLIVNSINICLQALGFVDAFRPGAKRSTVLKKVLTFPAYINQTAHFCWLGQAIIDQSERLQDSAFSCGWADADQRFCSSLRIFMLQASRPLKLQIGKIFTLSRNLFLQILNTSYTIFNMMINF

>LmigOR106

AGLGSQLACLTAAKAMKDHVQSDGQVRDTTQEVEELLGFSAVLMRWMGLWQPPGGAPAGPKLLIALSFDAMFFVIVAGGFASVVLDPPPMDSVLEVVLTLASSVTWGVRNVAILVRQNRLQNLILDVLDMKKRFAENGTEFRKAFQRRAKIRTALLVGIPMLGIPMWLVEPAFSKTIVATSENTTVVVRKTPLVMWMPMDTQTHPNYEITYAFQMLLISIVVDANVIIDIFFSCLLITVTADIAVLNNNVANMRLCKDDGFTKESGKEVNLSATWETTYKSKKSLEEKDQLYDGNTDVYASHTTDPSTQLYRTLVKNIQHHQVLMSIVNDLESIMSESSVLMLVVNSINICTQGIGFVDGFRPGSNKTTLMKRFLTFPAYVNQTAHFCWYGQDIIDQSERLLESAFNCDWANADSRFCSTLRIFMLQTSRPLKMQIGKIFTLSRNMFLQILNTSYTIFNMFINF

>LmigOR107

MESEAKSLVGPSGWALRQLGLWRPPGAAPAGPRLLAAFFVVATDALVSTSSAVQLAVDTPTNPETLRDVFFQTTCSGAWAIRTVLFMQQRDRLQRLVMTLLDTRKRYAENVPGTRSSYDRGAAIVFFAWQMLPLTAISLWALEPATVPAEPVLVGNTTVVLRREPLVLWLPIDTQRSPTYEVVFVMQVIGIATVSEVSVLLDIFFVSLMIHVTAEVEVLSGNVLNIHLSTLDGQLTQSREYGDGRLSYKGGGQLAAIDEPTDLSAAGSGYLPNKTLFYSQDMDDAQRRLYACLKTNIQHHQTIIHCVNELEEAMSNSTYLILLVNALTICLHAFGFVELFQGGGKGPAVVKRLLACPIYMGQTALFCVIGQSLIDHSERLLDSAFSCGWPSADRRFCSALLIFMRQASQPLKIRVGKIVTLSRNSFLQIMNVSYTIFNMLLNTQ

>LmigOR108

MACTFSLPTGCTTRGLTAAPCGADGVGGAFAGGPQRVGAAAAGAVAALVSAASVVQLAVDTPTDPETLRDVLFTSTCGLAWASRMVLFMKQGVRLQRLVTTLLVTRKRYAEEFPGIRNSYDRAAAVIFYAWQVLPLTAVSLWALGPVTGAPLAVASGNYTVLERREPLVMWLPVDTQRSPTYELVFAMEMVGVYAVAEISILLDIFLVCLMILVTAEVAVLNRNVSSTRLSRLDQDQPGGVTAIGREGYGGLSADGEWSAAANQTLPNSTLLPYQDTDAAKRRLYDCLKRNIQHHQTIMTRREQRTAFYYLGSLPASHKSVNLFHNLVSSLAGIRFLKYAAIKLVLRPELQMLVFGVLVTIIKTALFCLIGQSLTDNSERLLDSAFSCGWPTADRRFCSALLIFMQQASQPLSIRVGKIVTLSRNSFLQVMNVSYTIFNMLLNTQ

>LmigOR109

VNIAANISLVITSGVELCADKPRQTERASVTAFLFSVSIINFVKAVSLLRHRSRLRRLVRRLVAVRAAFADPAGTRGRYARHAALLASTWLVTAETNVAFWCLDPLISEAAGGAAAERQLPLPLWLPFNQSRPHSYGRLFALEAAVLMSAVQIAILVDALFVTLIINVTAEIHVLNSNIRSMSKAAASGGGLQSGGTIENTTTSDESTLVANLHRSNVRSSNSAISDISASGRNGKSADDEMYGLLVKNIQHHQLIIICVKELEKAVSTGTFALLSINILNLCSHIFSLVVMLEGENSVSAITKMLVAVPVFMCQSGLYCLTGQAIIDESARLSTSAFSCGWPDADQRFKRSLRLFMTRAAQPLHIRVGTLISLSRATFQELLKGSYQLFNVVYQVHTN

>LmigOR110

MLPSADDEKRLLGLMRFLLGRSVASRDGSFRTQLANGATTATLLSTAAANLIIMVCSGLKLYLDPPEETEKASQVAFLMTVSVANAMKGFSMVQQRARLQQVVAGLLAMRRAVCDGSGARHRYACSATLIGNIWMVMSVILGIVWGVDPVFNQPPQLNGTSPDPVLPLPIWLPLDASVPLTYWLMFALEAVVCGWTIFFVMIVDLLYVTLILNFAAELHVLNHNIQITCNAVDVTAHPKRKIGVSRHAGVSLYKGHNDDTAAIPNFSLAHFTAANPLMPAYIPDHFRVELSEDYDTYRLLVKSIQHHQLIVKCVNEFGKATGLPVLMVVSINVVNLCSNIISLAVLVEEDPHASAIAKSLIFTIALGSQTALYCLPGQMIIDQSERLAHSAFCCRWPDAGVRFKRSLLVFMACAGRPLRLRVGKLVTLSRETFQELLKLSYQLFNLVYQLQSS

>LmigOR111

LASRLFSSDDVVEETSSPLKTPLPTWTPAAAQRSPAYEALYLFEALCLTASSQALLCIDVFFIDLMLLVAAELRVLNDNVAAVSAGAARSDSREDTAGHGSGVSTVQQRSREFPDTFSAFDVLVDRRMSEDMYRQLVGNIRHHQMIIECVELLQMTMTYSIFALLFFNMTSICLNIFVTASLLQSDADLVTAMKAVFTTPVFLYESAMYCIFGQMIIDQSEQLPLSAFNCGWPETHTRLQRALLVFMLRSSQPLRIQVGKTYELSKETFVRVLNGSYALFNMLYTFQGNK

>LmigOR112

MGDGCEALGASVSVLRLLGLWVPTESSGAGGKAAYVPGALSCVAIGLLSLSCASKLFIDTPTELTELTVCAYLFVIITANFVKAFCLLLQQGTLHELVTLLVEAKKKNVIDVQHNEDIRSLYGVMSARLYRYLQVMIVVSSVAWLFVTVVFRVITAGSTNIEWPTPLPIWLPLDTQRSPAYELVYVAQVSCAVVTAATMLGADTLFFHLTLMIVAELQVLNDNVSVLGRPAPPSADTRQVVCTMNGAGERQTHPPGGVAERQSDSDGAVSLSDHQGTAAAESKYLVLIEIIQHHQIIIKMVSLLQTIMDYSVSVQLLTNVLDMCFLIFTMSELLHHEKSLHAVLQTILSLPCLLCESGAFCMFGQMIIDQSENLVHAAFSCEWLEADGRFRKPLYTFMLMATHPLQIKLGGTAKLSRSTFLQALNGSYSLINLLYHSRRPVG

>LmigOR113

MDITEEELEAVVPCMARSGLLGYWRSSASREGAGSYLRGFLSCCLITCISLSAAERLLTDTPSDLAELTMTAFELTVPLTVVSKGLFFILQRDTIHELVDLLVDMRRRYAERDDGPNRRRACYLYVLAVQRVLLVMALLIIGGWLAGPMLPHVFSFASQNESSVPWQTPLPLWLPVDLQRSPLYEALYLFQGLCVLTSLTSASALDACFCNMMLMIAAELQVLNDNISSPSGNETVVDKGESESITLEVHSELESVVPQFKSGATGDAGLSKTGRSRPRNQTSLRL

>LmigOR114

ATSAGKLCLDTPQELAAVADYGYAVFHLSAVTVKVACFILQRSTIEELVNLLDETRKTYGKTEANYQVRQLYQRRATNIYRVLQALAVAVLCMWISSPVIQRQGLKEGERPPPNPIWMPDNSPGYEIVYSVQSLCGSAAVQASMLIDTSFYKLTLMVTAELQILNDNLARLGRAAEAADRKGTAAKQDGKEVAVPAIKQTSAPVTEDNDQLLNDQMVDNVRHHQAIIKCFDLLQSVITYSVSIVLLTNILTVCFSIFVVYVLLQSDGGLKSASKTIIGIPSVLGETGMFCIFGQMVINQSERLRSSAYSCGWPDADGRFKRALLILVLRTSQPLQFTVGKLIHLSNETFLQILNCSYTLINVLYQFQGSKE

>LmigOR115

PLLQHKAADEFSGAEKVERQMPIPSWFPFDVQRSPAYEVVYAVQAVCGTAAVQLSMLLDASFYQLALLLTAELRVLNDNLALVGAATSATGGTGVAGRDSHHQHMPAPKLTTSAVTEDNDRSRTLLYFEFVENVRHHQAIMKCFQLLESVLNYSISILLLTNILTMCFSIFFASVMLQADGGLRRAMKITSSIPNLLIETGMFCIFGQMVVDQSERLPQSAYSCSWVDSDARFKRALLIFVLRTSQPLEFTVGKLIKLSRETFLKILNSSYTLISLLYQFQESND

>LmigOR116

EPADDLLDLTWTVFILFGGAIVFVKMVCFVHQRAVMQEAFQLLLACRNCHYGGDSIRTIRGSYQRLGNITYFSIQVMVIVAWMIIVFMPLLNHRISAAGREPTAKEHGPLPIWLPFEVHNSPFYEFTYAFQALWLAFVAETSICVDCIFVNLMLMITAEIHILNSTLSTLQEHSIINKVPVVTTNQSTEDILPFTHSSRIHDSKQLFEKLISIEGTDYSPSDMCDISCAESQKIIKQQIYHQLLQNVQHHQTVIKCTSSAQKAMNFSVFVLLSTNIIEICSSIFGTVELLKNDMPAAAMKTLCVIPIILSQSGMYCFFGQMISDESEKLLQSAYNCDWYEGDIHFQRVLFILMLRATGPLKLKVGKTMSLSRQTFLQVLNGAYALLNMAYHVSK

>LmigOR117

GAGGDSPRLLPLPAWLPLDLQASPTYELVYAAQVLLIPLSTTSVCSDFVFIGLMLRISAELEILNDSISGLHKIRKDVPTAPKNEAKSIGRVSDGEINLHISRNVKHHQAILKSVALLEEAMSTAIFILFLGTMIAISINIFAATAVLQTVGGMTKALKMITAIPPIMFEVGLYCIFGQIVTDQSEKLMHSAYSCGWVDCDPRFRHSLLTFCVGSRRPLEITVGTVYKLSKETFLQVLNASYAMFNMLYGFQSNT

>LmigOR118

MRQTRSVESVLGSSAALLRLLGLWSPRIEDFSRTHRVLRGGLMLALSFGLMVTTLLKLVMDCPRELEELSACIFSATMLCEVFFKMVFFVLKVPTLHKLVQLLSEIRTEDSIGERNDEIRRRYQIVVDKMFLFLMATAVVTETMWAAIPLMHQLLNMDGEVTRLLPLPLWLPLDVYASPTYEVIYGAQVLLMPLTTTSLFFDFVFIDLMMRIAAELEILNYNISSVHENRKTVSTMSKDIHKLCQTVSDNKTNVQLVKNVRHHQAILRAVVLLEEAMNTGVFILFLATTIAVSSNIFTATALLQAHDGRIKALKMLSAMPPVLFEVGLYCVFGQIVINQSEKLMHSAYSCEWVDCDTRFRRSLHMFCVGAIRPLEFTVGRMYKLSRETLLQVLHGSYVMFNMLYTIQNRK

>LmigOR119

MVVFGRGVEAVLGPSAKLLRLLGLWSPQKGDTSHNGSALTGYLTLALIFGLMVTSALKLLMDRPRELDELGACIFIVTMLAEVFFKMLCFVVQRPTLHKLVQLLTEIRADGSTGERNDEIRRGYQILVDRMFLLIMVTATATQTLWAAAPVIYQPLNEDGEVTRLLPLSMWLPLDMNASPNYEVIYLVQVLLMPLASASLLFDFVFIDLMVRIAAELEILDYSFSGLSKNPKSVSATSKNEFKSVHTVSDGEINLQLAKNVKHHQEILRSVDLLEEAMNTGVFIQFLASTIAISCNIFAATSVTLYDSQFKQLIIILLIQSGLYCIFGQVVTDQSEKLMHSAYSCEWVDCDTRFRRSLLTFSVGATRPIEFTVGRMYKLSRETFLQVLQGSYAMFNMLYTFQSNR

>LmigOR120

MLRLLGLYQQKGGGGRVKPKLLSALSLIFLLYHPVFAAMKLYMEPPEDLVEFALCSFSFIISDGVFVKTAIFIADRGMLHQMLQVLSDSRRLYGGEETSKKIRNRYENLAERVLLYMQVSTMLASVGWLAAPLVFRALAMASGDGGEVPRKLPLPVWLPVDVQETPTYEILYVIEAYCVTLTGLVTLCIDVLFIRLMLMVTAELEVLNYNVATMAKRREKISDERRSLEYQQGTESQDFYRGDKALPMASESYEDAMDNELYQQLVTNVRHHLIILRTVDLLEAAMSKSIIILLFINMGALCSNLLVVGVLLQAGEGVTRPLTLTAMIPFLLYQTGMFCVFGQMVTDQSEMLTTSAFSCGWNESDARFRRSLLIFMAMVNRPLEITVGKTCKLSREMFLQVLNGSYTLFNMFYQVHSTS

>LmigOR121

RGRLARLLGGVVRAATMFGLVFMWLGTVLKLCVDPPPQLEQLTLCSLVSSICTGFIIKAAFFLAFGGTLRQTVRLLADTRARFCTGDHNEATRRRYHKQSNNIYYFIQIVAAIAIVGWILCPLVTHILAKTDEDHPEARMQLPVPVWLPGDIHETPVFEMLYAFQSFTITFGAQFCLSIDIFFIHMMLMLAAELEVLNYNLSAMGHVNLPKLGSHGGKSISRYKSSGRQSALLSSGQQLGEQILTEDSGNEWLHQQLVKNVLHHKAILRSVSLLESSMTVSIFGLLFINMANLCSSMFVASKLLQKEGSIGKALNALLTVPSELYETCIYCIYGNVMTDQSERLLESAFHSDWVNGDTRFKRSLIIFMAVTRRPIVITVGKTCKLSKETLLQVLNGTYALLNMLFNIH

>LmigOR122

MPIWSPVDIYSSPTYEFIYLLQSFASLITSQCCLSIDIFFVHMMLMVAAELDVLNYNLSAMKHYDSQTPISDGEEFISNVKTSGRRLELPSSDKSFGEQALKGDIEGNGLHQLLLKNVLHHQAILRSVSLLQSAMNVSIFVLLFINMANLCSSLFVAAVLLQRDGNAAKALHALLCVPALTYETTIYCTYAHIMTDQSERLMYSAFSCGWVNSDARFKRSLVIFMMVTVRPIEITVGKMCTLSKQMLLQVLNGTYALLNMLYHFH

>LmigOR123

MGHSSEERESLEGPGVVLRRLLGLWRPRGRLARGLDMLLAGVTLVAISFLVVCVALKLYADPPEELEQIALCGLVASLCIGFFFKALLFMVLGGTLRQTVRLLEDTRLEFFSGDKNETTRRRYQQLSRNIYNYGQMVAVPAAIGWITCPLLSRLLTHTGDDQHEVKRQFPVPVWFPVDVYTSPTFELLYAVQSFCVLVVAECCIATDIFFVHTMLMVAAELEVLNSNLCAMGDAKLQMKRVKEEEAVSRYKTKGRRWAFLNSDQPVGEHGLPENAVHEWLHEQLVKNVRHHQAILRSVSLLQSTMDVSIFILLFVNMANLCASLFVAGVLVHKEGNVGRALNALVSIPALLYETTMYCIFGHVMTDHSERLMYSAFSSGWINSDARFKRSMLIFMMVTMQPMNITVGKTYTLSKQMLLQVLNGTYGLLNMLYHMHGSE

>LmigOR124

MGLWQPRGRAAQRVNALLASLTLGSLCFMALCVTLKLCADTPQEIEQLTLCTLVASICVGFICKTALFVIQGDTLQQTVRLLEDTSEQFCTGDHNRLTRRRYLRLSNNVYYYCQMVAVPAAILTNTDDEEQQLPWQLPLPAWFPGDIYETPHFQILYVVHSFCVLVAVQSCLSIDIFFVHMMLMVAAELDVLNCNLAAMDHITVQTTRNEEERFIPRYKRNGRRLPLLNSGQSLAEQTLSQDTAHKDLNQQLLNNVLHHQAILRSVSLLQSAMNVSTFTLLFVNMANLCSSLFVAGVLLQKEGNVGKALNALFSIPALLYETIIYCIYGHIMTDQSERLVYSAFSSGWVNSDPGFKRSMLIFMMVTVRPMAITVGKTCRLSKQMLLQVLNGTYALLNMLYHVHRSE

>LmigOR125

WRPRGGRAARLLNGLLTAFILASHAFLPVCVALKLYVDPPEELEQITLCSLVTSICMGFLFKAALFVAQGETLRQTVRLLADIRAQFGDRQQNHSTRRRYRRLSDSVYRRYQMVAVPAVIGWVLCPMLSRSVRGSDQAPQVAQRQLPVPVWFPVDVYASPTYEFLYVAHSFCALVAAESSVCVDIFFIHMMLMVAAELDVLNDNLTVMEDVNLYATPNERRGLTSVNGSTGRQSAIHDSSQSTLGENAAREGIHEQLSKNVQHHQAILRSVSLLQSAMNVSIFSLLFFNMANLCSSLFVAAVLVQRDGNVGKALNALTSIPALLYETRMYCIYGHIMTEQRARC

>LmigOR126

MEFESMMGPGLPLMRLTGLWQMGRQGGGVSRGLRLATIVLSVLLVVAGSTLHLVFDTPDQFEDITLCGFNIDIVSLDLLKGVLFVVQGAPLRELVQLLCDARAGFTFADINHAIRGRYEAVADRMRILLQATVVLPLVGWLSAPLMSRLAAGAGGSRAPRQLPVPAWLPVDIHATPTYELLYALQAFGCTAAGAFSICVDAFFIRLMLLISAEIEVLCENISAIGVPHPAQGSGGCICRCQPNAADLACTCKGCVKAFTSSPEEASDEMYQLLVKAVRHHQTIIRMVALLQQTMDALVFIVLFANMANLCCSLFATAILLQRGGSLTKTLKGLSAVPVVLYQTSLYCLFGHIVTDQSEKLYNAAISCGWVNCDARFKRSLLIFMVEAMKPLEITVGKFCKLSRQMLLQVFHSSYALMNLLYYYHYNTE

>LmigOR127

MGERGEAPAGLLGPEAAVLRLLGLWRPRERQGQGLTPPAVVAAATIAAVAFIPAGVVLRLCGDFPEEIEETAHCSYICIVCFGCIVKAVLFVMEGDTVRELVHLLQATRAEYGSDEGSDRIRSGYQGTVDRMYRYFQVMALLPTLYWICWPLVAAAVSPGEASGVSGARQLPLPFWLPSGASGTPTYHLLYAVQALSLSLTVASAVCLDVFFIRLMMMLAAELQVLNENIAAIDGCRASGSAYGREEEEFDSLVPSDDRALEPTKKSAANFSDDDLFSRLLNNILHHQAILRCIWLLQTAMNVSIFILLFINMANLCFNMFVTAGLLQDGRNVTKAVTAFSPVPGLLLQTAMYCLFGQITTDQSEKLLHSAFGCGWDDCDTRFKHNLLIFMLMVGRPVEITVGKTYKLSKEMLLQVLNGTYVLLNMLFHVHSDDHI

>LmigOR128

PGAVADWQFPVPHWVPVDMQRSPTYHLLYVLQSFCLLVASQSTIAVDLFFIHMMLMLAAEIEVLSENVSAMGKIDSGLMALENEDCGLSTKDLLSYRDGNGLVSEIFQKEYISEDQMRALLVKNVQHHQTILQAVGLLQDAMDISIVILLFTNMADLCSCMFASAILLQRGGNAAKALKPLMTIPPCFYETAIYCFFGNIVTEKSEQLVTAAWSCGWPLCGSGFRRGLLLFLTEAARPVEITVGKTFKLSKQMLLQVLNGTYALLNMLYHVHRSE

>LmigOR129

MSMPDSELRTLLGSGASIRQLMGLWWPRGRRGRGRACSAAAAAVSLASLAWLPTFSGLKLLIDPPPEIEEIAMCYLLIFACTGFFSKAAFLIYKGETVWKLLDLLSETRRLHRNGESNDNIRLSYQQQSRRVYLYMQGAICVAFVFWVSTPLLVRAFLASDEDSPESYRLFPVPLWFPGNMYLSPTYEILYSVQSFSVLVAAQSTVCVDIFFFHLMLMISAEVQVLNENIALMEKVNLKSEKQEDHELRLNIKEKAEDLSFRSVGYSTGKALTEEVSDENMCVQLVKNIQHHQLILRSVVLLQDIMNLSVFILLFVNMVDLCSCIFVGAVLLQRDGNVTKALKPLSTVPPLLYETGMYCIFGQILSDQSEKLTDSAISCGWVDCNDRFKRDFMFLLISAKKPLEITVGKTSKLSKQMLVQVLNGSYGLLNLLYHFQSIQ

>LmigOR130

MKPVGGGCGGLLGPGLTIRRLMGLWWPQGGRGRIAAAAAATLTVASLTMLVAFPALKLIMDTPSELEEITLCCFVIFLCSGFIIKSALFIYQGDILKELLQLFSDNRRIYSNDRNSEGIRQSYIKLSERVYVYMQVSVLPAVAGWVSAPMLARFFLTAESTQQFPVPLWFPGDIYQTPTYEILYAVQSFCVLVTGQCTVVIDVFFIHLMLMVAAELHVLNENISLMQKLNVKTRVSEAEEWQFRIRRNDEELTFPIHDHRARVGYFCSEDVSDENMCLKLVKNIQHHQQILRSVLLLKSVMNVSIFILLLLNMADLCSCMFITAVLLQRGGDVTKALKPLLTIPPLLYETGMYCFFGQILTDQSENLIDSAFSSGWVDCDSRFKRDLLIFLMAANRPLEVTVGKISKLSKQMLVQVLNGTYGLLNLLYHFHGSQ

>LmigOR131

MGRASAEVSNSRPLLGPGAAQLRAMGLWRPGRSLLHSLAAALMLACLAWVSATAALRLLIHPPAELEEVALCSFIATICSGFTIKHSERIIVAKVDRIPTPTTVVQVYMSTSTADDEDIEEMYEEIKEIQKVKGDENLIVMGDWNSVVGKGCVVPALIGWTFFPLVSRALNDSGEESPGAVADWQFPVPHWVPVDMQRSPTYHLLYVLQSFCLLVASQSTIAVDLFFIHMMLMVAAEIEVLSENVSAMGKIDSGLVALDDEDCGPSTKYLLSYRDGNGRISEISRKDDISEDQVRALLVKNVQHHQTILQIGEKIQFCRISSKRRHRLVGHVLRLEGIVNLFLKGSGVGRRYFPCLIDLTYQVEHFCSRLALLMISVLLQFYSRGFVSTQILQCSVKFLSHANSKERNVDHGDEGFVSHVVFHVRRALLLFLTEAVRPVEITVGKTFKLSKQMLLQVLNGSYALLNLLYSIR

>LmigOR132

PAAPRQLPVHVWLPADLNRSPTYEALFAAQSFSLMVLSQATVCMDIFFVHLMLLVAAELEVLNENLSAMERGRLQHGRSEYSETTDIHGEDSDRSTFTNTDRRLDVTVGTSQREHDERMYAELVKNVRHHQAVLRSVSLLQKAMDASIFILLFINMANLCGAVFVAAVLLQRDGNITKALKEVMLIPCVLYETGMYCLCGHMIISQSERLVTSAFRCGWPDCDRRFKSSLLIFMMAAIRPLEITVGKMCKLSKQMLLQVLNGSYALLNMLYHFHHTL

>LmigOR133

MDAVVGPLLPLMRLLGLWPCSGGSGRLPAAARCALTQLPVALMVAGSALKLCVDTPDQFEDVALCAFITNVVAAILVKAVMLVARGQRLRRLARLLADARARFPAHRSCTRGRYQALADRMERLFQVGGLVPLACWLSAPLVPQLTAAPGQGRGRPRQLPVPTWLPADLAASPTYQLVYTLQVLGCIGACASTVCADSLFVRLMLLIAAELQVLKENISSLRKTDSVRGGGYACRCRETVSFLASACKDCHDIVTPLSEKTTDEMHQLLVKIIRHHHMIMRMVSLLQEVMDVSIFILLFANMVNLCSSLFTAAILLQGGGSVVKVLKGLSPLPVVLYQTSLFCVFGHIITDKSGELTDAAVSCQWVDCDTRFKRSLLILMTVALKPLKITVGRVCTLSREMLLQVFHGSYALMNMFYYYHHKTK

>LmigOR134

ELQVLNDNLASVKAAPLLRSSPYSGWNRATNVTNESWFHSGVSSPTGAIACNSYQSSGDRSESTTSITGYTNRTAVEMYRALANSIKHHQAIIRCVEELESAMTYSIFVLLFLNMMNICVHIFVTSVLLQKEVERTTMSKMLCTLPIYMYETGLYCVFGQTIIDQHGCDVLQSEQLTASAFSGDWPEGDARMRKALLLLMLRASRPLQLTVGKMYVLSRHTFLQILNGSYTLFNMLYQVQKNK

>LmigOR135

AFMMPLWLPLDTQASPTYELLLGVQVPCCWICSETSVLLDCAMLALMLQAAAELAVLNDRLSAVGPGQRRAADDKRDLQANDHMFSGLVDNINHHQIIITYMHLLETLLSRGISVLLICNTISICFHIVATVALLQEDIEPVGMTKMVLGSTLYAYQTAILCLLGQRITTQSERLPVSAFSCDWPSADGRFRKLLMVFCLRSSQALIIRVCGLYSLSRETLLQVLKAAYTLFNFVYQTVGEEEPLN

>LmigOR136

RLPFAQHLWDDNGHWYGLSYAVQCVTGLWMAEVSFGVDCLFATVLMLAAAQLRVLALRLVRLKVDAGGAPGDQPGATDGAYRELCLCVESHQEILRFITHLNGTMSPVAMTQFVFSVLVACVALFQATYSTDITAVIKCVSFLPIPGGQVYLYCWAAHHVTEQAEAVSTAAYCSPWVDAGPRFKRALRILISRAQKPLVLTAGRLYPVNRETFLSLVNASYTYYALLGQMNKRSAN

>LmigOR137

SKAAALLDGAEELVCPNATFLRLMGLWRARGWTGAARGWFCWTLVLFVTATSAGKLCLDTPQELAAVADYGYAVFHLSAVTVKVACFILQRSTIEELVNLLDETRKTYGKTEANYQVRQIYQRRATNIYRVLQALAVAVICMWISSLVIQRQGLKEGERPPPNPIWMPDNSPGYEIVYSVQSLCGSAAVQASMLIDTSFYKLTLMVTAELQILNDNLATLGRAAEA

>LmigOR139

AEFEVLSDNFAALHKVHEQSEKGKIRGTVYERQIKEESPPASCAHIYIECVSDEQMYRQLTQNVRHHQALLRSISLLQTAMSAPIYLVLFVNMVNLCTNLFIATLLLQRDGSFSKALAVLLTIPTLLSQTAIYCLFGHALTEKSEKLTQSAFSSGWPECDVRFRRGLLMVMTLAEQPAEVTVGKMTKLSKQTLLQVLNGTYGLLNMFYQLHSQM

>LmigOR140

LLDISSPLDGLVGGRTRQALLLLDQAAVAFNHLCSVAAFTTMFVHFVVIACRHLQRSIDDLTADNCDIAAVVRHHQQILRFIREIEEAYCILMFWLFLPMMVVMCLIMFAFLTMTSLDIEFLEMLAFFLIYCVTNGVISICGSMLTSKAERVMVAAYSSAWPERSRGFSGAVRVVMVRFLQPAQLTVGKFVPLSINTFSKLLQESFSYLMVMLSLVNEKDSEAQPGVVVEATANHSAYH

>LmigOR141

RQSVTVAERLPLAVYAYIALLCLIWFPMPLLVEPHNPKLPFIQLHYWIDHTRFPVYEASYLMQVVSSFFFIFISTGMDCFFAVVMIHVTVQLKLLIYRITEIRLRDAPAVAAESEARWNRDVGDAHAEMYKELCLCIESHQKILGFVKYLESVMNPIALTQFIFSVLAACVTLFQETYNPDISAVFKCASYLPTPGAQVFLYCWGAHS

>LmigORCO

MQKPHGLVADLWPLIRMVQYSGHWMLEYSGGKALRAIYSSAVSLLVVTQFALMAVNLIQRSGDVNELAANTITVLFFLHPVTKFGYFAVRSKAFYRTLATWNQSNSHPLFAESQARFHQLSVVRMRRLVMYVVSVTALSVVSWTSITFMGDSTREVTDPDNANETITEEVPRLMISTWYPFDASSGMGYMLAFVYQLYWLTATLMHSNLMDVMFCCWLIYACEQLVHLKEIMKPLMELSATLDTVVPHTSELFRAASTLPTNEPLYDAGNGAADGLTIRGIYSSQRDFSGFNRRSAALSTVREADAGGAVSSAGGIGPNGLSKRQEMLVRSAIKYWVERHKHVVRFVGNIGDAYGAALLLHMLTTTVTLTLLAYQATKIDSVDVYAASVLGYLFYTLGQVFLFCVFGNRLIEESSSVMEAAYSCHWYDGSEEAKTFVQIVCQQCQKSLMISGAKFFTVSLDLFASVLGAVVTYFMVLVQLK

**File S2.** Amino acid sequences of 120 IRs of *Ceracris kiangsu* and other insects used in phylogenetic analyses.

>CkiaIR1

MDNRMIRLLSAVMLMRSTDSALQMKYDEAPFDAILSEMTVDILTGYFHNFTCVASMSDAADPLLDVVAPLATTARVTASGPGDAMLVSALDADCLGVVVRRLDAAAAVAALLKASRRATSRASSRLLVLPTDSLHPVHVESLFALTDIKLAPDTLVASLSDDGVSFELVTLRFTGRRSWRDEVLVARWVRGRGVVPPTADLFPDKLSDLRGRELAFATFHYPPYAVLEDSTGTQDGMETRILLEFAKKVNASWRLTVDAEHEWGEIWDNNGSGNGILGAVVTDAADAGYGALYQWLHEYLFLDFSRPYIRTGITCLAPRPRPLPGWQVPLLPFSQRLWAAVGASVLVATAALFAASSSSDHLLLGEDAVRAAAGSRYATVADCFFRSVGLLVLQTPDVERRHTRVVGPTRHVLSWLLVAYLLVTASYGSGLSSVLTVPKYEPPIDSVRDLYESGIEWAATHEAWVFSLREATQPVITDLYRRFRVHKEDELHASTVASDLAYSMERLPAGYFAIGNYIDEEAASRWLRPMREDIYWERSNVVVRKGWPHLRHLDALMDRLLDSGLLLAWEGQVSRQWMAPRVQLAVQTGISSSSSHAGDGPTKLRLGHVQGEFALLALGLLLSVVAFLAELLVHSVASSNVVTVYPPSERIGPGPKSRGSNGPGDKVWA

>CkiaIR2

MAGDPPVLSPAACLVVATACCLLGARASNNPTYSVRIAGIFEESDPPALRHAFLSAVEAVNRQTTMEFVLGNAGHRPLIRAHQEVIDSADSFGAKRKACSLVEQGIAGVVGPRQSSSTDVVRSMCDRLEIPQIIVNWDPYPPPIASYQFNLHPHADFIAQALVDVLHELKWRSYTVLFHNDESLVRLRGVLQEREPSDPPVAVRQLDEDGDNSPLLKEIKQAAESRLVLDCDTDMILPVLQQAADIKLAEVYQSYLLTSLDAHTLDFTQFRLAGTNITILSLIQPESEAVMKTMQDFEYPGTEVPSTMTPQTMRTETALMYDAVRVMAEALFKTSTMDWVQTQPLNCSDDSAVWDRGVSVREFISDMRHEGISGEIKFDAEGRRMGFQLEVLELSLNGFKSVGTWTPEEGFLGTSSETQTLEETQNIIQGRHLIVSSSLGPPYLSEKPQTTPPRIGNDRYEGYSMDLISQIADLLNFTFEFKLAPDGQYGSSDEKTGTWNGLVGELLAGSADLAICDLTITQESQSAVDFTMPFMTLGISILYKQPEKADPNLFSFLDPFTMDVWIYMATAYLGVSIMFFMLASMAPGEWDKGHPCDPDPTELENTFNMINIFWFSTGSLMGQGCDLLPKAVSTRLMAGMWWFFTLIMIASYTANLAAFLTNSKLEAPIEGVEDLAKQTKIKYGTYAGGSTAAFFSNSNDSLYQRMWLVMKQARPDVFTAGNQEGVERVKKEKGNYAFFMESTSMEYQTALDCNLRKVGGLLDSKGYGIALQRDSPFRTAVSGAVLTLQERGNLSALKTRWWKAPEGQECAEDDGAAVDSNELGILNVGGVFFVLAFGTLGAFFVAILELLWNCRKIAVEEKMTPWEALKSELKFVVQCSNDEKPVRKPPDADGDKEEEASQLQYGSLSADKW

>CkiaIR3

MTALCNTSVMMAVLILCLTSTIFCESQKDHSNNINFMLDFIRHMSAASPIKGVHAFVCWDTGDLQLMKALSRNGVPASVYRGWQHWKQLPTFHMDGNSLLFILDLKCGKSVALLKKAGEIGEFFSPPHTWLILHDVYPAGTLHHMAPTSDTMLPVPAPTNNGSNLIMQHHSALSTYNNTLTPNAMELGDEEYNTADVYDISPSSSAGYTNGNSIHMTQTLPRSSKNNAEFKSEHTNDDAPMGEIVDVCFYCEVFRDLNILVDSNVVVGSREADTKYTLLEAYRRSKLGELVVSELGYWESSTGMVWQAVHEVALSSLDLKRTKLVASIVVTNPETLDHLDDLHNSHIDTVTKLNYLLLLHAADILNASLELVVTDEWGYESNGSWSGLVGSLQRGEADVGGTALFVTADRMRLIDYIALTTPSVVAFVFRQPPLSLVSNLFTLPFSRAVWACAAALVVTCAGLLMAATRWEWRRGDPQLASYLQQFQAAAAQLSDKWGDVAMLAVGAVCQQGSPAESRGVPGRIVTLSLLVMVMFLYTSYSASIVVLLQSTTTSMQTLADLLYSPLGLGVHDIVYNSHFFPAADDPVRRALYRQKVAPPGGEPRFMTLEEGVRRMRTEPFAFHSELSPTWQLVQETFREEDKCGLQAIPFLQLMHPYIAVQKNTCYKEMFKIAYRRIWERGLQHRQLSRLYTMRKPRCAAGRGTSFVTVGIADCYPALLVPVYGVAIAVLVVLLEMLFHRSKGKKSPTLTTMNMSSNEGHKSSSFSVRTLQ

>CkiaIR4

MNAPAALLMLLLVCQESVGQEIYFNDVISLTRDYFVAKSASLVTVYTCWNSRLEKKLVRALCDRGLRASRLPLLDAAEPSSGGGHAAALASLEAAGPYRSGVLVDVACPGGELLLTKASARRMFNIQRHWLLVDSSGNTTADEASEFPALPASWRHLLSELWMMPDSEVVWMGAMDGGEMQLLDIYRLTNFTPVLNISLAGWAVSRDSEVSLYLLPRPDTSKRRNSLHGAKLKAGAAILFPKYFTGMYDLRLPHLDTWTKITYPLIEYLGQNFNFTMEVYYTDSYGWQTNGTFDGVIGMMQREEIQMAASSLFMSRDSMPHVDFAAEAFYLKTAVVFRQPTLASVANIFTLPFSAAVWGCCVLLCALTLLLFGVQLRLAAASGVEGELTHVTCPELFTFVLGSICQQGLPQTPTSLSGRVTVFVLALTSLFLFTSYSANIVALLQSPSHSIRTVSDLASSPLTLGVQDIAYNKVYLGETTDRELRQFVRRKMQPQGNRVFCNGAEGMERVRSGMFGFQVDTSTAYKIISETYTEREKCGLMEVNLFPLPPLCVATTKHAGYREMFSQSVGWQREVGILTRQRRLWLPQRPVCENMVSGFVSVGIMDFYPALLVLQYGVAGAVAVLALELLCFHSSSLWKHMMCISPLSAAKPTAAYSVTAENRSQLQRTGSRLK

>CkiaIR5

MMVLPLMVPLLLLGAKSWASAAPLESEVLALAVDVLRFSSASTLVAYTCNHYDSVRLSRLLVGERCAWVVLSPAVGTTPLGGSVFTFLHYDCPRNRRLLHVSGQLELVNTSSHWLVWSSGPPEASLPLRLDSDVMWARRQGGGVLLEAVYRVHPSRPLSVRPAGRWSPAAGIVLHRSAIDNGWSLLDGLMLGAAGVVVDTPPDSLGERLRVVQDRHLDTNIRFGWDLCDKLSFMFNFSVVLYKTESYGYLTPNGSIDGQAGMLVDGTIDLALSQLMMSHHRLDFIDFTAPSRMWTLKMVFRHPASRALYGTIFRPFSASLWLGAGLVFLLVLVAARLGCWVAAHNPADDSWSAAFLLVSSAISQQGTTLDTERPSWRMVVFLSFSCALLMDTYYTAAIVTSLLLPPPSTINTKADLVHSELAVGMENISYTHEYFEKSSDPVDHLLMTYKVWPRGTRRPNYHSLEAGVRKVATEAFAFTGEDVSLYPLLDRYVTEADKCSLVALDFLKSRSTYMPVRKNSPYSELVTMGLRKLLERGHVARMRSVWHARPPACLRESEFATVELVTLAPAFMLLAAALLLAALLLALELRQAASNRKQSFLGTGQSGHRIEGLVVSAPRISLWKPLPVSAAGPSTEPQLFTYYD

>CkiaIR6

MKNAQPALLLLLLPCACWALLDGATIDMPIDYFTYKRTRVVVTYTCTEYEAARLWQRLLGCDGAPWILSAPPPARLPRGSVAAIFLDYGCARNSNLFLQCAELGLLNTSTHWLVWSGGPPPADLPLRLDSDVTWAYPREDDVALEKLVLEQPEDSLLSLPVGSWSQRAGLTYQASSVQWPPRQLNGRMLRLAASVIYTPLDNLGGSLRKVEDKHLDMVARFGWEACSRLADMLNFSIVLYSTPSFGFAVVDNKTMDGVVGLLANGTVDVGVTALVITHARLKVIDFTGPGVLWATKIAFRHPKVVGVYGTLFRPFTAPLWVASGLLYALVLVAAWLAYRAVCRWEGGESSSWGAALLFLCSAISQQGVSQSCGLLGWRVLLVVSLWCAALLDTHYSAAIVTSLLLPPPSIINSKRDLVHSPLAFGSENVTYVHGYFESSDDPMDRALCASRLFGRGASSPNYFPPEVGVRKMA

>CkiaIR7

MSAPTRGLLAAVCLLAPQLCSARLDPQAASMASDYFSHSNAHTVVTYTCSEDDSSRLAHLLLASHRVWLMLSPDPEHMPYNSYIFLDYSCEQNQILLTQCAKAEMMNTSSRWLVVSTQEPDERLQVRVDSDVTWAAWAGGGGAVLWGLVRLHPGWPLQRWPVGRWAPSAGLHYDNTSLQWVAKKNLHSLALGAALPIVDTPLDNLSERLKNVNDRQLDTMARFGWSLSTTLAELLNFSIMLYRVRNFGSLMNDYEMDGAVALIHNGTVEFGAAGFIMTTSRMDFMDYTGPGRLWAPEMMFRHPKSASVLTTIFKPYTAELWVSSGGLFVLILVVSRIFCWVEHEVTGTVDEMDNSWNSTFLLVSSAIGQQGVSRSSEWLSWRMLLFVSFLCTNLLDTHYAAGIVTSLLMPPPSTINNKKDLADSSLGFGLENVSYTYQFFVKSDDPVDRALCSRKLYPPGGRPNFFSAEVGVRKMASQPFAFHAEDVRVGPLIDRFFSDDDKCALVFIPLLTPVATYHTVRKNSPVKELFNFGLRMMWERGHVQYLRKSWYLTRVRCLSETEYASVDLVPMSPAFMLLGCAFLLSGLLLLIEVRQYRRSQIEVVPVVANLSSENLTKKLGNSQTGQQKVHFSSTTQIKLLPSLDKGIYWDQFEPSKW

>CkiaIR8

MSSLRLITWIGTRHAGGIVQAESSWGAVVVFAACAIGLQGTTERSQWLSWRVVLLFTFLLSLMLNTYYGAAVVGSLLVPPPKTIRTLRHLIDSPMHVGIEDIGYNRDYLEKSTDPLVRELFVRKVFPPSAKRPNYFPIDVGVERMRTSLFAFQAEAVTMYPLILRTFSERAKCALSEIVLVPHGMTYLAVPKGSPLREHMAYGLRKVWESGLLNYQERIWHPPRPPCLQAGEAESIRIMSVAPAFLIAISGVPLACIIACIEQISALRERREKRLLAKKQRRKRAQSSRSSLMTLTTHHL?

>CkiaIR9

FSPYEWDNPHPCNDEPDVLENQFSLLNSLWFTIGSLMQQGSDMAPKAVSTRMVAGMWWFFTLIMMSSYTANLAAFLTVERMDSPIESAEDLAKQTKIKYGALSGGSTAAFFSDSNFSTYQSMWSFMESARPSVFTSSNVEGVDRVVKGKGSYAFLMESTSMEYVIERNCELTQVGGLLDSKGYGIAMPPNSPYRTAMSGAILKLQEEGKLHMLKTRWWKEKRGGGTCRDDTSKSSSTANELGLANVGGVFVVLMGGMGVACVIAVCEFVWKSRKVAVEESVSNILQDT

>CkiaIR10

IMLFVMGSISPYEWTNPYPCIEEPETLENQFTLSNSLWFTIGSLMQQGTEMAPIAVSTSMVAGMWWFFTLIMVSSYTANLAAFLTVESMYQPMKNVKDLADQNTIKYGAKGGGSTLNFFSDSEDEMYRRMYNTMINTPGVLTKDNDEGVEKVKNSNYAFFMESTSIEYVTERNCDLTQVGDKLDNKGYGMAMSKNMTYRNALSTAVLKLQETG

>CkiaIR8a

MWPLWVSVVAAQLQLASSQAASDPLLISFLVVTEVNASWVGGELRANLSGLEASYAGLRLQLDLSAIEVDREHEVEEFQQKVCGALATGVSALLDATWTGWRRLRDEAQHRGLPYLRLDATLANLVDAVDKYLQAREASDAALMFHTEEELDQALYHLIGNSVLRVIALNGLEADTVNRLKDMRPVASYFVIFADTAHMTQLYNKAAAGGLVRKAERWALVFTDWEWRSFKSDQLNLSTALLQMKASSCCALQGEPESCKCSLRKVAPAFLRAALSAVVESLAELHSKGLDVRAAPSQCSAADAGADDDAEGGADAEDATEPPANATGNYDAFLSAVAPRGQTNSTLFFRSAAAQLTFNMPLQLRMVNRSEDVSLGDWSPEKGLQLNTQLKPAKSFFRVGTAEGVPWSFPVRDEKTGEPLVGPEGEPIWDGYCIDLLKKLAEPTHMNFDYELVPAKNNDFGSRSPSGTWTGLVGDLAMGETDMIIAPLTMTSESEEVIDFVAPYFDQSGISIVIRKPVRETSLFKFMTVLRLEVWLSIVGALTVTGIMIWLLDKYSPYSAQNNKEMYPYPCSEFTLKESFWFALTSFTPQGGGEAPKALSGSTLVAAYWLFVVLMLATFTANLAAFLTVESMKSPVQSLEQLARQSRINYTVVKNSDTHEYFRNMKNAEDVLYNVWKDITLNSSSDQSSYSVWDYPIKEQYGHILQAIEQAGPVRNASVGFQKVIDQEEGKFAFIHDAAQMSYEVSKNCNLTEVGEMFAEQPYAIAVQQGSHLQEEISSQILDLQKDRYFESLTAKFWNNSAKGTCPNSDDSEGITLESLGGVFMATLFGLALAMITLAGEMFYYKSKKLTAVNVTSSSANVPKKQVTMGKEFRPVMEKTAPRVSYISVFPSNQLYW

>CkiaIR25a

MVLLLLMMSLLSNLFSALGQPTINYLHVNDDSNLIPNKALKQVVSDLSKQSMNFDGVFKATANGSDVEALMDSMCLGYNTSIDQNKKIHVVLDTTLQDVSSEAVKYFTSALELPTVSASCGQEGDLRYWSNMDKKQEKYLMQVLPPMDTMPEFIRSFCSEQNLTNAGILFDDTFIMDHKYKSLLQNVPTSHMINEIKFQNIAKQLSTFKQREVFNYFILGSMDTVNKVLEAAADMEFYGRQFGWYAVTQDEGNPSCQKCGKGASVLHVKPNDAEGTVLGAENPKLTYQFYYELFSNTFLAIGQMMDEGSWPDMQYIPCEEYEENENIPPVSKLNLLDALQQISMQNPGAYGQMMLSSNGHSHMQFNMTAFNVSLSDNSATEVGTWAADLDSPFITNVKPSVPVTQYTVVVALQQPFVMKYQDENGNTEFKGYCIDLINAIRNITNFEIEIYEVADGKFGNMDEEGSWNGMIKDLMDKKAHMALGALSVMAERENVVDFTVPYYDLVGITMLMKKPKTPTSLFKFLTVLENDVWLCMLAAYFFTSFLMWVFDRWSPYSYQNNREKYKDDEEKREFDLKECLWFCMTSLTPQGGGEAPKNLSGSLVAATWWLFGFIMIASYTANLAAFLTVSRLDTPVESLDDLSKQYKIQYAPIANSSAHVYFQRMAAIENRFYEMWKDMSLNDSLSEVESAKLAVWDYPVSDKYTKILQAMTEAGFPATIEEALESVSASKSSSEGFAFMGDATDMRYQVLTNCDLQMVGEEFSRKPYAMAVQQGSPLKDQFNNAILQLLNKSKLEKLKEQWWSQNPEKSNDCEKQDDQSDGISMQNIGGVFIVIFVGIGLACITLAFEYWWYKLSPQHNAVVEAAPPSTKSDSLQALNMMRSSFDKRYGRRQGVALAGVTNPW

>CkiaIR76b

MQVSPVLKMVVTTVCSNYFLNGSKMEVPEGEPDPGCVLRMPKLMEGKTISMGTLENPPLTLTNKTDGTLIGQGVIFEMVDILQHKLGFNYEVVKPEANVLGDENHGIIGLVHKKQVDLAVGYLPQFSQHAKLVQHSESLAEAPWVFLMKRPLVSASGTGLLAPFDATVWYLVLASVVLMGPAIYLIILVRVRLCAGSERLTRIFPLSSCVWFVYGALMKQGSTLMPVTDSSRMLFATWWIFITLLTSFYTANLTAFLTLSRFTLQITNLKDIATKKAHWAAQKGSAMEYLVYNNDEYSFLNQSLQAGFGQFVDMSDADMLLSMSKDDLVYLSEKQHVEHTMFSDYLEKTRNPKVEEKDRCTFVMTKQPFLHLPISFYYPLNSSLAHMFDPLLKALVETGMVSHLLRKDLPQIEICPLDLGSKERQLRNSDLYMTYMIVVTGFCAATVAFFGEMLTRQVKRCIAEAELQTGPSASYPDDWKNVKAHANSMP

>AlinIR8a

MRTCLWFHVVLFVAHEVAGQGVKLLVVKDNNAGIWDSVSTSFFEQLPVTVDKEDTNSTINDLCEVLKEGVWGVLDLTWSGLDEIKAVCNTWGLPYVRLEYGITQYLRGADKSLATIRKAPDAALIFQTEEQLDQSLFYLIRESSMRVILFKGLSDKEAETLTTMRPTPNFNIIFADTPSMNVMFLKAVERNLVRYDDRWILVFLDNEHNSFDRKTLVKRVTLATPTIDANAAANFAENVAETLEEVAKTSGIDLSPVPAQCEGSSTAAKDLTVFQEKLSEIVEKKPWLDWRQQESTMALHLDMDWTAESSKGEKLFIGSWNSKKGLTIAGNVTKIPRFFRVATGYMVPFAYPVIDPSTGAPKLDDKGNEVWEGYCIDLINRLAEDMDFDYELTTSYNFGRKLPNGSWDGLIGDLASGRVDIIVAALTMTSEREEVIDFVAPYFEQTGFSIVIRKPLRKTSLFKFMTVLRVEVWFSILAALCLTAFMIWFLDKYSPYSARNNKDKYPYPTREFTLRESFWFAVTSFTPQGGGEAPKSLSARTLVAAYWLFVVLMLATFTANLAAFLTVERMQSPVQSLKQLARQSRINYTVVQDSDAHSYFRNMKFAEETLYRVWKEITLNASANQSQYRVWDYPIKEQYGHILISMEKTGTVNSTEEGFQKVRENEDAEFALIHDALEIKYEVYRDCNLTEIGEPFAEQPYSIAVQQGSHLNEEISRRILDLQKDRYFESLSGKYWNSTMKGKCDSSDEDEGITLESLGGVFIATLFGLVLAMLTLGIEIVYERKAKKNVIKVKSAKPEKSEKSEKKEKMMNNPFFNDDKLFSREFGSFPKKPSKLLAPKPKVSFITVFPRDQLY

>AlinIR21a

MKLEVLFVTTLVHLTHSVKITKLLNSMAFADLETISCMAPDGIPYMVPLINSIAKRYLKDHATVILYDDYFYYHPRLKNMIDILISNYAYPLRHGLVNTTMAKPTVPAGILEARENEQMAFIVFTKESEIGAEAIREFTGHNTMTLLIAQTSVYHVKLFLQTKLAADITNLLVFVDPMIKIDHFVQKTARVLKECDILIFSHKVITDSLGISMPVIVTAWRRNHLTRQVQLFPPKYKRGLGGLHLVASASEIPPFVFRKHGHDSGAGYTITKWDGIEVHLLYMLSQMLNFTVEYKEPEFNEEEDVAQTVIKDLHTKKTTLAIGGVYLTPERIGGLTFSFPHTQDCASFISLASTALPKYRAIMGPFLWDVWLALTAVYLLAMFPIAFSVWHSIKPLLNDIREVENMFWYVFGTFTNCFTFTGKNSWSKADKTATKFFIGTYWIFTIIITACYTGSIVAFITLPTYPETIDSSKQLLEEDYKISLLGSGGWEGLFNDTEDPVASKLYESVERVPNLYSGLRNVTRNVHSWRQSAFLGSRRLLEYTVKTNFTPDEDSKRLMFHLSDECFVPLFVSIVMDKRTNYLEEFNNALERIIQSGFMTKIVREVEWQEYRSASGKLLTMHKGLKGAPEDRELNLDDTQGMFLLLGAGFGIGLLVLIIEISVWSSEQRKNRQFGELTLKQRAINKLKEHWETLYACLLAPANSGIIYFRERRVSSAFGEYVTQPYASWSITSIPSPTVEPPSPSPPNGEISSAPTVGNAASLSMDQLSFPPEKPIRMMSF

>AlinIR25a

MPSFTARLPGTTSVAKVFTVFMFYVSLLQKVHSQSATSINVMFVTEDRNDIARLAFDVVSDYVKRNSKLGIEMEVFRVTESGSDAKFLLENLCETFNASAKAGKPPHIILDTSVVGVTSEAVKTFSRALGIPTLSASYGQEGDLRQWRALEVEIAKYLLQINPPADIIPEVVRSIVILQNISSAGIVFDDSFVMDHKYKSLLLNVPARHIMGRVRNIQEIRNQLTRFKELDIVNFFILGSLSTIRNVLNEANGMKFFDRKYAWHAITQDKGQLKCDCSNATILHIKPEPDPGSKERLDNLRTSYNLVEEPEITSVFYFDFFLRGLLAAKALIEKAPWPKDYNKTSCDNYDENHDFIRKDLDLRSSLRDVKEAYSYAPFLISTNGKSFMEFNMKIEKVVIVNSIAESAEAIGTWKAGLSNQIQTKDIASMRNFSAVTVYRVVTVKQKPFVIETFENGKPKYSGYCIDLLEDIRSFVHFEYDIYVAPDNAYGNMDPSGNWNGMIKELIEKRAEIGLGALSVMAERENVVDFTVPYYDLVGITIMMKKQTTQTSLFKFLTVLENEVWLCILASYFFTSLLMWVFDRWSPYSYQNNREKYKNDEEKREFNLKECLWFCMTSLTPQGGGEAPKNLSGRLVAATWWLFGFIIIASYTANLAAFLTVSRLDTPIESLDDLAKQYKIRYAPINGSEAMTYFQRMADIEERFYEIWKDMSLNDSLSEVERAKLAVWDYPVSDKYTKMWQAMKEAGLPATLEEALDRVRKSQTTSEGFAFLGDATDIKYLVLSSCDFQIVGDEFSRKPYAIAVQQGSPLKDQFNNAILQLLNKRKLEKLKEKWWTENEDRMQCEKQEEQSDGISIHNIGGVFIVIFVGIGLACITLGLEYWWYKYKKPASPKQVGPMAQIISTNATNKQLSVTGLMDYNTREPRARYPIRRTAVNATQDYSRPTFPAQQERLSHW

>AlinIR41a.1

MPDMVKNLQNKTLKIMTFDYDPYTHFEPLDGTEIKLIQEFCKKHNCSLVAVDDGHFWGDIFENGTSDGLAGMVYDGRADFGAAAVYLWLPYFYFVDYSTSYLYSASTLLVPKPHPVSGWKTPFMPFDVFTWIGYGLSVIMAAVFMYVITYFTVKYTRFTEAVRKRRMFLDKLDCLFRALGLAVLQQPSTPLVPNTPIRHLFTSFEFLFLITSSIYAAELASYLTVPRYEKPIDTLIELADSGMIWIGEHESWTYSLRGMTDPEIVTIVNNYRIFSHEKLRQLAPTGEYGLIVERLPGGQYTEQEHVTDEVVAQSHMMAENLFGSPPVIAVRKGSPYRKYLNKVISNVLCGGFYLYWEREMSRKYLHSRRQLALREAEHPHYKDIPKRLTISHIQGGLYLYSLGITISLFVFILELFHFKAKGPKNRPKDK

>AlinIR41a.2

MLSSSSESLPKNCCYILTIIVSLCAADFRTKNEEDVFNAKMVYLARQVAQDYMSDRLRCIVVVSDEGLLEDFTGYNDTTVLRVLFNGSRDECDPTMHKYILQAFYHKCTRYIVQISKPTCFFPAWFLARNGSTYEKHNPRVLFLPVKPHATVYGEEVLAMNQTNISHDILIAETSPQTPIGAIAMPKEIDPNRPVTLYTNNFWQYVGEPGRIGRIYLDEWSWELGFKNGVDLYPDKVRDLRGKVLRLSAFPYLPYGNNEPMDGSEARILLEFCVVYNCTVVDVDDGHLWGEIYPENGTGVGEAGTIYMELSDFGVGANYLWLEFWPYLEFSNCYLYGALTVMVPRPELLSGLLTPFLPFPLSLWLVIVMCVVVSAVGLHWVTEATIKFAPHFLDEIYKNHKFITYTDSMIRSIGMLVLQQPQRLVTGSPVRHLFTAFEFTYLVITSAYAAELYDFLTIPRTTKPINSVFDLAESNLIWMTDHEVWVFGILHAEDPSIRKAASNFRALPTPELIKLGESNHPYGLGIERMAGGHYTELPYITDKFIEKSRVMRDNYYVSPLVVNMQKGSPYANRLNDIIGRMENGGLYYAWEADCVRKYLNYTKQLDMQWSTRPIKYPPKVLNVADLEGAFLLYFIGTALAVGLFFLEIYFKKGLKLKNSFLNPTPKWFDEYLLGQSGSHD

>AlinIR41a.3

MNNPLNLCTDGLIISMWLFVIGSSTTSQANVLFDNKHTILLGSLEKSVITQYFHKDKCIVLIVEDDTWTTNKELEHYYSLLSLILVSSIDICRDPYLVESIVSAIDSGCYSYILRVAEPKCVLKSWGESQLSYNVHTFQRVSPKMFATISNHQKDGSKIVDEFYSLPEAELSSNIVVAVFGNESLEWPVTIYTNNFYEPMSSADREPKVFLDRWNELNGFELQADLYADKILDLQGKELKVAVVDLLPYAELRRFIGQEAQILKHFCAHRNCTIKGITDEWFWGEIFENGSGNGLLGMVFDGRADFGIAGVYGWASVFRHTEFSASYLHSGVTLLVPKPVKVGGWLIPIFPFSSEMWLAYILSVIVAGISMHIITMATIKYTRFAEVVLKRGMFLTAVDTAFRALGLSVLQQPSTPLVPHTPIRHLFTAFEILYLVFCTVYAAELASYLTAPQYSKPIDSLEDLADSGMIWLGEHYGWVYSLLDVDTPSILKIVDHFKVVTFEEMDKLAGTGMYGLIVEQLAGGHFSERQYLSEKIIAQSHIMAEYLYDSPVITIMRKCSPYREHYNELIGRLLENGLLLFWEAEAARVYMSSWLQTALKSAIKVNMEDEARALTLSDCLGMFLLLAFGLFTSSVVFIVELWIIRQKNNTKD

>AlinIR41a.4

MVNVSLVGSTQRFRWILQLSDPAKFMENWEKARVDSLIRFKPRILFLPWNESNYATTLFEAPELNYIDDAVAVELEDADTNRNKQLKLVTNNFFVDIGSKNETTEIYLGVWPLNNSIEIFPNKIGDLQGKELRIATLQYLPYSQVSPELDGVELRILKSFCKQSNCSLVPVTDDFLWGELFENGTSNGIVGNVLQDKADLGVGAVYLWYYDHIEFAYPYMPSRVTVLLPKPSPMPEWRVPLAPFDFALWVALIVSIATVAFVLFYMNHYLQRFSSHHVSPNEFQSWSGVFLRAVGMAVGQSPQNAFSAGSTLRIVFTTFEILFLLYGTVYSSALASVVTVPAYYPPIDNMRQLYASGLPWTADHIAWVKNLMDADEPFIKDLLSKFEVHDQEMLSQLAKKGGYGFTIELTNGGHVSEASFLSADMINNLHVMKEALYYTYSTTIARKGSPYVDELNKLLHKCFDTGLLQLWESDMISKHGSSIIQTAFKLSKSAKASNEHHELVKLKLKHTQGAFILLLLGNTIGTLVFLFEFYCKSMKQVVKPTKMN

>AlinIR41a.5

MDLHSDWCNLMCFLIITQHIGSNWCVDHDEMTTGLAHEIAEHYFSEYNECITVVADLGALQKFSPPNNSFIRVSFDHTNDSCDPSVKESLRNSFREKCVRYVVQIAKPNCFFPSWFESMNLGYERHNPTVVFLPSINADDQNYGDDLLSKNETNISADILVAEISDAEEWAVKIYTNNFYQLTHEPERISKIFLDEWHPSKGFRFKADLLPNKLKDLKLKTLRVFTVQYLPYSSYDPFDGSEVRMVKEFCNVVNCTAVGLTNDGNWGTFDEETNTGTGQMGAIHSGEADIGVGGNVVWLEFFPHLDFSDAILGGASAIIAPRPKVLGGWFTPFLPFPLDLWVVVWAVVIASAILLYLFTTLTIRTIPHLAEKHRRNEKFVTFTDSLLRAIGMLISQQPSNLVTGSPVRHLFTSMEVIFLVITTCYCAQLYDFLTVPRTTKPIDTVKDVVENNLTWLAPSDLWIYALKHSDDPVVAKFVQLFQAYPPEEIIELSEKGEVGVTVEKLAGGHYVEDSYISYKFISQSRVSTGPDIYGMTPITLFMQKGSPYTEALNRFHGHMQNGGLHFAWEAGTARDHLNYTIQEGIAQSSRKQVYPPKVLRLADLEGAFLIHFIGVAVSIIVFFIESRVGKKKKSL

>AlinIR75d

MGALLPYSLITQYFINIHVSSIIVVSCCTTSQTAQLLRHLSQRGITASWAVDNLSPLEVRRSGIVLDLSCNQSKEILHDMSSRKMFGLEMEWLLMSEGSAPEEAELPDLYILPGSSVTLSVTSPSSISFYDTYRITRRLPYKFTLLGAVARDEDVLPQWKRPSRVNYEQNLLTTVSVIHSLDIRKLTDPDVAEEDRWPAIHFPVVVNVAYQLNFKFDLRLESVHGWKFPNGSFEGMIGVMEREEVDFGASGVIMREDRRKHVDYTVDYFEFKTGIIFKQPSLSSVSNIYLLPFSRHVWAACGGLLLFVLIILCIAVSSGDAQTFTPPATFLDMVNIVLGFVCQQGSYLAPVTISGRIVVFVSSLAALFLYTSYSANIVALLQSTSSVLKTLKDLTNSHLGLKVQINEYHLGYFLEAVDEDVITLYNKKVKNQPETFVNGTRGVEFMRTGDFAFCVEFDLAYKQISKTFQEEEKCGLGEMHLFFVPRLSIPVIKRSGHREHFTQTIIWQWESGMLDRISRIWLARRPRCESTGGGYLRVGLKDFNPALKVILVGIIISIWFFLCELITDRGFKAYYRKIKHNQEKIMGDDGHLIADLCFPSWKMLFKNKRF

>AlinIR75q

MQQVGLVGKDYNYIITSLDLHTIDLDIFKWAGTNITGVRLVNTESEHYREIMELMLAIKKEEEEEAYRPFTYDEQRRKRRNISSTLKSEDLGSNKIMSATSGRKTYSKRELQNKEGSRIIPPVEAMLIYDAVVLAAQALHSLSHVNPKQINCLMRSAWESGYSVINYMKMSEFYGLTGEVKFDNEGFRTDVALDIIELTQSGLHVKGNWSTYGGVNIQYPEPETDLTEATDDLRNTTFVVIIALTHPYGMLKESKYTLVGNDRFEGFGIDLIHELSEMTGFNYTFRVQEDKSSGNPTTLPNGTRVWNGMIGEVLAGRADLAIADITITREREHDVDFTMPFMSLGISILYRQPRAAPPSLFSFLSPFSYEVWGYMLSAYLGVSFLLYLMARISPPEWTNPYACIEEPVELENQFSLSNSLWFTIGSLMQQGSEIAPIAVSTRLVAAIWWFFTLIMVSSYTANLAAFLTIEQKVSLFDNVQELADQEVIKYGAKRGGSTANFFRDSHDPTYKKMWEFMSSHPEVMTDSNEVGVDRVDSTTDYAFLSESTSIEYETERRCNLYKVGRELDEKGYGIAMRQNSTYRNVLSRSVVKLQEGGQLDELKKKWWKEKRGGGSCLESPSGGAEDLGLDNVGGVFVVLLGGCIFATFLAFGELSFAIYMMEDKESFKEEFKKELKFIMKCSGTSKPRKIPSISSSTSSNPSVRSKSRSASRSRTSTVRTPNFSLQFP

>AlinIR76b

MSPFVHLMLVAMCANYQTNLLATDNQNFTCILKSEEQIKKEVYKGKVIKILTFDEMPLSGARKDGKGGMIGEGVAFELVETLKEKFGFDYTVERMAPIVGDESHGALGKLVSREIDMVAAFIPVLPDAHEFVKFGKDLSQAAYYVMLKRPADSNSGSGLLAPFDTVVWLLILVSLAVVGPVFYGVMWLRDRLCPGDIDQVYPLSTCVWFVYGALMKQGSTLNPLADSARMVFATWWIFILILTAFYTANLTAFLTLSISTLPIKEIDDVAKDNRHWFALQGGPIEHAIKDKEDEKLRKLRDSAASGRATFLETKQESIILQKITNDWYYLDDSYSLTRMMYDDYNRKSDMNAESSLRCAYVLTEKPFLVRSLAFAYGKDSPLPDLFNPILERFIESGILQHKLNLDLPDAVICPKDLGNKERKLRNADLWTTYLVVFSGVSVAFMIFLIEIIWRFYRKVKGSNQGVFNKQQMFQSRLNTDKLLAMRDQVQTKINGRDYYMVTNKGGNSHFIPLRTPSALLFQYG

>AlinIR93a

MSTGLFLVQFVLLMIMLKDHECNAAIKLHNDKRANDTLLIIIDESFVDSLDRNIEQQVRQIVSEISSRILKKGAVDILYHSNANIYLEPDVTAVFSMTSCLDLWTLFNRGKKFDILFISLTEANCPRLPPDSGITVPLYRRGWEIPQIILDLREDGSLTWETTAIIFDDNLEEDMLKSIIEVLNRPKKSSEMACSVVLYRLHSFPKDIQNKKKKNMDNLLENLPPIDMVNNFLVLIDQRKITPTLELVKKMGLVIPTAQWMFVVRNLNVMKRRTSPEKRYVDLIGEGENVAFLINSTRADSRCDMGLLCNARQLVEKLIIAIEKSIEQEIILADSLSDEEWDVLKPKKLERREHILDFIKKKTRDEASDCDSCTEWIIRSSDSWGMDFVQSKGSYGNKESNSTTGALLEVGHWQPRSGLVLVDHLFPNIVGGFRGRTLPIASVHFPPWQFVKYDEFGQPSEYGGVVFNVLNELADKLNFTYEIVLLPNGTSAANKFTLHKELGEVVIDSSVEFAAWDQVVLDLKSNKVFLGAVAFVETEERKADVNFTHPVATDAYAFLVSRPKELSRALLFIQPFTGETWLCIIATILLAGPLLWFVHRVTPFYDHYSHRGKGGYTRLYNCFWYLYGALLQQGGGVMPEADSGRIVIGTWWLVVLVVVTTYSGSLVAFLTFPKMDKVISNVDQLLERSAVSGDGMITWSFPKISTIHRLLKDTDNKKFNMFYEASEKLEQLTPEIIAKIQNGEHVYIQRKTMLLYIMKQEFLRTQRCDYSIGSEEVLKERLGLVVKSNSPYLKIINQHIHDMHKVGLINKWLEDSLPKKDKCWMSTLGSSSSTHTVNMSDMQGCFFLLFIGVFTSILLIGGECFMKWWKLRKQKSIIQPFIS

>DmelIR7a

MFHHLWLLMGLRSLAMGALHPPQPEAMTPLVAAALEILAEQVSPSQSTLAVMDLTQDAEHRDERQEQLMTIILRSVGSEMALRTFQKPPAEVPASFVVFLVNSAQAFNTLGFHFTDIHSTREFNFLILLTHRMSSRAERLQVLRDISRTCVRFHTSNVILLTEKRDGVVLVYAYRLLNMDCDLSVNLELIDIYKNGLFRHGHEARSFNRVLSLSGCPLQVSWYPLPPFVSFIGNSSDPEERAQIWRLTGIDGELIKLLASIFDFRILLEEPCNKCLSPDIKDDCSGCFDQVIISNSSILIGAMSGSHQHRSHFSFTSSYHQSSLVFIMHMSSQFGAVAQLAVPFTVIVWLALVVSSLLLVLVLWMRNRLVCGRSDLASHALQVLTTLMGNPLEARSLPRSSRLRILYAGWLLLVLVLRVVYQGKLFDSFRLPYHKPLPTEISELIRSNYTLINQEYLDYYPRELTVLTRNGSKDRFDYIQGLGKEGKFTTTSLIATMEYYNMMHWSTSRLTHIKEHIFLYQMVIYLRRHSLLKFAFDRKIKQLLSAGIIGYFVREFDACQYRKPFEEDYEVTPIPLDSFCGLYYISLIWLSAAVVAFILELLSQRIVWLRRIFE

>DmelIR7b

MKYWLYILSCCSLVASTMESSSDWDLAEALAQVVANSEMGRFKTLYIYTHTNSQSTGGHLEELLDQVLMIVPNNLQARRLLLQQSMEYKPYVHAVLALVDGLPSLSAIYARIRATQDLSHTLIYMSMPTDAYGEEMQATLRFLWRLSVLNVGVVLRPPGDHILMVSYFPFSALHGCQVISANVVNRYQVGTKRWASQDYFPSKLGNFYGCLLTCATWEDMPYLVWRPDGSGSFVGIEGALLQFMAENLNFTVGLYWMNKEEVLATFDESGRIFDEIFGHHADFSLGGFHFKPSAGSEIPYSQSTYYFMSHIMLVTNLQSAYSAYEKLSFPFTPLLWRAIGLVLILACLLLMLLVRWRHHHELPRNPYYELLVLTMGGNLEDRWVPQRFPSRLVLLTWLFATLVLRSGYQSGMYQLLRQDTQRNPPQTISEVLAQHFTIQLAEVNEARILASLPELRPEQLVYLEGSELQSFPALAQQSGSSARVAILTPYEYFGYFRKVHPMSRRLHLVRERIYTQQLAFYVRRHSHLVGVLNKQIQHAHTHGFLEHWTRQYVSAVDEKDESVARIASTSYSTLDGIDGDPSLSESEEDQQVAPVRQNVLSMRELAALFWLILWANLGAVVVFVLELLLPRIKLRKILRKMKSDIKKQISKLVRK

>DmelIR7c

MLHSAVHNVSLVYALVWAIDNYYGMATSTPLAVVQFPTSRESRRLHNDLIDAALGRSSGTGRIQFLLEDDRVEMTETDTDPPPPSGLTGRPIAIWFLDSLRSYFRLEMYLNQLGSPYKRNGFFLVIYTGLEDQPMESLKIMFRRLLNMYVLNVNVFLQRDGTVHLYTYYPYGPHHCQSSLPVYYTAFQDLAAPANGFGLTKPLFPRKLTNMHGCEMVVATFEHRPYVIIEDDPKTPGGRSIHGIEGLIFRSLAERMNFTIKLVEQKDKNRGEILPDGNFTGILKMMVDGEVNLTFVCFMYSKARSDLMLPSTSYTSFPIVLVVPSGGSISPMGRLTRPFRYIIWSCILVSLIFGFVLICLLKITALPGLRNLVLGRRNRLPFMGMWASLLGGLALYNPQRNFARYILVMWLLQTLILRAAYTGQLYLLLQDVEMRSPIKSLSEVLAKDYEFRILPALRTIFKDSMPTTNFHAVLSLEESLYRLRDEDDPGITVALLQPTVNQFDFRSGPNKRHLTVLPDPLMTAPLTFYMRPHSYFKRRIDRLIMAMMSSGIVARYRKMYMDRIKRVSKRRNLEPKPLSIWRLSGIFVCCAGLYLVALIVFILEILTTNHRRLRRAFNVINRYAA

>DmelIR7d

MDIRCVVALLLGLCKVQAVVWPHQHLLEEQLASQISATLQKIFINGLAVYNFGVFISTSYEEMDRDRVILVHQVLNRNLYPPNFPVAVVLASKMNRKITAQVFTQLLFVQNAEQAIAIAEGVNRNGLCVIVLLTSQPERPIMTKIFTYFMQERYNINVVILVPRLHGVQAFNVRPYTPTSCSSLEPVEIDIKDGDLWDVFPRRLKNLHGCPLSVIVWDIPPYMRINWKSSDPMDGLDGLDGLLLRIVARKMNFTLKLIPNEPNGLIGGSSFMNGTFTGAYKMLRERRANITIGCAACTPERSTFLEATSPYSQMSYIIVLQARGGYSIYEVMLFPFEKYTWLLLSTILGLHWIVGSRWRMPSPILAGWMLWIFVIRASYEASVFNFIQNSPVKPSPRTLDQALSGGFRFITDHASYRMTLKIPSFQGKTLISAGQPVDVFDALLKAPWKTGAFTSRAFLADHLVRHRKHRNQLVILAEKIVDNMLCMYFPHGSYFAWEINKLLFNMRSFGIFQHHSQILAWDNLPTTTDTDTPGKRIHSSTESVATGFAESMSFVVAALNCLMGALCISIVVFGLELLSRRRHWTGLEWLFERV

>DmelIR7e

MNISALLNSYYDLSGEQMNHINEFVARAVLHVVHHYILSVTPSLVLTLCCRSNHTCNFYNKMMSTLFREWGLAPLQIVNVLRGVPWHPVPGRRHFNVIFTDSFAAFEEIRMEYYSREYNYNEHYFIFLQARDRLLQGEMRLIFDYCWRYRLIHCSIQVQKSNGDILFYSYYPFGEHGCSDMEPQLINRYNGSMLVEPDLFPRKLRNFFGCPLRCALWDVPPFLTLDEDQEEVLRVNGGYEGRLLLALAEKMNFTIAVRKVHVNMRDEALEMLRRDEVDLTLGGIRQTVARGMVATSSHNYHQTREVFGVLASSYELSSFDILFYPYRLQIWMGILGVVALSALIQLIVGRMLRERMGSRFWLNLELVFVGMPLLECPRSHTARLYCVMLMMYTLIIRTIYQGLLYHLIRTHQLNRWPQTIESLVQKNFTVVLTPIVQEVLDEIPSVQHMRFRLLEANSELDPLYFLEANHQLRQHVTASALDIFIHFNRLSADKVHQRGEQGSGAHFEIVPEDIISMQLTMYLAKHSFLIDQLNEEIMWMRSVGLLSVWSRWELSESYLRNEQSFQVLGTMELYAIFLMVLVGLIVGLLVFILELVSMRSIYLRKLFT

>DmelIR7f

MNTTSDSNAGSSLSSGSGYSIYKSYLENSRIDMQGEDANLYVARALRLVIENVLAQLSTTLVVTISTRHLGTAHWFEYMMNILMDSWRMVAVQLLRIRPDLVVNPVPGRKRVSLLMVDSYQGLLDTNITASNANFDDPDYYFIFLQARDHLIPKELQLILDHCLAHFWLHCNVMIQTAQVEVLVYTYYPYTADACQKAYPIPVNTFDGRKWKASQMFPDKLSQMHGCPLTVLTWHQPPFVELVWDPKHNRSRGSGFEIQLVEHLARRMNFSLELVNIALLRPNAYRLAEGSSEGPIEKLLQRNVNISMGYFRKTARRNQLLTTPMSYYSANLVAVLQLERYRIGSLALLVFPFELSVWMLLLLALLIHLGIHLPSARRGNEEDGGGGLQVVALLLGAALARLPRSWRHRFIAAHWLWASIPLRISYQSLLFHLIRLQLYNTPSFSLDQLLAEGFQGICTANTQRLLLEMPQLARDPDSIQSVDTPFDWDVLNVLTRNRNRKIFAVANQDVTLSFLHSSAHPNAFHVVKQPVNVEYAGMYMPKHSFLYEKMDDDIRRLDASGFIHAWRRASFASVHRKEQVHMTSRRYINHAKLSGIYMVMAGLYLLAGLLFAGEVLLRQRN

>DmelIR7g

MNVTSLLNFESMKYIGAQTQAASINHHVAQALRVFIEDFYQRIAPAFIVVLSCRRPSPMNFYRNIMQLLYESVDTMIVQLVLVELGRPRRIAGPRTHNLLLVDSLDALLDIEIHTYTAQSDTSEYYFIFLQQRDALIPHDMQGVFAYCWRHQLINCNVMTQSSGGQVLLHTYFPYAPGQCNDSQPTRINMFLGESWKHRDYFPSKLHNLNGCPLIVLARKVSPFLDLDEGQRELRGLEGRLLQELSRRMNFSIQFSGLQDQLKNRTTWTEKQLLQKLVQERIAHLAIGYVRKRIQYATNLTPVFPHYSNRVVGCLLLNAHNLTSLEIWSFPFQALTWICLLLFASWLIFGLIVRSMYSALLFFILRYHLHQRLPGNLQDLTHGDYAAVMGRTTLQDLREVPSLQDLLGLKSVIVTSEREEEVLRTLDRCTLREGAGSHPLFFGLISQDALLHLTQRGHRAGAYHIIPQDVLEQQLAIYLQKHSHLASHLDHLVMSIRSVGLVHHWAGQMASERYFRSRFLYREKRIRQPDLWAVYILTAGLYLLSLVVFICELLASRRAGL

>DmelIR8a

MELPLLVLLLALRFAGSEVLKITFWIEPVQRAEFDTDIAMVLKELDALRLDVKVDDTTLTLTRSEDGLDMQRFCEILSTVGASAVIDLTYSHWEEGYNLVRSLGIGYVRLERIMRPFLDMFGDFMRQKRANNVAMVFMNARDAVEAMQQMLVGYPFRTLIMDASQTDPGQHFLERIRSLRPAPTYIALFARAAAMNGIFEKVQKADLFQRPLEWHFVFLDTRDRVFKYRRQAELCTRFTLNPRAICRSMPMPDLYCGSGFTMQRAMLLNVLRSLINAAQVSPGYPLAIYQDCNATASSSEVSDPLEKDDYNWLDMVHWSNFLAYAPPLPHIQDQFQSPVPGLTFAVNISAGYYSSEHEAKTDLAAWSSVGEMRLLNETISPARRFFRIGTAESIPWSYLRREEGTGELIRDRSGLPIWEGYCIDFIIRLSQKLNFEFEIVAPEVGHMGELNELGEWDGVVGDLVRGETDFAIAALKMYSEREEVIDFLPPYYEQTGISIAIRKPVRRTSLFKFMTVLRLEVWLSIVAALVGTAIMIWFMDKYSPYSSRNNRQAYPYACREFTLRESFWFALTSFTPQGGGEAPKAISGRMLVAAYWLFVVLMLATFTANLAAFLTVERMQTPVQSLEQLARQSRINYTVVKDSDTHQYFVNMKFAEDTLYRMWKELALNASKDFKKFRIWDYPIKEQYGHILLAINSSQPVADAKEGFANVDAHENADYAFIHDSAEIKYEITRNCNLTEVGEVFAEQPYAVAVQQGSHLGDELSYAILELQKDRFFEELKAKYWNQSNLPNCPLSEDQEGITLESLGGVFIATLFGLVLAMMTLGMEVLYYKKKQNALEITQVRPVNDSSGSGGNSSTAPPTATSTTKQAWHIPVLEAEEKPAKVSPPPSFETATFRGKKLPARITLGDGKFKPRHGLYARRNLGASDSHSGYME

>DmelIR10a

MAVLGTVFLLFMLDLKTLNLTRLNGLLVEPTRDLPQLELWLRAGSDHQDAENPYVQWFLLRTEIPLSIVTYQENRYWMDDPFGRRNLVLVMSLDQLLTNRGAAAPIQKASTFFYILADQDKDLSADEQLRLEGSCRQLWTQHKVYNRFFLTRDGVWIYDPFKRRDSAFGRLVRYYGSETLDKLLFRDMAGYPLRIQMFRSVYTRPEFDKETGLLTRVTGVDFLVAQMLRERLNFTMLLQQPEKKYFGERSANGSYNGAIGSIIKDGLDICLTGFFVKDYLVQQYMDFTVAVYDDELCIYVPKASRIPQSILPIFAVGYDIWLGFVLTAFACALIWLTLRVINLKLRIVSLGNQHIVGQALGIMVDTWVVWVRLNLSHLPASYAERMFIGTLCLVSVIFGAIFESSLATVYIHPLYYKDINTMQELDESGLKVVYKYSSMADDLFFSETSPLFASLNKKLSWNRDLRADVIDEVARFRNKAGVSRYTSLILESSHFTLLRKIWVVPECPKYYTISYVMPRDSPWEDAVNALLLRFLNAGLIVKWIQDEKSWVDIKMRSNILEADAESELVRVLTIGDLQLAFYVVIGGNLLAFLGFLAEHFRWKLQKKGV

>DmelIR11a

MRFAILWLFSGCLLPGIQVGIWVVVRAQPTGRDVLLSRLGNQQNELNTRRLANASSYLTRNYIANRINTLVVREICVECPYELSERQRQLVDQILASLAPELSVLLHKGTAEETTWEYTLFVVNDHTAFTGQVFIFPDELLEREFFCIVVVSEIQSRQFVRQTVGSIVKSNLQMHFVNVVVVAQLEDGTVGTYSYKLFKANCTPGITVRQINHFDRITGKPQQSMPDLYPVRNGHLGDCPFNVGAAHMPPHLIYKRHKDPPPASNVSIPAEDLAGIDWDLLQLLAKALKFRIQLYMPQEPSQIFGEGNVSGCFRQLADGTVSIAIGGLSGSDKRRSLFSKSTVYHQSNFVMVVRRDRYLGRLGPLILPFRGKLWGVIIVILLLAVLSTCWLRSRLGLSHPIEDLLTVIVGNPIPDHRLPGKGFLRYLLASWMLLTLVLRCAYQARLFDVLRLSRHRPLPKDLSGLIKDNYTMVANGYHDFYPLELTCRQPLDFSARFERVQRAAPDERLTTIALISNLAYWNHKHPNISRLTFVRQPIYMYHLVIYFPRRFFLRPAIDRKIKQLLSAGVMAHIERRYMQYENKRKVASNDPVLLRRITKSIMNGAYRIHGLVIVLATGMFILELLAGRSNGRLRRWMEWVHQ

>DmelIR20a

MLASLNRSTGLSAELLDLYGLVVHFLLSGEHTTLVYFNPAGLDCSWGVLWQRNLTAHPQIVWQRNYSYPDLYYQFNAKLLVLACLPMDSRAAIQLEILANSLSHLRTVVRLLIEVAGPDQVTLARQYLSFCLRRSMLHVELYFRDYHHSLILYSFRAFPSFELVMRWISVGQGVKLFLHKLDDLRGHRLRVIPDLSPPNTFFYRDARGDNQVTGYLWDFLATFAGRLNAGLEVVRPSWRAGSASDSSYMLEYSAKGLIDVGLTTTLITKWNLWAIHQYTYPLLVSSWCTMLPVEKPLATPDLFGRIVCPTLAMTLLLIILVTWLVFRQLRCLTRLKNSRPARIVPHLLTLLLLTTCSAQLLSLLIFPPYHVRIASFEDLLRGDQKILGMRNEFYNFDGAFRARYAGVFYLIDDPNELYDLRNHFNTTWAYTMPYIKWLVIKTQQRHFSKPLFRWSKDLCFFDFMPTSVIVAPDSIYWESIKDFTFRIHQAGLMKHWIRKSFYDMIKAGKMSIKDYSDLETLKPLNIGDLEIVWRVCGAAIAVASAIFIMELLYFYINVFFNSL

>DmelIR21a

MSYYWVALVLFTAQAFSIEGDRSASYQEKCISRRLINHYQLNKEIFGVGMCDGNNENEFRQKRRIVPTFQGNPRPRGELLASKFHVNSYNFEQTNSLVGLVNKIAQEYLNKCPPVIYYDSFVEKSDGLILENLFKTIPITFYHGEINADYEAKNKRFTSHIDCNCKSYILFLSDPLMTRKILGPQTESRVVLVSRSTQWRLRDFLSSELSSNIVNLLVIGESLMADPMRERPYVLYTHKLYADGLGSNTPVVLTSWIKGALSRPHINLFPSKFQFGFAGHRFQISAANQPPFIFRIRTLDSSGMGQLRWDGVEFRLLTMISKRLNFSIDITETPTRSNTRGVVDTIQEQIIERTVDIGMSGIYITQERLMDSAMSVGHSPDCAAFITLASKALPKYRAIMGPFQWPVWVALICVYLGGIFPIVFTDRLTLSHLMGNWGEVENMFWYVFGMFTNAFSFTGKYSWSNTRKNSTRLLIGAYWLFTIIITSCYTGSIIAFVTLPAFPDTVDSVLDLLGLFFRVGTLNNGGWETWFQNSTHIPTSRLYKKMEFVGSVDEGIGNVTQSFFWNYAFLGSKAQLEYLVQSNFSDENISRRSALHLSEECFALFQIGFLFPRESVYKIKIDSMILLAQQSGLIAKINNEVSWVMQRSSSGRLLQASSSNSLREIIQEERQLTTADTEGMFLLMALGYFLGATALVSEIVGGITNKCRQIIKRSRKSAASSWSSASSGSMLRTNAEQLSHDKRKANRREAAEVAQKMSFGMRELNLTRATLREIYGSYGAPETDHGQLDIVHTEFPNSSAKLNNIEDEESREALESLQRLDEFMDQMDNDGNPSSHTFRIDN

>DmelIR25a

MILMNPKTSKILWLLGFLSLLSSFSLEIAAQTTQNINVLFINEVDNEPAAKAVEVVLTYLKKNIRYGLSVQLDSIEANKSDAKVLLEAICNKYATSIEKKQTPHLILDTTKSGIASETVKSFTQALGLPTISASYGQQGDLRQWRDLDEAKQKYLLQVMPPADIIPEAIRSIVIHMNITNAAILYDDSFVMDHKYKSLLQNIQTRHVITAIAKDGKREREEQIEKLRNLDINNFFILGTLQSIRMVLESVKPAYFERNFAWHAITQNEGEISSQRDNATIMFMKPMAYTQYRDRLGLLRTTYNLNEEPQLSSAFYFDLALRSFLTIKEMLQSGAWPKDMEYLNCDDFQGGNTPQRNLDLRDYFTKITEPTSYGTFDLVTQSTQPFNGHSFMKFEMDINVLQIRGGSSVNSKSIGKWISGLNSELIVKDEEQMKNLTADTVYRIFTVVQAPFIMRDETAPKGYKGYCIDLINEIAAIVHFDYTIQEVEDGKFGNMDENGQWNGIVKKLMDKQADIGLGSMSVMAEREIVIDFTVPYYDLVGITIMMQRPSSPSSLFKFLTVLETNVWLCILAAYFFTSFLMWIFDRWSPYSYQNNREKYKDDEEKREFNLKECLWFCMTSLTPQGGGEAPKNLSGRLVAATWWLFGFIIIASYTANLAAFLTVSRLDTPVESLDDLAKQYKILYAPLNGSSAMTYFERMSNIEQMFYEIWKDLSLNDSLTAVERSKLAVWDYPVSDKYTKMWQAMQEAKLPATLDEAVARVRNSTAATGFAFLGDATDIRYLQLTNCDLQVVGEEFSRKPYAIAVQQGSHLKDQFNNAILTLLNKRQLEKLKEKWWKNDEALAKCDKPEDQSDGISIQNIGGVFIVIFVGIGMACITLVFEYWWYRYRKNPRIIDVAEANAERSNAADHPGKLVDGVILGHSGEKFEKSKAALRPRFNQYPATFKPRF

>DmelIR31a

MNLLISMFILILAAGEGEIIPSMEESVVTNFVKSLVKTKQAIVFSCLFKDFKEISLALMRINQFVSVVNLNQSYSLTSILTRENYARTSVMVNARCSGSSELLFEASENRYFNKTYQWFLWGVDLEVQSLFPLNLNYVGPNAQITYVNETADGYAYWDIHSKGRHLKSNLEINLIATLINDTLNIARDIFHLQSIDFRGQFNGLTLRGASVIDKEDIISNEQIESILSRPTKDAGVAAFIKYHYELLGLLRERFNFTVNFRNSRGWAGRLGNTTFRLGLLGIVMRNEADIAASGAFNRINRFAEFDTIHQSWKFETAFLYRYTSDLDTHGKSGNFLSPFSDRVWLFCLLTLGAFSIIWVLFEIIDYKILRIRVNSQKLEHLNQKSSVICIKTTCIERILQTFGACCQQGLDPNPVDRSVRFLVMTLFLFSLVMYNYYTSSVVGGLLSSSDQGPSTVDEITASPLKISFEDIGYYKVLFRESQNRSITRLIEKKLSSSRSLNELPIFSHIEDAVPYLKAGGFAFHCEVVDAYPVISEYFDANEICDLREVSGLMEVEILNWILHKNSQYTEIFKTAMCNAQEKGFVERILRRRQIKKPACQSLYTVYPVSLSGVLPGFVILICGFGASLLLLCLEKVYAHFGPRKFCGF

>DmelIR40a

MHKFLALGLLPYLLGLLNSTRLTFIGNDESDTAIALTQIVRGLQQSSLAILALPSLALSDGVCQKERNVYLDDFLQRLHRSNYKSVVFSQTELFFQHIEENLQGANECISLILDEPNQLLNSLHDRHLGHRLSLFIFYWGARWPPSSRVIRFREPLRVVVVTRPRKKAFRIYYNQARPCSDSQLQLVNWYDGDNLGLQRIPLLPTALSVYANFKGRTFRVPVFHSPPWFWVTYCNNSFEEDEEFNSLDSIEKRKVRVTGGRDHRLLMLLSKHMNFRFKYIEAPGRTQGSMRSEDGKDSNDSFTGGIGLLQSGQADFFLGDVGLSWERRKAIEFSFFTLADSGAFATHAPRRLNEALAIMRPFKQDIWPHLILTIIFSGPIFYGIIALPYIWRRRWANSDVEHLGELYIHMTYLKEITPRLLKLKPRTVLSAHQMPHQLFQKCIWFTLRLFLKQSCNELHNGYRAKFLTIVYWIAATYVLADVYSAQLTSQFARPAREPPINTLQRLQAAMIHDGYRLYVEKESSSLEMLENGTELFRQLYALMRQQVINDPQGFFIDSVEAGIKLIAEGGEDKAVLGGRETLFFNVQQYGSNNFQLSQKLYTRYSAVAVQIGCPFLGSLNNVLMQLFESGILDKMTAAEYAKQYQEVEATRIYKGSVQAKNSEAYSRTESYDSTVISPLNLRMLQGAFIALGVGSLAAGVILLLEIVFIKLDQARLWMLCSRLQWIRYDRKV

>DmelIR41a

MFIDLSWSLVLSAIVGKYLNESTICIFWNDKFEFQLLHKSDYISFVGINIKSFDDNGGHYIIDTGLKKKELQNKHLFLDELVIKIIISIEVTHCETFVVFDKDIDRFVNAFNKASVYSIWRSLHNKFVFAHIANESPESRNHFFEDQPNILFVVRDHSSASSFDIKTNKFVGRKAENPSQMILVDRYLASEQRFQFGKSLFADKLNNLQGREVIIAGFDYPPYTVIKHNMSTNAQDMGVSGESDFKNVYIDGTETRIVLNFCEQFNCTIQIDSSAANDWGKVYPNMSGDGALGMLINRKADICIGAMYSWYEDYTYLDLSMYLVRSGITCLVPAPLRLTSWYLPLEPFKETLWAAILLCLCAEATGLVLAYKSEQALYVLPGYREGWWTCTSFGVCTTFKLFISQSGNSKAYSLTVRVLLFACFLNDLIITSIYGGGLASILTIPSMDEAADTVTRLRFHRLQWAANSEAWVSAIRASDEALVKDILYNFHIYSDDELLRLAQDQHMRIGFTVERLPFGHFAIGNYLGPQAIDQLVIMKDDIYFQYTVAFVPRLWPLLDKLNTLIYSWHSSGFDKYWEYRVVADNLNLKIQQQVQETMTGTKDIGPVPLGMSNFAGFIIVWILGSAIATLTFLLELSLTYILKQSNLK

>DmelIR47a

MRQIKLLVWLLVVGVVSSTEQLQFLKNFLEAVHKERSISTILLIQRKVHKNDFLHGLYPIFWPIICLDETKRVELVNNFNKDFLALVYMESEADTLLLSALAADLNHIRDARIMIWLQMSPSENFLDRIVFQASKQKFLNLVVIENTLKTRRFYPFPQPKVQVIDKPFEEKEIYPALWRNFMGKNAIAVPDLVPPRSFNSFDPKTGHRRESGSIYNVFKAFTQRYNITMLLKWPLIRNTTQEEIIGKSVRGEIDLPITGQLISFRHPNGSRSQPLLGMTALSIAVPCGPELPMFDRFFLFYGLATPITITGYYVLLNTIEIILGTLSDRIKRHPRRKKILNLVLNLRVFSCILSLPTPQGNRLRSVKGQLTMVMSITGLILSCIVAAQTSTILTMKPQYRHIKNFQELSDSNITVVCNHLNYLTIKQQMDPKFMAKFMQNIWIVNSIEQMKMIFDLNTSYAYQTFSYKKDPFTLLQMHTTRKAFCRTPGLDLVSGLAYTAVLEKNSIYALALQDYTLKAFSAGLVYYWAEESIRDLISTVGRTQFEKLPIVIGYQSLKLQDYNVCWKILLIGGALAFCVFIVEVVVGLINRRI

>DmelIR48b

MILQQSSNLLKLLLLLAISSVRTQGLNDIIIELNQRLLISNNFLYCNQSDKLNEYEIKYLQHMPPISLMIFTSIESMNFTQVEYNLGADNKLFLIMGNEEPPYDFLHALNLHFQFAEYIIVIDEPVDLKKSTKWLDFVNHLWQQGYVQLLIYTSYDEKLYHKIIFPETVIEETLVEQYISIRGSFNNLYGYPVRVAAYNNAPRSMLYVNRWGKHIFAGFYMRFLRAFIDARNGSFVPVLTPSNSPGNCTLNLVNETVDVCADALAANPAAFSLTHGFRIASANVLVTHAKPLHSYRYLTAPFQWSVWACLVIYVLLVVNFLSFIGWLRSGKWEFSKYLLEVFSSLLFSGFYLKEIRGRERYILFGVLFIAGFVYSTEYLGLLKSMLISEVFEKQIDTFEALVESNITLMVDPYDKILFAKYNMPEILSPIMELVSFETLLKHRNRFDQDYAYILFSDRMALYDYAQQFLKHPKLLRIPIDFSFLYTGIPMRKRWFLKHHLGRAWYWAFESGLTRKLALDADFEAVRVGYLSFLITEHVEAQPLNVDYFVMPAIALAIGYILALLSFVIEMTAWRIREFLGCRKATMTSTGCSEGGHVDVD

>DmelIR48c

MSLLRIILIIIFLRIVSSIPDTIISHLSAELQIKIQIYFGLGNDLYDFSRLDGNYQKIIISHNISEEFKTYHDEPVLIIIRLERDLNLNLATLDVLRSYLTDRQYNDILLIDNDEENLNSYVDIRKAYWNAGFSQVLIYNSQQRTWSIKPYPYLQIRPTSLKEYIENRNTRNLMGYPLRVLVTNDPPHCFVDKDELPGSPNRYKGSIVTMLKIFADQLNATFQANPFREFRRYSTADCVQMVSDDEIDACGSIFIRTYTYATSQPVRLNRVVIMAPFGNPIEKFYYFFRPFDLYVWIGTGIIVVYIAVMGSLLHRWHFKEWNVGQYLLLAVQTLLNRELSLPQSSSGSKFMLLLLLFAIGFILSNLYVALLSMMLTTKLYQRPIENLADLKAANVNILLQTHNIRPNSVYGSSEELRERFLLVEESQHLEKRNGLDPSYAYVDSEDRMDFYLYQQKFLRRRRMKKLSNPVGYTWAVQVIKQNWVLEKHYNDHVQRFFETGLQNKLVDDVHELAVKAGFLHFFPTQTQTIEPLRLEDIVMAAMVLGGGHALAVICFLVELFA

>DmelIR51b

MCKVLTLLVVILLLALTNAAYNVTLLKSVLSLISTREPWINTPIFVGHNTQGGDLNDLIIWLHQTMGVTSLTMNLFLQPEHIRPLGHFKITRYNGIALFFCHDKHDIMWLTLDRNLRKLRRIRLIIILRNQRSGSQGAIKSIFNALWQYQFLNVLVLQRDQLYSYTPYPAMRFFKLDIHTEPLFPHAARNFHGYVVSTPAENDIPRVFHVHDPLTKSRKVLGYAYRTFVEYLDHYNASLRLTNPDENLDPTTSVNMNHIVQLIIDGQLEISLHPYVFTPPTATKSYPLLIYPNCLIVPMRNEIPRHMYLLRPFQLYSWYILLFAVFYITGILYCISPKLNKSSWPQRLGLNFLDAISKILFISPPITIYRPTWRHLIIFLQLSVLGFMSTSWYNIELDSFFTTIVVGEQVNSMDQLVHQQQRVLVKEYEINTFLRHVEPRLVEKVSRLLVPVNASEQVSALLSFNRSFAYPFTEERWQFFAMQQQYAFKPIFRFSSACLGSPHIGYPMRVDSHLETSLNHFILKIQDTGLLNHWVVSDFNDAMRAGYVRFVDNVLGYQSIDVDTLRLGWCVLGIGWILSALVFSCEYWHLYPWRFIA

>DmelIR52a

MALGWSVIILGFIGQLSAQILNYTQSRDLELLEGSLFRVLSRLNLEEEYNTLLIYGKECVFHSLLRKLEISAVTVPSGSTDYDWSFSTAILILSCGYDAENEENSYTLMKLQRTRRLIYLEDNSEPESVCMRYSLKEQHNIAMVKSDFDQSDTFYSCRLFQTPNYVEGHFFKDQPIYIENFQNMRGATIRTVADSLVPRTILYRDEKSGETKMMGYLGHMINTYAQKLNAKLHFIDTSKLGAKKPSVLDIMNWVNEDIVDIGTALASSLQFKNMDSVWYPYLLTGYCLMVPVPAKMPYNLVYSMIVDPLVLSIIFVMLCLFSVLIIYTQHLSWKNLTLANILLNDKSLRGLLGQSFPFPPNPSKHLKLIIFVLCFASVMITTMYEAYLQSYFTQPPSEPYIRSFRDIGNSSLKMAISRLEVNVLTSLNNSHFREISEDHLLIFDDLSEYLVLRDSFNTSFIFPVSVDRWNGYEEQQKLFAEPAFYLATNLCFNQFMLFSPPLRRYLPHRHLFEDHMMRQHEFGLVTFWKSQSFIEMVRLGLASMEDLSRKRNEEVSLLLDDISWILKLYLGAMFISSFCFILEILRCGERCKRLWRCRW

>DmelIR52b

MTWLVILLCFLGYMAAHIADISVQNQSLMDNELINLLLKLRNEEFYDTLLVYGKDCEFHSVIKNVDVAVVLVSDSMNFEWNFSSLTLILSCGPDIDNGGPNSTSIKLQRNRRLVLLKEDFQPSNICNIYTQKEQYNIALVRENFTKSKSIYTCRYFQDPNVDEVNLSGTKPIFIEQFQNMKGKAIRIVPDLLPPRVMLYQDANDGELKMIGYVANLITNFAQKVNATLQLDFLKPSTSITEISRMAKDDELDMGITLEASLNTSNLETSSYPYLLTSYCLMVQVPAKFPYNLVYALIVDPLVLGIIFVLFLLLSVLLIYSQKMSWQDLSVANILLNDKSLRGLLGQSFPFPLNASKKLRLIFTILCFASIMLTTMYEAYLQSFFTNPPSEPEICSFQDVGSYNRRIAMSALEVNGLIKTNNSHFREIRMDDLEIFDNMPECYELRDAFNLSYNYVVTGDRWRSYAEQQTLFKEPVFYFARDLCFSRLIFLSVPLRRHLPYRHLFDEHMMQQHEFGFVNYWMSHSFFDMVRLGLTSLKDLSRPLAYTPSLLMDDISWIMKIYLAAIVLCVFCFLLEIGVDKWKRWMKFRNLQILNTC

>DmelIR52c

MVWLIIILFCLGNSSSQILDVTNNSHLDFDYRLFGLLQRLQVEKSYDTLLVYGEDCAIPSLFERLQVPAVLVSSGSTNFDWNFSSLTLILSCNFQDEREENYRTLMKLQTSRRLILLKGHIKPESVCDFYSKKEQHNVAMVKENFYQLEVVYSCRLFQDQNYEKLNLFDGKSIYKDQFRNMHGAPIRTLSDKEPPRTIPYIDSKTGEEKFKGYVGMLISQFVKKVNATMQIREDLIKDDEEVSFVDITNFTSNDILDIGICEARTLEMSNYDAISYPYLMSSYCFMAPLPDSLPFSDVYMAIVAPSILIMFLIIFCICSVLIIYIQERSYRSLTIRSVLMNDICLRGFLAQPFPFPRQYNRKLKLIFMLVCFSSLISTTMYTAYLQAFLWGPPIEPRLTSFDDVKKSRYTMAINIYEREFLEALNVSLEDVEIYDYGKFSKLRSTFNTNYLFPVTALQWFTINEEQKLFKYKIFYYCDAFCLNQFDILSIPLRRHLPYRDIFEEHMLLQKEFGLTKYWIDQSYRDMIRANLTTFKDFSPLLENDYIEVHNLYWVFTMYFVGMGMGLCFFILEILRPLRYWRNCKIKCEYCYAFLKNFAK

>DmelIR52d

MVRIIIILLCLGYTKARILDATNTNHTDLEERLLSLLLRLQQEQFFNTLLIYGEDCAFSSLSRRLQVPTILVSSGSTSFEWNYSSLALILTCEFKAEREENYQTLKKLQMNRRLILLNGNIKPDSVCDFYSKKDQYNIAMVNNNFHQVGIIYACRLFQERNYEKVYLSEGNPIYVDQFRNMQGALLKSITFNLIPGSMAYRDPKTGQEKHIGYVANLLNNFVEKVNATLDMQVKLHKAGKKTSFYNITKWASEDLVDIGMSYAAYFEMTNFDTISYPYLMTSTCFMVPLPDMMPNSEIYMGIVDPPVLVVLIAIFCIFSVMLNYIKQRSWRSLSLVNVLLNDICLRGFLAQPFPFPRQSNRKLKLISMLVCFFSVITTTMYTSYLQSFMWGPPIDPKMCSFADLENSRYKLAIRRYDIEMLRPFNVSMDHVVVFDESSQLEYLRDSFDDNYMYPMSALSWSAFKEQQKLFAFPLFYYSEKLCLKPISFFSFPIRRHLPYRDLFEEHMLQQNEFGLSTYWIDRSFSDMVRLKLATMNDFSPPRLEDYIEVSDLSWVFGMYFTGLGISCCCFGLELLGLPSWTRRLRLTNWLRVRN

>DmelIR54a

MWTVITGIVLWAPVLVAGSAVDFIFRAAAEHSLSVIMIRIDYCPYNWAKDIFENQTIPVVVLSDSETFINIRMFSRPLHVACLPGHELQKDLALLENFTSSLMDFPSQKKIVYISNNFSDPTRMDYIFETCYHRRIWNIVGLLASDEHRYFYRYHLYPSFRTEYRSLESSTIFDKDFPNMHGHPLTVMPDQWLPRSVLYVDRRTGKQILAGSVGRFFHVLSWKLNATLQLSKKVTTGRFLNATALKELSESFSVDVPASLTIMERVEQLASTSYPMEVTHVCLMVPVARRIPIKDIYFILSSASNMFLAIVIVSSYGLALNLLRNMTHRDVRLVDFVLNDKALRGILGQSFNLPLSRSFSTRLIFLMLGIVGLNVSSIFGAGLDTLMAHPPRQFQARSFAGLRRTKIPLVTTEEDFPTWMKLRVPMLVVNVSEYNHLRNGRNTSNAYFASRLYWNLFSEQQKRFTRELFIYSTDDCLWSLALLSFQWPQNSLFTEPVSQLILEVNANGLYDFWVGMHYYDMTAAGLSGLEDPSLQLKEREHPTSLRIVDFQWMWQAYGTFMVIAILVFLLEVSWHRITSLFVSLVY

>DmelIR56a

MGSRFFIRNLILFGLLASSNMQIPFGELEKKFELDVDFLLGVTELVGHIQGLYSITVYADCIDIHPSIQQRIMDKFMVPVNTIGSNLSRPNYHKLDNSRIRIVLFTGLNDTILVNLNKTDVPYSDNFYMLAYASAIKNKCIELDFIEEVFTLLWKMSIQNAILLIRGEFMMEMWSYLYMGKIHKIKLTKPNSYLESLRKYNYRFSLEVINDPPAIFWYNSSEQADVTGGGNLSVSGPLGLIIINFLRHLNVTIDIVPIPGKQTSQYELFQQPDNLRAENGVNMVGSALLKYSPMVTQSRMCLLVSNRRMIPFSRFLDRLVSPGVHKLTFVSSIGIFVIKYFSHRPRSFVDAIFCTIRFFFAIPLPSIILNRLPVVDRFIEVFIIIFVQILLSSNISITTSALTTGFWEPPIINVETMRASGLHILTEDPTILQAFKENILPSSLADLVILVDEDTYFHHVTTLNNSYVYVVQAHNWQIFRLYQQQMTNEPFEIASEELCSKWRILGIPLNPKSPLRFMFKDYFYRILESGLREQWVHSGFKKFCEFNNLKKLPVDSVDSWQPLSIEFYSNVIRAYIIGLVIATLAFVAELLHNGYRRKNVKKT

>DmelIR56b

MLLDTDLASGVIRSPYSFDIPHAFIFNETQFVVPKFCGPYMEIVKHFAEVYHYQLFLDSLESLPKKSVVEQDIISGKYNLSLHGVIIRPEETSDFFNATQHSYPLELMTNCVMVPLAPELPKWMYMVWPLGKYIWTCLFLGTFYVALLLRYVHWREPGNATRSYTRNVLHAMALLMFSANMNMSVKLKHASIRVIIFYTLLYIFGFILTNYHLSHMTAFDMKPVFLRPIDTWSDLIHSRLRIVIHDSLLEELRWLPVYQALLASPSRSYAYVVTQDAWLFFNRQQKVLIQPYFHLSKVCFGGLFNALPMASNASFADSLNKFILNVWQAGLWNYWEELAFRYAEQAGYAKVFLDTYPVEPLNLEFFTTAWIVLSAGIPISSLAFCLELFIHRRKQRRPQYERFECYDY

>DmelIR56c

MQHLLNLLAPFGRMNVFQEIVWFVSPHQRLDQLDEFIMRIDEAFGKSATQTVVNNNTEMRMIYSSARRNHMSFVFTTGAEDPIMKVFSKVLLGRHFYVSMVIYVDKVGDMHPIYDLLTFAYNQQFFNSMVHFESMEGVNQLFGVSKFPVMSFENRTDFLKYMGKIWKQVQNARSDVGGFGFTTPLRQDLPHLFQSQGHYDGSTYRIIETFVRFINGSFKELIMPPDSLGGQVINMKDALQLIRERKMEFCAHAYALFMSDEELEKSYPLLVVQWCLMVPLYNSVSTYFYPLQPFDWNVWFFALGALLALVLLELMWLRMFGGWSGYRGAVLNSFCYIINVPIEGQLQQPCLLRFLLLATVFFHGFFLSAYYTSNLGSILTVNLFHAQINTMNDIVSAQLPVMIIDYEMEFLLNLNKELPQEFLELLRPVDSAVFSEHQTSFNSSFAYFVTEDHWEFLDEQQKHLKQRLFKLSSICFGSYHLAFPLQMDSSLWRDIEYFTFRIHSSGLLNFYARSSFGSALHAGLVQRMPDTQEYTSAGLQHLAIAFILLLVMSFLAGIVFVLETLSR

>DmelIR56d

MDNRAAELILRERNIFPTNGSDNITLLNNMFVLEMFYRITQLYHFKNFIFYISERLDLNNKDSQEFFHNFWTYFPMAPNLIITREHHLGIPMMQFISTPSLVMVFTTGKDDPIMELASHNQQGIHWLKTIFVLFPSLQSRDFETNPESLAQFTAEIKDVYDWVWRKQFINTFLITIKDNVFILDPYPTPSIVNKTGVWQAEEFFHKYAKNMKGYLVRTPILYDMPRVFKSDRPTNRYEKNFIHGTSGNLFLGFLEFVNATLMDTSANVTADYLNMTNLLDLVSQGVYETLIHSFTEITTKFVVSYSYPIGINDCCIMVPYRNQSPADQYMHEALQENVWVLISLFTLYITVAIYLCSPLRPRDLSAAFLQSICTLTYSVPTFIIRTPTLRMRYLYILLAIWGIVTSNLYISRMTSYFTTAPPVRQINTVQDVVEANLRIKMLAIEYERMAKSPLQYPESYLNQVDLVDKHMLDLHRDPFNTSFGYTVSSDRWRFLNLQQLHLRKPIFRLTEICEGPFYHVFPLHKDSHMRSVMTEYIMIAQQAGLMNHWERETFWEAVHLHRIHVHLFDDEPMALSLDFFSSLLRTWTLGLILAGLAFAAEMKWHEHVTFKRRPVIRITRKPRSFLRRFMKL

>DmelIR60a

MWCNNPGLIIIIFLGQILNLCQGIVNLSNETANTVIFMLPEKDLGPDVWKAGVGCLDSFAQIFFFRNPKERFTRAYNLMLVHAFHLSSPADQIQEGFSKLINEAVTNPGPPDREELFQMRVASDYNITNGTEDKGELILADNYVIVVDSVDRLKELMKKKIVEMRSWNPGARFLVLFHNATCRNRPLGVASNIFKDLMEMFYVHRVALLYANSTMNYNLLVNDYYSNVNCRILNVQSVGQCHDGKLYPNNAVVKASMQDYVSGFSPRNCTFFACSSISAPFVEADCILGLEMRILGFMKNRLKFDVNQTCSLESRGEMDGPANWTGLLGKVQNNECDFVFGGYYPDNEVADHFWGSDTYLQDAHTWYIKMADRRPAWQALVGIFEAYTWIGFILILIISWLFWFTLVMILPEPKYYQQLSLTAINALAVTISIAVQERPICETTRLFFMALTLYGLNVVATYTSKMIATFQDPGYLHQLDELTEVVAAGIPFGGHEESRDWFENDDDMWIFNGYNISPEFIPQSKNLEAVKWGQRCILSNRMYTMQSPLADVIYAFPNNVFSSPVQMIMKAGFPFLFEMNSIIRLMRDVGIFQKIDADFRYNNTYLNRINKMRPQFPETAIVLTTEHLKGPFFILVVGSCWAALTFIGELIIHRWRTQLVSTSEQQDRRSDKRRRRRRRRKPEKDNRWQRQVQVAPVVRFTPVKRRKVFQGQTSQK

>DmelIR60b

MRRSLYLIIAIGLVDVHCVSLRYILNALENELQYRAILLVESASEIESCWEQKYIQGAVPILNFNANQSLYLKDALNTNILALVCLNENVESTMQALYENLEDMRDTPTILFVLSDSKVQDVFLECLRRKMLNVLAFKGLDRGFVYSFRAFPTFRVIERNVMDILQYFEQQLEDLGGHTLTTLPDNIIPRTVVYKSPDGSRQLAGYLYPFLRNYVSTINATLKVCWHLVPEDGMIQLGEVVRLSEIHDVDFPLGMHGIEHGSTSQNVPLEVSSWFLMLPMEPSLSRAQFFIMLGFEKVTPVLLLLTILLSTAHRIEMGLRPSWRCYVLGDRVLQGTLGQAFFLPRRLSVKLMLVYSLILLNGFTFSNYSITSLETWLVHPPSGHPIHSWEQMRTLNLKVLIVPSELDSMTKALGKQFTESNSDLFELSKSGNFQDKRLAMDQSYAYPVTCTLWPLLEHAQIRLPKPEFRRSREMVLIPLLIMAMPLPKNSMFHKSLNRYRALTHQSGLYEFWFKRSFNELVALRKIHYKVNGDHQIYRDFEWQDFSYVWLGFVGGTIASILVLLAEIGYHRWQLNQN

>DmelIR60d

MRLAIYVAFLSSIGNRSGFLSSLLMSLGKELHYKTILLVGGSSTCWSLEPFETGVPILNLRGENNAYPQDTFNSQMLALACLQTESEDAVKLLYRSLKDMRDTPTLLFASSEEHIHDTLFLGCFRENMLNVLALTASSKEFIYSYQAFPTFRVIKRKLVEIHRYFEPQLKDLGGHIVSALPGNIMPRTMCYRNAEGERQLAGYLNTFIRNYVESINGTLRISWGLVPEDDMRHLTISRLSKIQHVDFPLGIIPLYNKTDKQHVYMEISSWFLMLPMETSVPRAHLFVKLGLERLLPIIVVVGAVLGNAHRIEVGLGPSWRCYYLADKVLRGALAQPIVLPRRLSPKLMLIYSLLLLSGFFLSNYYMASLTTWLVHPPASDRILEWDQLRYLHLKVLTIPEEFKYMSLILGTDFMTAYGSIFQLTNSTDFQRRRISMDPSYAYPVTTSLWPFLELSQVRLRRPLFRRSYDMVLQPFQVMSLPLPRNSIFHKSLLRYAALTRETGLYYYWFRRSYYELVALGKISYKEEEGNPYCDLKWNDFRIVWLAFLGGTIISCLALLLEVAHYRWHLGNSSL

>DmelIR60e

MVIKMISFLLVSVLLCLVGASDSESMQVQVLQDLNLALQTELNVFIDFECCATSEILHKLDSPRILLSSNSREARDLRIRGNFTESTLIIVSVMDSDLNPLVASLLPRLLDELHELHIVFLSNEEPGFPKQDLYTYCFKEGFVNVILMSGKGLYSYLPYPSIQPISLSNVSEYFDRARIIRNFQGFPVRILRSTLAPRDFEYSNEQGGLVRAGYLFTAVKELTYRYNATIESVPIPDLPEYDVYLAVAEMLHTKKIDIVCYFKDFSLEVAYTAPLSIIREYFMAPHARPISSYLYYSKPFGWTLWAVVISTVLYGTVMLHLAARGARVEIGKCLLYSLSHILYNCHQKIRVAGWRDVAIHGILTIGGFILTNVYLATLSSILTSGLYDEEYNTLEDLARAPYPSLHDEYYRSQMKAKTFLPERLRRNSLSLNATLLKAYRDGLNQSYIYILYEDRLELILMQQYLLKTPRFNMIRQAVGFTLESYCVSNSLPYLAMTSEFMRRLQEHGISIKMKADTFRELIHQGIYTLMRDDEPPAKAFDLDYYFFAFVLWTVGLISSLLVFFAELVSGHL

>DmelIR62a

MYLQFLFALFLSRYQIVATENFDRAFELALFLDRIGRVHRLHAITIVNSLGSVDPSYLDDLHRGLMCNSSNHFYMLPQMTATDKDSSHVHFSSLQDEETIYLVFARDSKDAVIYLQAERARGRRYTRTMFLLRKQESQKDIKYFFELLWKLQFRSALVVVAARNFYQMDPYPTVRVIRMRRLSSYDPHHVFPPANRKNFRGYRMRLPVQQDVPNTFWYKNRRTKAWELAGLGGILINQLMMHLNVTMDLFRFEVNGSSLLNMAALTDLIVKGKVELSPHLYDTLQSNTSVDYSYPTQVAPRCFMIPLDNEISRSLYVFLPFSLTMWLCLLFVLLVVHFVYVRRLIPDGHFWAILGVPGAGQVRYGNRKPVRRFSTFLILFGIFILGQTYSTKLTSSLTVTLIRRPDNSLEELFLLPYRILVLPTDVYAIVDSLGHAEQFSTKFSCTDAENFSQKRISMHPEYIYPISTIRWRFFDMQQRFLRKKRFYFSKICHGSFPYQYQLRVDSHLKDALHRFLLHVQQAGLHDLWLDTCYRKAHRMGYLKDFSTLAELEEKLRLRPLALNLLVPAFSLFLCGMLGSGIAFLVEIRHSFGCRQKPPSINRNPGD

>DmelIR64a

MHWWLLVFLPLSCQGLPEHELLELELDYGLAEPQRTSLLQSSLILQFSQDYKHIPRITYFTCQKPHLQTPNQIPNAAEHRDAFAAKNFQLIKSLYESELFVRIVLLDVLAQSPTSGRPNRPGNGPTGGFSQTPSQAQSNSEWLEGVLRMEALRQIAVVDLACGAVSRRFLELASAKMLYSEKFHWLLIEDFAWHGRTQTAEGSGKRDDGEMEEEEPPGQQIQATDDEDLPSIESFLGGMNLYMNTELTLAKRMSEAAHYTLFDVWNPGLNYGGHVNLTEIGSFTPTEGIQLHTWFRTTSTVRRRMDMQHARVRCMVVVTNKNMTGTLMYYLTHTMSGHIDTMNRFNFNLLMAVRDMFNWTFVLSRTTSWGYVKNGRFDGMIGALIRNETDIGGAPIFYWLERHKWIDVAGRSWSSRPCFIFRHPRSTQKDRIVFLQPFTNDVWILIVGCGVLTVFILWFLTTIEWKLVPHDGSALIKPKGGAPPRHHYQQQQQQEQVEAPVRPITAVSVVVSKEKVEEKQEEYEDSTPIDAGTLWQRCYQKLNKYIKDRKAKQKKAPERVGLFLESVLFFVGIICQQGLGFSTSFVSGRCIVITSLLFSFCIYQFYSASIVGTLLMEKPKTIKTLSDLVHSSLKVGMEDILYNRDYFLHTKDPVSMELYAKKITSVPTTKENEADEDEPVDPNPVSTDPAKSYRDIVHSHETGAHAKDNAASNWLDPETGLLRHLGFAFHVDVAAAYKIIAETFSEQDICDLTEVSMFPPQKTVSIMQKNSPMRKVISYGLRRVTETGILTYHFNVWHSRKPPCVKKIETSDLHVDMDTVSSALLILLFSYAITLMILGTEILYSKWHNRIQLKWVGAT

>DmelIR67a

MLPILVPVLLLFNETSWINPILTSIYKDRHHETVLLLQHSQHGNASGLERFPWPVFSFNEQMDFYVRGKYNSEMLVLIWQTGNSDWDLDLWQALDRSLLNMRKVRVLLLRKWEKIPTADVAATAEHLLFLHVAVIGQGNRIYRLQPYAPQSWLQVDPIESPIFIKIRNYFGRYIVTLPDQFPPRSIVYRNPKTDEIQMTGYVYKFLLEFIRIYNFTFRWQRPIVQGERMNLILLRNMTLNGTINLAISLCGFETPSELGVFSDVYDMEEWYIMVPRAQEISIADVYVVMVSGNFLIVLIIFYFIFTILDTCFGPLLLKERVDWSNLMLNERMISGIMGQSFNMSARNTISSKVTNATLFLLGLVLSTLYAAHLKTLLTKRPTSQQISNFKQLRDSPVTVFFEEAERFYLKHAWDRPIRYIKDQLNFRETIEYNALRMGLNRSNAFSALTSEWMIVAKRQELFKQPIFTVQPELRVIQTSVLLSLVMQSNSIYEDHINDLIHRVQSAGIVEYWKHQTLREMITMGMISQKDPFPYVAFREFKVGDLFWIWLLWVSFLFMSFVIFLCELLVDCFISKTLIRNKRPH

>DmelIR67b

MELLYLNTLQSLSLLEGNRLVQTVQELNNIYQTELNVFLEFGNGADILESAQGTFVPTLWIKNPQNQKVMKGNFTSCTLTILYLEDEHLDRGLYYLANWLWEYHHLEVLIFFNGGSYDKLIQIFSRCFNEGFVNVLVMLPGSDELYTFMPYQDLKILNLKSIKEFYSLSRKKMDLNGYNITSGLVIAGAPRWFSFRDRQNRLILTGYMLRMIVDFTNHFNGSVRLMNVLTVNDGLELLANRTIDFFPFLIRPLKSFSMSNILYLENCGLIVPTSRPLPNWVYLLRPYAFDTWIAWLIMLIYCSLALRILSKGQISISAAFLKVLRLVMYLSGSRDMGTRPTTRRLFLFVILTTSGFILTNLYVAQLSSNSAAGLYEKQINTWEDLDKSDSIWPLIDVDIKTMEKLIPDRTKLLKKIVPTLEADVDTYRRNLNTSCIHSGFFDRIDFALYQQKFLRFPIFRKFPHLLYQQPLQISAAFGRPYLQLFNWFVRKIFESGIYLKMKDDAYRHGIQSGLLNLAFRDRHLEVKSNDVEYYYLIAGLWFGGLTLATVCFLLELLIGYAKIKVTISCKMNIM

>DmelIR67c

MFCWLIFLNIILLSDRSESWSAREVIHQFNHDQQLQLNIYLDCNDVELQIGQEVSNLFVNSTADKMKILGRFSSHSLIIACFKDSTRNRTLNGVKELLWGLQYLPILFVVDSNMDFYFQQALRHGFIHVLALNFMNGSLYTYKPYPKVEVHQIKDMQKFYKLTKLRNLQGQAVRTTVETMTPRCFRYRNRHGQLVYAGYMYRMVKEFISTYNGTEEHVFGNVDTVPYKEGLAALKNGEIDMMPRIIHALEWYYFYRSHILYNIKTYIMVPWAEPLPKSLYFIQPFRGTVWITIMVSFVYASIVIWWIRYRQQGNSSLTQSFMDVLQLLFQLPLSKIWHFNMGTHQVVSFIVLFVFGFMLTNLYTAQLSSYLTTGLFKSQINTFDDLFREKRTLLVESFDAEVLHNMTKEKIIQKEFESIILITSIEEVFKHRKSLNTSYAYEAYEDRIAFELSQQRYLRVPIFKILKEVYDQRPVFVALRHGLPYVELFNNYLRRIFESGIWIKLQEDSFLEGIASGEISFRKSKSREIKIFDKDFYFFAYILLGMGWCVSTIALFLELWSFKYSVTNVLHEG

>DmelIR68a

MRCLWILIVAFISLAMATSIPIPIANPAPLSGYEMQLKILLQKILWVANVKRCFAVITDDLHYPIYDRIFFESVGRRVIPFFVMRTNESDDLQRPSRQVELFVKAIKSSDCELNVITILNGWQVQRFLGYIYDNRSLNMQKKFVLLHDLRLFESDMIHLWSVFIDAIFLKRQLDNKYTISTIAFPGILSGVLVMKNIANWELGKGLNGRILFADKTSNLFGTSLPVAISEHVPMVLWANATKSFQGVEVEIMNALGKALNFKPVYYKPNQTENMDWTELDGGASVAYGSGNPDGYAQNGTHIDSMLVDEVAAHSARFAIGDLHLFQVYLKLVELSAPHNFECLTFLTPESSTDNSWQTFILPFSAGMWVGVLLSLFVVGTVFYAISFLNAIINGNVSSEFFRCLRPNRNVPMDPKIYRRISFRIAISRYRSSKGDRMPRDLFDGYTNCILLTYSMLLYVALPRMPRNWPLRVLTGWYWIYCILLVATYRASFTAILANPAARVTIDTLEDLLRSHIPPSTGATENRQFFLEANDEVARKVGEKMEVFGYSDDLTSRIAKGQCAYYDNEFYLRYLRVADESGSALHIMKECVLYMPVVLAMEKNSALKPRVDASIQHLAEGLIAKWLKDAIEHLPAEALAQQEALMNIQKFWSSFVALLIGYVISMLTLLAERWHFKHIVMKHPMYDVYNPSLYYNFKRIYPQH

>DmelIR68b

MKFLVGLLLQWYLPGIYALAEIACRIAVEQNVQVTYLYRCASCPASFDADYSALELDLYRCVGSRLPVITRNMEAHELEPFRRTDSLSIFQIPAAEKGDSLVRRILDMLNPHQRRKHMHKYLFVWPNAGRHQLLRLFRGSWAKKLLYGLAITGRENGTFDFDPFAWGGLQVIQRLDGEVPYARKVKDLRGYPLRFSMFTDPLMAMPRSPVETAGYQAVDGVAARVVGEMLNASVTYVFPEDNESYGRCLPNGNYTGVVSDIVGGHTHFAPNSRFVLDCIWPAVEVLYPYTRRNLHLVVPASAIQPEYLIFVRVFRRTVWYLLLVTLLVVVLVFWVMQRLQRRIPRRGVIQFQATWYEILEMFGKTHVGEPAGRLSSFSSMRTFLMGWILFSYVLSTIYFAKLESGFVRPSYEEQVDRVDDLVHLDVHIYAVTTMYDAVRSALTEHQYGLLENRSRQLPLGIATSYYQPVVRRRDRRAAFIMRDFHARDFLAITYDSQAERPAYHIAREYLRSMICTYILPRGSPFLHRLESLYSGFLEHGFFEHWRQMDLITRVGASPDAEEFLEDLGDQTDTDSGSNELAIRNKKVVLTLDILQGAFYLWSVGIGISCLGFAVEHAHWFWRRQTLRNAVEARTS

>DmelIR75a

MQLVQLANFVLDNLVQSRIGFIVLFHCWQSDESLKFAQQFMKPIHPILVYHQFVQMRGVLNWSHLELSYMGHTQPTLAIYVDIKCDQTQDLLEEASREQIYNQHYHWLLVGNQSKLEFYDLFGLFNISIDADVSYVKEQIQDNNDSVAYAVHDVYNNGKIIGGQLNVTGSHEMSCDPFVCRRTRHLSSLQKRSKYGNREQLTDVVLRVATVVTQRPLTLSDDELIRFLSQENDTHIDSLARFGFHLTLILRDLLHCKMKFIFSDSWSKSDVVGGSVGAVVDQTADLTATPSLATEGRLKYLSAIIETGFFRSVCIFRTPHNAGLRGDVFLQPFSPLVWYLFGGVLSLIGVLLWITFYMECKRMQKRWRLDYLPSLLSTFLISFGAACIQSSSLIPRSAGGRLIYFALFLISFIMYNYYTSVVVSSLLSSPVKSKIKTMRQLAESSLTVGLEPLPFTKSYLNYSRLPEIHLFIKRKIESQTQNPELWLPAEQGVLRVRDNPGYVYVFETSSGYAYVERYFTAQEICDLNEVLFRPEQLFYTHLHRNSTYKELFRLRFLRILETGVYRKQRSYWVHMKLHCVAQNFVITVGMEYVAPLLLMLICADILVVVILLVELAWKRFFTRHLTFHP

>DmelIR75b

MLQLHNLILHNLIHMAKLSHVLILHCSLSHLALLAQSKNIFTQFQPLHSDIQLNDDFLNHNILKLGVFLDINCDKSGTVLDMASAKRFFSHRYHWLIYDRSMNFSVLESHFKEAQIFVDADVTYVTHDPFSKNFLLYDVYNKGRQLGGELNITADREIFCNKTNCRVERYLSELYTRSALQHRKSFTGLTMRATAVVTALPLNVSIKEIFDFMNSKYRIQLDTYARLGYQARQPLRDMLDCKFKYIFRDRWSDGNATGGMIGDLILDKADLAIAPFIYSFDRALFLQPITKFSVFREICMFRNPRSVSAGLSATEFLQPFSGGVWLTFALLLLLAGCLLWVTFILERRKQWKPSLLTSCLLSFGAGCIQGAWLTPRSMGGRMAFFALMVTSYLMYNYYTSIVVSKLLGQPIKSNIRTLQQLADSNLDVGIEPTVYTRIYVETSEEPDVRDLYRKKVLGSKRSPDKIWIPTEAGVLSVRDQEGFVYITGVATGYEFVRKHFLAHQICELNEIPLRDASHTHTVLAKRSPYAELIKLSELRMLETGVHFKHERSWMETKLHCYQHNHTVAVGLEYAAPLFIILLGAIILCMGILGLEVIWHRHCTLH

>DmelIR75c

MTSWPLYRLIVFNLLEINLSNLMVFHCWSIKEAFPLVEMLNQNGIFSQYIDVQNPDNLANVHKEYLDSDLVRLGVFLDLGCDKAELVTNQSSRARLYNQNLHWLLYDEAGNFTKLTQLFEGANLSLNADVTYVSREDEERFILHDVYNKGSHLGGKLNITVDQTLQCNRSHCQVKEYLSELHLRPRLQHRMDLSSVTFRLAALVSVLPINSSEEELLEFLNSDRDSHMDSISRIGNRLIMHTQEILGFKLHYIWCGTWSVQDAFGGAIGMLTNESAELCTTPFVPSWNRLHYLHPMTEQAQFRAVCMFRTPHNAGIKAAVFLEPFMPSVWFAFAGLLIFAGVLLWMIFHLERHWMQRCLDFIPSLLSSCLISFGAACIQGSYLMPKSAGGRLAFIAVMLTSFLMYNYYTSIVVSTLLGSPVRSNIRTIQQLADSSLDVGFDTVPFTKTYLVSSPRPDIRSLYKQKVESKRDPNSVWLSPEEGVIRVRDQPGFVYTSEASFMYHFVEKHYLPREISDLNEIILRPESAVYGMVHLNSTYRQLLTQLQVRMLETGITSKQSRFFSKTKLHTFSNSFVIQVGMEYAAPLFISLLVAYFLALLILILEICWARYAKKKFSTIIPQNQ

>DmelIR75d

MKVQVAHWLPLIFFLLVSGTPRVAGSWRSEYSRQDPDPKTRWGNQLPDMLVAYYRHHGVHSLMLVVCHTDIADFRLWKLWQHFNLNNFYVQVSTESSLRDLQHVDALDEHKDAPPPKSFHANNSTHWETSFLLPALPYKMGILLLEFSSECALNLLRWSAASEHNYFTTNRFWLLLTEDPGDIDLLEDPEIFIPPDSELRVLHYENVGNFSCSLIDLYKVAAWKPLKRTLVGHNIRNSRHVIHALQHFGSAITYRQDLEGIVFNSAIVIAFPDLFTNIEDLSLRHIDTISKVNHRLMLELANRLNMSYNTYQTVNYGWRQPNGSFDGLMGRFQRYELDLAQLAIFMRLDRIALVDFVAETYRVRAGIMFRQPPLSAVANIFAMPFENDVWVSILMLLIITTVVLVLELFFSPHNHDMSYMDTLNFVWGAMCQQGFYVEVRNRSARIIVFTTFVAALFLFTSFSANIVALLQSPSDAIQSLSDLGQSPLEIGVQDTQYNKIYFTESTDPVTKNLYHKKIASKGENIYMRPLLGMEKMRTGLFAYQVELQAGYQIVSDTFSEPEKCGLMELEPFQLPMLAIPTRKNFPYKELIRRQLRWQREVSLVNREERKWIPQKPKCEGGVGGFVSIGITECRYALGIFGCGAAVSFVLFLFEFIFRHFKQVYRIIKGYREVQR

>DmelIR76a

MENLLVESYYFSTVLSFFAQQFFADSHATCIFWHPAFDFRLETVHPMPLIIMDWHRWANRSDQDVYDYKIKEDEFEGKGIPYNDWTLRLTVAIERSHCETFIAFQEQIPEFARYFYHASIYSIWRSLRNRFMFVYTKEFEDKKDSYLSGYIFQDQPNILVITSQYLNSSTFEIKTNRFVGPRNFNKNPEPVEFYILQRFDAKGTKATWETQSAMSSKMRNLKGREVVIGIFDYKPFMLLDYEKPPLYYDRFMNTTDVTIDGTDIQLMLIFCELYNCTIQVDTSEPYDWGDIYLNASGYGLVGMILDRRNDYGVGGMYLWYEAYEYMDMTHFLGRSGVTCLVPAPNRLISWTLLLRPFQFVLWMCVMLCLLLESLALGITRRWEHSSVAAGNSWISSLRFGCISTLKLFVNQSTNYVTSSYALRTVLVASYMIDIILTTVYSGGLAAILTLPTLEEAADSRQRLFDHKLIWTGTSQAWITTIDERSADPVLLGLMEHYRVYDANLISAFSHTEQMGFVVERLQFGHLGNTELIENDALKRLKLMVDDIYFAFTVAFVPRLWPHLNAYNDFILAWHSSGFDKFWEWKIAAEYMNAHRQNRIVASEKTNLDIGPVKLGIDNFIGLILLWCFGMICSLLTFLGELWRGQG

>DmelIR76b

MATGIELLVAAALCVACPPLNDSPPTNLIQMGENGTLSPVTELPMDVDASEAGFDADAPVETLETINRKKPKLREMLDWIGGKHLRIATLEDFPLSYTEVLENGTRVGHGVSFQIIDFLKKKFNFTYEVVVPQDNIIGSPSDFDRSLIEMVNSSTVDLAAAFIPSLSDQRSFVYYSTTTLDEGEWIMVMQRPRESASGSGLLAPFEFWVWILILVSLLAVGPIIYALIILRNRLTGDGQQTPYSLGHCAWFVYGALMKQGSTLSPIADSTRLLFATWWIFITILTSFYTANLTAFLTLSKFTLPYNTVNDILTKNKHFVSMRGGGVEYAIRTTNESLSMLNRMIQNNYAVFSDETNDTYNLQNYVEKNGYVFVRDRPAINIMLYRDYLYRKTVSFSDEKVHCPFAMAKEPFLKKKRTFAYPIGSNLSQLFDPELLHLVESGIVKHLSKRNLPSAEICPQDLGGTERQLRNGDLMMTYYIMLAGFATALAVFSTELMFRYVNSRQEANKWARHGIGRTPNGQSVAPSRWLRGWRRLNSGHGQLLGASTHGQNVTPPPPYQSIFNGGSHGDPLNRWRRPLANGNALGNGVLLGGDSEGGVRRLINGRDYMVFRNPNGQSQLVPVRSPSAALFQYSYTE

>DmelIR84a

MIKLQVKVISWPLIILTAFLRVLQIESINTNFLELAAFEDFLRSEHLSHVLVVRGDDADGDWKIECHQKLLANYRVQFYRPEMSANFEDLMFYGSPRTAVLVLNSEHVLVRRQVFGVASEAGYFNNSLAWFILGSGRESLPVEQLIDQLLSGYRMGIDADITVALRGPDNASMLFYDVYRISRQANTPLIIEKKGLWTHSGGYQKFGNFKNTWVIRRRNFLNVTLIGSTVLTEKPPGFGDMEYLADDKQLQQLDPMQRKTYQLFQLVERMFNLSLAISLTDKWGELLDNGSWSGVMGQVTSREADFAVCPIRFVLDRQPYVQYSAVLHTQNIHFLFRHPRRSHIKNIFFEPLSNQVWWCVLALVTGSTILLLFHVRLERMLSNMENRFSFVWFTMLETYLQQGPANEIFRLFSTRLLISLSCIFSFMLMQFYGAFIVGSLLSESARSIVNLQALYDSNLAIGMENISYNFPIFTNTSNQLVRDVYVKKICKSGEHNIMSLQQGAERIIQGRFAFHTAIDRMYRLLLELQMDEAEFCDLQEVMFNLPYDSGSVMPKGSPWREHLAHALLHFRATGLLQYNDKKWMVRRPDCSLFKTSQAEVDLEHFAPALFALALAMVASALVFLLELFLHWLPDFRRRLGTMST

>DmelIR85a

MSIQWLKHILLLAILVNLAGTRENHIPLDLKKSSIVMVKMSQILCKARIKVLFVYFENQTSHEHTGQILKEVTKCDISNQNTPLEAVKDDGILMYMVMITTNISQPLELSLIRKKSAAKHRSHVFLLVRDADTVSDAWMRASFRQFWKIWLLNIVILYWRDGRLNAYRYNPFMDNYLIPVDNKPNEVPTLEQLFPKTIPNMQRKPLRMCIYKDDVRAIFWRQGTILGTDGLLAAYVAERLNATMMITRPHSYNNHNLSSDICFLEVAKEYVDVAMNIRFLVPDTFRKQAESTVSHTRDDLCVIVPKAKTAPTFWNIFRSFGSLVWALILVSVLVANVFCYILKSEVGRVPMQLFAGALTMPMTQIPPNHSIRLFLIFWLYFGLLICSAFKGNLTSMMVFQPYLPDINQLGALARSHYHIIIRPRHVKHIQHFLTLGHKHESRIREQMLEVSDTQMYEMMRNNDIRFAYLEKYHIARFQVNSRVHMHLGRPLFHLMNSCLVPFHAVYIVPYGSPYLGFLDSLIRSSHEFGFERYWDRIMNSAFIKSGVKVVNRRRGSGNDEPVVLKLQHFHAVFALWLVGIGMACIVLAWEHLTHNYNLAVTKRRD

>DmelIR87a

MSTPEQRFWLAALLFLLSQHSEVRGFGINLMKVQTEDKGQEACILALLRKYFDSGDGLSGSVLCINRNYQLPNIEEQLLRGVNNYENYPWSLLITNSREGPSPAKFLMNEKPQCYFLIVDNLEDEDLDEVFEHWKGMVNWNPLAQFVVYLASLEETDEEMNDLMVELLLTFINKKIFNVNVIGQSEENQFYYGKTVFPYHPDNNCGNRVISVELLDACDYPSEETDSEDENDEDEGDGAQEEDDGPQEEGDGEQEEEDGPQEQEDGDQAKGDEGQENDDGGLENKVENEFRIGASDDDELENDLSSNSSEPEAIIEEFFRAKFEDKFPRDLSGCPLTASFRPWEPYIFRNSEEQPVDDYYYGLQGDEDDYNDTSPNYGESDDESYADPGEDGDGAIPDTETQSGGKLKLSGIEYEMVQTIAERLHVSIEMQGENSNLYHLFQQLIDGEIEMIVGGIDEDPSISQFVSSSIPYHQDELTWCVARAKRRHGFFNFVATFNADAGFLIGIFVVTCSLVVWLAQRVSGFQLRNLNGYFPTCLRVLGILLNQAIPAQDFPITLRQLFALSFLMGFFFSNTYQSFLISTLTTPRSSYQIHTLQEIYSNKMTVMGTSEHVRHLNKDGEIFKYIREKFQMCYNLVDCLNDAAQNEHIAVAVSRQHSFYNPRIQRDRLYCFDRRESLYVYLVTMLLPKKYHLLHQINPVIQHIIESGHMQKWARDLDMRRMIHEEITRVREDPFKALTFDQFRGAIAFSGGLLLVASCVFAFELCYVKYVYRTEKRERKTKKITKKVHNIKIQHD

>DmelIR92a

MLLQPLVMHLSQLLRIIVGQYFAEFPSILIVYNNSASTTPLQLEYLSALELVLRELSKPIRLQWINVAFLKDLNDLEDQVMGALNSSVTEGFITILSQTHHFIHARYYATRNANVRLKDKRYLFLCEDESPAELLCMDILQFYPHHLMVRPGTETAPTGPTGPHPDPRRGGGASVSTKNKDDGEGGAGNKTTSPYRDINFELWTQKFVGAVGNLDALLLDAFLPNETFANRVELYPNKLLNLQRRSLLVGSITYVPYTITNYVPAGQGDVDPIHPQWPNRSLTFDGAEANVMKTFCQVHNCHLRVEAYGADNWGGIYDNESSDGMLGDIYEQRVEMAIGCIYNWYDGITETSHTIARSSVTILGPAPAPLPSWRTNIMPFNNRAWLVLISTLVICGTFLYFMKYVSYRLRYSGTQVKFHHSRKLEKSMLDIFALFIQQPSAPLSFDRFAPRFFLATILCATITLENIYSGQLKSMLTFPFYSAPVDTIEKWAQSGWKWSAPSIIWVHTVQSSDLETEQILARNFEVHDYSYLSNVSFMPNYGFGIERLSSGSLSVGDYVSTEALENRIVLHDDLYFDYTRAVSIRGWILMPELNKHIRTCQETGLYFHWELEFIDKYMDKKKQEVLMDLANGHKVKGAPQALDVRNIAGALFVLAFGVAFAGCALVAELLIHRMDLSK

>DmelIR93a

MNPGEMRPSACLLLLAGLQLSILVPTEANDFSSFLSANASLAVVVDHEYMTVHGENILAHFEKILSDVIRENLRNGGINVKYFSWNAVRLKKDFLAAITVTDCENTWNFYKNTQETSILLIAITDSDCPRLPLNRALMVPIVENGDEFPQLILDAKVQQILNWKTAVVFVDQTILEENALLVKSIVHESITNHITPISLILYEINDSLRGQQKRVALRQALSQFAPKKHEEMRQQFLVISAFHEDIIEIAETLNMFHVGNQWMIFVLDMVARDFDAGTVTINLDEGANIAFALNETDPNCQDSLNCTISEISLALVNAISKITVEEESIYGEISDEEWEAIRFTKQEKQAEILEYMKEFLKTNAKCSSCARWRVETAITWGKSQENRKFRSTPQRDAKNRNFEFINIGYWTPVLGFVCQELAFPHIEHHFRNITMDILTVHNPPWQILTKNSNGVIVEHKGIVMEIVKELSRALNFSYYLHEASAWKEEDSLSTSAGGNESDELVGSMTFRIPYRVVEMVQGNQFFIAAVAATVEDPDQKPFNYTQPISVQKYSFITRKPDEVSRIYLFTAPFTVETWFCLMGIILLTAPTLYAINRLAPLKEMRIVGLSTVKSCFWYIFGALLQQGGMYLPTADSGRLVVGFWWIVVIVLVTTYCGNLVAFLTFPKFQPGVDYLNQLEDHKDIVQYGLRNGTFFERYVQSTTREDFKHYLERAKIYGSAQEEDIEAVKRGERINIDWRINLQLIVQRHFEREKECHFALGRESFVDEQIAMIVPAQSAYLHLVNRHIKSMFRMGFIERWHQMNLPSAGKCNGKSAQRQVTNHKVNMDDMQGCFLVLLLGFTLALLIVCGEFWYRRFRASRKRRQFTN

>DmelIR94a

MALPKQLKFINIFLVLLIIYGSSDGTENQHEIFLNRLLQAVHNERSVETLFLLHHSNLANCSLQDWNPPRIPTIRSNELTVFNVEKTFNHNALALVCLMKNSYREILNTLAKSFDCMRQERIILMIHRKSDSKFIEDITHEVKNLQFLHLIVLIVQEKYNGQVFASTLRLQSFPEPHFKRIRNVFAIQRIFYRPINFHGKVLNAIPNDIPILFVALNEMFTEYARRYNSTLRIQNRTIKEDIEITEDNYDIDMKIQLHNSQNFLHHMNIAMDIGSNSLIILVPCATELRGLDIFKELGVRTLTWLALLFYIIFVLVEMLFVFISNRFNGRNFTMRYTNPLINLRAVRAILGQTSPISNRYSLSIQHFFVFMSLFGTLFGGFFDCKLRSFLTKRPYYSQIENFSELRKSGVTVVVDHTTRQFIEQEINANFFRDEVPNVRTTTIQELINHVYSYDRKFAFVANSIPWRTFREEMKSINQKILCDSKNLTILENVPLTFSIRRNAIFSHHLRNFIINAADSGMITCWFKMAGKVIRKHIKTTLRESEQQPSHLPLSFDHFKWLWAVLCIAYVMSFMVFVMEILWSKYQRRTRSVSIV

>DmelIR94b

MSLIFNLLFILILSQAVSQETEFLQLKYLNNIVRSMIKLHKMETLVIVKHHLDNNCSLQNWNAHGMGIIRTNDQGKLIMKDTFNSRTLAIICIGQNSHITLLRNVFETFGKVQQKKIILWTQMELKEKFFQEISKKSRDLKLLNLLVLKAVTKDKLLIYRLNPFPSPHFKRIENIWTPNDTLFMDTKFNFHGMTAVVKHDYNWTIQMGNIRKFPISRIEDKEVIEFALKYNLTLQFFNDVERFDIELRKRIILKSNSTQPIDSGIPMVFSSLLIVVPCGNYLSIQDVIKVSGIEKWIFYIILVYVIFVLIEITFLGVTILISRQSRHQMIPNTLVNLCAFRAILGLPFPETRRTSLSLRQLFLAIALFGMIFSIFINCKLSSMLTNPCPRPQVNNFEELKTSGLTVVMDHDAENFIEKEIGVDFFNQYMPRKVTLTFTERAKLLFSLKGNHAFTLFSESFAIIESYQRSKGLRAHCTSEDLIVAERVPRIYILENNSILDRPLRRFIRQMQESGITNHWLKNIPSSLEKNLMQITIPYDRERVHPLSIEHLTWLWCILILGYSISMIVFFVEMSLKRRKKNLENRAPNICIC

>DmelIR94c

MSKVFKLLVLPLIYLSLTKGSKNPQLKFLRELINVIEEGREIRTIMVIKHSRDEYCHLDQWNPRGSPILRTNEMGSIRISGYFNDQAVILACMGENSDYGLLKSLANAMDNMRQERIILWSEREPTKMLMDYISQQADRYNFAQIIIVTMNEDVDAVPSLHQLNPYPTPRFRQITNISNIRRTSFFGCGLSFQGKTAILKESVVSNIRFKVWSPSGPIPLSELKDYEIVQFAVKYNLSLKLYDQNESKSDHFDIQLGPLFITKDFPTQMAFVSPNTACSLIVIVPCSPKWRFMDVLHKLGVLKLIGCLLIAYAVFVLIETLILWLTHRISGREVRLTSLNQLLNPRAFRGILGLPFPEFRRSSISLRQLFLVISVFGLVYSNFVSCTLSALLTKPAQNPQVRNFKELRDSGLITIMDKYTHSFIEKHIDPEFFDHVLPHYLILQKKEALRMIWNFNDSYSYVMYTTTWKSLNTVQKSFDERVFCESESLTIAWNLPRMYVLGNNSVLKWMLSRYITYMPQTGIPDSWTEQLPKVLKLLYNVTSPRRIKEGAVPLSIQHLSWIWHLLFIGESIATLVFIVEILLQKSNQHTSNMRERSSEDDDFV

>DmelIR94d

MGQLHLLLVALVLLSPGGDSFYHSLIHHLNRELKIEYVLLLGNFDTTWLDILWQLPVSVLQIKEHSRETYSLLENPSHNVLTIAFVNDSPEDILEILYRNLRMLNTQPVLLVIRKSTIRVNSLLEWCWHHQLLKVVAIAQDFMESLIVYSYNPFPVLQFIERRLDNSTVIFEKRLENLHGYEVPIALGGSSPRLIVYRDLEGKLIFSGPVGNFMKSFEQRYNCRLVQPYPFDESAISPARDLIASVQNGSVQIALGAIYPQVPYTGYSYPIELMSWCLMMPVPEEVPHSQLYSMVFSPMAFGITIVAMVLISLTLSMALRLHGYRVSFSEYFLHDSCLRGVLSQSFYEVLRAPALIKAMYLVICLLGLLITSWYNSYFSTFVTSAPRFPQLTSYESIRHSNIKIVIWKPEYEMLLFFSENMEKYSSIFQLQEDYKEFLHLRDSFDTRYGYMMPMEKWSLMKEQQRVFSSPLFSLQDDLCVFHTVPIVFPMVKNSIFKEPFDRLILDVTATGLLSRWRDMSFTEMIKAGQLGLEDRGHPKEFRAMKVGDLIQIWRFVGWMLGLATIVFLLELICFWRHKMWQNMKYMFCRNKNI

>DmelIR94e

MDCPKWILSGLCLISLVSGATVIELLGTLKLELDFEYVLLMKNRNFSLSDQVWNGTSLTKDVMDEVQVPVLQFNENVSYFLHNSISRRLVTLGFMSDANLDEHRGLLTALVANLRHMTTSRVIFLVQSKASTDFLYELFRNCWRKKLLNVIVIFQDFETTSTFYSYSNFPILQIEERIYETSLQTLPIFPDRLRNLHGYEMPVILGGTAPRMIAYRNKKGNVVYDGTVGHFMTAFQQKYNVKFVQPLQAKNPLDFAPSMQTVGAVRNETVEISISLTFPTIPPFGFSYPYEQMNWCVMLPVEADVPPFEYYTRVFELAAFLLTLGTLVLISCLLASALSLHGYATNISEFLLHDSCLRGVLGQSFVEVFRAPTLVRGIYLEICVLGILITAWYNSYFSSYVTSAPKQPPFRTYDDILASKLKVVAWKPEYAELVGRLLEFRKYETMFLVEPDFNRYLALRDTLDTRYGYMITTNRWVLINEQQKVFSRPLFQKRDDFCFFNNIPFGFPLHENSVFMEPVQKLIMELAETGLYYHWITTGFSELIDAGEMHFVDLSPHREFRAMQIQDLQYVWYGYAFMVVLSSLVWLLENLAYTVKSKTIFPTHFMQRNKK

>DmelIR94f

MWQQVLLAETSNWFRSDVLQRFWTHLRVEIRFRTMLNYRLESCDCWFDNVLGSDNSTALLWNDQTYPHYLRRRQDTDILVVSCLRFHQYQEVLLALSLMLDQMRSMPVVLQLCGDEDSMQELNSARLLLKHSQDLKMPNVVLLSSTFFTSATLYSYEMFPEFNVQKLVYQAYLTLFPYKLGNLKGHPIRTVPDNSEPLTIVRKTLNGSIAIDGLVWQFMIEFAKHINATLQLPIEPHPEKSIKLVQILDLVRNQTVDIAASLRPYSLNVQRSSTHIYGSPMMVGNWCMMLPTERVIGSHEALTRLMKSPWTWLILLLFYSVHRFLAQKTRLRSSLIHLIKLLINLSLICFLQAQLSAYFIGPQKVNHISNMQQVEESGLKIRGMRGEFMEYPIDMRSRYASSFLLHDLFFDLAQYRNSLNTSYGYTVTSVKWELYKEAQRHFRRPLFRYSEEICVQKLSLFSLIQQSNCIYCYRSRIFILRMHEAGLIRLWYRRSYYVMVTAGRFPIGDLSTVHRAQPIRWTEWQNVVLLHGVGLLFSVVVFVIELTVHYANVCLNNL

>DmelIR94g

MSTAVNSVHSKLVSLISRGQELTSIFFYAPAKEKCHLEDTISSATWGLPLVIWRTDRTVILNGFIGEGLLVLACLPGFHWRALLGSLARSLKYLRQARILIELMQDRDEFLVSEVLQFCLSQDMINVNAIFDDFPETENLSSFEAYPSFEVVNQTFTPDTQVSDLYPNKMLNLRGGVIRTMPDYSEPNTILYQDKEGNKEILGYLWDLLEAYAHKHNAQLQVVNKYADDRPLNFIELLDAAQSGIIDVGASIQPMSMGSLSRMHEMSYPVNQASWCTMLPVERQLHVSELLTRVIPYPTLALLLLLWIFYEVLRGRWRRHSRLQSIGWLVLATLVSSNYVGKLLNLFTDPPSLPPVNSLAALMESPVRIISIRSEYSAIEFTQRTKYSAAFHLALHASILIGLRNAFNTSYGYTITSEKWKIYEEQQKRSSKPVFRYSKDLCFYEMIPFGLVIPENSPHRAPLHSYTLLLRQAGLHDFWVNRGFSYMVKAGKINFTAVGERYEAKTLTITDLRNVFIIYVSVLLISLILFTCELFVSWVNYWLGF

>DmelIR94h

MLSNISFSSAPELVDLYGLVLKFLVSSETTLFYFNPTGQKCSWETLPRTILSNHPQIIWFREETYPGLYKRHSSNLFVMACLSSTSYDGQLQLLAESLTRYRSVRVLIEVQDKEGSFLASQILLLCQQHSMLNVVLYFSRWTRTLNVFSYLAFPYFKLLKQRLSGSLRPKIFINQLKDLQGYKIRVQPDLSPPNSFSYRDRHGECQVGGFLWRIVENFSKSLKGDTQVLYPTWAKAKVSAAEYMIQFTRNGSSDIGVTTTMITFKHEERYRDYSYPMYDISWCTMLPVEKPLSVEILFSHVLSPGSALLLILAFILFFLIVPQLIKCLGITFRGRLIGMASRIFALVMLCSSSAQLLSLLMSPPLHTRIKSFDDLLTSGLKIFGIRSELYFLDGGFRAKYASAFHLTENPNELYDNRNYFNTSWAYTITSVKWNVIEAQQRHFAHPVFRYSTDLCFSSETPWGLLIAPESFYREPLQHFTLKINQAGLITQWMTQSFHEMVRAGRMTIKDYSRTNLMKPLRIQDLRKCWVIFAVGLGTSTVVFTIELLLIYTNVFLNSL

>DmelIR100a

MATTLQLIMLALVGGTLGQANNTDHKQVLTSIVKQLEGGLELHLRTSEDGGNDLVQFLMQEKSSIIISAKQEEVPSRAKIMRHHFFIFDGVHQMQEIRTSLFNTDGFYILALENNTIEDDVLLMEFAADVWLQHGHSRIYYVQLSKKSVLLFNPFLQRLVVVQDSKTYSRIYKDLEGYHLRIYIFDSVYSSVIGDGENKVLSVTGADAKLAKTVARQLNFTADFVWPDDEFFGGRLANGEYSGGVGRAHRGEVDIIFAGFFIKDYLTTHIQFSAAVYMDELCLYVKKAQRIPQSILPLFAVHMDVWLCFLLVGLLGALVWLILRAVNLILGIEGVPDGSRATRISYFGAARRIFVDTWVIWVRVNVGRFPPFHSERIFVASLCLVSVIFGALLESSLATVYIRPLYYRDVNTLRELDESGQPIYIKHPAFKDDLFYGHNSEVYRRLDAKMMLVAEGEERLIEMVSKRGGFAGVTRSASLQLSDIRYVMTKKVHKIPECPKNYHIAYVLPRPSPYLEEVNRIVLRLVAGGIVGLWTGEAKERAKWSIQRFPEYLAELDVGRWKVLTLSDVQLAFYALTIGCLLSAIVCMAEILLGRQRRLHSPK

>LmigIR1

NASWRFTVDAEHEWGEIWDNNGSGNGILGAVVRDAADAGFGALYQWLHEYLFLDFSRPYIRTGITCLAPRPRPGWQVPLLRPSPPLWAAVTASVLLATVALFAARRSSDRLLGEDAVRAAAGSRYSTVEDCFFRSVGLLVLQTPDVERRHTRVVGPTRHVLSWLLIAYLLVTASYGSGLSSVLTVPKYEPPIDSVRDLYESGIEWAATHEAWVFSLREATQPVISDLYRRFRVHKEDVLHARTVARDLAYSIERLPAGYFAIGSYIDEASRWLRPMREDIYWERSNVVVRKGWPHLPRLDALIDRLLDSGLLLAWEGQV

>LmigIR2

VSSIFALRSIEIIPDILVARQLDDVDSFELVTMKFTGEETWRDELVLARWRRGHGFDPPTVDLFPDRMADMEGRQLVFATIDYPPYVILKSDSNILDGVESRILLEKLFVVGWQKNATWRVVDHAVDRWGTIWENGSGNGLLGAVATGAADAGFAAVYRWFPEFLWVDYSRPFLRAGIACLAPRPPEVPLLPFAPPLWAAVAASVLLATIALYTARITSNWVLGDVQATGRYSTVEDCFFRSVGLLVLQTPDLERRHTRVVGPTRHVLSWLLIAYLLVTASYGSGLSSVLTVPRYDRPIDTVSDLHESGLEWAERHLVYLYSIRELTDQIYVDLIDLFRVLTSDVLHSRTTTRDLAFVIERLPGGYFTIGDYIDEEAASRWLRPMREDIYWEYVVFAVPKGWAYLQRLDDLIDRLLQAGIMYAWEGQVAHKWLVPRVQLAAQIGMRSYAQSPDGPIQLQLTHVQGEFALLGLGLCLALVVLLVEVAV

>LmigIR3

FELVTMKFTGEETWRDELVLARWRRGHGFDPPTVDLFPDRMADMEGRQLVFATIDYPPYVILKSDSNILDGVESRILLEFVSKKNATWRVVEHAVDRWGTIWENGSGNGLLGAVAAGAADAGFAAVYRWFPEFLWVDYSRPFLRAGIACLAPRPPDIKGGFEPPTAYSLTLRIIAPQCTRPLPRAPCPPWSDMSVQYTISSSYSGRYSTVEDCFFRSVGLLVLQTPDLERRHTRVVGPTRHVLSWLLIAYLLVTASYGSGLSSVLTVPRYDRPIDTVSDLHESGLEWAERHLVYLYSIRELTDQIYVDLIDRFRVLTSDVLHSRTTTRDLAFVIERLPGGYFTIGDYIDEEAASRWLRPMREDIYWEYVVFAVPKGWAYLQRLDDLIDRLLQAGIMYAWEGQVARKWLVPRVQLAAQIGMRSYAQSPDGPIQLQLTHVQGEFALLGLGLCLALVALLVEVAV

>LmigIR4

EEDKWGAIWENGSGNGLLGAVAMDDADAGYAAVYHWYPEYHFVEYTRPYVRAGLTCMAPRPRPQPGWQRSVDMLDNRDFVLVCYIITRCLLHVVPMNRVIGWIESSLDACMVLDTRLLTQRRCLLSSPDSVVLWGHRLQYLHLHVGLYHIKYYSHLVTIWSSLGVTLSALFGGRLRNLLCLRRYEPPIDSVVDLRDSGLEWAETHIAFLNSLRGRTEQVYLDLVKQYRVLSVDVLKSRTTTRDLAFIIERLPGGYFTIGDYIDEEAASRYLRPMREDIYWEYVVLAVQKGWPYAQQLDELIDKLFEAGIMHAWEGQV

>LmigIR6

MRSLLIVTMLVNSMCDGRLVLQNNDVSSIDSLIRELGSRILSKEFGSYKCVAALSDVPEAVAYFTQWFPTTVLISLVSGDVSAEDLLVSALDADCQGFLVRCRDAAAGVDAILKASKFAMRRVNRRLLVLPTAQSPHINITAIFDLRNIDLVPEIVVARPSGDNSFDLVTLKLTGEDTWRDELLVAQWYRGRGLQPPSADLYAHRLADLGGRRIVISTVDYPPYVIFNSGDNVFDGIEARIIIEFIKKINVTWSVVEDTTYKWGTVWCNGSGNGMLGAVAADEVDVAFSALYRWYPEFMEYSRQYVRSSVVCLAPRPLMLAGWQVPVLPFSPAMWAAVGFSVVVATAALYAVKKLSDWVLGSDDDPSGGRYSTVEDCFFRSVGLLVLQTPDVERRHTRVVGPTRHVLSWLLITYLLVTSSYGGGLSSVLTIPRYEPPIDTVTDLHDSGLEWAQTDIAYLNSLRGLTDPVYLDLIDRYRVLTPDMLRSRVTTRDLAFAVEKLPGGYFAIGDYIDEEAVSNWLRPMREDIYWGTVVLATRKCWPHLERLNDIIDRLFEAGIIRAWEGTVAREWLVARVQLAAQVGMRSAQAPDGPIKLRLTHVQGGFALLILGLCLSLLLFFLEIFVYWKSNGSTRLEFSSTIKVTLEHYRS

>LmigIR7

MLAWVAVTSFFLWPDVVARHQPAVVTNSSLEDCLAYVFSSQPFTWAPLLLVTTTSPDDREPDVSAITNAVVHTVQLPVVLVKLPTTSVYADLVAGYIIVLPDSDLDQDTFQSILVNISYVANWSPRRPVLVIFRAGYGRHEDSVRMILDCLDKRNAIEATVFVVNVERTSWEVFGYYTVRNISAYTWFPYRQGHCRDDGVETELIGNIATTGRHNFTVFPEKLPRSLHGCSVVAATYHLPPFVSKVESEGRQTSFSGYDVILFRLLARAANFRPVVSAPHFSGPQWGERLENGSWSGVVGRMLRRQADAAFGGLHDNAXXXXXXXXXXXXXXXXXLPDALTWFAPRARRACALCEFGLIVPARDAGLLLLGALLMSVPFKLFSGQDEISALRRLDAAFLFALRMLVTTPARAPRTLRPRIVFIFWLQLTYILITGYQSTVTSKMVQPGTEEQILTLEQLLRSDLYCGTLLPFQRWMDTVMKSKAPKISYLCPNIADCMFRMAYSKDLAVMTTKVHTEFMGKVKYVDQAGRSLFLPFTENLFTYYVAVHVQQGSPLLPLFDDVILRVQAGALMTPAMREYERLFRYGTVDHSDLPEEVDSQGFIALSHLSGAFTAWAVALATAFCVFVAELAYWRSGFRLFARQLRCSRLPEKEKDKNAFGSDNFITEKKGRKLRL

>LmigIR8

LCGKATLADVTRPAQSIIQTVAPVTTEKAAAPLQEPHVCLASPQIPIRCGCTYGTAICIVEARKPPHLGNVCGSWEWDTAVKFHKVNKRLNIFEQTNLHWTVDKRRKTRNERCSLSLLSRKNSGLTSLETFANTRRISAAEVWEMTSRIFFHETINSDRIVDWPPRYADLNPCDLYLWGMLKELACREMNFSSGTVLTYDPFLVKDSIWSDSYTPKLQEVKETADIYYKKYMNLNGNNVRASMFTVNPSAIEDNTTVSKFRGCDAQMIETLAKYMNATLVMLPNDGSGFGKWNGTVNTGTDGDVMFDRADIAPNTRYVIVERLKVHGYTYPHDKEDLCILVNKSPRIPQYLNIILPFALIAWLTILLSLPFSALFWSLIRRFGARPSNSEPFARVYIGSFLKIFSAFLSVAVSALPTVGRERILFVMWTFFSLIITNTYQGSLTSYLTIPKYMPDIDTMEELSKSGLKILIHPELLPVFKLDTGNPVMDALNRNLVSDIDMEGYPEKIQSNTDTCALVNAYVGQFLIRSRHYVINGFPLLHLTNECPMPAVVAFATPKFSPLQPRFDVLIRRIVEAGLYKKWQKNMLDESIASGDLLLISNREGKADPERITLSHLQMPFYLLFLGYFLTSVLFLTEYLRLKMNTRSTNEEEAKLERREGCQTLENDSASYLLDV

>LmigIR8a

MWPLWMSVVAAHLQLASSQATSEPLLIRFLLVTEVNASWVGGELRANLSGLEARYVGLRLQLDLSAVEVDREHEVEEFQQKVCGALASGVSALLDATWTGWRRLRDEAQHRGLPYLRLDATLANFVDAVDKYLHAREASDAALIFHTEEELDQALYHLIGNSVLRVIALNGLEKDTVSRLKDMRPVASYFVIFADTAHMAELYSKAAAGGLVRKAERWALAFTDWDWRSFRTDQLNLSTALLQMKPASCCALQGEPDTCKCALRKVAPAFLRAALSAAVDTLAELHSKGMDVRATPRQCSSTGDGGADDEDGDGAGEEEESAEPPANATGNYDAFLRAIATRGQSNSTLFFRAATAQLTFNTPLQLRMVNRSEDVSLGDWSPEKGLQLERQLKPAKRFFRVGTAEGVPWSFPVRDEKTGEPLVGPDGEPIWDGYCIDLLKKLAEPTHMNFDYELVPAKDNDFGSRSPSGSWTGLVGDLAMGETDMIIAPLTMTSEREEMIDFVAPYFDQSGISIVIRKPVRETSLFKFMTVLRLEVWLSIVGALTVTGIMIWLLDKYSPYSAQNNKEMYPYPCREFTLKESFWFALTSFTPQGGGEAPKALSGRTLVAAYWLFVVLMLATFTANLAAFLTVERMKSPVQSLEQLARQSRINYTVVLNSDTHEYFRNMKNAEDVLYNVWKDITLNSSSDQSRYRVWDYPIKEQYGHILQAIDQAGPVPNASVGFQKVIDQEEGKFAFIHDAAQIRYEVSKNCNLTEVGEMFAEQPYAIAVQQGSHLQEEISRQILDLQKDRYFESLTAKFWNNSAKGTCPNSDDSEGITLESLGGVFIATLFGLALAMITLAGEIFYYKRKKLTAVNVTSSSAKVPKKQVTIGKEFRPVMDKTAPRVSYISVFPRNQLY

>LmigIR9

MQLHPSPCENWTEPHVWIHYPYRKENPMLRVPASQFVVVETLATTHRYWNLEGAHISAVMYPAYPTAMPAGQREINTKTCDGDTTGYIELHANSVRIFKYVRGSDHFLRLTLEEKLNARVVARYPKDENFVEGEELPNGQLTGALAEMASGQAQLGLNLRFVRPLPTPEILYLQPVCSFARVGIAVARAPLAPLHVAIMRSFDLKVWFSVAAFYILSAMIWSLLTSSINYKSVLQEIQKFLLIGCGSFAQSPSQKFFASACAIFSLIIVTIFQEKEKERKGEEEIRKELGKTNGSLVNVLTTPVYDRDINTANELLQSGLPIATNRTSLIHMFQLAAEGSPVMTELCKHMKPTDKHNLTELLFDRKQALIIADAGLAQLKNHHPSQEKMLHMIKDTFIVLQRAYAARTDCPVKEQVEKIIWCLTEAGIPRYWNTQASRSEFSNCPLPEDTPKPFSLKQFKIAFYTLCIGLLFSSFVFLLEIISYKRCQKHILK

>LmigIR10

LDVRLLASTHGLCRSAVADVATSILRSHHPRPAVVSTGPTYLPPATTFLTPLQNEQRLLVTDSASPVVEFFQFGRAHSRLWSATNDFIILLLKAYDHDGGDCNADCANLLRHLWSAFGAARVLVRHVEVSDGCHVHRLGPTVALFDPFDTSAGTIVSTTWTNVELIKTLLDNRTRDLRGRPFRVCMFARNYTALPVDANGVLVYRSSLETVKYAGVDGWALQTLAQYMNFTTVFRQPYDRERYGELLPNGSFTGALGELMRGRVEIIFNAIFMKSYGSHEIYFTKGVSKDDIRLVVPKGGLLSKRHLLTMLMRPEVQLSILVTYLLMTLLWYASSRLHMFFKPEDAERPDILMIAFQNFHPPTSSALEKLPRHAAERFLVVSYLLFHMLVFTIVQGKMLEVINNPRYEDDINTLQELVASHLPIKTGSATLIDIFGPTGSAPQPESLLQSLSRKTNENHQKGGRKPDGNLRFNVMIFRAYVSASFVALLLSTGHRHSSVAPMAFTFTVLGNECIKELDDVIDNMVLLEKGSAVEEVARSRDAACVVRDSDLRHGKYSAYRRADGSWLLHAVQEAALSFELGYATRRDSPFLAAIDRFLWRAVEAGLSRCWERRGYLLVTLRARADPASIDTAAAAAKTYKQFTVSDLQMPFYAWIVGVASSVICFAAELLYSWWLSGHKN

>LmigIR11

MSACEVTIGADRIGVLTFAQPVRNAGCRFYFQTQFQEVMFKPYVQPFEDRVWIALLGYVVATALLKWLFHYVLRERADPITALFAVIAALLNQASGDPRRCAVRVLYFSLCALFLILRIGYGARLTATSTLRQAAPPYRRMEDILASDWGVNVISNSFALESMQMSPPNSTAWKLWQTKIDNNPYSTVETTEDGLWNVLDKKKLAFFGFEDACRDVLHRRFSPDQSCRISELDGVFFKAPLSFALPRDSEFLLTINYWILRMFETGIIDRLSRKWLPKPPVCDETKYEAETFADVLPMVILFASGFCLAIIVLIGEICVYSFKNRNMKVVSKNRKVRKTQKNYLYPML

>LmigIR12

LQPEVSSHSTDPSGKKFDGHRFKKDTNVNRCRGGVAKRIKALCSDERGHSPLGDYMAFETAWQIKIVCRNRDSNLGPLPFAGKRTTDCAIPTRLTTPPHNFTSASTSPPSSRSASLVTSSALPKNWYEISGKCLSCVHATVSQTYQKLDKVLRHVIATARYEVSTYSGLVPGNQKEDKSWTGVIGLLQQGSAEIITDLLTVTASRVNAVDFSSPATTDKFGLFIKDEVYTDVNRWSFVSPFETTLWMAVIGTILLYMLCVTVMNLASYDKNPQCSTKEILMGIFAAFCLRGYSLLLPRWSLRLAYLSAFITAVVIHAAYCARVVSHLANSNRSLPFTDLEEAYAAGYEIQVVPGTSAAETFKYASDGIIRTINEEMIEPRYFYLPVTINEGLLHMCNWKKTCFVSERNSVRCSQKQPCGIIEVSVTMPSVYLAFALRKKSPFRRIISYQMEKLRTGGILKRLKSTSCNRAQADQGSDFKRVQLKNAAPLLAIIYFAVIISFLVVILERALFRYTQKTNSKSVPKFRKYRKRIRQTPMYLP

>LmigIR13

MDRMCDCCVRAQEELAAVREQLSVLLAAVSRLKALGGSGASHGTPQVVLASPTIPAVGTSSRVPDAVESPSPQGVWRVQRRSRRTGRKVNVEAGRAATPALPNKEFCTKSIWLVFLRGDEILEKAFEDMYIPLNCQLLVVHQYGQQHFITEIFHLKEKLHLRKVLYGIWSAEHDLQKTADGFYSRRCDLLGETVKVSTFKSYNSEYGLSQLRLLSETLNFTYKFVKVKEMFPGRLTASGYSGMLGAITRREVDMTIDLLTHTTARSHVVDFLFPTQKDTHSMFIKLSLDEGIPWTSYLSPFCGRLWGTVVLIITLDAVALAVLLRSSGHKVWTSDFLHLLLDVLGMYSLQGLKGSTGTDRPPCHPPPEVVIGYEIAKDLEEQLNGMENVLKGTHEMNINKSKTRVMECSLTKSGDAQEIRLGVSVGSRTSAAQLVSVSAYVTAIVLQAAYCANVVSVVASRRYSPPFSDLAGLLADRTYRVAVMRESITNDVFEFAGDKEMKLVYQHFIEPHNNDMPVLEEDIDVILCHRYRYCFTGESKLVDSHTVSCEIFEVRLKTPPSHLGFALWKGHPYKNIFNYRYTIRIARLADVTRPAQLIVQTVAPVTTEKAAAPLQEPHICLSSPQIPLCCGFTYGTPICILEARKPPHLGNVFDYDPALVPFLTDRLTRDENSTNGIEDDTSNGEGD

>LmigIR14

MRRGAININGDYTGKKVELAETSSKMGLDVLAFSDVRVRGEKEDEVGEYNVYLSGVKAGRAQWGVGFYIRKAIEPSIIAIRPFFTWQELLKTRQSEFMWLLLLRRDQSPSAVLQDIDIRFDTLFFVAVPTTESDVHIVEAYRVAKGYPLVMQTFCTWNTTYHPSCRTESTIARRGDLMGYKMKSGIVEHSPMVQEGLSNLMRPPRHELASRYQFKEMSGWGIRSRDGSWSGLIGRLVSGNLDVGVAYKLYIQTPSEQEFIWGHFLWPFDKWLWLCSFALVVTTWLVLRVQERFTRDDESEISARGTYNDFLQVLGIFLMQGVYGGRTAPRACVRASLCAASLLALLLYTAYCGALTASLASRRPRLPFRDMAGLLRDASYSLVTIADSSILNVLSAERQAEDEALRGGSDECGPQMEHRQPPVEREILNPVHSESSLRSHLMKDSNNPVDRKIYEKHIEGKHMPHTSEELFGWLCSSQKHAMLVSKTRYMHGTDFVQCPVTSVHETSGSQEGFVLTKKSPYREAINYHHDCHRDGEASGSDPPLSSKPMDPINPGNYATNQQLCLYPVCEGSSLHQFRQLPIGTEDVLRTQATSIIDADVSHDLQSTAPHNLDSRQQDRIHISDEAPRQTH

>LmigIR15

MFIGFEQYASCKIPIKTVAAVVSSLQDEIHFGCVYLTYSSREAELLPQSRFHVEFRRGLRNSIQLGFLPDTHETQLRHNCSSSPLLVVPHTDLLLNHSSVIKDLLRKRQSDFMWLLLLNDGNMDAALADIYVEFDSNFFVIFESTDGTVVIVEAYRVAKDFPLVTKKFCTWQINTNLTCTKEGIRSRRNDLMGYELKTGVVEVWPDMYDLGNGSCSGYLCDIWNILSRKLNFTKLAKHNGHTIIRKPLNVSNTVAIEEEEKEARQRKINVWIHSTWITKPVQKEFRTLFPHLMEPYGPAAVAELSAARQSQSARPLQSWVRMRPRAWLFVPSLLLVSSKQTRWVKDGVSQQRVGFESGELMLLFLRMQNSQCGDHLLRLLQENLFGVRYRLQRSSLFGSRLSDGSWSGAVGLLAGGEAEVALSVMMITPSRLSAVDFSLPFFSTREYLYIREPEEEDLAWDGFLRPFDGPLCVCTVAVVLVIWGVLKVHHRCAPSAGTQLDEGRLYSDFLHVIGIFCMQGCASASRDSARLSLLVASMCALVVYTAYCGALTASLATHQPRLPFTDLKGLLQDGSYQLLLLDGSGEMTILAIWPSCAFVWSPSHIGVLVNEHVNRLAKQATTTPALEIGFTESNLKLALHLKESEDPVTKEIYNNMVVKDDLPEDSNEAFDRLCSSEKQVLLCQLMDFRLNENSLKCNVTTTQEYITSQLAIAFRKRSPYREIINYQLTNYEWGAQPSVVRTSVLAVCVSASEYEAPVWDASAHTKQVDVSVKETLRIVTGCLRPPPTGRRRHQGLKEACGESALPYRTVARWVKAFNEDRQTVAATHRAGLPSAPEEDCILLPRWRTVIDARRFVNSPMKPD

>LmigIR17

MFFYLLIRDVATGGIREVYRFYVGHAVLYFNPYPLSKNSTQMDGSQLTVNEDGVINDVFPAGELWKFIAKRLNISTQLIIKKDPSCGSYDHGQWSGMVGDVHEGKAHVGLSLFTMSASRASVVSFTTPIFLGRYFIFIRSDEITHVGPNPLELLVPLKHDLWLAVIFTLFAYTTVMAGIFYIQRKTLCDAVFTIIGAFVQKSGKNMAADRIIRFGPLDGITSYHQRITSEQCLRFAAYVTGSVLLSAYSAGIISILTITEPKLPFQNFAEIEEDGSYMLGVTDNSFEYDFFKLSQQKLLQKLFNHQMDIHNLPRSKPEGLSRVCTGTKYAFLSTDLSVQSSWSQQNCNVIAVPIDMFRCTLALILRKNSPYIGGMNFHIQILRGAGLLNHMLILYNIRDTSFHQLYAERNIMELQNIFPIICVLVLGIIFSFMCLIME

>LmigIR18

FADSVNSVWLEKNLAKQRKALTFAAHNWTSGVRRCPRNKVLTIADLSYVATPGVFTKGVRRNVTWLVAANGGEWRRQLERCYVPLDALLLVAEALSAGQLVEITEVFSPRRGRPPTFAFFGRWTPATGLYTKRDDVTALYRRRTSLDGAQLAVITYHDPPSVTVTQNETHTTVGGYFGEVWNTLAKHLRFRTQWLVQEWPNPGYNRADGTWDGAAGALLKGEADVALVATTMRPYQGTPIAFSYPLYYSSRSDDIEGRPGVNSSYSASGAGRDRLFVRRMDSPRSSWGDFVAPFERRLWVAVALSVPALAAALAALYRLGRHFGTADATGPYDYSFYDSLLYVFGAFCQQAIITFAAYSAALISSLTVVSDDLPFSDIEGLLRDGTYTVGVLNRTETFHGLMSPPGNTNGGLFYLRNIFTREDALIIYYTVELHVLGILPYPIKGSNIPRSNLLLPLFRNHTFVYPLHKHPSVLVVPTRFIALEVKSSKVFKVLKTRENQVGRHAKTATWSNKEMVSLLMPKHVLIFLALLVNIEVSLYEGC

>LmigIR19

MCINKPPDGELSYKIHIYDIGKESDYKYYARDTVVRTDGNFQMDMNGNRELFDKSILQLLQDILEFSLLCVEYHEIKIYLIKLGHIQYGRVRRLKVSNHNGKKYSRDLSILRQLATDKIDIGSTTILLTYDRIGMADYTIGIDTFNSSYVFVKNPAAENSPVTGINASRKQFSLNMLEQPSWDLSEVMLVTIGALSQQGADRHPNTMAARILFLVLFLLAVLMYTAYSASVISIMSSTKPVSNSLQGILESSSKMSVALHDIHYYHTQFQIERNPLSQNLHKLELVPEFLSLEEGLEGALEGNIAFSCGRVDAHTYLQNSQHTDESLCGLGEIPILRGMGYQRSFALKHNSPLRRAFDRGLLRLMEHGLIKREWHRYFGKRPASELHCEETSSSTGFVRITLDDVWPAVKMFGVGVIIAILLLPTELLVRQWLVLLEGNNLCENVLRSKMLTPIIK

>LmigIR20

MPSLCKASVIMTVLTLCLTSTIFCESQKSYGNSINFILDFIRHTTTASSIKGVHAFVCWDAGDLQLLKALSRNGMLASVYSGWQHWEQLPTFHMDGNSLLFILDLKCEKSFAFLKKTGRNVEFFKPPHMWLILHDAYHPDTQLFTDHSAETMLPAAKNNGKNVLKQQHNVSSYNNSIILDAMELGDEDFSSADVYDLSYGTSPKRRAGYTNSNSTHVTHVPSGVGMKNSEFTSNHINDNSAVVEAAETGFYCEVFRGLDILVDSQVTVGRRDADSKYTLLEAYRRRRQGELVVSELGYWEATAGIVWRNVREVSVRRLDLKRTKLVASIVVTNPETLDHLDDIHNRHIDTVTKLNYVLLLHVADVLNASLELLVTDEWGYESNGSWSGLVGSLQRAEADVGGTALFVTADRMRLIDYIALTTPSVAAFVFRQPPLSLVSNLFTLPYTVRQS

>LmigIR21

LSLVSNLFTLPFSRAVWACAAALVVTCTVLLLAATRWERTRGDPQLADYLSQFYAVAGQLRDKWGEVAMLAVGAVCQQGSPAESRGVPGRIVTLSLLVTVMFLYTSYSASIVVLLQSTTSSIRTLADLLYSPLGLGVHDIVYNRHFFPAADDPVRRALYRQKVAPPGAEPRFMTLEEGVRRMRTEPFAFHSELSPAWQLVQETFREDEKCGLQAIPFLQLMHPYIAVQRGSAYKEMFKIAYRRLWESGLQHRQLSRLYTMRKPRCAAGRGSSFVSVGIADCYPALLVPVYGVAVAIIVVLAEILFHRRVEVLRW

>LmigIR22

MDEGHQADCVFLDFKKAFDTVPHCRLLTKIRAYGIGSQLCDWLEDVLSNRTQPAFMFREPSLAAVGNVYTRPFSRGVWLSYSLAAMLLALLVVGSQRLLASARGLLDDMTDPAVAPPWADVPLVGFSIICEEGASLVWGYELLSAFSAVHSFFNKHTVYYNPLSIFVFEIFDFEIRESFSGHPVFSTRELDQFLYQRTSKTLQEINNFFSSMILSVHSRRSPIEFPEHFRDNRALVEPTSNEPSCSPLNCFYISSKRDRKYHKNNWMYFKVRVNPYCEGYRDTFNINARPTPWRWSGIRPPPQNVSSRILLMFLLVLAVFHVTAYSACIVSLLQLPSGSINDLHSLFGSKLRVVMQNLPYNFNYGNTHLVTPLCLAVKETKDPLTRSFYQERVYPQPYGKVFKPLEDCVALMRGGRFACHADEAAYKVIGDTFLEAEKCSLKSVPMFPLRAIILGVRKHSQYKETLSVIQAWLRETGLLKREWTRWVAQKPRCLNRDSGYAEVGLTEVSPALLMLCYGVAGSLVALLLELLLHRAIAARPGRHQRQPKTPARGNALVNRAFLR

>LmigIR24

MKPPAALLVLLLVCQESSGQDSYFTDVISLTRDYFVAKRVSLVTVYTCWNSWLEKDLLRSLWDRGLRASRLPLAEAAETSCGGSHGAALASLEAAGPYRSGVLVDVACPGGQQLLTKASARRMFGIQRHWLLVDSSGNSTAGDEPEFPALPARWRDVLSSLWMMPDSEVVWMGAADDGAIQLLDVYRLTSFTPVLNISLAGWAVRRNSGVSLYLLPRPDTSKRRNSLHGAKLKAGAAILFPKYFTGMYDLRLPHLDTWTKITYPLIEYLGQNFNFTMEVFYTDSYGWQTNGTFDGVIGMMQREEIQIAASSLFMRRDRMPYVDFAAEAFYLKTAVVFRQPTLASVANIFTLPFSAAVWACCLLLCVLTLLLFGVQLRLAAKRGIEGELTHVTYAELFTFVLGSICQQGLPQTPTSLSGRVTVFVLALTSLFLFTSYSANIVALLQSPSHSIRTVSDLASSPLTLGVQDIAYNKVYLGETTDRELRQFVRRKMQPLGNRVFYNGAEGMERVRKGMFGFQVDTSTAYKIISETYTEREKCGLMEVNLFPLPPLCVATTKHAGYREMFSQRVGWQREVGILTRQRRLWLPQRPVCENMVSGFVSVGIMDFYPALLVLQYGVAGAVVVLALELLYFHRSRLWQRMICISQLSGAKPTSMYAVATDQISQKRSQLQRTGSRLK

>LmigIR25

LHNGTYGGMIELMQRNLSQVSGSSLIMKKNRWDVVDYTGPAWRFKSYLAFRHPTSQGLRSSLFAPFCSSVWVASGAVWLLIMSSLRLITWIGARQAGGIVQAESSWGAVVVFAACAIGLQGTTERSQWLSWRVVLLFTFLLSLMLNTYYGAAVVGSLLVPPPKTIRTLRHLIDSPIQVGIEDIGYNRDYLEKSTDPLVRELFVRKVFPPSAKRPHYYPIDVGVERMRTSLFAFQAEAV

>LmigIR25a

MEAGVTWVPDLQAETPEGVPAAAQGAPSAEKVHVNDDKNSIPNKALKQVVADLSKQGMIFDGVFKATANGSDVEALIDSMCLEYNTSIDENKKIHVVLDTTLQDVSSEAVKYFTRALELPTVSASCGQEGDLRRRLSWWENMYKKNLSTICLPRCVCVCICVGVGVFAYLPFSPQVIKTLLFSLYAVMDHKYKSLLQNVPTRHIINEIKFQNIANQLSTFKQREVFNYFILGRMDTVNKVLEAAADMEFYGRQFGWYAITQDEGNPSCQKCGKGASVLHVKPNDAEGTVVGAENPKLAYQFYYELFRNTFLAIGQMIKEESWPDMQYIPCEEYEENKNVPPTRKLNLLEALQQISMLNPGAYGQLMLSSNGHSHMQFNMTAFNVSLSDNSATEVGTWAADLDSPFITKVKPSVPVTQYTVVVALQQPFVIKYQDENGNTKFKGYCIDLINAIRNITNFEIEIYEVADGKFGNMDEEGRWNGMIKDLIDKKAHIALGALSVMAERENVVDFTVPYYDLVGITILMKKPKTPTSLFKFLTVLENDVWLCILAAYFFTSFLMWVFDRWSPYSYQNNREKYKDDEEKREFDLKECLWFCMTSLTPQGGGEAPKNLSGRLVAATWWLFGFIIIASYTANLAAFLTVSRLDTPVESLDDLSKQYKIQYAPIANSSAHGGGEAPKNLSGRLVAATWWLFGFIIIASYTANLAAFLTVSRLDTPVESLDDLSKQYKIQYAPIANSSAHVYFQRMAAIENRFYEIWKDMSLNDSLSEVERAKLAVWDYPVSDKYTKILQAMTEAGFPANIEEALERVRASKSSSEGFAFIGDATDIRYQVLTNCDLQMVGEEFSRKPYAIAVQQGSPLKDQFNNAILQLLNKRKLEKLKEQWWNQNPEKRNDCEKQDDQSDGISIQNIGGVFIVIFVGIGLACITLAFEYWWYKLRPQHNAVVEAAPPRTKSDSLQALNMMRSSFDKRYGRRQGVALAGVTNPW

>LmigIR26

MLGGKAGSVDAGATNLRQNVSRVPTCKRSCCPAADDDVAEALMFEIITITITQHAEYLITVTSTQTDNLHERLISENQKHLDPLARFNYALFLHLKEFYNFTFSVQATRSWGYKTKAGRFDGMMGVIQRNEADIGASSALIKKERLEIVDYAGHTWKFWPRFLFLHPSGQRLHTALLTPLSTKVWFCAILAGLMITLILNTSSYVHADNFCNLDGSWSSTLITTIGTFTLQGAGSSWSQISWRITLLTALLLASLLNIHYGAAVVGSLLIPAPHTIRTLQDLMESPLRVAFENVSYNREYVARTTDKLGRELIHRKKPEFVQLSEGVIKIRKGFFAFHTEGGSVFRLAAMTFTESQKCALSDVSLFTPAVMSMPVKKKSPLRELFARGWSTRRPNCDFLRQSLPMDGPMVEVKKIDSTNPWLKL

>LmigIR27

GTDLLCADHCRNHGFRRSLGGLILRAGTVVHDLPLVNLGHRLTNVEEKHVDTMARFGWELTTILSKKLNFTPVLYATDSFGYKQDTGNDTLDGLVGMLETGLVDVGSAGLAMFKDRLNRIDFVGPSRLWVSKIMFRHPTVAAEQGALFRPFTPGLWLGVMLVFAALALVSHAICRAHGWSAQRAWGDTFLLVASAVGQQGSSEGVQWPSWRLLLMVSLVCATLLDVHYSAAIVSSLLLPPPRTINTREDLLRSPLHFGIENISYAHDLIEMSEDPVIRSLYQKKVAPPGAVRPNYFTLEEGMTKVATEMFAFHSQEFQMYQLVEKLFTEQDKCVLVTIPLFPPQMTYVTVAKNSPLRETFTVGMRAMWEQGHLRHLRMRWHSKKPACMAERDFVNVDLATISPAFMLLLSATLISLLLLLRESAKARQTQDNTPVPLSH

>LmigIR28

LEALYRVHPSRPLTVRQAGSWTPHQGIVLHRSAVHNGWRLLDGLILGAAGVVVDTPTDRLGERLRVVQDRHLDTNIRFGWDLCDKLRFMFNFSVVLYKTESYGYLTPNGTIDGQAGMLCDGTIDLALSQLMLSHHRLDFIDFTTAPSRMWTLKTVFRHPASRAVYGTIFRPFSAALWLSSGLVFLLVLVAARLGCWAAAHNPADDSWSAAFLLVSSAISQQGTTLDTGRPSWRLVVFLSFSCALLLDTYYTAAIVTSLLLPPPRTINSKADLVHSQLAVGMENISYTHEYFEKSSDPVDHALMKYKVWPAGTARPNYHSLEAGVRKVAAEAFAFTGEDVSLYPLLDRYVTEADKCALVALDFMKSRSTYMPVRKNSPYRELLTIGLRRLLERGHLAR

>LmigIR29

LLKDLQGLALGAALPIVDTPLDNLGERLKNVNDRQLDTMARFGWGLSTTLAELLNFSIILYRVRNFGSLINDHEMDGAVALIHNGTVEFGAAGFIMTTRRMDFMDYTGPGRLWAPEIMFRHPKSASVLTTIFKPYTAELWVSSGALFVLILVVSRLFCWVEHKVTATVDEIDNSWNSTFLLVSSAIGQQGVSRSSEWLSWRMLLFVSFLCTNLLDTHYAAGIVSSLLMPPPRTINNKKDLADSTLGFGLENVSYTYQFFVKSDDPVDRALCSRKLYQAGGRANFFPAEVGVLKMAREPFAFHAEDVRVGPLIDRFFSDDDKCALVFIPLLTPVATYTAVRRNSPVKELFNFGLRMMWERGHVNYLRKAWYFTRVRCLSETEYASVDLVPMSPAFMLLGCAFLLSGFLL

>LmigIR76b

MQVSPVLRTVLTTVCSNYFLNGTKMEVPEGEPDPGCVLRIPKLMEGKTIRIGTLENPPLTMINKTDGTLIGHGVIFEIVDILKHKLGFNYEVVTPRANILGDENHGIIGLVHSRQVDLAVGYLPQFSQQARLVRHSESLAEAPWVFLMKRPLVSASGTGLLAPFDATVWYLVLASVVLMGPAIYFIILVRVRLCAGSERLTRIFPLSSCVWFVYGALMKQGSTLMPVTDSSRMLFATWWIFITLLTSFYTANLTAFLTLSRFTLQITSLKDIATKKAHWAAQKGSAMEYLVYNNDEYSFLNQSLQAGFGQFVDISDTDMLLRIKKDDLVYLREKQHVEHTMFRDYLEKTRNPKVEEKDRCTFVMTKQPFLHLPISFYYPLNSNLAHIFDPLLKALVETGIVRHLLRKDLPQIEICPLDLGSKERQLRNSDLYMTYMIVVTGFCAATVAFFGEILTRQVKRCIAEAELQTGPSTSYPDDWKTVKAQANRMPYTMYLNGNIINVKQPAYSITKDFQSSNRKSLSRQRNTNYVFQYTS

>OasiIR1

LGPPYLKEKTPTTPPRVGNDRYEGYSMDLIAQIADLLNFTFEFKLAPDGQYGSRDEKTGSWNGLVGELIAGRADLAICDLTITQERQSAVDFTMPFMTLGISILYKQPEKADPNLFSFLDPFTIDVWIYMATAYLGVSIIFFILARMAPGEWDKSHPCDPDPTELENTFNMINIFWFSTGSLMGQGCDLLPKAVSTRLIAGMWWFFTLIMIASYTANLAAFLTNSKLEAPIEGVQDLAKQTKIKYGTYGRGSTAAFFRNSNDSLYQRMWLVMKQARPDVFTADNQEGVERVKKEKGNYAFFMESTSIEYQTALNCDLRKVGGLLDSKGYGIALQRDSPFRTAVSGAVLTLQERGNLSALKTRWWKAPEGRECAEEDAAATDNNEMG

>OasiIR2

MFAAYIGVSLMLFVMGRISPYEWTNPYPCIEEPETLENQFTLSNSLWFTIGSLMQQGTEIAPIAVSTRMVAGIWWFFTLIMVSTYTANLAAFLTVESMYQPIKNVKDLADQNTIKYGAKRGG

>OasiIR3

MVGVVFAIFVTFFELLWDVGHKSLKEKIPFKTLLMEELRFVAKLHGTTKPVRKYNQDAEVEDTNNFIPLSPYTNSYGFVDSKEPLT

>OasiIR8a

MWPLWMSVVAAQLQLATSQAASDPLLIRFLLVTEVNASWVGGELRANLSGLEARYAGLRLQLDVSAVEVDREHEVQEFQQKVCGALAAGVSALLDATWTGWRRLRGEAQRRGLPYLRLDATLANFVDAVDKYLHAREASDAALIFHTEEELDQALYHLIGNSVLRVIALNGLEKDTVTRLKDMRPVASYFVIFADTAHMAELYSKAAAGGLVRKAERWALVFTDWDWRSFRTDQLNLSTALLQMRPASCCALQAEPDSCKCALRKVAPAFLRAALSAAVDALAELHSKGLDVRAAPKQCSEGSGDDEDGDGGEEEDGVEPPANATGNYDAFLRAIAPRGQSNSTLFFRAAAAQLTFNTPLQLRMVNRSEDVSLGDWSPEKGLQLDKQLKPAKRFFRVGTAEGVPWSFPVRDEKTGDPLVGPEGEPIWDGYCIDLLKKLAEPTHMNFDYELVPAKDNDFGSRSPSGSWTGLVGDLAMGETDMIIAPLTMTSEREEVIDFVAPYFDQSGISIVIRKPVRETSLFKFMTVLRLEVWLSIVGALTVTGIMIWLLDKYSPYSAQNNKDMYPYPCREFTLKESFWFALTSFTPQGGGEAPKALSGRTLVAAYWLFVVLMLATFTANLAAFLTVERMKSPVQSLEQLARQSRINYTVVMNSDTHEYFRNMKNAEDVLYDVWKDITLNSSSDQSRYRVWDYPIKEQYGHILQAIDQAGPVPNASVGFQKVIDQEEGKFAFIHDAAQIRYEVSKNCNLTEVGEMFAEQPYAIAVQQGSHLQEEISRQILDLQKDRYFESLTAKFWNNSAKGTCPNSDDSEGITLESLGGVFIATLFGLALAMITLAGEIFYYKRKKLTAVNVTSSSAKVPKKQVTIG

>OasiIR25a

MTLLLLMVTLLSNLLSALGQTRISYLHVNDDRNLIPNKALKQVVSDLSKQGMIFDGVFKATANGSDVEALIDSMCLGYNTSIEENKKIHVVLDTTLQDVSSEAVKYFTRALELPTVSASCGQEGDLRYWRNIDKKQEKYLIQVMPPIDTIPEFIRSFCSEQNLTNAGILFDDTFIMDHKYKSLLQNVPTRHIINEIKFQNIGKQLSTFKQREVFNYFILGRMDTVNKVLEAAADMEFYGRQFGWYAITQDEGNPSCQKCGKGASVLHVKPNDAEGTVVGAENPKLAYQFYYELFRNTFLAIGQMIEEGSWPDMQYIPCEDYEENKNVPPMRKLNLLDSLQQISMQNPGAYGQLMLSSNGHSHMQFNMTAFNVSLSDSSATEVGTWAADLDSPFITKVKPSVPVTQYTVVVALQQPFVIKYQDENGNTKFKGYCIDLINAIRNITNFEIEIYEVADGKFGNMDEEGRWNGMIKDLIDKKAHIALGALSVMAERENVVDFTVPYYDLVGITILMKKPKTPTSLFKFLTVLENDVWLCILAAYFFTSFLMWVFDRWSPYSYQNNREKYKDDEEKREFDLKECLWFCMTSLTPQGGGEAPKNLSGRLVAATWWLFGFIIIASYTANLAAFLTVSRLDTPVESLDDLSKQYKIQYAPTANSSAHVYFQRMAAIENRFYEIWKDMSLNDSLSEVERAKLAVWDYPVSDKYTKILQAMTEAGFPANMEEALERVRASKSSSEGFAFIGDATDIRYQVLTNCDLQMVGEEFSRKPYAIAVQQGSPLKDQFNNAILQLLNKRKLEKLKEQWWNQNPEKRNDCEKQDDQSDGISIQNIGGVFIVIFVGIGLACITLAFEYWWYKLRPQHNAVVEAAPPRTKSDSLQALNMMRSSFDKRYGRRQGVALAGVTNPW

>OasiIR76b

MKRPLVSASGTGLLAPFDATVWYLVLASVVLMGPAIYLIILVRVRLCAGSERLTRIFPLSSCVWFVYGALMKQGSTLMPVTDSSRMLFATWWIFITLLTSFYTANLTAFLTLSRFTLQITSLKDIATKKAHWAAQKGSAMEYLVYNNDEYSFLNQSLQAGFGQFVDISDADMLLRIKKDDLVYLREKQHVEHTMFRDYLEKTRNPKVEEKDRCTFVMTKQPFLHLPISFYYPLNSSLAHIFDPLLKALVETGIVRHLLRKDLPQIEICPLDLGSKERQLRNSDLYMTYMIVVTGFCAATVAFFGEILTRQVKRCIAEAELQTGPAASYPDDWKNVKAQANRMPYTMYLNGNIINVKQPAYSITKDFQSSNRKSLSRQRNTNYVFQYTS

**File S3.** Amino acid sequences of 106 OBPs of *Ceracris kiangsu* and other insects used in phylogenetic analyses.

>CkiaOBP1

MWARLYNCAALLLLLVAATHGWDVNMKLTGRIMDAAKEVDTKCRSSTGVPREMLHRYADGQTVDDADFKCYLKCIMIEFNSLSDEGVFVLEEELENVPPEIKEEGHRIVHSCKHINHDEACETAYQIHQCYKQSDPDLYSLVVRAFDATIDA

>CkiaOBP2

MRTSAAAAGAALLVLAAVASAMEMTPEFMEIVNKCKTEHEPTDDELKGMMMLKVPESEKGKCFMGCVLQEIGVVKEGKFDKEEAKKHAESKMTDKDELEKHMQLIEKCSQEVGGETDSCGIGPKLMECIKQFAPEFDIALPQPPSE

>CkiaOBP3

MRTYLALLVAAVALFAVAQAGPEDKLKESVEKCKASENLDNLDGLKTGKAPSTKEEKCFIGCFAMDMNVLNSDGHYDAASTKEMINNCEHLKNKPDEKSAALEVADDCGHKVTDCSGHCECGPKAVGCLIKGMMDKGFEESFASLDKVLQKVDG

>CkiaOBP4

MRVEVVAAVLLLAAVTNAEDSLVDIVIREVKGCMDSEHLGSIGGLRSTNDPNSAEQKCFLGCMLKKFKALDAGGHYDAEGLKTTIQHCPRMKAHPDIQKAAMQVADECNGKVTGCDDYCSCAPLASKCLHEGMKNKAFQTIFIALDEALDKMES#>CkiaOBP5

NWDSVFTTGSLEDERDLVAKCFFECVLEKTGAMDEKGNINSDTTKALFLASQEGTGPAIEGHDELIDMCVPGRDEEDICEKGYALVKCVTMEELSRRQAGK

>CkiaOBP6

MKALLVAFVAALGCLALAVAAISESMARAEEAAAKIELSELFEECNETFPIPKATINYFFSHGRLQNENDYGSKCYIHCLTDRSGEIDSDGNFDADMIKVMTRRFPNETHIEGLNEMVDGCVAARGESDFCERAYGLVSCLIKEKLARLGHSH

>CkiaOBP7

MRTILLVSVSAMLLAATEADDADQMKVISEIKACMASENLDSLDPIRTNNEARTSQEKCFIGCMMKNLHVLNSDGQYDSALLKEHMSHCPEMAKDPQKKADTLLVAEDCAAKVTGCNGYCECGVVAGNCLAQGMEAKGHETKYTTGFETSWRRCMLRSGPSGT

>CkiaOBP8

MAVSVIAGALGLLAAALATTVTTDIPTEEILRWVETCNKSHPISQELLRSLATSGGLLADESDTNARCYLECYDRLVGVANSDGMLNVENVVAILMHYYPKMAEIGAESVAEIVSNCSSKSGTGQCMTSYLISKCYTEGLGVKSPNVSMFDSSFS

>CkiaOBP9

MQAPQQLLVLLALCVAAAAAALQAPWCPTTASHGVQEDMGKCAEEIKDAILKEYAKTVASSRTSSAEMSEEDRILVGCMVSCLFRKGSHGRLQTGSKLALAELGAMRLFSDGAGDARYLNATAAAVRRCSASSSSLLPDDGGPRHECELGFFMFECVSDQITEYCQWQPEW

>CkiaOBP10

MSTLFACCAAVWLLIAAALLQSTKGDEIMHNTDIPATMAECNATFSLGWSCWDNLLSDGHVMDESKYQQKCWFYCLLDKTGAMHADGAFDKDLLKMVLQGFPNGSSLEHLDETTYTCVAQSGEVDLCESAYAVVKCIMTEELSSMHHSSW

>CkiaOBP11

MKAHMAPVATATAAMLLLLLLLLAADVRGQDDEMKEMMEQLHQTCVAESGAAEGNIDEASKGNFIEDANLKCYMKCMFVQMTCMTDDGVFDADTAIAMLPDNLKDVASKALNACKGEKGSDACDTAFKMNQCLFKQAPKDYMLV

>CkiaOBP12

MFHFYAITFCCLLWLVFHCSVNCSDINIWRKCNETYPVPDATLISFSNNGTMPDENNVTSSCFTDCYGKKTCMLTSDGGFNWTTLEHMLSNFKMKHSAMDMFGKCKHDPSDDVCLQSYLSLQCVAETILSLINAS

>CkiaOBP13

MNWGLWLTVTMALVLQLSVSEGLKCHTDEDTQNPDEFQEVAAMCMKNTSGSELNSSDSENKSNGNNYHKTNLENNNDNWNNGGMGQTFPGYNSENEGYGFRGSGSCNANGDGYNSNSNNMNQNNMNGMSQSPRNRSRSSGQQSEAADVDLEDIEPCAVHCIFSQMGMLGDDALPDSSAVAKVMLRGVKDTEVKDFVQEAVEDCFDQVESDRKGSKCEFSKSVALCLRQKGRENCEDWGEQEDDQQSNQNKNGNNNGNYSNNSNQYGNKKWN

>LmigOBP1d

MWARLNDCAALLLLLAAAARAWDVNMKLTGRIMDAAKEVDHTCRSSTGVPRDMLHRYAEGQTVDDDDFKCYLKCIMVEFNSLSDDGVFVLEEELENVPPEIKEEGHRVVHSCKHINHDEACETAYQIHQCYKQSDPELYSLVVRAFDATIGD

>LmigOBP4

CDPRHDTATMSALFTCCVAAWLLLAAALLQPTKGDDVWHNTDIPATMAECNATFRLGWRCWDNLLSDGHVIDESKYQQKCWFYCLLDETGSMHADGAFDKDLLKTVLQGFPNGSSLAHLDETTYTCVAQRNEVDLCERAYAVVKCIMTEELSRMHQSS

>LmigOBP5

MCLVVLLFGFVQSLSLLFVPDSCLLLFVVVMFYFPCKISQANGSFVMTMLCLEQTCKMMDQLHQTCVGESGVSEGNIDAARKGNFIDDGNLKCYMKCIFVQMTCMSDDGVFDADTAIAMLPDNLKDVASKALNACKGEKGSDACDTAFKINQCLFKQAPKDYILV

>LmigOBP7

AAETKVMEGIKACMASEHLGSLGQLKANNEARTPEEKCFVGCVMKHLHVLNSEGQYDLALVKERANNCPELAKDPQKKADTLRVAEDCAAKVIGCSGYCECGVAAGECLAQGMEAKGHETIYDFLRKIVDKMDV

>LmigOBP8

AVRLLLLLPPLLLLALSCVTAAPSITSTEMRMDMMVIQHCNETHPVALIDMNKALINKKIEPQNTVFKCFVFCLLNKYEWMDDEGGFLIANMKHNLSDSHLDQLSIDFIVYKCSATGSSDKCERAYRFTECFWGEVTKFPENSDEKYEDPDLFALYQ

>LmigOBP9

TDATATAAMDKASSAAVTACLLIAVAALHTQALSLEQLRQTSKIVRNMCLKKTGVDLALVEGIQEGQFPDNQDLKCYMKCCMGAMQVLRQGRYNVNAAKNQADKMLPPDLKGRFIDMLDACSDRGDGVDDDCEMAYQLTKCSYETDKEIFLFP

>LmigOBP10

AISESMSRAEEAASKIDIPELFEECNETFTIPKVTLNYFFSHGRLQNENDYGSKCFVHCLTDRSGEIDSDGNFDVDLIKVMTRRFPNETNIEGLNEMVETCVADRGETDFCERAYGLVSCLVKEKLARLGNSH

>LmigOBP11

VATMRSLLPVAVSAVLLVAPSKTLEPDFTKGISDVKACMASENLDSLDALRTNKEARTAEEKCFIGCIMKFVEVLNSDGQYDVALFKDHINGCPEMAKDQQKKAALLEVAESCAGKASACSGHCECGVIVANCL

>LmigOBP12

AVILTAASTLWFAAAAFAAMVTTEIPTEDILQRVQVCNKTYPVSQEMLRSLASTGGLLSDESDVNTRCYLECYERLGGTVNKDGKFNPEKAVTLLVSYYPKIAELGVDSVTEILKNCNSKSGTGQCMTSYLIRNCFIAGLNAKSPHTSVFDTSSSHI

>LmigOBP17

MKAFQICTLICAVVAHCMCDKEEAMKILRASVDKCSAGYGLSRETTQYIVRHNFTIKDENDENQRCFVQCVGQEMGDFNSEGIFDVDHATETAEKWLEWNGRTKSNLREEMEECAKITGTGTCMTTYLITKCAMKAGE

>OasiOBP1

MWARLSDCAALLLLLASAARAWDVNMKLTGRIMDAAKEVDHTCRTSTGVPREMLHRYADGQTVDDDDFKCYLKCIMIEFNSLSDDGVFVLEEELENVPPEIKEEGHRVVHSCKHINHDEACETAYQIHQCYKQSDPELYSLVVRAFDATIGDD

>OasiOBP2

MKAAMAPLATATAAMLLLLAAAVRGQDDEMREMMDQLHQTCVGESGVSEGNIDAARKGNFIEDANLKCYMKCIFVQMTCMSDDGVFDADTAIAMLPDNLKDVASKALNACKGEKGSDACDTAFKINQCLFKQAPKDYILV

>OasiOBP3

MDKASAAAATAFLLIAVAALHAQALSLEQLRQTSKIVRNMCLKKTGVDLALVEGIQEGQFPDNQDLKCYMKCCMGAMQVLRQGRYNVNAAKNQAEKMLPPDLKDRFLSMLDACSDRGDGADDDCEMAYQLTKCSYETDKEIFLFP

>OasiOBP4

MRTSAAAAAAATGAALLVLAAVASAMEMTPEFMEIVNKCKTEHEPTEDELKGMMALKVPESSNGKCFMGCVLQEIGVVKDGKFDKEEAKKHAAAKMTDKDELEKHMQLIEKCSQEVGGETDSCGIGPKLMECIKQFAPEFDIALPKPSE

>OasiOBP5

MRTYLTLVFAAAALFAVAKADAEKVKEAVEKCKSSENLDSLDGLKSNKAPSTEEEKCFIGCMMMDMKLLSSDGQYDAASTKEMINSCEYLKDKPDEKSAALEVADDCAGKATGCSGHCECGPKAVGCLINGMVDKGYEESFARIDKMLQNLE

>OasiOBP6

MGAAVAAAVLLLVAVTNAEDSLMEIVIREVKGCMESEHLNSIGDLRSYNDASSPEQKCFLGCMLKKFKALDADGQYDAEGLKATIEHCPRMKALPNVQKAALQVADECAGKVTGCSDYCSCAPLAAKCLHEGMKNKSFQTIFIALDEALDKMQS

>OasiOBP7

MRTILPLAVSAMLLVAPSKTHEQDFTKGISDVKVCMASENLGSLDGLRANKEARTAEEKCFIGCLMKFVEVLNSDGQYDVALFKDHINRSPDLAKMQQKKAALLEVADSCAGKASACSGHCECGVIVANCLAEGMEAKGEETIYDLLEKIFAKMDA

>OasiOBP9

MSALFTCCVAAWLLMAAALLQPTKGDEVWHNTDIPATMAECNATFRLGWRCWDNLLSDGHVIDESKYQQKCWFYCLLDKTGAMHADGAFDKDLLKTVLQGFPNGSSLAHLDETTYTCVAQRNEVDLCERAYAVVKCIMTEELSRMHHSS

>OasiOBP10

MKALLVACVAALGCLAVAVAAISESMSRAEEAAAKIDLPELFEECNETFTTPKATLNYFFSHGRLQNENDYGSKCFIHCLTDRSGEIDSDGNFDVDLIKVMTRRFPNETNIEGLNEMVETCVADRGETDFCERAYGLVSCLIKEKLTRLGHSH

>OasiOBP11

MSLAARLFSVTLLLAPVLFSDISTAGEVFTMSQIKAAVNECNDTYFLSQKNWDSVFTTGSLEDEKDLVAKCFFECVLEKTGAMDEKGTINSDITKAVFLASHEGTGTPVQGHDELIDMCVPGRDETDICEKGYALVKCVTLEELSRRHARK

>OasiOBP12

MFYFYAFTLCCLLWVLFHCSVNCVDIDIETIWRECNETFPASEESLISFGKNGTIPDENDSTARCFADCYGKKTTMLTSDGSLNWTTLDFIMRSYNMKPTATETFGKCQKDTSNVECMKSYLSLRCVAETIASLSNIR

>OasiOBP13

MVNHHQGVVAIAAALTAMAAAAPSSIAEATRFSKETVSKCQEKWQVSEEIIEEMQRNKGALPNEDSVEQRCFAECVAKEMGMINNGGGVAADKIVKMLEAVFQMASKETGEKLKLDSRALKRDLEACQFKGEDDECTNSYDTLKCLRTLGTSDNMRRYVTKES

>OasiOBP14

MFYFYAFTLCCLLWVLFHCSVNCVDIDIETIWRECNETFPASEESLISFGKNGTIPDENDSTARCFADCYGKKTTMLTSDGSLNWTTLDFIMRSYNMKPTATETFGKCQKDIPTSHQLKTLCTSLSLKHVNPSHQFHRGRGHGWLLKHVNRGPCTQRCKYT

>OasiOBP15

MRTSHVYTIFCAIIVTCYCDSVEVSDGPEEATMMKCAVELGFGHDEIQRIKSSPIPDETNENERCLMKCIGRKMKYLTSEDIVDVHHLLELSGEMIEKEGYTKSEMRQMLVECTKKTGTEKCMTAFKNLRCLMNAFK

>OinfOBP1

MAAAGPRGHLVSTVPTADMRTCLTLVFAAAALFAVAKADAEKVKEAVEKCKSSENLDSLDGLKSNKAPSTEEEKCFIGCLMMDMKLLSSDGQYDAASTKEMINNCEYLKEKPDEKSAALEVADDCAGKATGCSGHCECGPKAVGCLINGMVDKGYEESFARMDKILEKLD

>OinfOBP2

MKGFQICTLICAVVAHCMCDKEEAIKILRASVDKCSTGYGLSRETTQYIVKHNFIIQDENDENQRCFVQCVGQELGDFNSEGIFDVDHATETAEKWLQWNGRNKSNLREAMEECAKITGTGTCMTTYLITKCAMKAGE

>OinfOBP3

MRTILTVAVSVMLLVAPSKTHEPDFTKGISDVKVCMASENLDSLDGLKANKEARTAEEKCFIGCLMKFVEVLNSDGQYDVALFKEHINRSPDLAMDQQKKAALLEVADSCAGKASACSGHCECGAVVANCLAEGMEAKGEETIYDLLEKIFAKMDA

>OinfOBP4

MFYFYALTFCCLLWLLFHCSVNCSDINIWRECNETYPVPDATLISFRNNGSIPDENNITARCFTDCYGKKTNMLTSDGSFNWTTLEHILSNFKMKHTAMDVLGKCRKDPSDDECMQSYLSLKCVAETILSLINAR

>OinfOBP5

MSALYTCCVAAWLLMAAALLQPAKGDEMWHNTDIPATMAECNATFRLGWRCWDNLLSDGHVIDESKYQQKCWFYCLLDKTGSMHADGAFDKDLLKTVLQGFPNGSSLAHLDETTYTCVAQRNEVDLCERAYAVVKCIMTEELSRMHHSS

>OinfOBP6

MRTLLLFSVSALLMAADAEADEATQTKVMADIKACMASENLGSLGQLRANSEARTAEEKCFVGCMMKNLHVLNSDGQYDLALLKERANDCPELARDPRKKADTLLVAEACAPQVTGCSGYCECGVIAGNCLARGMEAKGHETIYDFLRKTVDKMDV

>OinfOBP7

MKAAMAPLATATAAILLLLAAAVRGQDDEMREMMDQLHQTCVGESGVSEGNIDAARKGNFIEDANLKCYMKCIFVQMTCMSDDGVFDADTAIAMLPDNLKDVASKALNACKGEKGSDACDTAFKINQCLFKQAPKDYILV

>OinfOBP8

MFYFYAFTFCYLLWMLFHCSVDCVDIDIGTIWRECNETFPASEEALISFGKNGTIPDEKDSTARCFADCYGKKTTMLTSDGSLNWTTLDFLMRSYDMKPTATETFGKCQKDTSNVECMKSYLSLRCVAETVESLTDIR

>OinfOBP9

MKALLAGCVAALGCLAVAVAAISESMSRAEEAAAKIDLPELFEECNETFTTPKATLNYFFSHGRLQNENDYGSKCFIHCLADRSGEIDSDGNFDVDLIKVMTRRFPNETNIEGLNEMVESCVADRGETDFCERAYGLVSCLIKEKLARLGHSD

>OinfOBP10

MALAVRLFSATLLLASVLFSDISTAGEVFTMSQLKAAVNECNDTYFLSQKNWDSVFTTGSLEDENDLVAKCFFECVLEKTGAMDEKGTINSDITKAVFLASHESTGTAVQGHDELIDMCVPGRDETEICEKGYALVKCVTVEELLRRQARK

>OinfOBP11

MDKASSAAATACLLIAVAALHAQALSLEQLRQTSKIVRNMCLKKTGVDLALVEGIQEGQFPDNQDLKCYMKCCMGAMQVLRQGRYNVDAAKNQAEKMLPPDLKGRFISMLDACSDRGDGADDDCEMAYQLTKCSYETDKEIFLFP

>OinfOBP12

MKLTGRIMDAAKEVDHTCRSSTGVPREMLHRYADGQTVDDDDFKCYLKCIMVEFNSLSDDGVFVLEEELENVPPEIKEEGHRVVHSCKHINHDEACETAYQIHQCYKQSDPELYSLVVRAFDATIGD

>OinfOBP13

MRTSAAATGAALLALAAVASAMEMTPEFMEIVNKCKTEHEPTEDELKGMMALKVPESSNGKCFMGCVLQEIGVVKDGKFDKEEAKKHAAAKMTDKDELEKHMQLIEKCSQEVGGETDSCGIGPKLMECIKQFAPEFDIALPKPSE

>OinfOBP14

MRTLLIYAIVCAIIVKCYCDSAEVSDGSEEDAMMKCAEELGFGHDEIQRIKNSTVPDERNENERCLMKCIGQKMKYLTSEGIVDVDHLLELSGEMIEKEGYSKSEMRQMLEKCAKKTGTETCMTAFKNMRCLMNRSK

>OinfOBP15

MVNHHQRVVAIAAALAAMVAATPSPIAEAIRFSKETVSKCQEKWQVSEEIIEEMQRNKGALPNEDSVEQRCFAECVAKEMGVINNGGGVAVDKIVKILEAVFEMASKETGEKLKLDSRALKRDLEACEFKGEDDECTNSYDTLKCLRTLGTSENMRLYVTKES

>OinfOBP16

MASALCLMAAAHRRHSHRYGIGIKNLNETMDFCNKTYPVSVDTLLERALNNGTLPDETNENARCFIECVERAKGTVNTDGTWNTTRAKQIEIDMNLALGRNISQDISNIVDECSTNSGSGSCMTVYLIKKCIDSRMSAFREPSQHNTHE

>OinfOBP17

MAVTLTAASALWFVAAAFAAVVTTEIPTEDILRWVQTCNKTYPVSQEMLRSLASTGGLLSDESDVNSRCYLECYERLGGTVNSDGKVNVEKAVTVMVSYYPKVAELGVDTVTEILKNCNSKSGTGQCMTSYLIRNCFIEGLNAKSPHTSVFDTSSYYI

>OinfOBP18

MAAAAAHLWCLLPPLLLAVCCALAAPSITSAEMRMDMMVIQHCNETHPVALIDMNKALINKKIEPQNTVFKCFVFCLLNKYEWMDDEGGFLIANMKHNLSDSHLDQLSIDFIVYKCSATGSSDKCERAYRFTECFWGEVTKFPENSDEKYDDPNLFALYQ

>SgreOBP1

MWARLCNCAALLLLLVAAAQAWDVNMKLTGRIMDAAKEVDHKCRGSTGVPREMLHRYADGETVDDDDFKCYLKCIMIEFNSLSEDGVFVLEEELENIPPEIKEEGHRVVHSCKHINHDEACQTAYQIHQCYKQSDPELYSLVVRAFDATI

>SgreOBP2

MASHCHATVAAVVVAAAFAAVVAESPPHMAAFTFTKEMLSTCQEKSEISQEMVDEMQKNKGLLPDESSVAQRCFQECMAKEMGLLKNGGGVAVDNIVTVLKAALQMASEGSEDTYTIDTDAVTRDLEGCQFEGEDDECNNSHDTMKCLRSLGNPENMKRYITKES

>SgreOBP3

MLLAAPAKADEPDLTKAIKDLKDCMASENLDSLDGLKTNKEATTTEEKCFIGCMMKSVHVLNSDGEYDVDFLKEHINHCPELMKDQQKKAAAIEVAESCAAKVTGCSGYCECGVVAGDCMSEGMEAKGYETIYEWLEKVIVKVDA

>SgreOBP4

MKSYFAFVFAAVALFAVAKADPAKLKQAVEKCKASENLDSLDGIKANRQPFTSEEKCFLGCMTLDMKFLSADGQYDAASTKQMINNCEHLKDKPDEKSAALAVADDCGKTVTGCNGYCECGPMTVGCLIKGMMAKGYEESFARIDKVLQKLDG

>SgreOBP5

MDKGTTAALTASLLIVVAAVHTQALSLEQLRQTSKIVRNMCLKKTSVDLALVEGIQEGKFPDDQNLKCYMKCCMGAMQVLRQGRYNVNAAKNQAEKMLPPDLKDRFIAMLDACSDQAVGEDDCEMAYQLTKCSYEADKEIFLFP

>SgreOBP6

MKAHTASLATATVVILLLVAAVVRGQDDDMKEMMEQLHQSCLGESGASDANIDEARKGNFIEDGNLKCYMKCIFVQMTCMSDDGVFDADTAIAMLPDNLKDVASKALTACKDEKGSDACDTAFKINQCLFKQAPKDYILV

>SgreOBP7

MATTVLSPAALLLALLLAAGSVTARRGPRPFGRCASSVGDIDRDTMMKIVRNFEVPDETSDDQKCMLRCALMTNRLVRDGVLDTRQILMNIRADAHFAGRMASVYGQNITLDMDRLSNDVEACVTSEESPDCDTVYNQFKCVSDLISNGNMASYASFVEVDGSETGDWMRRRQMLASRPHGPPGPMGGHWGPPPPHHRGGPRGGGRPPPPPPESDENDVEELE

>SgreOBP8

MRTSAAATGAALLVFVAVVSAMEMTPEFMEIINKCKAEHEPTEDELKGIMMMKVPESEHGKCFMGCVLQEVGVVKDGKFDKEEAKKHAAAKMSDKDELEKHMQLIDKCSQEVDGETDSCGIGPKLMECIKQFAPEFDIALPHAPSE

>SgreOBP10

MESAMKTLLVVCVAALGFLVAAEISESMSRAEEAAAKINLPELFEECNETFPIPKVTLNYFFSHGRLQNENDYVAKCFIHCLTDRSGEIDSEGDFDVDLIKVMTRRFPNETNIEGLSEMVDKCVAGRGETDFCERAYGLVSCLVKEKLARLGHSH

>SgreOBP11

MNTLFTCCVAVWLLIAAALLQPTKGDEMLPNTDIPATMAECNATFKLGWRCWDNLLSDGHVIDESKYQQKCWFYCLLDRTGAMHADGAFDKDLLKMVLQGFPNGPSLAHLNETTYTCVAQRSEVDLCERAYAIVKCIMTEELSRMHHSS

>SgreOBP12

MKWSLWLTATIALVLQLSISEGLKCHTDEDSQNPDEFQEVAAICMKNTSGSELNRSDRENKRNGNNYHKNNFGNTNDNWSSGGMGQTFPGYNSENEGYGLHGSGRCTANNDGYNNNRNNMNQNNMNGMRQKPRNRNRRSGQQSEAANVDLEDIEPCAVHCIFRQMGMLGDDALPDRSAVAKVMLRGVKDTEVKDFVQEAVEDCFDQVESDRKGSKCDLSKNVALCLRQKGRENCEDWGEQEDDQQSNQNKNGNNNGNNSNNSNNQYGNKKWN

>SgreOBP13

MDKCSFHFCLTNTLIYFSLELSSVLPWITRAEVMKRVNVWTASDELRKKLLDALEECIITENEDLNSSLWSPIKGSPPYGGNTWNIGVATANSNKSINQWRSYNEMMGNRTTSVNRDQKVHIDGVYWKNDSDDQDYKNWKESKCFNRGGNHQMQQRCRRSSELPGGNALSSCVDQCLFVKLQVVDKNGLPVEALFMELLDTSIPEQQMRRKARSELHYCFQKMASVAEEDTCTFGKQFASCLDLNVQDIKKHQSNSSNINKLH

>SgreOBP14

MIFSVRFFTVTLLLGAVLFDGICRAEETFSKNQLKAAVNECNDTYFLSQKSWDSVFTTGSLDDEKDLVAKCFFECVLLQTGAMDDKGTINSDVTKAVFLASHDGTAVDGHGELIDMCVPGRVETDTCEKAYALVKCVTVEELSRRQAR

>AglyOBP3

MISSTFYTSLMFGIVMLISCSFGRFTTEQIDHYGKACNATEDDLVVVKSYKVPTSDTGKCLMKCMISKLGLLNDDGSYNKTGMEAGLKKYWSEWSTDTIESINNKCYEEALLVSKDIIATCNYAYVVMACLNKQLDLDKST

>AglyOBP4

MRGNYSLVVFLLFGFGLLEIYCQKQETSGKCRAPDKAPLNLEIIINICQEEIKSALLQEALDILNDGTLEQNTPSYSRSKRDADEDLSNEERRVAGCLLQCVYKKVKAVDETGFPVVDGLMKLYNEGVQDRNYYMATLSAVRHCISIAQQLKQQQPSKSFDDGQTCDLAYEMFECVSEKIEENCGVENKSNNLSQRQV

>AglyOBP5

MKMSTNGATMKCVAIAVVLFQMSVIFAEAGHQRRGKELLDTEDSDFFRCKQASRKSCCGPENAMKRFGDKDKVAADECYAQVAEKFATVTATTPKQDLFSGEAVKITKKKQFCLHECIGKKNKLLTEDGSLNKTFIADYAMKSVFKEQWQKQIGQKALDKCLEETYIPWPAEETENKCNPVYVQFQHCLWLEYESNCPDNKIKLTKKCEKTRNRYRMQKSPSNQ

>AglyOBP6

MQKVVFLCIFAIICQTVFTVGFERTWILRQKRVTNDDECRTLIPSSEKKLPTCCQMPNILPGLDNAWEVCFEKFKQFKDKHATKEYKEMAHGNEPPCLFQCVFMQSGLTTSDGKVNEDAVIKKMAEGMDNDEKWKSIWRNTFNKCLNDVKQEDKEQIKMTNTPTGRLMKCFLRDLYMNRPKNVWVESSECSNLKDLVEKCPKMPPPVFKSPPKLI

>AglyOBP7

MVARKRMYMLPATVLLAVVAATILKDSDAYLSEEAIKKTQKMLKNVCSKKHSVEEEVFTDIKKGIFPENNNNIKCYFACNFRTMQMVNQKGILDKKMFKDKMTMLAPPNVLAILLPPIEQCIGNDKDTEICRSSYNFIKCAHRVDPKSLEFLPL

>AglyOBP9

MIIKKTLLVSGFVLFGCMFSINKAADDADAKDKELMSKLITVAFKCFKDADWGTCGEMITTKYDITQAKYKQCTCHMACAGEDLGLINSNGQPEPAKFLEYVKRINNSVIKSQLQHIYDKCQNVKGTEKCDLAEQFAICAFKESPEMKERVTKLIEMLVKMKPKSK

>AglyOBP10

MEHLRGTNVVFAIVMALLVVQSSTRPQPDELDDIKKTLYNACSEKFPLTEEIKNNVKNSIVIDDQNFKCFLRCCFDEMSLIDEDGIIDGESLAAMAVDKIKPVAEKIVHDCLPAGKQEKQDGCEASFKFFSCGIKLNPLTIELLPLQ

>DponOBP3

MHCSRACLIVFFSICGLSSSLKITLPPELQEYVDDLHKLCLEKGGLTENDHQTYDINHKNEKMMCYMKCLMLESKWMKSGGEIDYDFIETQAYPEVKDLLLNALNKCRTIEEGADLCEKSYNFNKCLYDADPVNWFFV

>DponOBP21

MALTTWVLSIMLILPAIRALSDEMKELAQMLHNTCVAETGVNEDFIQKVNAEKIFADDENLKCYIKCLMAQMACIDDDGIIDEEATIAVLPEEYQALAAPVIRACGTKHGANPCENAWLSHRCYAEMEPSAYMLI

>DponOBP20

MKVFVVLCIVLFAFTLIVSAKKNKSNDEEKAKSYKKVFKECQKKDETRVDASIIRKLKKHKQVDLPANFGEHKLCVFTGIGLLKADNTVDEDKLKKKIASAKPQKDIVDNIVMDCTSSKSTLQETALNLDKCLTTYSIEF

>DponOBP19

MKAMFVTLTVATVVVFASADLTEEQKQKIVANGKACVAETGADPELIKAARQGKFADDAKLKAFALCMSKKSGFQNEAGEIQSDVVKQKLGLAIGDEAAAKKLVEKCLVSKGSGEETAIETFKCYYENTPTHIAVF

>DponOBP18

MNGFSVFFLLLLAAVVKSDFDFSNYKEFENLAGDQREKAIKLFKECMAETGATHEMMEKSVEGDIPDDIVFKNHLVCIGKKSGFIDENGMHIKEKLKEKLTLLLGNEELVDKILDKCFMEKGSPQDTAFELAKCCHREYHN

>DponOBP17

MQVTMNQGWFLLLVSVVSVFAELDQTSLPPEAKELMAALHKNCIEQVGVSEADVDKLRAANFEEDANLKCYTRCLMAESGVMDENGAIDIEAFGEILPEAIRGNIQAIFRSCSLTKNDIVDQCVKAYEMVKCWHKENPESYFMI

>DponOBP16

MKLMWILVLGAALKSTEGAMTEAQMKAALKLIRNVCQPKNKATDAQIAAMHNGDWNQDKNGMCYMNCVLNYYKLQLPDNSFDWETGLKVVESQAPPSMAGFIMETIKSCKDAVKTGDDKCKAALEITKCLYDQNPEKYFLP

>DponOBP15

MGTTIFLLVGLFMMTNAYVPNVNDKIRDFCIDDSGVSIEMVENLLANPEKELIDVESCYVHCIFTEMGLLSENGNVEIENFKSLKASEAPYIDLNCLEEIKSIDHCNEMMILRACHV

>DponOBP13

MSNLLKLSIAFAVVSVISCQDFTEEQRKKIIENRQQCIEETKVNPDLIEKADLGDFAEDQALKCFTKCFYQKAGFVNDKGEVQKDVVEAKLPPQADKKRALEIVDKCALKGKDACETVYLIHKCYFEHTHPEADEKTAKDGKSEEKKA

>DponOBP12

MHFQWLTNVSVFLCILGVAQLVAAGKPNDLFTRITPGDVEVCGKDTGVDRKDFEEAREKGALNHSMLCFLKCAMEKAGFLKDGHLEIDQAKEASPDKMTEPVVECFKAVGPISTCDDIQKVENCLPGS

>DponOBP10

MQLLFVAVLVIALVQVNSLTDKQKELLTQHYNQCVAISKVDQAVLQKARAGDFANDPNLKTHIKCISEKIGFQGTDGKFRRDVIEKKLKETLPGDNAKNAKLIETCVVANKDPQLQAFNAFKCLYTNAKINLL

>DponOBP9

MKSLAVVFAVLIAASLADQVQDIWDRVHQQCQQSPNTHVPQEIFDQLKRGETPILPANFGLHANCMLKKMNLQDNDGHIISSGVKEAAQRHYQSAEKINQIVKDCSATKKTKEETALNLFTCLGQNRVNIG

>DponOBP6

MKSIVALFVCALTATALADAEINETAFKAGRDRIMAMSRTCDENPATAVDQKALKKYLQSNGPAPANGAAHALCITKNLGWQNEDGSVNKPVITEKVKAIFGSVDAKVQQYIEECTEAKATPEDTAEQLLKCYRKHSPKIE

>DponOBP4

MNTALKVFLVALAIPTIMGLSDEMQELANQLHTTCIGETGAAEDAITNARNGDFSEADSFKCYIKCLLSQMAIIDDNDGTIDVDAMVAVLPEEIQEATEPIIRKCGSIIGANPCDSAWLTHKCYYKEGPEHYFLI

>DponOBP2

MKQLVMVVLTALCVVHCKGLECGLSKISSEHFRKIASECVKDNETLNRIWELTSETSMEEDSVSSDEEVPVTKGREAPNFHDLGSSAHRNMKMSGASRTKRSRKGFNNESPMSNVQKKSSPASTTTEHTTTMQSEENEENAAANNVEESGEVCILQCIFEKLEMTDTNGLPDHKKVASALVKSASGRETQDFLQDSVDECFQETEEGDFENSCEYSTKLVTCLAGKGKSNCADWPVGDLPF

>DponOBP1

MLTKTILIWAAILLTVFIPKGNCRLTEKQLAAAIKLVRNMCMGKSKANPEDIEKMHQGNWDVDYQAQCYMWCGFNMYKMLDKENHFDKKAALQQMDQLPIDLQEYVVKCMDQCENAVTNFDDKCVVAFEYSKCLYFCDPEKYFLP

>DponOBP5

MSEKTHFALVALLLTCLVNIIDADQREKAVEFQRDCMEAHGLLEDELHEIMDGKPIQNEAFYFHFFCVVKKAKLISDNGIVNTDHFEENLKGVIDEENMAHVAALTRRCLIQRDDIFTTIKMAIDCFYSSEHKL

>HarmOBP1

MSKFTFFVLCVVAVSLSKVYASDEDKAKLHEALKPLVEECMKDHEVSLDDLKAAKEAKSADGVKPCFLACVYKKAEVLNDKGEFDADHALEKLKEFVSDEDVLAKVAEVGNTCKAVNDKAVSDGDAGCERAALLTACFLEHKAEILV

>HarmOBP5

MSKFTCLVLCVVAASLSQAYASEEEKAAFREAIKPIVEECSKEHGVSHDELKSAKDNQNADSIKPCFLGCVYKKAEVFNSKGEYDVDKALEKLKKFVSNDEAYAKFAEVGKKCASVNDKAVSDGDAGCERGALLTACFLEHKAEVPL

>HarmOBP3

MSKFTCFVLCVLAVSLGEVRSNALEKAAIRAAVYPLIVDCAKEHGVTLEQLKAAKASHSAEGINPCFQSCVYKKTGIFNDNGEYDVANAKTKLQKFVTDEDEYARIAEVGKTCASVNDKSVSDGAAGCERAALLTACFLEHRAQIII

>HarmOBP4

MSKLTCVVFAAVAVVFSNVNADDETRASFRQVLGPLVMECRNEFGITEDDLKKAQQERSPDALKPCFIACVFKKFGIITSAGKYDSDASISRIKDVVKNDDLLAKLKSVGEKCNSVNDASVSDGDAGCERAALLAKCFIENKSELSI

>HarmOBP2

MMDRKRLCLLIIALFLAQGSDAMSRQQLKNSGKMLKKNCMNKNQVTEDQIGSIDKGKFVEDKKVMCYIACIFEMTNVVKNNKLNYDASIKQIDLMYPPDLKESAKAAVEKCKDVQKKYKDICEASYWTAKCMYDFKPEDFIFA

>HarmOBP6

MSKFTCLLLCVVAVSLSKVHATEEEKEAIRAAVRPIMQECGKEHGVTLDDLKAAKAAHSADGIKPCFQSCVYKKAGIFNDNGEYDIANAKTKLQKFVTNDEEYARIAEVGKMCASVNDKPVTDGAAGCDRAALLTACFLEHRAQIII

>HarmOBP13

MFTGTLPLVVFLATFAYGGKEKPVFSDEIKEIIQTVHDECVAKTGVAEEDITNCENGIFKEDPKLKCYMFCLMEEASLVDDDDAVDYDMLVSLIPEEYVDRTTKMIFSCKHLDTPDKDKCQRAFEVHKCSYEKDPDLYFLF

>HarmOBP8

MLLIEIVKFLTLVAMCEAMTMKQIRNTGKMMRKSCQPKNNVADEQIDPIAEGVFNEDKEVKCYMACIMKMANTIKNGKLNYEAAIKQADLLLPDDIKEPAKEAITACRKVADAYKDICDASFHITKCIYTQNPGIFYFP

>HarmOBP7

MFRFGVLSFVVLLFCMESSYALSSEEELSIKEALHPFVVECAEEYGMTEEMFEEAKKKGSAEDIDPCFMSCFLKKTGFFDDSGKFDAEKSISFAKEHITSESAIKFLEAGAGECVKINDEDVSDGENGCDRAKLLFDCLTELKKKMSE

>HarmOBP9

MCKFSVLFLYSAVMAVNIWSASCISEEDKAAIITAIAPLAQNCGSECGLDNDDFEKYKEDGSDMDPCFKACLMTQMGVLDKEGKYDGKGLHKAMEEADYPGDKDDAQKFLDELDRCFDAKGDNSGSDEEAKMKRADVLFRCMQDMKEK

>HarmOBP15

MGSRHVFFALVVLAVSVKKEKPSKHPMPYITSRFVKVLEECQHELKLNEHILEHLFHFWKLEYSLLGKDPGCAIICMSTKLDLLDLYGRMHRGNAAEFAKKHAAGDEVPSKIVTIIHFCQKKHEQDGDECLQVLEVATCCRTGLHDLNWQHQVEVIVPDVLTEI

>HarmOBP17

MKTFVILAACVMLVQASGLTDEQKEKLKKHRSECLTETKVDEQLVNKLKGGDYKTESEPLKKYALCMMMKSELMTKDGKFKKDVALAKVPNAADKPTVEKLIDACLANKGNTPHQTAWNYVKCYHEKDPKHAIFL

>HarmOBP16

MFKSIVFCALIIVASHADVLKKRDSKGASLKPLSVCCDIPELGDPKNLEKCSNPKMPGPCDDIQCIFEASGFLIDRNTLNADAYKNHLMKWQEEHKPWKVAVDRAIEECANNQTRQYLDFPCKAYDVFTCTGIAMLKKCPEAAWKC

>HarmOBP18

MKSFVVFCVLVAGAFAANVSLPPKQNEKANQIATECMKESGLKPEVLAEAKKGHISDDEHLKKFTFCFFKKAGIVSEDGKLNTEVALAKLPPGVDKAEAEKLLETCKGKTGKDVTDTVFEIFKCYHHGTKTHILLGF

**File S4.** Amino acid sequences of 83 CSPs of *Ceracris kiangsu* and other insects used in phylogenetic analyses.

>CkiaCSP1

MGTSLDSQSSTLPTITMQKCTLALLLACLVAAAAAYTTKYDNIDLDDILRNDRLLKKYHECLLSDSDASCTPDGKELKAAVPDALTNNCAKCNEKQKAGAEKVISFLIKEKPDLWTPLENKYDPTGTYSQQYGDELKSVSA

>CkiaCSP2

MKAALVLLSALAVIVVAAAEEKYTTKYDNVNLDEILANDRLLNKYVQCLMEEDDSNCTADGKELKSVVPDALSNECAKCNEKQKDGTKKVLKHLINHKPDIWQQLKAKYDPEGTYSKKYEDKEKELHE

>CkiaCSP3

MARLSFALSLLSLLAALAAAQDKYPDTFDQLDMKALLANKDRIEAAITCLNEDSNSSCTPTAEFLKSVLAEVVSTDCGKCTEAQKKKVGAFFAHVSQHYQQQMQELLDKYDPTKEYRAKYAQSWAADGIKVW

>CkiaCSP4

MVAAAPEEKYQSPYEGTDVDTILQNDGKVQAILKCLLSDTDDVCSKEDKQSKDMLPEALATQCAKCTEKQKQGMAKFFAHVSSKFPDMFKQLAAKYDPTGENLPKFTTGRSFAVYSTW-

>CkiaCSP5

MTRSNMSPLARTVVFLSFLAVLATASAQEGYQTAYDNVDVDVMIQNDTLVQTMMKCLVSATDDLCGPENKHVKELLPEMLATACRKCTEKQKHSMTKFFGQISRKHPDIYKQFLEKYDPTGELLGKIKTTAW

>CkiaCSP6

MLLCQHSAAVVAVFAAAACMVAVTSAAPPKCASIAANDKKYTTRYDSIDIDSILKSDRLLRSYMDCLMDRGPCTQEGCLLKAAIPDALQTECSKCSDVQKKQAGRVLAWMLENKRNYWDELIAKYDPEGNFRKKYGYDEDDDEDKK

>LmigCSP1

MQKCTLALLLACLVAAAAAYTTKYDNIDLDDVLHNDRLLKKYHECLLSDSDASCTPDGKELKAAIPDALTNECAQCNEKQKAGAEKVIRFLIKEKPDLWTPLEKKYDPTGSFRQKYDQELKRVSA

>LmigCSP2

MKSCALALLLVGLVAAAAAYTTKYDNIDLDEILHNDRLLNKYHECLLSDTDTPCTADGKELKAAIPDALTNECAKCNEKQKNGAEKVIRFLIKEKPDLWTPLEKKYDPNGTYRQKYGEELKKVSS

>LmigCSP3

MQALTLVLFALVASAAAYTTKYDNIDLDEILNNERLLKKYHECLMSDSDASCTPDGKELKVSIPDALVTDCSKCNEKQKEGSNKVIRFLIQKKEDLWKPLQAKYDPEGTYLKKHPELLSA

>LmigCSP4

KYDNIDLDDILHNDRLLKKYHECLVSSSDASCTPDGKELKAVIPDALTNECAKCNEKQKAGAEKVIKFLVKEKPDLWEPLEKKYDPSGSFRQKYGPELKKVSA

>LmigCSP5

AAAYTTKYDNIDLDDVLHNDRLLKKYHECLLSDSDASCTPDGKELKXAIPDALTNECSKCNEKQKAGAEKVIRFLIKEKPDLWTPLENKYDPSGSYRQKYGQELKRVSA

>LmigCSP6

AAAYTTKYDNIDLDEILHNDRLLKKYHECLLADDDASCTPDGKELKAAIPDALTNECAQCNEKQKNGAEKVIRFLIKEKPDLWTPLENKYDPSGSYRQKYDQELKRVSA

>LmigCSP7

AAAYTTKYDNIDLDEILHNDRLLNKYHECLLADDDASCTPDGKELKAAIPDALTNECSKCNEKQKNGAEKVFRFLIKEKPDLWTPLETKYDPSGSYRQKYADELKRVSA

>LmigCSP8

AAAYTTKYDNIDLDEILNNERLLKKYYECLMSDSDASCTPDGKELKVSIPDALVTDCSKCNEKQKEGSNKVIRFLIQKKEDLWKPLQAKYDPEGTYLKKHPELLSANKNSI

>LmigCSP9

EEKYTTKYDNVNLDEILANDRLFDKYAQCLLEEEDNNCTADGKELKRLIPDALSNECAKCNDKQKEGTKKVLRHLINNKPDVWQQLKAKYDPDGTYTKKYEDREKELHQ

>LmigCSP10

EEKYTTKYDNVNLDEILANDRLFDKYAQCLLEDGESNCTADGKELKKAVPDALSNECAKCNEKQKEGTKKVLKHLINHKPDVWQKLKAKYDPDGTYSKKYEDREKELHQ

>OasiCSP20

MQKCSLALLLACLVAAAAAYTTKYDNIDLDEILNNERLLKKYHECLVSDSDSSCTPDGKELKATIPDALVTDCSKCSEKQKEGANKVIKFLIQKKEDLWKSLQAKYDP

>OasiCSP19

YDNIDLDDVLHNDRLLNKYHECLLSDSDASCTPDGKELKAAIPDALTNECAKCNEKQKAGAEKVIRFLIKEKPDLWTPLENKYDPTGSYRQKYGDELKRVSA

>OasiCSP18

MSEGAEGTAACVGSCRDVTAVYIAAAGGCGRRLSRHSLPTPHRTLANMKSCAFALLLVGLVAAAAAYTTKYDNIDLDEILHNDRLLNKYHECLLADTDTSCTADGKELKAAIPDALTNECAKCNEKQKAGAEKVIRFLIKEKPDLWTPLENKYDPTGSYRQKYGDELKRVSA

>OasiCSP17

MSEGAEGTAACVGSCRDVTAVYIAAAGGCGRRLSRHSLPTPHRTLANMKSCAFALLLVGLVAAAAAYTTKYDNIDLDEILHNDRLLQNYYECLMKDDETGCTPDGVELKRTIPDALKTECSKCNEKQKEGTKKVLKFLINHKPDMWGNLKAKYDPDGTYAKKWEDKEKELHE

>OasiCSP15

MARSSLLLLLALVALAAAENPLISQLENIDVDAVLADPQRVDAAVKCFLSDADDDCNVRSKVIKSLISEMLKTNCAECSEKQKAGVAKFMAHIAKNKPEEMKQLLAKYDPSGEARAKYGDSWRQKGIIP

>OasiCSP14

MHRHSAAAVAAIFVVTACMVTVTSAAPGKCASIVANEKKYTTRYDNIDIESILKSERLLRSYFDCLMDRGACTQEGCLLRAAIPDALQTDCSKCSDVQKKQAGHVMAWILENKRNYWDELIAKYDPEGNFRKRYGYDEDDDEEEK

>OasiCSP13

MSRVARVVVCLCLAAASAQEGYQTAYDSVDVDVIIQDDTLVQSIMKCLVSATDDLCGPENKHVKGLLPEMLATGCAKCSEKQKHSMNKFFGHVARKHPDLYKQFLEKYDPTGELLSKIKTTA

>OasiCSP12

MQTPTLALLIVTAALAAAAAADDRYAKYDHVDVERMLRNQRFVNAAIKCLLEEGPCTPEIRDLKKMLPDALKSDCSKCSAKQKENVRKVVDFMMKQRAADWARLSRKYDPEGLHQKRIEAKLREQQQQQQQ

>OasiCSP11

MKASALLLLLLLTAACVVAAAPEEKYQSPYEGTDVDAILQDDAKVQAILKCLLSDADDVCSKEDKQSKDMLPEALATQCAKCTEKQKHGMARFFAHVSQKFPDLFKQLAAKYDPTGENLAKFSAARRLSA

>OasiCSP10

MAAARQLLVLAAVVAAAAAQFGGTDPGSLLADPQALTQVIRCLLASADDGCSLQGRLLKSVLPKLLQTNCAQCSEAQRQDVAGVLRHLVNDRPEDWQRLADKYDPEGTLRRQHGDEWRARGVNL

>OasiCSP9

MSRLCFALSLLSLLAALAAAQDKYPDTFDKLDLQELLGDKERVQAAIKCLLEAADTECRPAGKLLKSVLAEIVKTDCGKCTETQKQKVAGFFSFVSQNYPEQMQQLLDKYDPTKEYRTKYAQSWAADGIKV

>OasiCSP8

MKAILVLTAVLAVVASVAADDKYTTKFDDIDIDKILANERLLHQYELCLTEDETKCTPEGKELRKDIPDALETECAKCNEKQKEGIKKVIKFLINHKPETWQKLKEHYDKDGKYSEKYKKLEDELKE

>OasiCSP7

MRNSCLAVALLTTVAVVCGGYTTKYDNFDVDQVLHNDRLLKRYHECLVSDSDAACTVEGKELKSVIPDALQTDCSQCNEKQKAQAEKVISFLIHNKPDLWQSLQNKYDPDGSYRKRHDAELKKLSS

>OasiCSP6

MKAALLLVSALAAIAMAAAEEKYTTKYDNVNLDDILANDRLFNSYAQCLLDDGEDRCTADGKELKKIIPDALSTECSKCNEKQKEGAKKVLKHLINHKQDVWQQLKAKYDPDGTYSKKYEARERELHE

>OasiCSP5

MKASLVLLSALAAIALAAADDKYTTKYDNVNLDEILANDRLFDKYAQCLLEEGDSNCTADGKELKRVIPDALSNECAKCNDKQKEGTKKVLKHLVNNKPDVWQQLKAKYDPDGTYTKKYEDREKELHQ

>OasiCSP4

MKAALVLLSALAVVAVVAAEEKYTTKYDNVNLDEILANDRLFDKYAQCLLEEGDSNCTADGKELKRVIPDALSNECAKCNDKQKEGTKKVLKHLVNNKPDVWQQLKAKYDPDGTYTKKYEDREKELHQ

>OasiCSP3

MASNLLVYCCLFAVVAVWVTAEEDKLDAINVDEVLGNDRLVHSYLECVMDDNDSKCTKEGREVKSRLPGLVKTGCNDCTPKQLERAIKTLKHITEKHPEEWKKLKAKYDPTGEYTQKYAETWKQRGVDF

>OasiCSP2

MAGKLTVCCLLGLLALCVEAAPQDPLDSFNVDEVLSNERLLKSYIQCMLDDGEGRCTKEGKEIKKRLPQFVATGCLECTPSQLDRAIKTLKHVTEEHAEDWARLKAKYDPTGEYSRKHADTWKQRGINF

>OasiCSP1

MAIALSARALSYTRRSDMQALTLALFALVASAAAYTTKYDNIDLDEILNNERLLKKYHECLVSDSDSSCTPDGKELKATIPDALVTDCSKCSEKQKEGANKVIKFLIQKKEDLWKPLQAKYDPEGTYLKKHPELLSA

>OinfCSP23

MRACRGLLLLAAYAAAVVTADASMRGKTLVEFANTDWAAYLGDRPAIERHIKCVLDKMPCDESGLKLKSLLPFMMDQNCRKCSNIEKLFTRKLKRMMAKHYPEAYRDMALLARHLQREAAAAAITTTTPITTTVADIDGDTTTVSSSTALSCVCRCRSLYRRRHRHRTATASGRSRQQRDLAFSQPSTAPLR

>OinfCSP22

MAALSLRLALVAAAAVCLASAQQQQQQQRPRVSDQQLDRALADKRYLTRQLKCALGEAPCDPVGRRLKTLAPLVLRGACPTCTPEETRQIQRTLSHIQRNYPKEWSKLVKQYAGF

>OinfCSP20

MRNSCLAVALLATVAVVCGGYTTRYDDFDVDQVLNNDRLLKRYHECLVSDSDAACTVEGKELKSVIPDALQTDCSQCNEKQKSQTEKVLSFLIHNKPDLWRSLQDKYDPDGSYRKRHDAELKKLSS

>OinfCSP19

MKAALVLIAALAVVVVAAAEEKYTTKYDNVNLDEILNNDRLLNKYVQCLMEDGESNCTADGKELKKAVPDALSNECAKCNDKQKEGTKKVLKHLVNHKPDVWQQLKAKYDPDGTYTKKYEDREKELHQ

>OinfCSP18

MAHLQAALCTVATLAALLVAVCAQGGPAQSFARPERLDVDSAIHNETRFQEAMACILGEDDYRLCDHHTVGIKYDGWRMLERGCGGTCTPDEQSVYRFLAYVSHNKPDQWRRILDKFDPDGKLQQSNSEQWRQHGIKV

>OinfCSP17

YDNVNLDEVLRSDRLLNNYFQCLMDETDERCTADAKYLKEVVPDALNNGCSRCRPNQREGAEKVIKFLMNNKPDMWNKLEAKYDPDG

>OinfCSP16

MRKALAPLLILVIAARALPAAADDGKYTTKYDNVDLDEILNNERLFNKYMDCVLDDGNDRCTADAKDLKELLPEMVQTDCAKCSDKQKAKVDKVVKFVRDNKKDAWE

>OinfCSP15

YDNVNLDEVLASERLLNSYYRCLMENTDEHCTADAKYLKEVVPDALSNGCSRCRPNQREGAEKVIKFLMNNKPDMWNKLEAKYDPDG

>OinfCSP14

GKLTVCCLLGLLAVFVQAAPQDPLDSFNVDEVLSNERLLKSYIQCMLDDGEGRCTKEGKEIKKRLPQFVASGCLECTPSQLDRAVKTLKHVTQEHAEDWA

>OinfCSP13

MFRCVFAVLAALVALAATQSQIENIDIDAVLADPAKVDAVVSCFLNDDYQGCNERSKFIKGLIAETVKTNCGSCSDGQKAGVVKFLVHISRNKPESMKQLLAKYDPNGEALVKYGDIWRQNGISV

>OinfCSP12

MVQMEPHFRDATMGRLCFALSLLSLLAALAAAQDKYPDTFDKLDLQELLGDKERVQAAIKCLKEEADTECRPAGKLLKSVLAEIVKTDCAKCTETQKQKVAGFFSFVSQNYPEQMQQLLDKYDPSKEYRTKYAQSWAADGIKV

>OinfCSP11

MSRLLLLVASAALLAAAWVRPAAASFAAPAVLDVDSPTQSEAAFQDTMRCVFGDNDYEVCDSNSVSIKYIMWRLLEKGCNSCTDDELKVFRFMAYLSNNRQNDWQQVLNKYDPQGEFRSRNAAQWKQHGVAV

>OinfCSP10

MAGLRLRLALVLVGCVLLGGAQAQRRGRGRAGGAAAASHDLDVDAVLADERTLAALLRCVVSRGDGPCSEQGKAVKAFVVSAVETNCASCTEQQQAAAEKVVRHLTTRRKSDWNKLVARYDPTGELRSRYRKHWEQRGVRI

>OinfCSP9

MKAILVLTAVLAVVASVAADEKYTTKFDDIDIDKILANDRLLRQYELCLTEDETKCPPEGKELRKDIPDALETECAKCNDKQKEGIKKVIKFLINHKPETWQKLKEHYDKDGKYSEKYKKLEDELKE

>OinfCSP8

MSRVARVVICLGVAAVLATASAQEGYQTAYDSVDVDVIIQDDTLVQSIMKCLVSATDDLCGPENKHVKGLLPEMLATGCAKCTEKQKHSMTKFFGHVARKHTDLYKQFLEKYDPTGELLSKIKTTA

>OinfCSP7

MHRHSAEAVAAIFVVTACMVTVTSAAPGKCASIVANEKKYTTRYDNIDIESILKSERLLRSYFDCLMDRGTCTQEGCLLRAAIPDALQTDCSKCSDVQKKQAGRVMAWILENKRNYWDELIAKYDPEGNFRKKYGYDEDDDEEEK

>OinfCSP6

MASNLLVYCCLFAVVAVWVTAEEDKLDAINVDEVLANDRLVHSYLECVMGDNESKCTKEGRELKSRLPGLVKTGCSDCTPKQLDRAVKTLKHITEKHPKEWKELKAKYDPTGEYTQKFAETWKQRGVNF

>OinfCSP5

MVRPLVALLLLGALVAAQDKYTTKFDSVNLDDILNNDRLLNKYAQCLLDADDRNCTPDAKELKKAIPDALTNECAKCSEKQKEGAQKVVDFLIDNRPAQWKELEAKYDPSGEYRKRYDDRIKKLKS

>OinfCSP4

MKATLAVGVASLALATVLAFGAHFQLLDRIEGDGDEVAIQCMLSDDDEGCTEPLAELKGKLWGVVSTNCGACSDDEREYIIDYFSGLFRRNPHSRKPLQSKYDPTGEYRRKYGKIWRKKGIELD

>OinfCSP3

MRATVTALVAALAAAAVLASGGRFKHLHRLRGDGDQAAIRCLLSDANDGCTEALTELKGNLHIVASSNCGVCTEDERENVIGYFKDLFIRNPKWAQKIQDKYDPTGEFRRKHGAVWRKKGIPVYDLATV

>OinfCSP2

MAKVLAALLVLVTIAAVPLLTAADDGQYSTKYDNLDLDEIFNNERLFNKYVECLLDDGKERCTPEGVELKAMLPELLQTDCAKCHARQKARMDKIITFIRDNKKDVWEKLTAKYDP

>OinfCSP1

MARALALCCLLALFALAADGAPQDRLDNVNVDEVLSNRRLLKTFVQCILDEGDGRCTKEGKDLKQSIPRLVETGCSDCSARQLENGVKVLKHLTENYPQEWAQMKAKYDPT

>AgamCSP1

MKLFVVVALALVAAVAAQDKYTSKYDNINVDEILKSDRLFGNYYKCLLDQGRCTPDGNELKRILPDALQTNCEKCSEKQRDGAIKVINYLIQNRKDQWDVLQKKFDPENKYLEKYRGQAQKEGIKLD

>AgamCSP2

MKLFVAIAFALLALAAAQEQYTTKYDGIDLDEILKSDRLFNNYFKCLMDEGRCTPDGNELKKILPEALQTNCEKCSEKQRSGAIKVINYVIENRKEQWDALQKKYDPENLYVEKYREEAKKEGIKLE

>AgamCSP3

MKFFVVVALALVAAVAAQDKYTTKYDGVDLDEILKSDRLFNNYYKCLMDTGRCTPDGNELKRILPDALKTDCAKCSEKQKSGTEKVINYLIDNRKDQWENLQKKYDPENIYVNKYREDAKKKGINL

>AgamCSP5

MRKVWLLASVVLAFLDFVKSQEVARTLYSTRYDNLDIDTILASNRLVTNYVDCLLSRKPCPPEGKDLKRILPEALRTKCARCSPIQKENALKIITRLYYDYPDQYRALRERWDPSGEYHRRFEEYLRGLQFNQIGGSNGGSGVGNTVLSNL

>AgamCSP6

MKHLTMVAIFAMVVVLASAQKYTDKFDNIDVDRVLSNDRILNNYLKCLLDKGPCTQEGRELKKTLPDALKTNCEKCSEKQRTSSRKVIAHLEERKPQEWKKLLDKYDPEGIYKSKFEKINKRS

>AgamCSP7

MLSAAVIVVMAALVIVGPQPAAANDSQNINRLLNNQVIVSRQIMCVLEKSPCDQLGRQLKAALPEVIQRNCRNCSPQQAQNAQKLTNFLQTRYPEVWAMLIRKYGAV

>AgamCSP8

MLHNLFLSLSLYVSVCGDPSGSTCAAEATTARTQVSDEALDKALSDKRYLMRQLKCALGEVACDPVGKRLKSLAPFVLRGACPQCTPAEMNQIKKTLAHLQRNFPSEWNKLVQTYAG

>DponCSP8

MKIFIVVCCAFIGLVLADTPKYTTKYDNVDLEEIIKSDRLMKNYVNCLLEKGKCTPDGAELKRVLPDALHTECSKCSDSQKKGSRKIMRHLIDNKPEWWTELENKYDKEGAYKKQYREELKKDGIKL

>DponCSP11

MAPFPQSWLQFGALLLLLALVQGQILNGNVYVEKQLLCALDRAPCDNLGRQIKDALPEIIGKNCKACDNKQLSNAKRIARFVQNKYPNVWNDLVRKYGNPTN

>DponCSP6

MKTIIFLVVVASFYGLSSCKPQEKYTTKYDNIDLDAIIRNDRLLRNYIDCVLGKKKCTKDGEELKVHLPDALQSDCSKCSEAQRNGSRKIITHLLKNKRGWFNELQAKYDPAGNYLSKYSEELRKEGIVI

>DponCSP1

MKVVLLLVVVVGVAFGEEYTSKFDNVDLDQILSSDRLLRNYINCLLEKGKCTPDGTELKKNLPDALENECSKCTPKQRDGAKKVIRYLIENKRDYWDEVAAKYDPEGTYYKKYQEQAKKENIKL

>DponCSP3

MWKLVLLGSLLICIGQTLAEVTEKSQYTTKYDNVDINEVVHNERLLKNYVNCLLDRGPCSPDGLELKKNMPDAIETDCSKCSDKQREGLEAMMRFLIDNKPEYWNPLQEKYDPTGSYKKRYLDAKRAEVAIQPAEKTP

>DponCSP4

MLLIISVLIGMALDLTDAKPAAKNYASKYDHIDVGAILNNRRMVNYYSACLLSQGACPPEGVELKRILPEALQTNCARCSEKQATIALMAIKRLKKEYPKIWSELSAKWDPSDSFVKKFETTFESLHGPGRRVESTTSAGNKLDPSEADGNTIDANITQTSPEGSDRVNQPDTTTTPQIITTNPSFSTSTKPAFSSTKRPSPIPGLVPFNTFFTNPPIPIRPIVNLNLGGNIGATVKAIKQVEKMVADIALEKIGIIRSILRPWRKAKKTRYA

>DponCSP2

MKVVLLLVVVVGVAYGEEYTSKFDNVDLDQILSSDRLLGNYMNCLLDKGKCTPDGTELKKNLPDALENDCSKCSAKQRDGAKKVIRYLIDNKRDYWDQVAAKYDPEGKYYKKYQEEAKKENIKL

>DponCSP5

MQCLGLFVVLVLGCSLVAAQSPYTSKYDNVDVDKILKNERVLTNYIKCLMEEGPCTPEGRELRKTLPDALASGCSKCNEKQKDTTEKVIRHLMDKRTKDWDRLSKKYDPQGVYKQRFEKELSARKLA

>HarmCSP14

MNSAIVLCVVALAGMVLARPDGDGDKYTSRWDDVDLDEILENDHLLIPYIKCSLDEGKCAPDAKELKEHIQEALETGCAKCTDKQKEGTRRVIAHLIKKKLQEWEKLKAKYDPEGKYAKKYEKELEEVKNA

>HarmCSP13

MKVLLVLCLFAAAALADDKYTDKYDNINLDEILENKRLLLAYVNCVMERGKCSPEGKELKEHLQDAIETGRSKCTEAQEKGAYKVIEHLIKNELDIWRELAAKYDPKGDWRKKYEDRARANGIQIPE

>HarmCSP12

MNSAIVLCVVALAGMVLARPDGDGDKYTSKWDNIDLDEILGNDRLLVPYIKCALDEGKCAPDAKELKEHILEALETGCDKCTDKQKEGTHRVIAHLIKYKLEEWEKLRAKYDPEGKYAKKYEKELEELKRA

>HarmCSP11

MNSAIVLCVVALAGMVLARPDGGTYTTKYDNVDLDEILANDRLLIPYIKCLLDEGKCAPDAKELKEHIREALENGCAKCTDKQKEGTRRVIAHLIKHKNADWQKLKAKYDPEGKYTHKYEKELEEVQH

>HarmCSP10

MKVLVVLSCLIVAAFAADKYNAKYDNFDVDTLITNDRLLKAYINCFLDKGRCTPEGSDFKKTLPEAIETTCGKCTDKQKNNIRKVIKAIQQKHPKEWDALVKKNDPSGKHRANFDKFIQGSR

>HarmCSP9

MNSLIVFCVLSLAALTIARPDGATYTDKYDNVDLDEILGNRRLMVPYIKCMLDQGKCAPDAKELKEHIKEALENECGKCTEAQKKGTRRVIGHLINHEADFWNELAAKYDPERKYTTKYEKELKEVEA

>HarmCSP8

MKCIYVLSFLLALAAVQAEDKYSTENDNLDIDAVVANVDTLTSFVACFVDQEPCDAVAADFKKDIQEAVTTRCAKCTDAQKHIFYKFILGLKEELPRGYEEFGRKYDPENKHFSALENAVSPA

>HarmCSP18

RPDTYTDKYDNVDLDEVLSNRRLLVPYVHCLLEQGKCAPDAKELKEHIREALENACGKCTDAQQSGTRRVIGHLINKEPEFWKQLNAKYDPNNKYTKKYEKELKEVQEDKQNH

>HarmCSP15

RPESQYTNKYDNVNLDEILVNKRLLVPYIKCALDQGKCSPDGRELKSHIREALENYCAKCTPVQQDGTRRVIAHLINHEPDYWRQLSVKYDRDGKFAVKYEKELRTIA

>HarmCSP16

MKILVLLLAAVVTAQYEEDTYGTDHDDLDIVALVEDKDQFNSFIDCFIDEAPCDDVAETFKSVIPEAVLEVCAKCTPAQKHIVRVFNESFKKKMPEKFQKFKNKYDPEGKYFENFEAAVGAF

>HarmCSP17

ASTYTDKWDNINVDEILESQRLLKAYVDCLLDRGRCTPDGKALKETLPDALENECSKCTDKQKSGSDKVIRHLVNKRPEMWKELSAKYDPNNIYQDRYKDKIEAVKGQ

**File S5.** Amino acid sequences of 23 SNMPs of *Ceracris kiangsu* and other insects used in phylogenetic analysis.

>CkiaSNMP1

MQLPVGLAAGGGGVFFLAVVAGWYGMPKLMHSQMANGLALKKGSDIRQMWSNFSDPIDFRVYILNLTNPEAVHSGEKPVVQEIGPYFYEEYKQKVKLRDHKEDDTVSYNQKVTWLFNQAKSGPGLTGDEMITMPHPLLLGLLLTLERDKPGMLALVNKAIPPLFRKPESIFVTAPARNFLFDGIVINCTVTDFSAKALCTGLKKEAKELKREGDNFFFSFFGHKNGTVDNNRLRVKRGIENIDDLGRVVAFNGENKLATWRGDPCNDLRGTDSTIFPPFSDAKEPIVAFGADLCLSLGAAWERSAEYMGVPGNSYTGEMPDMKGNPEHHCYCPTEDTCLEKGALDLSPCAGAPVMATLPHFYLSSETYLRTVTGLNPTKENHELFMVFETTTGSPMEARKSLQFNMFLHKINKMDLLANVPYALMPLLWVEEGLALEEKYVSTLRMLFRMQGIMSGVKWTMMVMGMGMGGAGGYLYFKRRKELVVGPAEPKKVAAGHGDAPGHPISLESSHSSY

>CkiaSNMP2

MLSARVCGCGRRGLLWGLAAGVVVLALAVALKWAVFPYILTAKIMQAVQLRDDSAAMERFEQLPQPLLYKAYLFNVTNPDQVGLGAKPVLQQVGPYVYEEWRRRRNVTRLWDGSLSYQLETSFHFSPERSPGLSEDDEFTYLNVVMMGIVVQVAEDYASMLSMVEPVLSELLSGGAQLFLRTSARQLLWSGVPTVDCRGNLSAVAVLACSALPSLLPQTVTQAEPGVYTFSFFGFKNGTASQLWRVDSGLEEVSSLGSVLAYDNSPRLKVWSPPDSSCNDIKGTDSTIF

>AaegSNMP1

MLIKNRKNLMLKPGTQMRGMFEKIPFPLDFKLYLFHVTNPDVVMKGGKPRVREIGPYFFEEWKEKYDTVDNEEDDTLTFTLKNTWIFRPDLSKPLTGDEMITIPHPLILGALLMVQRDREAMMPLVSKGMDIIMNPLTTGFLTTRVMDLLFDGILIDCSSHEFSAKALCSGLESEGAVMPFNETHFKFSMFGLKNGTDAGRWVVYRGVKNIMDLGRVVSFNDETEMDIYDGDECNRYIGTDSTIFPPFLTTKDKLWAWSPEICRSIGAEYGGKSKYAGLPMSFFKLDFGDARNEPEHHCFCRDPPDICPPKGTIDLAPCLGAPIIGSKPHFYDSDPKLLAAVDGLTPNEKDHDVYIHFQLLSGTPVSAAKRLMFSMEIEPIRDHAVLGNLPTVILPLFWAEEGASLNKTWTNQLKYTLFLGLRFNTAVKWLTIIIGTIGTIVGGFMHYKRTTKMVNVTPVQSVNGSSAKGKGAGMTVVGHQPDSKGGSVTAPVIPSAKDLLQNSRNLPTVIEGLDKPQKVTVTEMQERY

>AaegSNMP2

MMVMNTELRQDTPQFKRWEAVPQPLDFKVYIFNVTNPYEVQMGRRPRVVEVGPYVYFQYRHKDNIRFSRDRSKVHFSQQQMYVFDAESSYPLTENDQLTVLNMHMNSILQIIDTQAKETITNFRSDVNNTLEKIPVVRVIKRIIEKTTPIQSILQLAEDETYDSLRLINAELNRIFGRPDSMFLRTTPREFLFEGVPFCVNVIGIAKAICKEIEKRNTKTIRVQPDGSMKFSFFNHKNMTNDGTYTINTGIKEPALTQMIEYWNGRNTLDRWINQSAGSSSKCNKIVGTDGSGYPPFREGVERMTIFSSDICRTVDIKYVGPSSYEGIPALRFETDSHFLNEIGPEYGNDCYCVNRIPKAIVKNNGCLYKGALDLSTCFDAPVVLTHPHMMGAAQEYTSLIDGLYPDPEKHQIFVDVEPLTGTPLNGGKRVQFNMFLRRIDSIRLTDRLQTTLFPVLWIEEGIALNEDMVKLIDDSLMKVLTLLDIVQWVMIGSGLLLAIIMPIVYFIKRRPSSGSITPTLTTTTSTVSISDGGGLGGNPQK

>AlinSNMP1a

MGAPLRLGVAGGALFLFGSVFGFWGFHKFLNSQIAQTVQLKKGNEMRDTWATFPVALEFKVYLFNLTNPEEVQNGGKPKVQEVGPYFFDEWKSKGNFEDDSAEDTVSFNMKAVWYFQKDRSEGLTGDEMITIPHPVVFSMIAQVERDKPGALPMLAKALPALFNNLTSPFIAARAMDILFDGLPINCSSKEFGPKAVCTLINANPKGLIKKSPELFLFSFFGPKNGTLDEGRFTVKRGINDPKEVGLMVKYNNKTKLDVWAGPECNTLSGTDSTIFPPFIDDSEDIVSFSPDLCRSLGAKFRYKITYKGVPGNHYTADLGDMSANEDEKCYCPTPTTCLKKGAMDITKCAGAPIILTLPHYYLADPSYLDEVEGLHPEEEKHQIFLNFEPITGTPLGARKRLQFNIKSHPVKKIPFMKSLPTTMIPLMWIEEGLELDQKFIDILNANLFRVMKIVGVSKWVMMLLGLGMGGFGAFLYYKRKGEAGQPSEKSPTPKTVQVESISGKF

>AlinSNMP1b

MPSQKSESRNSESKMSQYPRVSQVSKPTKGSRATSPVFSNLMERMREMPTKIKEAPPRQFGKFGAAMVAGGVGFGWVAFPYILSFAISKMVNLAPGGEIHDIWKDIPQSLDFNIWIWNVTNPMEVQNGGKAVLQEVGPYRYIEWKKKVDLIDNPADDEITYSSLNTWYFQKDRSYPLTGDEIVTIPHLPLMSMLLVAEQDFPPAMMTVLNAAIPRIYGKLDSVFMQIKAKDLLFDGYPIDCTSRDLIGRTVCVAVKANSKPLVKNGRNKYLFSVLGTKNATPEDVRITVKKGTVNTYDIGKVVKVNGNPMNSVWKDECNVLDGTDATIFPPYRSADNVSIVAYATDICRSIRGTYIGEGSYNGVRGHQYAVDLGDMSSNPKDVCYCIKKCYKKGTVDLTKCQGAPLVGTLPHFYLADESYLDGVIGMKPDREKHQITFIMEPITGVPLLARKRFQFNVDMHPIRFVNVTKNIRPTLFPILWVEEALDLGPELMGFLQARLLTNLTLVDIVKWTLIVVGAGIGIMGIVKHQMEKEQRKKHERGASVSPAPSNASQERLVGQSAFRSDSEFSFKSSEMLMDPARLTGASKTTPPPLIPHPHIPTPPQVFTLERSLQERLSPEVEGIPPVEVPPSRLSVVTSVTPVEESAPAAGAQPGSKPASGKSKK

>AlinSNMP2a

MMRNGWTSVDLRMGNIHINRVLYLGAFGAVIFIIGLFFATSGTDMMINSKIKKGIVLEEGSEGLKRFQKTPFPLEFKVFLFNITNTDDVMMGGKPVLTEMGPYTYDLYKEKPELKFLKDGMIEYNMTYQFHFNAQKSRGSESDMVTGLNVPLLGTATMVEQTFPMGLGFLNNAIPFLFPNITDIFVTTTVKDLLFDGILLRCNYTSGPAMPICNGLKGRAPPTIWREEETKNYRFAMFRHKNKTSEGPYKVKTGKGDVTEVGQIVEYQHRQTLKNWDKNSSCTIIKGTDTTIFGPLKNPHDDLYIFVPDVCLSFTANYVNTSIQNGIPLNKYFAAEKNMASYSKDPDNLCRCAKDDEGVRHCLKDGVIDASPCQGAPVIMSNPHFLDADAEYQNAVVGLKPIEEKHKTFVMLEPKTGAPVEGRKRMQMNLKVKKVNSITLLENVTERIIPLLWIEEGTRLEGPLLQELQKLYHVMGLLGTFSWVLLVAGLVIMGIAGVLYLKVRHLFCFAGTQIVAPVDSSIGGAQKMNTFGVTNQGSDDYQEHGYPGTAIYPQLGDGQGKNGDLVHTVAHPQAR

>AlinSNMP2b

MPSLNFVNELLELGFPELLRDNYAMRLNRTVDELLFSGITTHCPPNASLSAATVCSILRHFPGLKSLQKYPNGDMNVGIMRFKNDTLSDTYEVYRGNHDFDKIGQIVTLNGQQSVDNWYGDECNKVAGSYGETLLKPFLTEDSTMKVYGSDLCSSLPVGFKETSSYEGVDSFKFGPQKKFLGSVVDYPENYCYCPGSIDGITLGQGCMKAGAMEFSACQAVPVVLSFPHFYKASSHFQNAVGGLDPDSDKHESYIHLEPITGIPLKGVKRIQINFQMKGTPAMKITKNARDTLIPFLWVEEVAALGDDQVNLLKDMLLKMLKILSIVRWVLIAVGSLMVLVGCVMSFLSARKEHRHQY

>AmelSNMP1.X1

MRFKKLIHDITTRFFHRGVHVILHSSFVKLQSFVTTGRKLHETLRVPVGRKCVTMKPKKLGIIGGSLLAFGILICAIAFPPFLRSQVKKQIALKDGSEMRELWSNFPVPLDFKIYLFNVTNPMEITAGEKPILEEVGPFFYDEYKQKVDLVDREEDDSLEYNLKATWFFNPSRSEGLTGEEELIVPHVLILSMIKLTLEQQPAAMGILNKAVDNIFKKPESVFVRAKAREILFDGLPVDCTGKDFASSAICSVLKEKDDALIADGPGRYLFSLFGPKNGTVLPERIRVLRGIKNYKDVGKVTEVNGKTKLDIWGEGDCNEFNGTDSTIFAPLLTEQDDIVSFAPDICRSMGARFDSYTKVKGINTYHYKADLGDMSSHPEEKCFCPSPDSCLTKNLMDLTKCVGAPLIASLPHLLGAEEKYLKMVDGLHPNEEEHGIAMDFEPMTATPLSAHKRLQFNLYLHKVAKFKLMKNFPECLFPIFWVEEGILLGDEFVKKLKTVFKTISIVGFMKWFTIVSGTCVSGAAAALFFKNKDKNKLDITKVTPQKGEEKKWPNQMTISTIQSAAVPPNLDAD

>AmelSNMP1.X2

MKPKKLGIIGGSLLAFGILICAIAFPPFLRSQVKKQIALKDGSEMRELWSNFPVPLDFKIYLFNVTNPMEITAGEKPILEEVGPFFYDEYKQKVDLVDREEDDSLEYNLKATWFFNPSRSEGLTGEEELIVPHVLILSMIKLTLEQQPAAMGILNKAVDNIFKKPESVFVRAKAREILFDGLPVDCTGKDFASSAICSVLKEKDDALIADGPGRYLFSLFGPKNGTVLPERIRVLRGIKNYKDVGKVTEVNGKTKLDIWGEGDCNEFNGTDSTIFAPLLTEQDDIVSFAPDICRSMGARFDSYTKVKGINTYHYKADLGDMSSHPEEKCFCPSPDSCLTKNLMDLTKCVGAPLIASLPHLLGAEEKYLKMVDGLHPNEEEHGIAMDFEPMTATPLSAHKRLQFNLYLHKVAKFKLMKNFPECLFPIFWVEEGILLGDEFVKKLKTVFKTISIVGFMKWFTIVSGTCVSGAAAALFFKNKDKNKLDITKVTPQKGEEKKWPNQMTISTIQSAAVPPNLDAD

>AmelSNMP2

MWSYQVCAIICVIFGIYACITNLFSDGLFSIKNAILKNLPLIKGKDMYDEWILPVNLIFKCYFFNVTNPDEVMEGNNPNLVEYGPFTYREVFEKQIVDVDEELDEIIYDVKSTFTFDKYASLNISKRDTVTILNPAYIGTISMASIIGLTTLPPSYIEKFGNNIPKLFPNRSSIFLKANPKEILFDGVKLTCNERKFPELSTICKTLKALRSPVLKEGEKEGVYYLSIFQRVNGTIRGRFSVNRGVNNISELGNIGSYNGRRVQTIWRTEKCNTVRGSDTITWAPLINPMPSVLSFIPDLCRSIEADYDKEVSIYGLIGSRFVMRERTWFLNQSQCYCLERNKVPNCLPQGLIDVSDCLVMLRYVMLQKVPIIMSEPHFLHGDPQLLMYALGLNPSEDLHETFIVIEPYTGTPLSGQKKIQLNLKLERQPVDLLSNISEGYFPLLWCANVRIFSKIIKLQY

>BmorSNMP1

MQLAKPLKYAAISGIVAFVGLMFGWVIFPAILKSQLKKEMALSKKTDVRKMWEKIPFALDFKIYLFNYTNAEDVQKGAVPIVKEVGPFYFEEWKEKVEVEENEGNDTINYKKIDVFLFKPELSGPGLTGEEVIVMPNIFMMAMALTVYREKPAMLNVAAKAINGIFDSPSDVFMRVKALDILFRGIIINCDRTEFAPKAACTTIKKEAPNGIVFEPNNQLRFSLFGVRNNSVDPHVVTVKRGVQNVMDVGRVVAIDGKTKMNVWRDSCNEYQGTDGTVFPPFLTHKDRLQSFSGDLCRSFKPWFQKKTSYNGIKTNRYVANIGDFANDPELQCYCDSPDKCPPKGLMDLYKCIKAPMFVSMPHYLEGDPELLKNVKGLNPNAKEHGIEIDFEPISGTPMVAKQRIQFNIQLLKSEKMDLLKDLPGTIVPLFWIEEGLSLNKTFVKMLKSQLFIPKRVVSVVCWCMISFGSLGVIAAVIFHFKGDIMHLAVAGDNSVSKIKPENDENKEVGVMGQNQEPAKVM

>BmorSNMP2

MLAKYTKTIFSVSVAFLVVSIVLATWGFPKIIRKQIQKNVQISNTSKMYDKWVKLPMPLDFKIYVFNVTNRDAINQGEKPNLKEIGPYVYKQYREKIILGYGDNDTIKYNLKKTFVFDPVASGDLREDDELTVINFSYMAAIISVQEMMPAAVGMINRALEQFFTNLTDPFQTVKVKDLFFDGLFLNCEGDNTALGLICGKIRAEKPPTMRISKSANGFYFSMFSHMNRTVSGPYEMVRGTENLSDLGHVISYQGKRIMSAWDDQYCGQLNGTDSTIFPPLEDGNIPEKLYTFEPDICRSLFASLVGKDTLFNISTYYYEISDMTLGSKSANPDNKCFCKRNWSVKHDGCLLMGVLNLAPCQGAPAIASLPHFYLGSDELADFFGDGIKPDKEKHNTYVHLDPITGVVIKGVKRLQFNIELRNVPSVPQLKEVPSGLFPLLWIEEGAEIPEWLRKEIMDSHTMLWYVDAARWLVLAVAVVAVLVSATLVARSAALIPWPRNSNSISFILGNSVNTSKVHS

>DmelSNMP1

MQVPRVKLLMGSGAMFVFAIIYGWVIFPKILKFMISKQVTLKPGSDVRELWSNTPFPLHFYIYVFNVTNPDEVSEGAKPRLQEVGPFVFDEWKDKYDLEDDVVEDTVSFTMRNTFIFNPKESLPLTGEEEIILPHPIMLPGGISVQREKAAMMELVSKGLSIVFPDAKAFLKAKFMDLFFRGINVDCSSEEFSAKALCTVFYTGEIKQAKQVNQTHFLFSFMGQANHSDSGRFTVCRGVKNNKKLGKVVKFADEPEQDIWPDGECNTFVGTDSTVFAPGLKKEDGLWAFTPDLCRSLGAYYQHKSSYHGMPSMRYTLDLGDIRADEKLHCFCEDPEDLDTCPPKGTMNLAACVGGPLMASMPHFYLGDPKLVADVDGLNPNEKDHAVYIDFELMSGTPFQAAKRLQFNLDMEPVEGIEPMKNLPKLILPMFWVEEGVQLNKTYTNLVKYTLFLGLKINSVLRWSLITFSLVGLMFSAYLFYHKSDSLDINSILKDNNKVDDVASTKEPLPSANPKQSSTVHPVQLPNTLIPGTNPATNPATHHKMEHRERY

>DmelSNMP2

MIHWSLIVSALGVCVAVLGGYCGWILFPNMVHKKVEQSVVIQDGSEQFKRFVNLPQPLNFKVYIFNVTNSDRIQQGAIPIVEEIGPYVYKQFRQKKVKHFSRDGSKISYVQNVHFDFDAAASAPYTQDDRIVALNMHMNAFLQVFEREITDIFQGFANRLNSRLNQTPGVRVLKRLMERIRGKRKSVLQISENDPGLALLLVHLNANLKAVFNDPRSMSVSTSVREYLFDGVRFCINPQGIAKAICNQIKESGSKTIREKSDGSLAFSFFGHKNGSGHEVYEVHTGKGDPMRVLEIQKLDDSHNLQVWLNASSEGETSVCNQINGTDASAYPPFRQRGDSMYIFSADICRSVQLFYQTDIQYQGIPGYRYSIGENFINDIGPEHDNECFCVDKLANVIKRKNGCLYAGALDLTTCLDAPVILTLPHMLGASNEYRKMIRGLKPDAKKHQTFVDVQSLTGTPLQGGKRVQFNMFLKSINRIGITENLPTVLMPAIWVEEGIQLNGEMVAFFKKKLISTLKTLNIVHWATLCGGIGVAVACLIYYIYQRGRVVEPPVK

>OasiSNMP1

MQLPVGLAAGGGGVFFLAVVAGWYGMPKLIHSQIANGLALKKGSDIRQMWSNFSDPIDFRVYILNLTNPEAVHRGEKPIVQEIGPYFYEEYKQKVKLRDHKEDDTVSYNNKVTWLFNRGKSAPGLTGDEMVTMPHPLLLGLLLTLERDKPGMLALVNKAIPP

>OasiSNMP2a

MLSARVCGCGRRGLWWGLAAGAVVLAAALVLRWAAFPAILTAKITQAVQIQDGSAAMERFVQLPQPLLYKAYLFNVTNPDQVSLGAKPALQEVGPYVYEEWRRRRDVTRLEDGSLSYRLETTFRFSPERSPGLSEDDEFTYLNVVMMGIVVQVAEDYASMLTMVEPVLSELLSGGAQLFLRATARQLLWDGVPTVDCRGNLSAVAVLACSALPSLLPPTVQQAQPGVYAFSFFGFKNGTARQWWRVDPGLSDVRSLGAVLSYDNSSRLKVWTPASSPCNDIRGTDSTIFPPFRDPKEPIVAFGADLCLSLGATWERSAEYMGVPGNRYTGEMPDMTGNPEHHCYCPSETSCLEKGALDLSPCAGAPVIATLPHFYLSSESYLRTVSGLQPTKEHHELFMVFESTTGSPMEARKRLQFNMFLHKINKIDLLANVPYALMPLIWVEEGLALEEKYVSTLRMLFKMQGIMSGVKWTMMVVGMGMAGAGGYLHFKRRKELVVGPAEPKKVAAGHAEPPGHPIRLESSHSRY

>OasiSNMP2b

MLSARVCGCGRRGLWWGLAAGAVVLAAALVLRWAAFPAILTAKITQAVQIQDGSAAMERFVQLPQPLLYKAYLFNVTNPDQVSLGAKPALQEVGPYVYEEWRRRRDVTRLEDGSLSYRLETTFRFSPERSPGVSEDDEFTYLNVVMMGIVVQVAEDYASMLTMVEPVLSELLSGGAQLFLRATARQLLWDGVPTVDCRGNLSAVAVLACSALPSLLPPTVQQAQPGVYAFSFFGFKNGTARQWWRVDPGLSDVRSLGAVLSYDNSSRLKVWTPASSPCNDIRGTDSTIFPPFITPNHTIHIFAHDICRSMHADFEREQEVSGVRGLRFVASRSLLRQGGRNACTCPEGHCLPSGAISVKECFRAPIAVSYPHFYQAEPEYLQYAEGLSPSKELHETFVVIEPESGTPLLGAKRLQFNMRAARVPQVPALANLTDGLFPLLWVEEGVELEEAELSQVRALFVARASLGGVAWAVLAGGVAALLFCAYRLARGRLRERNTTLALDKGAAAGGKLSVPTLGAAYPESAPRRASPPPAAPVDATNF

>SgreSNMP1

MQLPVGLAAGGGGVFFMAVVAGWYGMPKLISSQIASGLALKKGSDIRQMWSNFSDPIDFRVYVLNLTNPEAVHRGEKPIVQEIGPYFYEEYKQKVKLRDHKEDDTVSYNNKITWLFNQGKSAPGLTGDELVTLPHPLLLGLLLTLERDKPGMLALVNKAIPPLFRKPESIFVTAPVRNFLFDGIVINCTVTDFSAKALCTGLKKEAKELKREGDNFFFSFFGHKNGTVDAGRLRVKRGIQNIDDLGRVVAFNGEPKMSAWRGDPCNDLRGTDSTIFPPFRDPKEPIVAFGPDLCLSLGANWERKAEYMGVPGNRYTAELPDMKGNPEHHCYCPTEQTCLEKGTLDLSPCAGAPVIATLPHFYLASETYLQTVSGLQPTKENHELFMVFESTTGSPMEARKRLQFNMFLHKINKIDLLANVPYALMPLIWVEEGLALEEKYVSTLRMLFRMQGIMSGVKWTLMAVGMGMAGAGGYLHFKRRKELVVGPAEPKKVVAGHDTTGHPIRLESSHSRY

>SgreSNMP2

MLSARVCGCGRRGLWWGLAAGAALLAVALVLRWAAFPAILTAKIKQAVQLHDGSPAMERFVQLPQPLLYKVYLFNVTNPDEVEQGAKPVLQQVGPYVYEEWRRRRDVTRMANGSLDYRLETTYHFSPERSPGLSEDDEFTYLNVVMVGIVVQVSEDYSSLLSMVEPVLSELVPGGAQLFQRASARQLLWSGVPTVDCRGNLSAVATLACGALPSLLPATVQQTEPGVYVFSFFGFKNGTSKQWWRVDSGVEDVRTLGSVISYDNSSRLKVWSPSNSPCNEIRGTDSTLFPPFITPNDTIYIFAHDICRSMHAEYEREQDVSGVHGLRFVASGSLLRRGGPNACTCPDGRCLATGAISVRECFRAPIAVSFPHFYQASPEYLQYAEGLSPNKELHETFVVIEPETGTPLVGAKRLQFNMKAVRVSQVPALRNVSDGLFPLLWVEEGVELEEKQLSQVRALYVARASMGGVAWAVLAVGVAALLFCAVRLAKARVAERNRSLSLEKGVTAGGKLSVPTLGAAYPESATKRPSPPAAAPAASTAPAAPVDATHF

>TcasSNMP1

MIKGKVKSMINLNKGSEIRQMFVKVPFALDFKIYMFNVTNPMDVQKGALPVLKEVGPFCFEEWKEKVDLDDNDDEDVMFYNPKDTFYKANGPGCLDGSQMITMAHPLILGMVNTVVRTKPGAISLISKAINSIYGNPDSIFMTASAMDILFDGVVIKCGVKDFAGKAVCSQLKEAPDLRHVDENDLAFSFIGPKNATPGKRFKVLRGVKESHDVGRILEYDNKKEMEVWPTKECNQYKGTDGTVFPPYLTKEEGLASYAPDLCRSLVAVYSGDTKYDGIPVRIYTATLGDMSKNADEKCYCPTPDTCLKKGMMDLFKCAGVPVYVSLPHFYESDESYVKGVVGLNPNKKDHGIQILFESTTGGPVKAAKRLQFNMPLEPNPKLPIFANLPNTVLPLFWVEEGVALNNTFTKPLKDLFKIMKIVKIAKWLIMLGCLGGLGAAGYLYFSKKGEANITPVHKVKPAENGVSTLGGEVNHAMSDNEIEKY

>TcasSNMP1-like

MVKWQRQLKPGNEVRDFYIKLPIPLDFRVYFFNISNPEEVKQGEKPILKQIGPYCYDAYKEKINVEDDKDNDTLTYNPYDTYFFNQMRTGDLSQDDYVTILHPLTVGIVNAVATQKPQYLSAVNKALPVIFKENSSIYLTAKVREILFDGVLINCNVKDFSANAVCSQFKGQPAMVEVEKNIYSFSLLGSRNGSIPTRITIHRGVKNAADIGRVVTIDNKTDLDVWPEPECNAFRGTDGWVFPSFLEKEDGIWTVASDLCRSFKAQYVEDLKFHGVVVRKYFADLGDMSSNPAEKCFCPAPEKCLPKGVMDLTKCMKVPLYCTLPHFLRADEKLLQQVEGLSPELERHIIKIYFEPLTGTPMLGQRRIQFNLQLMPIPKVAMMKTVPEALHPILWIEEGVELEGFLLKKVTSVFTLLKLMTFVRYIMLGLSIQGILYGGYKLYQESKSKKVSPVQNGTTESKNHNQGKTGGIELPSMNKRNKENTKNA

>TcasSNMP2

MDIVNDALPFLYPGIKNIFVTNTVRNILFDGVTMSCGSDEVAMICDGLKKRRPPSIRPADNNKDYLVAMFHHMNGSVDGPYEMQRGLKDSSKGQVVGFKDNNMLTLWTGDCNTIQGTDLTLNPNLNDLPPKIYFFASDFCRSFSVKFDKELVYLGLKSYKFKNSNLFHIEKNCFCDKNPENEVPGCTPAGTMDVSPCTGSSVVLSQPHFLNAEKSLLDEAQGLAPNENRHGTFIIMEPKTGLALVVKTRFQMNVYLQDFEDVDLLANVSAGFFPLLWLENVWRCYLRLF
