# Supplementary material for: Identification and Expression Profile Analysis of Chemosensory Genes From the Antennal Transcriptome of Bamboo Locust (Ceracris kiangsu)
Source: Front Physiol. 2020 Sep 9;11:889. doi: 10.3389/fphys.2020.00889 (PMC7509195; doi:10.3389/fphys.2020.00889)
Supplement: TABLE S2 — Primers used for qRT-PCR. [file Table_2.docx]

**Table S2**  Primers used for qRT-PCR.

| **Primer name** | **Nucleotide sequence (5′–3′)** | **Primer name** | **Nucleotide sequence (5′–3′)** |
| --- | --- | --- | --- |
| CkiaOR1-F | CACACAGCAAATAAGACAGC | CkiaOR64-F | CCGACTTGGTGAAAAACATT |
| CkiaOR1-R | GAAATAGACTAAGACAAACC | CkiaOR64-R | CCTTCTGCAACAGGAGAGAA |
| CkiaOR2-F | ATACGCAATACAAGCCCT | CkiaOR65-F | TGACTCACTCTATTTTCGT |
| CkiaOR2-R | GATCCACTCGCCTGAAAAC | CkiaOR65-R | CATTTTATACATTGCCACT |
| CkiaOR3-F | ATACGCAATACAAGCCC | CkiaOR66-F | AGTCGGCGTTAAACTACTCT |
| CkiaOR3-R | GATCCACTCGCCTGAAAAC | CkiaOR66-R | TGTTATTTTCATGGCTCTTC |
| CkiaOR4-F | GCCTGCCACTGGTACTTCG | CkiaOR67-F | CTGGAAAACGCTATGAGTA |
| CkiaOR4-R | ACAGCATCAGCAGCGAC | CkiaOR67-R | CGGGAAGGTAAACATGAAC |
| CkiaOR5-F | TACATCCCAAAAAAGCCAA | CkiaOR68-F | GTACAGTGCTCTAGCGAGGA |
| CkiaOR5-R | ACAGGTAGAGAGCATCGAA | CkiaOR68-R | CCGAAAGAATGCAGACAAAC |
| CkiaOR6-F | CCTCTACCTGGCGTCCTA | CkiaOR69-F | AGAACATACGACACCATCAA |
| CkiaOR6-R | TTTTCTCTCCCCGTTATCC | CkiaOR69-R | GAAACAACGCAACAAATCCG |
| CkiaOR7-F | CTCAGGCAGTCGCACCAG | CkiaOR70-F | TCTTTTCCTCATCGTTACC |
| CkiaOR7-R | CAGCAGGAACGTGTCGCAC | CkiaOR70-R | CTCCTTTCCCAGAATTCTT |
| CkiaOR8-F | CTGGGCAAGAGCGGGGA | CkiaOR71-F | TGAAGAACATCCAGCACCAT |
| CkiaOR8-R | CAGCAGCAGGCACAAGAC | CkiaOR71-R | CCGTCTACAAATCCAATCC |
| CkiaOR9-F | ACTCCTACAGCGTCAGCCT | CkiaOR72-F | TTCTTCCAGTCCACTTG |
| CkiaOR9-R | TGGACCTTTTCCGATACA | CkiaOR72-R | TTCCTCAGCATACCTCT |
| CkiaOR10-F | ATCGCGGGCTGCTTGTGG | CkiaOR73-F | GCCTCCGCCATAACCAAAAT |
| CkiaOR10-R | TATGGTCATCGGGGTGGGG | CkiaOR73-R | CAACACCCGCAGAGACCTCT |
| CkiaOR11-F | GGAGAAAGAGGGAGCGGA | CkiaOR74-F | CATCCAAATAGAAAGATGGG |
| CkiaOR11-R | ACAGAACCAAGGGAGAGGC | CkiaOR74-R | CTGTTGAGTATAAGCAGGCA |
| CkiaOR12-F | CTACTACCGACTACGAA | CkiaOR75-F | CCTCCTGTCTCTCTCCTGTG |
| CkiaOR12-R | AGCAAACAGAGATACCAA | CkiaOR75-R | CATTTTTCTTTTTGGCTTCT |
| CkiaOR13-F | AGAAACCTTCGCAGCCTCA | CkiaOR76-F | AGTGTCCAAGGGAGTGTGCT |
| CkiaOR13-R | ACCTAAACACCGCTCCA | CkiaOR76-R | CCGTTGTCTCTTTCAGCGT |
| CkiaOR14-F | GAGATATCCGAGGGAGACG | CkiaOR77-F | GGCAATAGTTAGTTTGTG |
| CkiaOR14-R | CTGGAAGCAAGTGGAGGC | CkiaOR77-R | TCAGCTTGTAGTACGAGGTG |
| CkiaOR15-F | TCACCATCAAGCAATTATC | CkiaOR78-F | CTATGCCAATTCACAGAAAA |
| CkiaOR15-R | ACATCTGGAAGCAAGCA | CkiaOR78-R | TGGAAAGCAACACAAACACT |
| CkiaOR16-F | TAAAACATTAGCACCCAGT | CkiaOR79-F | ATGTACGACCAGTTAGTGG |
| CkiaOR16-R | CCATCATGAAAAGCATAC | CkiaOR79-R | AGTGACGAAGATGTTGAG |
| CkiaOR17-F | CACATCGGAAAGTGAGAAA | CkiaOR80-F | TTGTCCTTCTCATGCTCAGC |
| CkiaOR17-R | CGAACAGTAGGCAGACAG | CkiaOR80-R | TTCGTCAAATTGGTCTCTC |
| CkiaOR18-F | TCTACGGAGAACTGAGGGA | CkiaOR81-F | ATAAAGCACTTCAAATGGC |
| CkiaOR18-R | GATATTGGTGGCAAACTGG | CkiaOR81-R | AACGAGCAGATTAGAACACA |
| CkiaOR19-F | TGGCGGGGCTGTGGTACTT | CkiaOR82-F | CTTCTCTTTGCGTTGGGTT |
| CkiaOR19-R | GGCGGTTGACGATGCTGA | CkiaOR82-R | CCGCGTCAATTTATTGTTG |
| CkiaOR20-F | AGTTAGTTGGCGATTTGGA | CkiaOR83-F | CGAGAACCGAGGAAGAAGGA |
| CkiaOR20-R | AAGTGTTGTTTGCTGGTTG | CkiaOR83-R | GTCAGGGTCTGGTCAGCAAG |
| CkiaOR21-F | AGTTAGTTGGCGATTTG | CkiaOR84-F | TCTTTATTCTCCTGTTTGTC |
| CkiaOR21-R | AAGTGTTGTTTGCTGGTT | CkiaOR84-R | GTTTCTCACTCTGATCCGTT |
| CkiaOR22-F | CGGACCTATTCTTTGCTTC | CkiaOR85-F | TGCTGCTTCGTTATCTTTA |
| CkiaOR22-R | TTTCCTGTGCGTTATCTT | CkiaOR85-R | ATCGGATGTCCTCACTGTTT |
| CkiaOR23-F | TTACGAAACATACGAAAAG | CkiaOR86-F | GCATGGATTTCGGATTTC |
| CkiaOR23-R | TCAACCATAAGCAGAACTC | CkiaOR86-R | GGTGTATTTTTCTCTGGT |
| CkiaOR24-F | AGTTTTAGTCGTTTCTCG | CkiaOR87-F | CGCACTCTCGCTTTCGTCCC |
| CkiaOR24-R | CTTATGTACCACCTTTCGC | CkiaOR87-R | CCACCAGCATCACCGCCTT |
| CkiaOR25-F | GTGGAGAGTGAGCGGTTG | CkiaOR88-F | AAGATATTGAGCCTGTT |
| CkiaOR25-R | TGGCGTAGTAGGAGTAAGA | CkiaOR88-R | CTGTGTAGTGATCCTTTGAC |
| CkiaOR26-F | CGGAGGGACACGACACAAG | CkiaOR89-F | CACCCACCAGAAAATTACCA |
| CkiaOR26-R | ACAGCACCAACCGCAAAA | CkiaOR89-R | ACGCAGACTTCATCTCCAAA |
| CkiaOR27-F | GCTTCTTCGCCACCACC | CkiaOR90-F | ACATCAAAGGAATAGAAG |
| CkiaOR27-R | TCCGTCGATTCCTTGCATT | CkiaOR90-R | TGAAGTAAATGAGAAAGA |
| CkiaOR28-F | GAATTTTCTGAGCTTCGG | CkiaORCO-F | TGATGTACGTGGTGGCGGTG |
| CkiaOR28-R | CCTTCTCTTTTCGTTATC | CkiaORCO-R | ATGGTCTCGTTGGCGTTGT |
| CkiaOR29-F | AGCGTCGTAGCAGTGTGTG | CkiaOBP1-F | TGGGCACGCCTCTACAAC |
| CkiaOR29-R | CATCTGTAGAGCCGGG | CkiaOBP1-R | TCAAATGCACGAACTACC |
| CkiaOR30-F | CTGAACTGTGCGAGTGTG | CkiaOBP2-F | AGCGCTCCTAGTACTCGC |
| CkiaOR30-R | TCTGGGTTGAATGTTATC | CkiaOBP2-R | TCAAACTCTGGGGCAAAC |
| CkiaOR31-F | GCCTATCGAGCTTAGGTGA | CkiaOBP3-F | TACCTGGCTCTCCTAGTT |
| CkiaOR31-R | GGATGTGTTGGTGTGTTT | CkiaOBP3-R | TCCATCATACCCTTTATT |
| CkiaOR32-F | CTTCTTTGTGTCTTCGGTG | CkiaOBP4-F | GTCGCTGCCGTTCTGTTG |
| CkiaOR32-R | AACATTTCTCTTGCGTGA | CkiaOBP4-R | GGTCTGGAAGGCTTTGTT |
| CkiaOR33-F | AGATTCCGTTATGAATCCA | CkiaOBP5-F | TGTTCACCACTGGCTCTC |
| CkiaOR33-R | AAAAATAGTGCTGCTGC | CkiaOBP5-R | ATTTACCAGCTTGGCGTC |
| CkiaOR34-F | GCAGTGGAGACGGAGAGGA | CkiaOBP6-F | ATTCCCCATTCCTAAAGC |
| CkiaOR34-R | GTGGTAGGCCCAGGAGCT | CkiaOBP6-R | TCCAAGTCTCGCCAGTTT |
| CkiaOR35-F | CCTTCATCAACACCCACC | CkiaOBP7-F | CAATCCTACTTGTCTCCG |
| CkiaOR35-R | CCTCACTGACTGACCCTCG | CkiaOBP7-R | TATTTTGTCTCATGGCCC |
| CkiaOR36-F | CTTCTGAGGCTGGTGAG | CkiaOBP8-F | TCACAACTGACATCCCCA |
| CkiaOR36-R | TGGCTAGTAGGGGTAGTGC | CkiaOBP8-R | TGACTTCACCCCTAGACC |
| CkiaOR37-F | GCTGGTATTCGAGAGAAG | CkiaOBP9-F | TCAAGGACGCCATTCTCAA |
| CkiaOR37-R | AAACAGAGAGTGATGAT | CkiaOBP9-R | TCACCATGCACCCAACCAG |
| CkiaOR38-F | GTGGCAGCAGAGAGAGAAT | CkiaOBP10-F | CCAGCAAAAGTGTTGGTTC |
| CkiaOR38-R | ATGAAGAGCAGGAAACAGT | CkiaOBP10-R | GTATAGGTTGTCTCGTCGA |
| CkiaOR39-F | AGCAGAGTGCGGCGGTGT | CkiaOBP11-F | TGGAGTCTTTGATGCTGAT |
| CkiaOR39-R | ATGAATGTGGGGCGGTTG | CkiaOBP11-R | GATTTATTTTGAATGCTGT |
| CkiaOR40-F | TCAACCACATACCATGGGA | CkiaOBP12-F | ATTTTGTTGCTTGTTGTGG |
| CkiaOR40-R | CTGCTCGGTGACGAAAAT | CkiaOBP12-R | ATCTGGTATTGTGCCGTTA |
| CkiaOR41-F | GATACGCACAAGGAAATA | CkiaOBP13-F | ATCAGGATCTGAGCTTAAC |
| CkiaOR41-R | TGGTACAAAGTCAGACACG | CkiaOBP13-R | CTGCCCCATACCACCATTG |
| CkiaOR42-F | GGCAACTCTATTCTCAAG | CkiaIR1-F | GGCGAGACGAGGTGTTGG |
| CkiaOR42-R | GCTCAGGTAAATCCCCACC | CkiaIR1-R | AGGGCGGGTAGTGGAAGG |
| CkiaOR43-F | TCGGCAGTGGGAAGACAG | CkiaIR2-F | TCCTACCTGCTCACCTC |
| CkiaOR43-R | GCGAAGAAGGCGTGGTACA | CkiaIR2-R | CCGTCTCCGTCCGTAT |
| CkiaOR44-F | TAATCCAGCAGCGGAAGA | CkiaIR3-F | ACATAACACAAACTCTT |
| CkiaOR44-R | TCACACACAGGACAAGAGC | CkiaIR3-R | AACCACATTACTGTCAA |
| CkiaOR45-F | CACCAGTGGGACAACAACA | CkiaIR4-F | TGATGTTACTGCTGGTGTG |
| CkiaOR45-R | GACCATAGCCGACACGAAG | CkiaIR4-R | TACGAGTGAGGCTCTTT |
| CkiaOR46-F | ACAAGGAGAAAGGCGGAT | CkiaIR5-F | CTGGGACCTGTGCGATAAG |
| CkiaOR46-R | GAACGCTGAAAGCAAACTG | CkiaIR5-R | GTGCGGTGAAGTCGATGA |
| CkiaOR47-F | GTATTCCGCCTTACTCTGG | CkiaIR6-F | CTGCGGAAGGTGGAGGACA |
| CkiaOR47-R | TAGTTGCTGTTGTTGTCCC | CkiaIR6-R | GCGAAGCCGAACGAAGGTG |
| CkiaOR48-F | TGCTGTAGTGTCCTCGTC | CkiaIR7-F | TACGAAATGGACGGAGCA |
| CkiaOR48-R | TGCTTCTGCCTGCTCGG | CkiaIR7-R | GACTTGGGATGGCGAAACA |
| CkiaOR49-F | TCAAAATCGTTTCCTGTCG | CkiaIR8-F | ACCATGTACCCGCTCATC |
| CkiaOR49-R | ATTGGGCTCATTGTGTCCT | CkiaIR8-R | CCGCTCTCCCACACCTTC |
| CkiaOR50-F | ACCAGTACGCTCGTCATTT | CkiaIR9-F | GCACATACTGAAAACACG |
| CkiaOR50-R | GCAGCCTCTCACTCTCCAA | CkiaIR9-R | TGACTTCCAGACAAACT |
| CkiaOR51-F | GGAGGGCGAAGTACGAGTA | CkiaIR10-F | GTATTGAGGAACCAGAAAC |
| CkiaOR51-R | TCTTGGAGAGCGAGAAGCT | CkiaIR10-R | GAAACCATGATAAGAGT |
| CkiaOR52-F | GACCACAAGACGACAACCCA | CkiaIR8a-F | TCTAGGTACAGGGTTTGG |
| CkiaOR52-R | TGAAACAGATGACGAGCGAG | CkiaIR8a-R | AGTCAGGTTGCAGTTTT |
| CkiaOR53-F | CATGCTTCTGATCTGTGT | CkiaIR25a-F | TGATGAAAAAGCCAAAA |
| CkiaOR53-R | TCTGCTCTATGAGGTTGTTG | CkiaIR25a-R | CTGTAGGGACTCCAACGA |
| CkiaOR54-F | GGAGGTGGTGTTTAGGTGG | CkiaIR73b-F | GCACTTTGATTGGACAAG |
| CkiaOR54-R | TGCGTGTAGGAGAAAGGGT | CkiaIR76b-R | GAGAACTGCGGCAGATAC |
| CkiaOR55-F | ATTCGTCGCCATAGCCAATA | CkiaCSP1-F | TGTTGTTAGCGTGCCTGG |
| CkiaOR55-R | CGTCCCAAACCAGCAGTAC | CkiaCSP1-R | TTCTTGAGCAAGCGGTCGT |
| CkiaOR56-F | GCTTCTTCTGGTCCCTCATC | CkiaCSP2-F | GGTACTCCTCTCGGCTTTGG |
| CkiaOR56-R | TCATATTCTCCTCCATTT | CkiaCSP2-R | TTGAGCAGGCGGTCGTTGGC |
| CkiaOR57-F | TTCCAGAAGCGGTCGGGCG | CkiaCSP3-F | TTGCACTTTCGCTGCTC |
| CkiaOR57-R | GGCGATTTCGTAGTAGGGG | CkiaCSP3-R | GCCTCAATGCGGTCCTTG |
| CkiaOR58-F | AGCTCAACAATGCTCTCT | CkiaCSP4-F | AGGACAAGCAGAGCAAAGAC |
| CkiaOR58-R | ATTCTTCTCGCTCGTATCCC | CkiaCSP4-R | TAGACACGTGCGCGAAGA |
| CkiaOR59-F | GACGCCCAGGAACACCACG | CkiaCSP5-F | GCTGCTGCCAGAAATGCTCG |
| CkiaOR59-R | TCCAGCAGCAGCGACAGCAG | CkiaCSP5-R | GAGTTCGCCAGTGGGGTCG |
| CkiaOR60-F | CGGTCGAAGGAATGGTGAAC | CkiaCSP6-F | GCAGACAGAGTGCTCCAA |
| CkiaOR60-R | TACTGGGCGAGAGTGGGTAG | CkiaCSP6-R | ATCATCATCCTCATCATAT |
| CkiaOR61-F | ATGAGGCGGTCTATGTGGTC | CkiaSNMP1-F | CCTACGCTCTCATGCCACTC |
| CkiaOR61-R | AGGGTCGTGGTTTATGGTGT | CkiaSNMP1-R | CCATCACCATCATCGTCCA |
| CkiaOR62-F | ATTCTCAGTCGCGCCACTTC | CkiaSNMP2-F | CGTCACCAACCCCGACCAGG |
| CkiaOR62-R | CCACAATCTCTCCGTCCTCC | CkiaSNMP2-R | GGAGAGCGAGCCGTCCCACA |
| CkiaOR63-F | GCACGCGAATTGATGTATTA |  |  |
| CkiaOR63-R | GCAGATGGTCACCATGTTTA |  |  |
