# Supplementary material for: Identification and Expression Profile Analysis of Chemosensory Genes From the Antennal Transcriptome of Bamboo Locust (Ceracris kiangsu)
Source: Front Physiol. 2020 Sep 9;11:889. doi: 10.3389/fphys.2020.00889 (PMC7509195; doi:10.3389/fphys.2020.00889)
Supplement: TABLE S3 — The summary of the Illumina sequencing data. [file Table_3.docx]

| **Sample** | **Raw Reads** | **Clean reads** | **Clean bases** | **Error(%)** | **Q20(%)** | **Q30(%)** |
| --- | --- | --- | --- | --- | --- | --- |
| MA1 | 45,604,660 | 44,695,370 | 6.7G | 0.02 | 97.75 | 93.42 |
| MA2 | 48,043,646 | 47,127,276 | 7.07G | 0.01 | 97.83 | 93.56 |
| MA3 | 40,654,174 | 39,458,482 | 5.92G | 0.02 | 97.54 | 93.17 |
| FA1 | 47,223,384 | 46,211,960 | 6.93G | 0.01 | 97.88 | 93.71 |
| FA2 | 40,818,656 | 39,947,564 | 5.99G | 0.02 | 97.54 | 93.14 |
| FA3 | 49,180,560 | 48,217,648 | 7.23G | 0.01 | 97.93 | 93.22 |

**Table S3**  The summary of the Illumina sequencing data.

Note: FA: female antennae; MA: male antennae . Representation of three biological repeats with Arabic numbers 1, 2 and 3.
