# Supplementary material for: Identification and Expression Profile Analysis of Chemosensory Genes From the Antennal Transcriptome of Bamboo Locust (Ceracris kiangsu)
Source: Front Physiol. 2020 Sep 9;11:889. doi: 10.3389/fphys.2020.00889 (PMC7509195; doi:10.3389/fphys.2020.00889)
Supplement: TABLE S4 — Length distribution and quality metrics of the C. kiangsu transcripts and unigenes. [file Table_4.docx]

**Table S4**  Length distribution and quality metrics of the *C. kiangsu* transcripts and unigenes.

|  | Length Range | | | | Total Number | Max Length (bp) | Mean Length (bp) | N50 Length (bp) |
| --- | --- | --- | --- | --- | --- | --- | --- | --- |
|  | 500- | 500-1000 | 1000-2000 | 2000+ |  |  |  |  |
| Transcript | 19,299 (30.33%) | 16,067 (25.25%) | 5,093 (23.72%) | 13,172 (20.70%) | 63,631 | 35,159 | 1,265 | 2,011 |
| Unigene | 10,649 (27.19%) | 10,191 (26.02%) | 10,011 (25.56) | 8,315 (21.23%) | 39,166 | 35,159 | 1,498 | 2,259 |
