# Supplementary material for: Identification and Expression Profile Analysis of Chemosensory Genes From the Antennal Transcriptome of Bamboo Locust (Ceracris kiangsu)
Source: Front Physiol. 2020 Sep 9;11:889. doi: 10.3389/fphys.2020.00889 (PMC7509195; doi:10.3389/fphys.2020.00889)
Supplement: TABLE S5 — The summary of functional annotation of C. kiangsu transcriptomes. [file Table_5.docx]

**Table S5** The summary of functional annotation of *C. kiangsu* transcriptomes.

| **Anno Database** | **Number** | **Percentage** |
| --- | --- | --- |
| Annotated in Nr | 19,438 | 49.63% |
| Annotated in Nt | 10,845 | 27.69% |
| Annotated in GO | 10,465 | 26.72% |
| Annotated in COG | 9,889 | 25.25% |
| Annotated in KEGG | 12,353 | 31.54% |
| Annotated in Swissprot | 13,892 | 35.47% |
| Annotated in Pfam | 18,949 | 48.38% |
| All Annotated | 23,241 | 59.34% |
