# Supplementary material for: Identification and Expression Profile Analysis of Chemosensory Genes From the Antennal Transcriptome of Bamboo Locust (Ceracris kiangsu)
Source: Front Physiol. 2020 Sep 9;11:889. doi: 10.3389/fphys.2020.00889 (PMC7509195; doi:10.3389/fphys.2020.00889)
Supplement: TABLE S6 — Conserved domains of odorant binding proteins in C. kiangsu. [file Table_6.docx]

**Table S6** Conserved domains of odorant binding proteins in *C. kiangsu*.

| **Proteins** | **Domains** | **Position (AA)** | **Domain definition** | **Interpro family** | **E-value** |
| --- | --- | --- | --- | --- | --- |
| CkiaOBP1 | 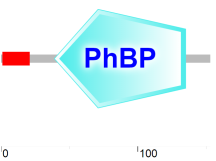 | 39–135 | Insect pheromone/odorant binding protein domains | PBP_GOBP (IPR006170) | 9.61E-16 |
| CkiaOBP2 | 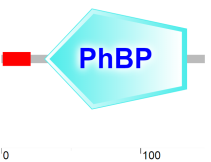 | 31–133 | Insect pheromone/odorant binding protein domains | PBP_GOBP (IPR006170) | 2.87E-14 |
| CkiaOBP3 | 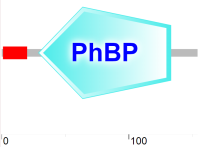 | 29–133 | Insect pheromone/odorant binding protein domains | PBP_GOBP (IPR006170) | 5.49E-01 |
| CkiaOBP4 | 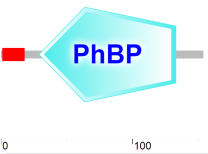 | 29–133 | Insect pheromone/odorant binding protein domains | PBP_GOBP (IPR006170) | 3.98E-01 |
| CkiaOBP5 | 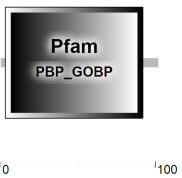 | 3–92 | PBP/GOBP family domain | PBP_GOBP (IPR006170) | 2.40E-13 |
| CkiaOBP6 | 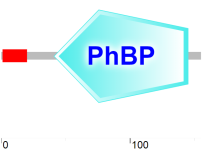 | 41–145 | Insect pheromone/odorant binding protein domains | PBP_GOBP (IPR006170) | 6.35E-07 |
| CkiaOBP7 | 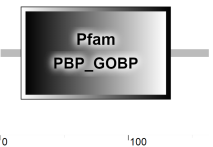 | 16–133 | PBP/GOBP family domain | PBP_GOBP (IPR006170) | 4.90E-08 |
| CkiaOBP8 | 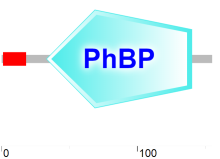 | 33–140 | Insect pheromone/odorant binding protein domains | PBP_GOBP (IPR006170) | 6.28E-02 |
| CkiaOBP9 | 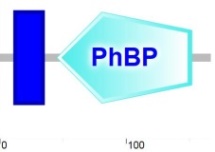 | 46–161 | Insect pheromone/odorant binding protein domains | PBP_GOBP (IPR006170) | 2.68E-01 |
| CkiaOBP10 | 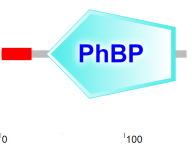 | 37–141 | Insect pheromone/odorant binding protein domains | PBP_GOBP (IPR006170) | 3.94E-03 |
| CkiaOBP11 | 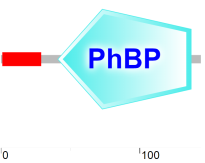 | 41–137 | Insect pheromone/odorant binding protein domains | PBP_GOBP (IPR006170) | 1.83E-21 |
| CkiaOBP12 | 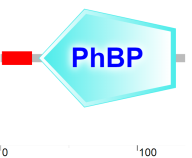 | 29–128 | Insect pheromone/odorant binding protein domains | PBP_GOBP (IPR006170) | 1.04E-01 |
| CkiaOBP13 | 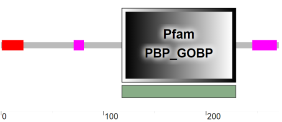 | 118–229 | PBP/GOBP family domain | PBP_GOBP (IPR006170) | 2.70E-06 |
