# Supplementary material for: Identification and Expression Profile Analysis of Chemosensory Genes From the Antennal Transcriptome of Bamboo Locust (Ceracris kiangsu)
Source: Front Physiol. 2020 Sep 9;11:889. doi: 10.3389/fphys.2020.00889 (PMC7509195; doi:10.3389/fphys.2020.00889)
Supplement: TABLE S7 — Conserved domains of chemosensory proteins in C. kiangsu. [file Table_7.docx]

**Table S7** Conserved domains of chemosensory proteins in *C. kiangsu*.

| **Proteins** | **Domains** | **Position (AA)** | **Domain definition** | **Interpro family** | **E-value** |
| --- | --- | --- | --- | --- | --- |
| CkiaCSP1 | 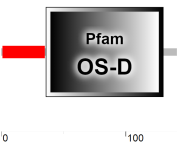 | 36–130 | Insect pheromone-binding family, A10/OS-D | IPR005055 OS_D_A10/PebIII | 1.40E-34 |
| CkiaCSP2 | 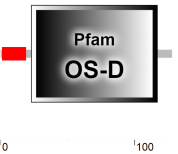 | 23–117 | Insect pheromone-binding family, A10/OS-D | IPR005055 OS_D_A11/PebIII | 1.10E-35 |
| CkiaCSP3 | 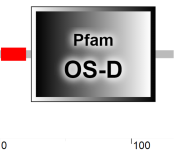 | 24–118 | Insect pheromone-binding family, A10/OS-D | IPR005055 OS_D_A12/PebIII | 3.90E-22 |
| CkiaCSP4 | 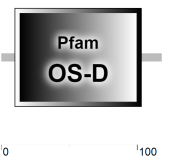 | 10–103 | Insect pheromone-binding family, A10/OS-D | IPR005055 OS_D_A13/PebIII | 8.10E-25 |
| CkiaCSP5 | 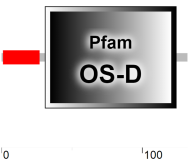 | 31–124 | Insect pheromone-binding family, A10/OS-D | IPR005055 OS_D_A14/PebIII | 3.30E-26 |
| CkiaCSP6 | 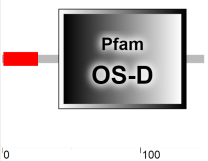 | 41–133 | Insect pheromone-binding family, A10/OS-D | IPR005055 OS_D_A15/PebIII | 3.20E-34 |
